# Transient Triamidoamine Neptunium(V)–Mono(Imido) Complexes: C–H Activations and Hydrogen Atom Transfer Driven by Effective Nuclear Charge

**DOI:** 10.1021/jacs.6c00505

**Published:** 2026-05-19

**Authors:** Michał S. Dutkiewicz, Iskander Douair, Jingzhen Du, Conrad A. P. Goodwin, Leonardo Tacconi, Mauro Perfetti, Andrew J. Gaunt, Samuel M. Greer, Benjamin W. Stein, Roberto Caciuffo, Eric Colineau, Jean-Christophe Griveau, John A. Seed, Attila Kovács, Brian L. Scott, Ashley J. Wooles, Laurent Maron, Olaf Walter, Stephen T. Liddle

**Affiliations:** † Department of Chemistry and Centre for Radiochemistry Research, 5292 The University of Manchester, Oxford Road, Manchester M13 9PL, U.K.; ‡ European Commission, Joint Research Centre, Postfach 2340, 76125 Karlsruhe, Germany; § LPCNO, CNRS and INSA, Université Paul Sabatier, 135 Avenue de Rangueil, Toulouse 31077, France; ∥ Chemistry Division, 5112 Los Alamos National Laboratory, Los Alamos, New Mexico 87545, United States; ⊥ Department of Chemistry Ugo Schiff, 9300 University of Florence, Via della Lastruccia 3, 50019 Sesto Fiorentino, Italy; # Materials Physics and Applications Division, 5112 Los Alamos National Laboratory, Los Alamos, New Mexico 87545, United States

## Abstract

Metal-mono­(imido) linkages have been known for seven decades, and they are found in transition metal, main group, lanthanide, thorium, and uranium complexes. However, transuranium-mono­(imido) complexes remain unknown in any scenario. Here, we present evidence for transient neptunium­(V)–mono­(imido) complexes. Treatment of [Np^III^(Tren^TIPS^)] (**1**, Tren^TIPS^ = {N­(CH_2_CH_2_NSiPr^i^_3_)_3_}^3–^) with N_3_R (R = SiMe_3_; 1-adamantyl, Ad) results in N_2_ evolution and dark purple solutions consistent with the formation of [Np^V^(Tren^TIPS^)­(NR)] (**3NpNR**). However, solutions of **3NpNR** rapidly turn orange, where for R = SiMe_3_ the isolated 1:1 products are [Np^IV^(Tren^TIPS^)­{N­(H)­SiMe_3_}] (**4a**) and [Np^IV^(Tren^TIPS‑2H^)­{N­(H)­SiMe_3_}] (**4b**, Tren^TIPS‑2H^ = {N­(CH_2_CH_2_NSiPr^i^_3_)_2_(NCH_2_CH_2_NSiPr^i^_2_C­[Me]=CH_2_)}^3–^). The latter contains a dehydrogenated-Pr^i^ vinyl functionality accounting for the source of the two amido H atoms. The reaction for R = Ad proceeds similarly, but only [Np^IV^(Tren^TIPS^)­{N­(H)­Ad}] (**5a**) could be unequivocally confirmed, though its isolation suggests generality of the imido-to-amido functional group transformation. Complexes **4a**/**4b** exhibit slow relaxation of their magnetization, adding to the small number of transuranium single ion magnets. Experimental and computational analysis suggests that the amido products are formed by C–H activation and two sequential hydrogen atom transfer reactions involving a three-step proton-coupled electron-transfer sequence of H^•^ radical abstraction, electron transfer, then another H^•^ radical abstraction step. In contrast to transient **3NpNR**, the 5f^2^ uranium­(IV)-imido complex [K­(2.2.2-cryptand)]­[U^IV^(Tren^TIPS^)­(NSiMe_3_)] (**8UNSiMe**_**3**_) is robust, even in boiling THF, suggesting the transience of 5f^2^ **3NpNR** is not due to the 5f^n^-count but the increased effective nuclear charge of neptunium vs uranium. This work highlights divergence of uranium- and neptunium-imido stabilities, emphasizing that the latter is an inherently challenging synthetic target.

## Graphical abstract

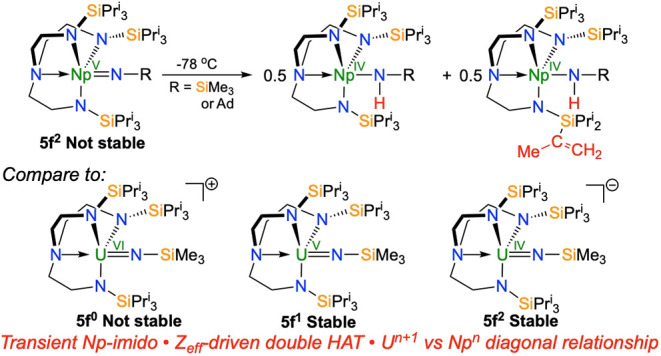

## Introduction

Metal–ligand (M–L) multiple bonding is a fundamentally important area of chemistry due to its impact on structure-bonding concepts and reactivity in small molecule transformations and catalysis.^1^ In recent times thorium- and uranium-L multiple bonding have undergone transformative developments; however, the situation is far less developed for transuranium elements.^2–11^ This is in part due to the challenges of working with such radioisotopes, including their availabilities, specific-activities, and access to suitable facilities, but also due to periodic trends.^12^ Moving left to right in the actinide (An) series the effective nuclear charge steadily increases because of poor shielding by the angular 5f-orbitals that are occupied and the rather diffuse nature of the usually vacant and higher-lying 6d-orbitals. Consequently, lower oxidation states are increasingly favored after uranium,^13–17^ but mid- or high-oxidation states are usually required for M–L multiple bonds to accommodate the M–L linkage and often present anionic ancillary coligands. It is hence the case that this dichotomy makes it inherently far more challenging to assemble isolated M–L multiple bonds at transuranium elements compared to uranium.

It is striking that the high oxidation state M–L multiple bond chemistry of the transuranium elements is dominated by bis­(oxo) actinyls, AnO_2_^n+^ (An = Np, Pu, Am), tetra­(oxo) actinyls, [An^VII^O_4_(OH)_2_]^3–^ (An = Np, Pu), and bis­(imido) [Np^V^(NDipp)_2_(Cl)­(bipy^tBu2^)] (Dipp = 2,6-di-*iso*-propylphenyl, bipy^tBu2^ = 4,4′-di-*tert*-butyl-2,2′-bipyridine ),^18–20^ where the latter is actinyl-like due to the isoelectronic and isolobal relationship between O^2–^ and RN^2–^. For these complexes the immutable effective nuclear charge issue is overcome by assembling two or more trans M-L multiple bond linkages at the transuranium element combined with the inverse-trans-influence (ITI),^21–23^ the latter being the phenomenon where strong donor ligands at actinide ions mutually stabilize each other due to the involvement of 5f- and 6p-orbitals in the bonding, which is opposite to the trend in transition metal chemistry where destabilization would normally occur. Low oxidation state An^III^=CR_2_ (An = Np, Pu) multiple bonds have been reported,^24,25^ but these result in part from the pincer-chelate ligands pinning the diphosphoniumalkylidene to the An-centers. The sole exception to date in the transuranium arena is oxidation of the neptunium(III) complex [Np^III^(Tren^TIPS^)] (**1**, Tren^TIPS^ = {N­(CH_2_CH_2_NSiPr^i^_3_)_3_}^3–^) by N_2_O to give the neptunium­(V)-mono­(oxo) complex [Np^V^(Tren^TIPS^)­(O)] (**2**),^26^ which contains a terminal, isolated An-L multiple bond and under an inert atmosphere is also a stable example of neptunium­(V)—indefinitely as a solid and for days in solution. The stability of **2** is due to a combination of the quadridentate Tren^TIPS^ ligand, which imparts thermodynamic and kinetic stability,^27^ and the hard oxo, where additionally a stabilizing ITI seems to operate due to the trans R_3_N→Np^V^=O linkage in **2**. More recently, the neptunium­(V) complex [Np^V^{NP­(Bu^t^)­[N­(C_2_H_4_)_2_]_2_}_4_]­[B­(C_6_F_5_)_4_] lacking any metal–ligand multiple bonds was reported, though this complex is on the cusp of stability; it can be isolated but readily undergoes proton-coupled electron-transfer (PCET), converting to the neptunium­(IV) complex [Np^IV^{NP­(Bu^t^)­[N­(C_2_H_4_)_2_]_2_}_3_{N­(H)­P­(Bu^t^)­[N­(C_2_H_4_)_2_]_2_}]­[B­(C_6_F_5_)_4_].^28,29^ The isostructural plutonium­(V) complex [Pu^V^{NP­(Bu^t^)­[N­(C_2_H_4_)_2_]_2_}_4_]­[B­(C_6_F_5_)_4_] has also recently been reported, and it also engages in PCET to form [Pu^IV^{NP­(Bu^t^)­[N­(C_2_H_4_)_2_]_2_}_3_{N­(H)­P­(Bu^t^)­[N­(C_2_H_4_)_2_]_2_}]­[B­(C_6_F_5_)_4_],^30^ and more readily than the neptunium analog in-line with the aforementioned effective nuclear charge trend. Together, these pentavalent complexes that readily convert to tetravalent derivatives underscore the inherently unstable nature of nondioxo pentavalent transuranium elements, which have otherwise often required hexafluoride ligand sets to be stabilized.^15,17^

Having reported the neptunium­(V)-mono­(oxo) complex **2**, our attention turned to preparing and isolating an analogous neptunium-mono­(imido) complex, because despite thorium- and uranium-imido chemistries taking great strides forward over the past 25 years,^9^ the recent renaissance of transuranium chemistry has not yet resulted in the realization of a transuranium-mono­(imido) complex in any scenario.^11^ We continued using the Tren^TIPS^ ligand because of its success as a ‘privileged ligand’ for thorium, uranium, and, thus far, neptunium.^6–8,26,27,31–42^ Furthermore, although a hypothetical neptunium­(V)–imido complex of the form [Np^V^(Tren^TIPS^)­(NR)] (**3NpNR**) would, like **2**, be 5f^2^ rather than the 5f^1^ formulations of the analogous uranium­(V)-oxo and -imido complexes, potentially introducing destabilizing antibonding interactions to a neptunium­(V)–imido linkage, 5f^2^ uranium­(IV)-imido complexes are well-known, and stable.^9^ Also, Tren^TIPS^ is known to support actinide metals over a wide range (+3 to +6) of oxidation states.^27^

Here, we present evidence for the formation of transient neptunium­(V)–mono­(imido) complexes. We find that two-electron oxidation of a neptunium­(III) precursor by organoazides readily occurs, but the resulting imidos rapidly activate C–H bonds to produce amido derivatives as the only isolable reaction products. The amido derivatives exhibit slow relaxation of their magnetization, which is exceedingly rare in transuranium chemistry. The computed reaction profiles independently reproduce the experimentally observed outcomes, suggesting novel C–H activation and hydrogen atom transfer (HAT) that involves a sequence of H^•^ radical abstraction, electron transfer, then another H^•^ radical abstraction step. This overall three-step HAT sequence is highly unusual in PCET chemistry, and sheds light on transuranium HAT chemistry. The preparation of an isostructural uranium­(IV)-imido that is 5f^2^ but stable, like 5f^1^ uranium­(V)–imido analogs, emphasizes that the highly reactive nature of the putative 5f^2^ neptunium­(V)–imido complex is not a result of the 5f^n^-count but the underlying effective nuclear charge of transuranium actinide elements. Thus, this work highlights stark differences in the stabilities of uranium- and neptunium–imido complexes, and also decisive differences between the ability of mono­(oxo) and mono­(imido) moieties to stabilize (days vs minutes, respectively) high oxidation state transuranium ions.

## Results and Discussion

### Synthesis and Solid-State Structures

Given the established reducing nature of [Np^III^(Tren^TIPS^)] (**1**),^26^ we examined its reactivity with organoazides, Scheme 1a. During routine characterization of batches of **1** to check identity and purity we obtained better quality crystallographic data on **1** than reported previously and details can be found in the Supporting Information. In separate reactions, treatment of **1** in toluene with N_3_SiMe_3_ or in hexane with N_3_Ad (Ad = 1-adamantyl) at low temperature (−74 to −77 °C) afforded rapid effervescence of N_2_, and the dark red solutions turned purple, consistent with the formation of **3NpNR** (R = SiMe_3_ or Ad). However, the purple solutions fade rapidly in minutes, even at low temperature and/or with the exclusion of light, affording dark orange solutions suggesting that one or more follow-up reactions had occurred.

**Scheme 1:**
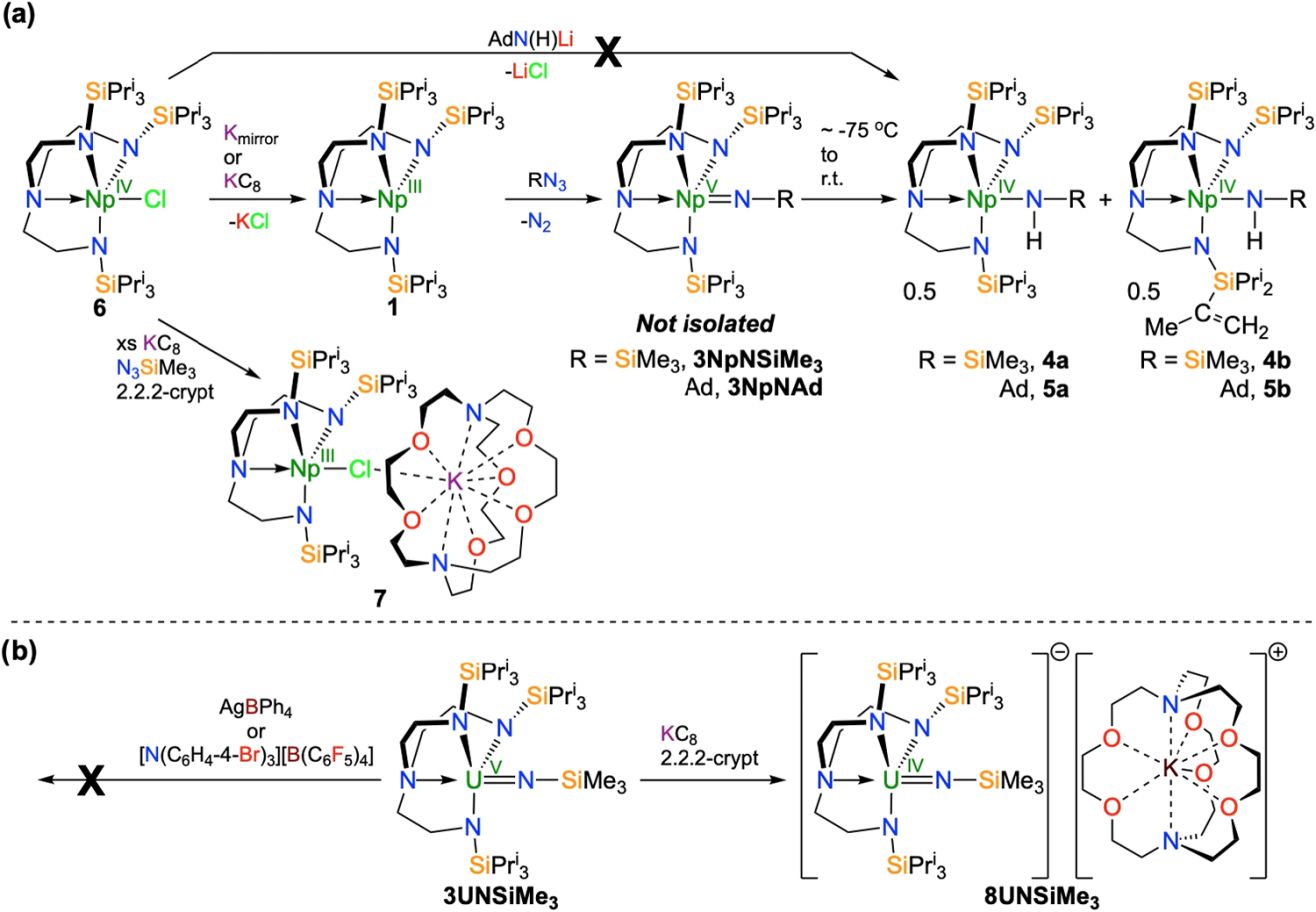
Synthesis of **3NpNR** (R = SiMe_3_, Ad), **4a**, **4b**, **5a**, **5b**, **7**, and **8UNSiMe_3_** ^a^

a Reactions conducted in benzene, toluene, D_6_-benzene, D_8_-toluene, and D_14_-hexane all produced complexes containing N–H linkages with no evidence for D-incorporation, i.e., N–D formation.

For the N_3_SiMe_3_ reaction, orange crystals, isolated in 54% yield, were subsequently identified by single crystal X-ray diffraction to be a 1:1 cocrystal mixture of [Np^IV^(Tren^TIPS^){N(H)SiMe_3_}] (**4a**) and [Np^IV^(Tren^TIPS‑2H^)­{N­(H)­SiMe_3_}] (**4b**, Tren^TIPS‑2H^ = {N­(CH_2_CH_2_NSiPr^i^_3_)_2_​(NCH_2_CH_2_NSiPr^i^_2_C­[Me]=CH_2_)}^3–^), Figure 1a,1b; for **4a** and **4b** each anticipated imido is in fact an amido group, and in **4b** one Pr^i^ unit has been converted into a vinyl group, where the two abstracted H-atoms in principle account for the amido H-atoms, hence preserving the mass-balance of the reaction. The Np^IV^–N­(H)­SiMe_3_ Np^IV^–N distances in **4a** and **4b** are 2.221(2) and 2.203(2) Å, respectively, and are as expected for Np^IV^–N_amido_ distances since they are similar to the **4a**/**4b** Tren Np^IV^–N_amido_ distances (2.260(2)–2.274(2) Å, av. 2.266 Å) and the Np^IV^–N_amido_ distances in [Np^IV^(Tren^TIPS^)­(Cl)] (av. 2.223 Å)^31^ whereas a Np^V^–N_imido_ would be anticipated to have a bond distance <2 Å.^43^ The Np^IV^–N_amine_ distances in **4a** and **4b** of 2.638(2) and 2.635(2) Å are similar to the Np^IV^–N_amine_ distance of 2.605(2) Å in [Np^IV^(Tren^TIPS^)­(Cl)],^31^ consistent with the Np^IV^ formulations of **4a** and **4b** required for charge balance with amido ligands. The Np–N–Si angles in **4a** and **4b** are 166.48(16) and 168.81(19)°, with the slight bending reflecting the presence of the amido H-atoms that were located in the Fourier transform difference map.

**Figure 1:**
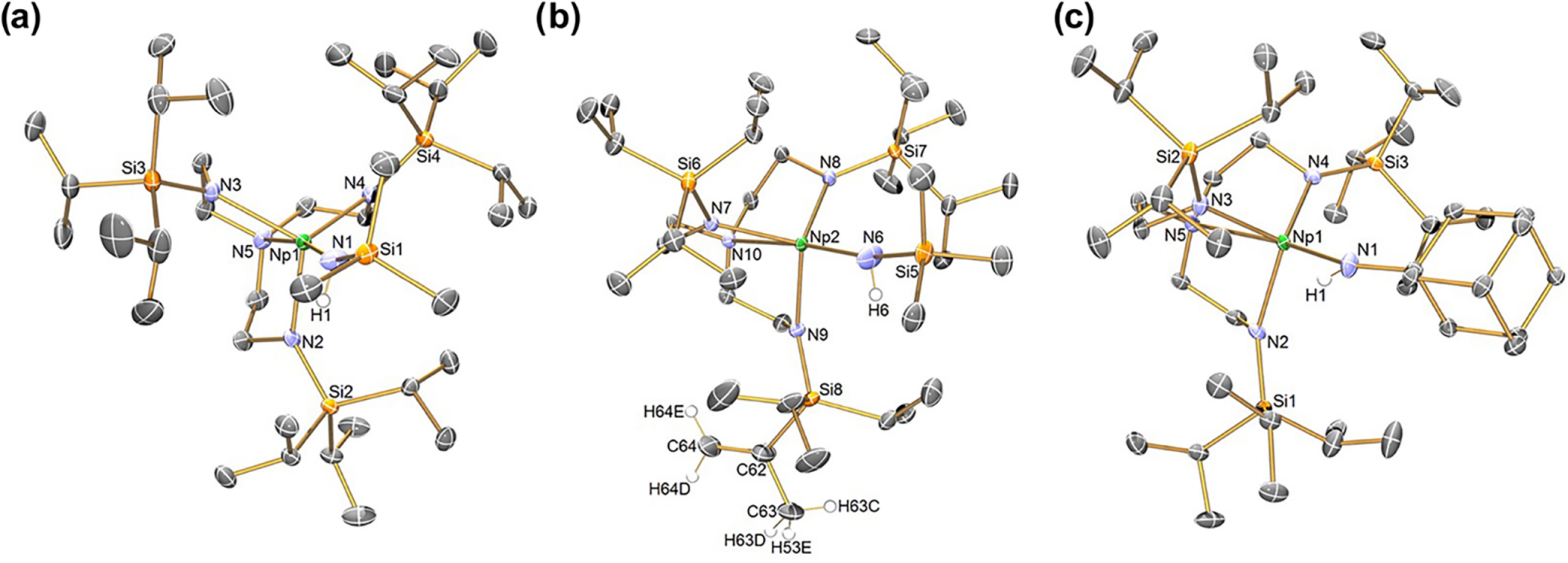
Solid-state molecular structures of **4a** (a), **4b** (b), and **5a** (c) at 150 K with selective labeling and displacement ellipsoids set to 30%. C-bound H-atoms are omitted for clarity, with the exception of the MeC=CH_2_ group in **4b**. Disorder components and other molecules in the crystallographic asymmetric unit are, where present, not shown for clarity.

Reactions of **1** with N_3_SiMe_3_ consistently produce 1:1 mixtures of **4a** and **4b**. However, on one occasion, use of D_6_-benzene instead of toluene as the reaction solvent afforded **4a** in pure crystalline form in <1% yield (Figure S5). In pure **4a** the Np^IV^–N­(H)­SiMe_3_ distance is 2.202(3) Å, and the Np^IV^–N_amido_ (2.256(3)–2.272(3)­Å, av. 2.265 Å) and Np^IV^–N_amine_ (2.628(3) Å) distances are very similar to the **4a**/**4b** cocrystal data. Although **4a** could be isolated, the practicalities of executing additional handling steps experimentally in a transuranium setting meant that the analysis that follows was done on cocrystals of **4a**/**4b**; this was deemed to be reasonable since their cores would be expected to be largely equivalent given the peripheral nature of the Tren modification, and the characterization data overall support that approach.

Where the reaction of **1** with N_3_Ad is concerned, orange crystals formulated as [Np^IV^(Tren^TIPS^)­{N­(H)­Ad}] (**5a**) were isolated in 9% yield. The crystal structure of **5a**, Figure 1c, contains six molecules in the asymmetric unit. This implies that the crystalline product is likely to be a 1:1 cocrystal of **5a/5b** analogously to **4a**/**4b**, but this could not be definitively ascertained through structural refinement. However, the presence of amido rather than imido groups was confirmed by av. Np^IV^–N­(H)­Ad distance and angle metrics of 2.167(8) Å and 157.9(12)°, respectively. In general, manipulations involving **5a** were more complicated than those of **4a** or **4a**/**4b** due to lower solubility and so, given the radiological nature of the compounds, efforts focused on **4a**/**4b**. However, the structural characterization of **5a**/**5b** serves the purpose of establishing that the imido-to-amido transformation is general for the {Np^V/IV^(Tren^TIPS^)} fragment.

### Spectroscopic and Magnetic Characterization

The ^1^H NMR spectrum of **4a**/**4b** contains multiple resonances (Figures S9–S12), but despite the paramagnetism these could be confidently assigned using a combination of 1D and 2D NMR methods (Figures S14–S17). The spectra are consistent with the proposed 1:1 mix of **4a**/**4b** and desymmetrization of the Tren^TIPS^ ligand in Tren^TIPS‑2H^, and clearly confirm the formation of the vinyl =CH_2_ unit from dehydrogenation of one Pr^i^ group. Further details of the NMR assignment of **4a**/**4b** can be found in the Supporting Information.

The IR spectrum of **4a**/**4b** (Figure S22) exhibits a broad, weak absorption at 3220 cm^–1^, which is consistent with the presence of N–H bonds, and compares well to the computed N–H stretches of **4a** and **4b** from analytical frequency calculations at 3261 and 3274 cm^–1^, respectively. The weak nature of the N–H stretch is typical of heavy-atom structures where coupling of vibrational modes greatly reduces observable absorption intensities.^44,45^ In addition, an absorption at 824 cm^–1^, which is not present in **1** nor [Np^IV^(Tren^TIPS^)­(Cl)],^31^ is assigned as a N–H bend; analytical frequency calculations predict this to be at 851 and 861 cm^–1^ for **4a** and **4b**, respectively.

The UV/vis/NIR data confirm the Np^IV^ formulations of **4a** and **4b** (Figure S24). Specifically, in the range 6000–20,000 cm^–1^ (500–1650 nm) the characteristic intraconfigurational bands for a 5f^3^ Np^IV^-ion are observed.^26,31^ The spectrum of **4a**/**4b** is very similar to that of [Np^IV^(Tren^TIPS^)­(Cl)],^26,31^ though we note that while the absorptions have the same overall pattern the molar absorptivity of individual peaks varies. Above 18,000 cm^–1^ a broad feature centered at ∼25,000 cm^–1^ with an extinction coefficient of ∼1250 M^–1^ cm^–1^ emerges, and this is assigned as being f-d in nature with also the onset of charge transfer bands.

The variable-temperature DC SQUID magnetometry data for **4a**/**4b** are also consistent with the Np^IV^ formulations, Figures 2, S27, S29, and S31–S36. The effective magnetic moment (μ_eff_) of **4a**/**4b** is 3.36 μ_B_ at 300 K, slightly lower than the theoretically predicted value for a 5f^3^ (^4^I_9/2_) ion (3.62 μ_B_). This is a common feature for actinide ions,^46^ attributed to the comparable strength of crystal field, spin–orbit coupling, and interelectronic interactions. The effective magnetic moment then exhibits a gradual decline down to 5 K (3.22 μ_B_), reaching 3.18 μ_B_ at 1.8 K. The magnetization (*M*) vs field (*H*) data, at 2 K, are almost saturated by 7 T, with a value of ∼1.8 μ_B_, consistent with the Kramers 5f^3^ nature of **4a**/**4b**.^26,47–49^ The magnetic data were fitted using the full Hamiltonian approach of CONDON,^50^ assuming a simplified trigonal crystal field. The value of spin–orbit coupling (ζ = 2129 cm^–1^), and of the Slater–Condon parameters (*F* ^2^ = 45,212, *F* ^4^ = 38,018, and *F* ^6^ = 28,345 cm^–1^) were fixed to literature values,^51^ as they are typically little affected by the crystal field potential (cf. in 1 M perchloric acid, ζ = 2129 cm^–1^).^52^ The orbital reduction factor, although fitted, produced a best value κ = 1, indicating a fully unquenched orbital angular momentum. The following crystal field parameters (Wybourne notation) were obtained: *B*_2_^0^ = 8398, *B*_4_^0^ = 6726, *B*_6_^0^ = −6488, and *B*_4_^3^ = 2246 cm^–1^. The resulting fit suggests that combining a triamidoamine and primary amide ligand at Np produces a pseudo quartet *m*_*J*_ ground state, composed of two doublets of *|*±9/2⟩ and *|*±7/2⟩ character that are separated by only 0.25 cm^–1^, indicating a certain degree of axial character,^53^ consistent with the strongly magnetic nature of **4a**/**4b** as suggested by the *M* vs *H* data.

**Figure 2:**
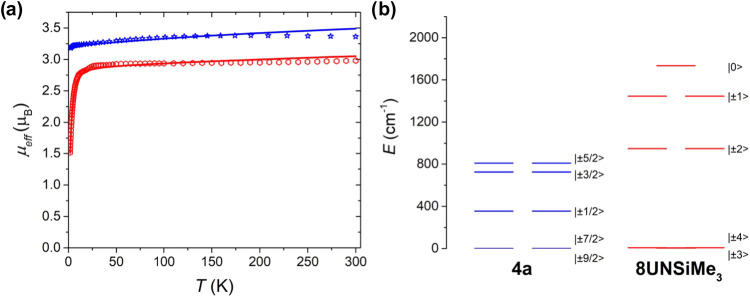
Experimental and calculated magnetic data and energy level diagrams for **4a** and **8UNSiMe**_**3**_. (a) Variable-temperature SQUID magnetometry μ_eff_ (μ_B_) vs T (K) for powdered **4a**/**4b** (blue stars) and **8UNSiMe**_**3**_ (red circles) in an external field of 0.5 T. The blue and red lines represent the full Hamiltonian modeling. (b) Energy level diagrams calculated from the full Hamiltonian modeling of the ^4^I_9/2_ (*5*f^*3*^) and ^3^H_4_ (*5*f*^2^*) states of **4a** and **8UNSiMe**_**3**_, respectively. The *|*±*m*_*J*_> notation refers to the principal *m*_*J*_ component of each level.

Investigation of the AC susceptibility data of **4a**/**4b** at 2 K and 4600 Oe reveals two distinct relaxation processes in the χ″(ν) curve, one below 10 Hz and the other around 400 Hz (Figures S30 and S31). As the temperature is increased, the low-frequency process rapidly disappears, while the high-frequency process shifts to higher frequencies until it is outside the experimental range. Accordingly, the temperature-dependent behavior of the high-frequency process was investigated, employing a single-component Debye model to fit the observed curves. In order to exclude the low-frequency process and avoid any potential errors introduced by its inclusion, the fit was performed within the frequency range of 100 to 10,000 Hz. The extracted values of α are consistent with relaxation dynamics dominated by a quantum tunnelling process at very low temperature, which is replaced by a thermally activated process as the temperature is increased. Consequently, the temperature dependence of the extracted values of relaxation times τ has been fitted with a phenomenological model including a QTM and a Raman process, while the inclusion of a *U*_eff_ barrier is not realistic, since the respective states are composed of two doublets of *|*±9/2⟩ and *|*±7/2⟩ character. Consequently, **4a**/**4b** add to the small number of transuranium complexes that have been shown to exhibit slow relaxation of their magnetization.^54–59^

Since the Np^IV^ centers in **4a** and **4b** are 5f^3^ Kramers ions we collected EPR data on the 1:1 mixture of **4a**/**4b**, and for comparison EPR data on Np^IV^ 5f^3^ [Np^IV^(Tren^TIPS^)­(Cl)] (**6**),^31^ Figure 3. Analysis of these spectra is complicated by the strong hyperfine coupling to the ^237^Np nucleus (*I* = 5/2) and the relatively low fields available for these radiological experiments. The analysis is challenging at these fields because the whole spectrum is not observed and hyperfine coupling is of similar strength to the *g*-anisotropy, which precluded accurate and meaningful spectral simulation. However, the observation of EPR spectra is consistent with the Kramers 5f^3^ Np^IV^ assignments of **4a** and **4b**,^60^ and the recorded spectra are consistent with the large hyperfine coupling expected for Np^IV^.

**Figure 3:**
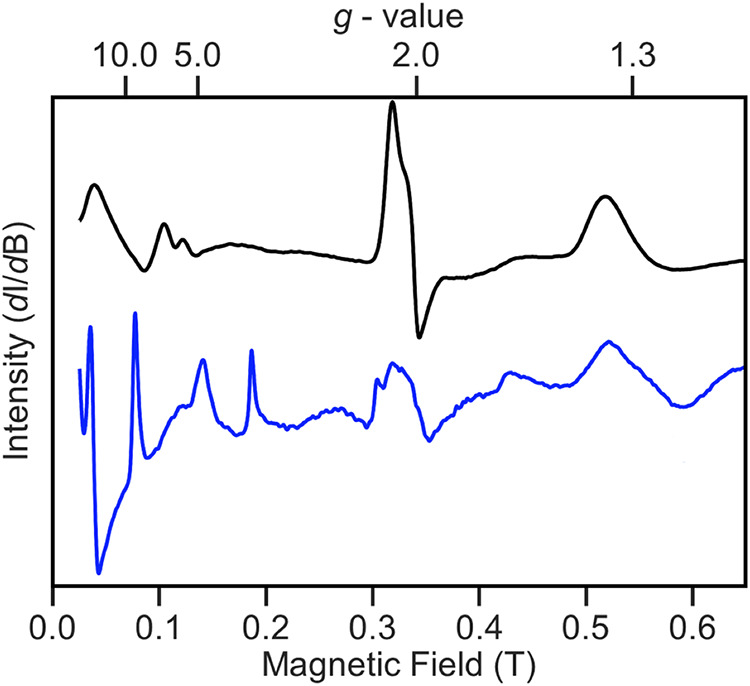
X-band (9.53 GHz) EPR spectra of frozen toluene solutions of **6** (black line) and **4a**/**4b** (blue line) at 6 K.

### Synthetic Considerations and Neptunium–Uranium Comparison

In order to probe the reactions that produce **4a**/**4b** and **5a**, we examined synthetic variations. Interestingly, treating **6** with LiN­(H)­Ad does not afford **5a**, Scheme 1a. We attribute this lack of reaction to steric factors, but of more importance this suggests that **5a**, and by inference **4a**/**4b**, derives from a transient Np^V^–imido complex. We surmised that reduction of neptunium from Np^V^ to Np^IV^ may be driving the reaction. Hence, in an attempt to trap a more stable Np^IV^-imido we treated **6** with excess KC_8_, N_3_SiMe_3_, and 2.2.2-cryptand at −78 °C, Scheme 1a. However, rather than forming any imido or amido complexes with Np^IV^, we instead isolated the trivalent complex [Np^III^(Tren^TIPS^)­(μ-Cl)­K­(2.2.2-cryptand)] (**7**), details of which can be found in the Supporting Information and Figures S7, S18–S20, and S25. Reactions with **1** and KC_8_, N_3_SiMe_3_ and 2.2.2-cryptand at −78 °C to avoid the KCl inclusion complex did not yield any isolable compounds. The N–D isotopologues of **4a**/**4b** were not prepared since that would require wholesale deuteration of the Tren^TIPS^ Pr^i^-substituents. However, to test whether the amido H-atoms derive from the conversion of one Pr^i^-group to a vinyl moiety, we separately repeated the reaction of **1** with N_3_SiMe_3_ in D_6_-benzene, D_8_-toluene, and D_14_-*n*-hexane. However, we do not find any evidence for D-incorporation by ^2^H NMR spectroscopy (Figure S13), suggesting that the Pr^i^ to vinyl group conversion, and not the reaction solvent, is indeed the source of the amido H-atoms.

The rapid decay of the transient Np^V^-imido to Np^IV^-amido formulation contrasts to uranium imido chemistry where the imido linkage has now been found with uranium over a wide range of oxidation states and ancillary ligand classes.^9^ Early work on [Cp_3_U^V^NPh] disclosed conversion to [Cp_3_U^IV^N­(H)­Ph],^61^ but we have previously found Tren^TIPS^–U^V^–imido complexes such as [U^V^(Tren^TIPS^)­(NSiMe_3_)] (**3UNSiMe**_**3**_) and [U^V^(Tren^TIPS^)­(NAd)] (**3UNAd**) to be indefinitely stable in the absence of air and moisture.^62^ To probe whether the unstable nature of the transient Np^V^-imido is due to the number of 5f-electrons, since structurally analogous **3UNSiMe**_**3**_ is a 5f^1^ U^V^ complex and putative **3NpNSiMe**_**3**_ is 5f^2^ Np^V^, we reduced **3UNSiMe**_**3**_ with KC_8_ in the presence of 2.2.2-cryptand to afford the 5f^2^ U^IV^–imido complex [K­(2.2.2-cryptand)]­[U^IV^(Tren^TIPS^)­(NSiMe_3_)] (**8UNSiMe**_**3**_) in 72% isolated crystalline yield, Scheme 1b. By contrast, attempts to oxidize **3UNSiMe**_**3**_ with AgBPh_4_ or [N­(C_6_H_4_-4-Br)_3_]­[B­(C_6_F_5_)_4_] returned only **3UNSiMe**_**3**_, Scheme 1b, suggesting that the putative uranium­(VI)-imido complexes [U^VI^(Tren^TIPS^)­(NSiMe_3_)]­[BAr_4_] (Ar = Ph or C_6_F_5_) are, like **3NpNR**, also unstable.

The solid-state structure of **8UNSiMe**_**3**_, Figure 4, exhibits a U^IV^–N_imido_ distance of 2.032(2) Å, which is longer than the U^V^–N_imido_ distance of 1.954(3) Å in **3UNSiMe**_**3**_,^62^ but significantly shorter than the Np^IV^–N_amido_ distances in **4a**/**4b**/**5a**. The UV/vis/NIR spectrum of **8UNSiMe**_**3**_ (Figure S26) exhibits weak intraconfigurational f–f absorptions that are characteristic of a ^3^H_4_ ion.^6^ Variable-temperature DC SQUID magnetometry data for **8UNSiMe**_**3**_, Figures 2 and S28, return an effective magnetic moment of 2.98 μ_B_ at 300 K, which is smaller than the expected value for a 5f^2^ (^3^H_4_) ion (3.58 μ_B_). The theory-experiment discrepancy is more pronounced for **8UNSiMe**_**3**_ than **4a**/**4b**, suggesting a lower (<1) orbital reduction factor κ for the former compared to the latter. The effective magnetic moment of **8UNSiMe**_**3**_ remains almost constant down to 12 K (2.77 μ_B_), at which point it rapidly drops to 1.51 μ_B_ by 1.8 K. A constant effective magnetic moment over most of the temperature range with an abrupt, rapid decline at low temperature is consistent with other examples of U^IV^ complexes with strong donor ligands, e.g., oxos, imidos, and phosphino-silyl-alkylidenes, that enforce effective axial symmetry over the spin−orbit ground multiplet producing a paramagnetic ground state at low temperatures.^39,40,53,63–79^ Certainly, the *M* vs *H* data for **8UNSiMe**_**3**_ are not the usual unsaturated linear plot with small *M* value that signifies a singlet ground state, and instead are approaching saturation at the highest available *H* (7 T) with a value of 1.1 μ_B_ indicative of a ground Kramers doublet. The low-temperature *M* vs *H* curves are almost identical at 2 and 4 K, indicating that the excited states must be significantly separated in energy from the ground state. The magnetic data were fitted using the Hamiltonian approach of CONDON, producing the following crystal field parameters: *B*_2_^0^ = 4664, *B*_4_^0^ = −2932, *B*_6_^0^ = 3378, and B_4_^3^ = 536 cm^–1^, and a spin–orbit coupling value of ζ = 1740 cm^–1^. The latter value reflects the κ value of 0.93, (cf. 1.0 for **4a**/**4b**) and the increased overall energy level splitting, Figure 2b, suggest that the apical imido in **8UNSiMe**_**3**_ exerts a stronger crystal field than then amido in **4a**/**4b**. The fits reveal that **8UNSiMe**_**3**_ has two singlets of *m*_*J*_ = |±3> components and one doublet of *m*_*J*_ = |±4> components; however, given the total energy separation of these four *m*_*J*_ components is <9 cm^–1^ then they can be considered to effectively be a pseudoquartet ground state like **4a**/**4b**. As expected, the total splitting of the *m*_*J*_ states for **8UNSiMe**_**3**_ (∼1800 cm^–1^) is more than twice that of **4a** (∼820 cm^–1^), consistent with a stronger ligand field at U^IV^ than Np^IV^ on grounds of metal oxidation state and also coordinated ligands (imido vs amido, respectively).

**Figure 4:**
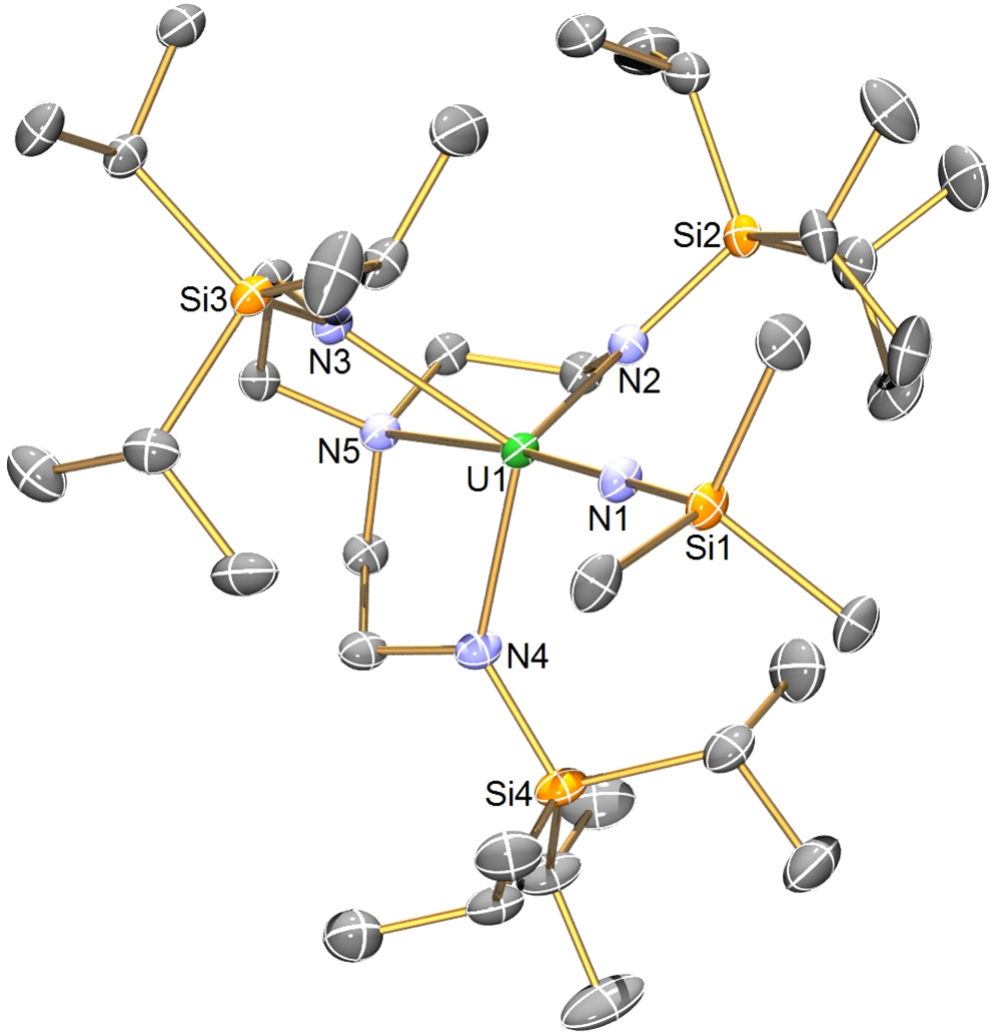
Molecular structure of the anion component of **8UNSiMe**_**3**_ at 100 K with selective labeling and displacement ellipsoids at 40%. Hydrogen atoms, disorder, and the [K­(2.2.2-crypt]^+^ cation component are omitted for clarity.

With the formulation of **8UNSiMe**_**3**_ confirmed, we tested its stability, noting that its straightforward isolation contrasts to the analogous reaction with neptunium that produces cocrystallized **4a**/**4b**. We refluxed a THF solution of **8UNSiMe**_**3**_ at 80 °C for 2 weeks and by ^1^H NMR spectroscopy found no decomposition at all. The robust nature of **8UNSiMe**_**3**_ stands in contrast to the instability of the putative Np^V^-imidos **3NpNR**, at even −78 °C, and we note that the proton source in the PCET conversion of [Np^V^{NP­(Bu^t^)­[N­(C_2_H_4_)_2_]_2_}_4_]­[B­(C_6_F_5_)_4_] to [Np^IV^{NP­(Bu^t^)­[N­(C_2_H_4_)_2_]_2_}_3_{N­(H)­P­(Bu^t^)­[N­(C_2_H_4_)_2_]_2_}]­[B­(C_6_F_5_)_4_] is THF.^29^ Although the formal charge states of **3NpNSiMe**_**3**_ and the U-component of **8UNSiMe**_**3**_ are different, the actinide components are isostructural and of the same 5f^n^ count and yet their stabilities are very different. We therefore conclude that the 5f^n^ count is not central to the observed stability differences.

### Quantum Chemical Calculations and Mechanistic Analysis

To provide further insight into the divergent stabilities of **3NpNSiMe**_**3**_, **3NpNAd**, **3UNSiMe**_**3**_, and **8UNSiMe**_**3**_ we turned to DFT and *ab initio* calculations and computed **3NpNSiMe**_**3**_, **3NpNAd**, and **8UNSiMe**_**3**_, noting that DFT characterization of **3UNSiMe**_**3**_ has been reported previously (See Supporting Information and Figures S38–S51 and Tables S4–S10). Geometry and SCF optimizations proceeded to convergence in each case with good agreement of experimental and computed bond metrics. The computed properties in terms of bond orders, charges, spin densities, NBO breakdowns, and QTAIM analysis are all unexceptional and compare well to previously published data on **3UNSiMe**_**3**_ and **3UNAd** ^62^ indicating that the bonding of the U- and Np-imido linkages are similar. The straightforward nature of the calculations suggested that unusual multireference character should not be responsible for the imido-to-amido conversion that yields **4a**/**4b**, but to confirm this aspect we conducted SO-CASSCF calculations on **3NpNSiMe**_**3**_. The full model of **3NpNSiMe**_**3**_ utilized a (2,7) active space, which has been successfully applied in previous SO-CASSCF calculations of actinide complexes.^48,80–82^ The resulting ^3^H state yielded a main configuration of 64% 5f_σ_ and 36% 5f_δ_, with the corresponding singlet state being 36 kcal/mol higher at this level of theory. To further confirm these results, we performed (2,7) and (12,16) active space calculations on truncated [Np^V^{N­(CH_2_CH_2_NSiH_3_)_3_}­(NSiH_3_)], and found the main configurations to be 82:18 and 76:17% 5f_σ_/5f_δ_, respectively. In these active spaces the populations of the formally doubly occupied orbitals proved to be very high (≥1.97 e) whereas those of the formally vacant orbitals were very low (≤0.03 e), confirming the relevance of the minimal active spaces. Additional calculations on the truncated structures included CASPT2 accounting for dynamic electron correlation and determination of the SO ground state.^83,84^ The latter procedure was performed using the CASSI method,^84^ which allows CASSCF wave functions for different electronic states to interact under the influence of a spin–orbit Hamiltonian. In the state-averaged calculations all the states of the high-spin and low-spin Np were considered, i.e., for [Np^V^{N­(CH_2_CH_2_NSiH_3_)_3_}­(NSiH_3_)] 21 and 28 roots. To provide further comparison and comparative validation, we undertook DFT calculations on **4a**, **4b**, and **5a** and *ab initio* calculations on **4a**. As above, all the computed DFT parameters are unexceptional, though as expected replacement of an imido with amido results in less effective axial symmetry and so the ^4^I configuration is rather mixed. Importantly, overall we find no evidence for open shell radicaloid character in the imido linkages of **3NpNSiMe**_**3**_ and **3NpNAd** beyond their 5f^2^ formulations, suggesting that the reactivity that produces **4a**/**4b**, and its contrast to **8UNSiMe**_**3**_, likely results from the effective nuclear charge of neptunium, and that it is greatly increased when compared to uranium.

To further probe the conversion of **1** to **4a**/**4b**, we computed potential reaction profiles (Tables S11–S63). Complex **1** reacts with N_3_SiMe_3_, Figure 5, via an adduct transition state (**TS 1-I**) with a low barrier of only 4.5 kcal/mol, to produce an intermediate (**I**) whose spin density at Np (3.17, *c.f*. 4.15 in **1**) suggests the presence of 5f^3^ Np^IV^. The ready oxidation of **1** is in-line with prior electrochemical characterization of **1** that suggest it is strongly reducing,^26^ and the one-electron oxidation of Np corresponds to a pseudo-oxidative addition reaction where the N_α_–N_β_ bond is already activated in the strained three-membered NpN_2_ ring. The Np–N_β_ and N_α_–N_β_ bonds are readily broken via a transition state (**TS I-3**) with a barrier of only 3.0 kcal/mol, resulting in extrusion of N_2_ and formation of **3NpNSiMe**_**3**_, where the 5f^2^ Np­(V)–imido formulation is corroborated by a Np spin density of 2.59. Complex **3NpNSiMe**_**3**_ lies 46.2 kcal/mol below the starting point of **1** when the reaction is conducted in toluene. Thus, the formation of **3NpNSiMe**_**3**_ is kinetically and thermodynamically favored, and this is consistent with the experimental observation of rapid N_2_ evolution when **1** is treated with N_3_SiMe_3_. The energetics of formation of **3NpNSiMe**_**3**_ are little affected by the solvent, Figure 5, where we find that reactions in benzene, toluene, or *n*-hexane are all within 2.4 kcal/mol of one another, which is only ∼5% of the total energy stabilization when **3NpNSiMe**_**3**_ is formed in aromatic solvents.

**Figure 5:**
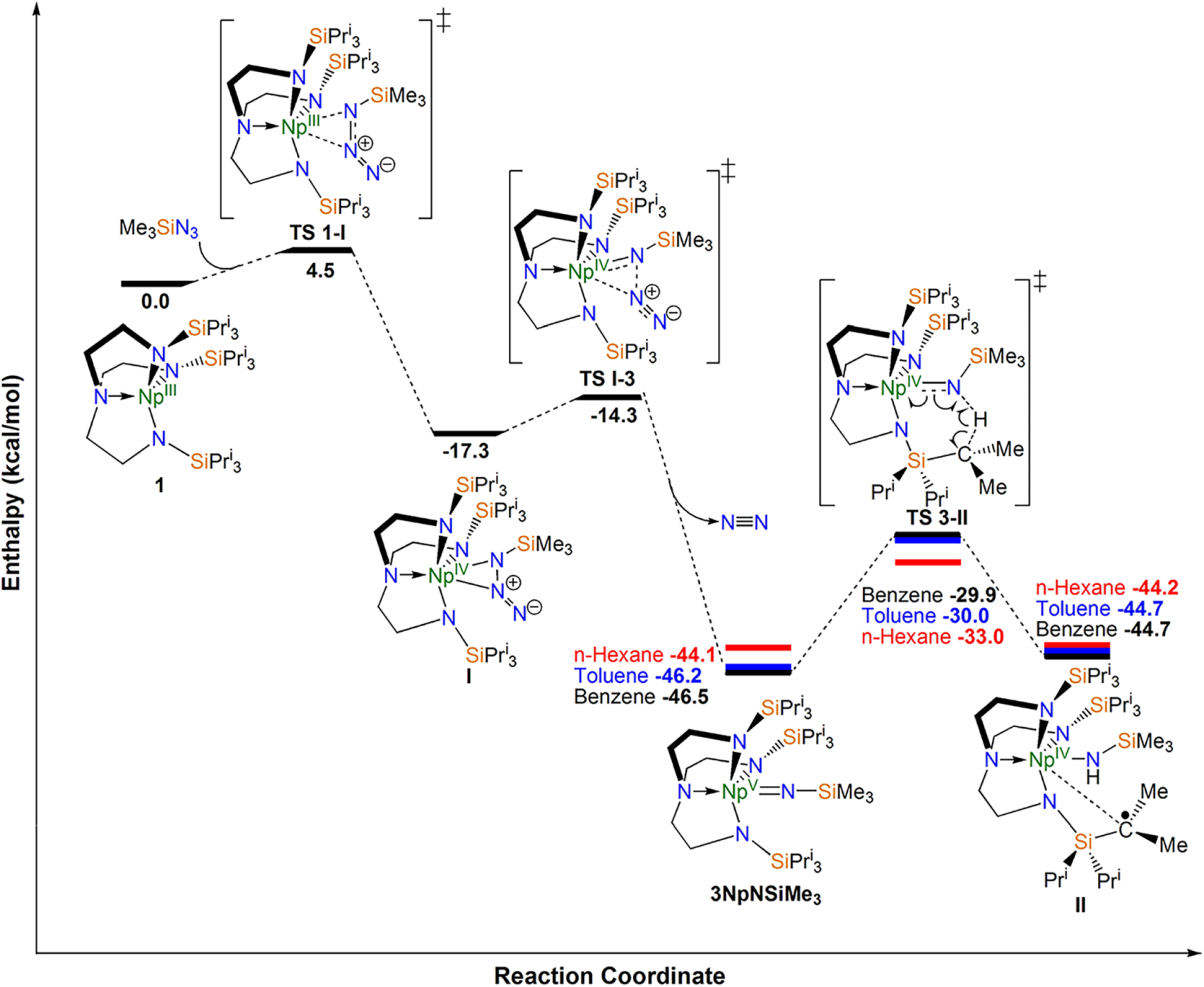
Computed reaction profile for the reaction of **1** with N_3_SiMe_3_ to give **3NpNSiMe**_**3**_.

We next considered how **3NpNSiMe**_**3**_ converts to **4a**/**4b**. We first examined direct reactions of benzene, toluene, or hexane solvent with **3NpNSiMe**_**3**_, Figure S52, and find barriers of ∼36 kcal/mol to convert **3NpNSiMe**_**3**_ to **4a** in reactions that are overall highly exogonic (∼ −70 kcal/mol). While this suggests that these reactions are potentially accessible at room temperature, hinting at wider potential for transuranium-imido C–H activation chemistry, they would be rather slow and hence would essentially not occur at all at low temperatures. The calculation is consistent with the fact that experimentally we do not find any evidence for N–D formation when the reactions are run in D-solvents. That scenario is also not consistent with the experimental observations of **3NpNSiMe**_**3**_ quickly converting to **4a** and **4b** even at low temperature, since this mechanism does not account for the formation of **4b** as well as **4a**. However, on the intrinsic reaction coordinate we identified that **3NpNSiMe**_**3**_ can convert, via a transition state with a barrier of 16.2 kcal/mol for the reaction in toluene, to the Np^IV^-amido-isopropyl-radical complex [Np^IV^{N­(CH_2_CH_2_NSiPr^i^_3_)_2_(CH_2_CH_2_NSiPr^i^_2_C^•^Me_2_)}­{N­(H)­SiMe_3_}] (**II**) that is only 1.5 kcal/mol higher in energy than **3NpNSiMe**_**3**_. The spin density on Np is now 3.21, confirming the presence of 5f^3^ Np^IV^ (cf 2.59 for Np^V^ **3NpNSiMe**_**3**_). The reaction is little changed when replacing toluene with benzene solvent, but the reaction becomes slightly more favorable in *n*-hexane, by 0.1 kcal/mol and with a reduced barrier of 11.1 kcal/mol.

From **II**, three potential pathways present themselves: (i) solvent H-atom transfer; (ii) intermolecular H-atom transfer; (iii) intramolecular then intermolecular H-atom transfers (Figure 6). While the reaction that produces **II** is only modestly exogonic in hexane, and slightly endogonic in benzene or toluene, the three reactions are all kinetically competitive and provide a staging post to substantially exogonic final products overall. The reaction with solvent, using benzene as an exemplar, has a barrier of 26.1 kcal/mol, which is experimentally feasible, but like the direct reaction of solvent with **II** produces only **4a** and not **4a**/**4b**. In principle, activation of the Tren backbone NCH_2_CH_2_N linkages, since the methylene C–H bonds are slightly acidic being between two N-atoms, is favorable. This presents an initial experimentally accessible barrier of 18.3 kcal/mol for an intramolecular H-atom transfer to produce **III**, which is 1.2 kcal/mol more stable than **II** and almost athermic compared to **3NpNSiMe**_**3**_. Complex **III** can undergo an intermolecular H-atom transfer reaction with itself, with an accessible barrier of 24.4 kcal/mol, to produce **4a** and **IV**, where one of the Tren NCH_2_CH_2_N arms is now NC­(H)­C­(H)­N. The **4a**/**IV** product mixture is 19.8 kcal/mol more stable than **II** and hence is in principle a feasible reaction pathway, however it is not what is experimentally observed. Instead, with a barrier of 20.2 kcal/mol we find that **II** can react with itself in an intermolecular fashion, where the C-radical of one molecule of **II** abstracts a H-atom from the methyl group of a radical CMe_2_ unit in another molecule. That reaction is thermodynamically highly favorable (31.3 kcal/mol lower in energy than **II** and 76 kcal/mol lower in energy from (**1**)) and kinetically accessible, and this pathway, which leads to the greatest thermodynamic stabilization overall of all three potential reaction pathways, accounts for the experimentally observed formation of **4a** and **4b**.

When considering the analogous reaction profiles for **3UNSiMe**_**3**_ and **8UNSiMe**_**3**_, in all cases we consistently find significantly larger energy barriers at every step (Figures S53–S56). For example, the barriers for direct reactions of **3UNSiMe**_**3**_ and **8UNSiMe**_**3**_ with D-solvents are 58.6 and 64.9 kcal/mol, respectively. Where conversion of **3UNSiMe**_**3**_ and **8UNSiMe**_**3**_ to the corresponding amido-isopropyl-radical species is concerned, these reactions are consistently enthalpically uphill with barriers of up to 94.6 and 113.0 kcal/mol. These calculations thus reproduce the experimentally determined robustness of the U–imido complexes, and the magnitude of the energy barriers suggests that the formal charge state of the U and Np complexes is not a key driver with respect to their divergent reactivities.

**Figure 6:**
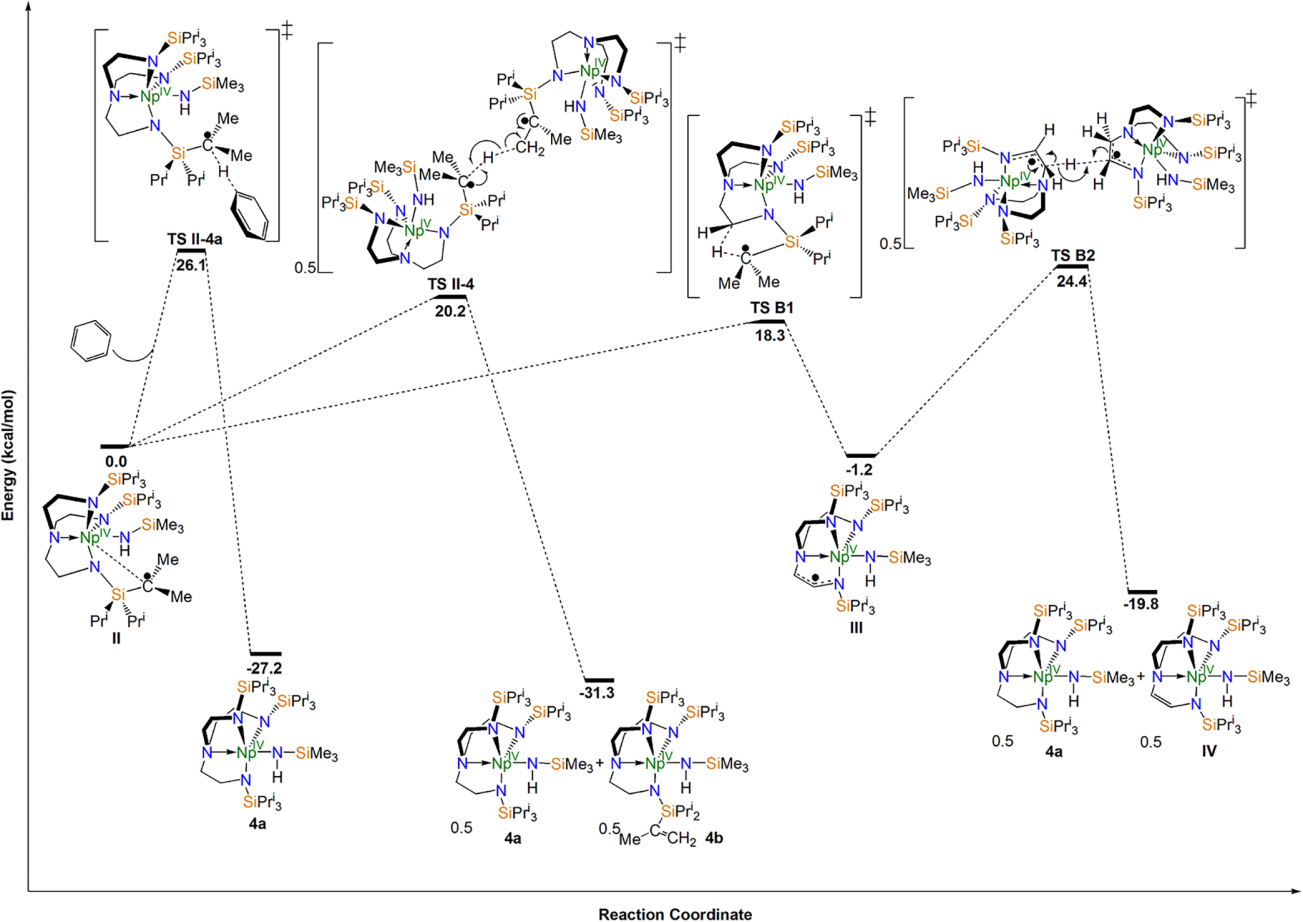
Computed reaction profiles for potential reaction pathways onward from **II**.

The overall mechanism that produces **4a**/**4b**, which is summarized in Figure 7, merits specific discussion since it involves transfer of H^+^ and e^–^ (or H^•^) and hence it falls within the broad and important domain of proton coupled electron transfer (PCET).^85–87^ PCET has become quite a general descriptive term covering a wide range of reactions, but it useful to view such reactions as describing a continuum,^86^ from hydrogen atom transfer (HAT) to multiple-site concerted proton–electron transfer (MS-CPET); HAT is where the H^•^ (or H^+^/e^–^) come from the same donor and ends up in the same acceptor (X–H + Y → X + Y–H), emphasizing that the H^+^/e^–^ destinations are spatially proximate or the same, whereas CPET can involve different reagents and the H^+^/e^–^ can end up in spatially separated locations (X–H + Y + Z → X + Y–H^+^ + Z^–^) not necessarily in the same molecule (multisite). The first reaction step that converts **3NpNSiMe**_**3**_ can thus be generally classified as PCET, but more specifically as a metal-mediated canonical HAT reaction.^84^ This is because the Pr^i^ group produces a H^•^ leaving behind a SOMO “hole” where the C–H bond was, and so there is no ambiguity about which orbital the H^•^ originates from. The imido is formally protonated, and the e^–^ formally transfers to the Np ion, as indicated by the computed spin densities (see earlier), but the e^–^ transfers into the Np=N π* (which has Np and N character) to produce the N–H bond and release an e^–^ onto the Np ion. Hence, the H^+^ and e^–^ both transfer to acceptor sites involving the imido center. The intermolecular reaction between two molecules of **II** can also, by the above definitions, be classified as a HAT reaction. Metal-imidos, e.g., d^0^ imidos and Co derivatives, can react to produce amido or amido-alkyl derivatives, but usually do so by reaction with an external substrate.^88,89^ This is indeed usually the case in actinide chemistry, for example alkane oxidations with uranyl and biochemical reductions of actinyls spanning U to Am,^90–102^ and uranium-nitrides and imides that have been found to engage in photochemically promoted PCET-type reactions.^62,103–108^ Nevertheless, such reactivity usually exhibits a single HAT step, as exemplified by the PCET conversion of [M^V^{NP­(Bu^t^)­[N­(C_2_H_4_)_2_]_2_}_4_]­[B­(C_6_F_5_)_4_] to [M^IV^{NP­(Bu^t^)­[N­(C_2_H_4_)_2_]_2_}_3_{N­(H)­P­(Bu^t^)­[N­(C_2_H_4_)_2_]_2_}]­[B­(C_6_F_5_)_4_] (M = Np, Pu).^28–30^ Thus, the double HAT reaction of **3NpNSiMe**_**3**_ is, as far as we are aware, highly unusual in PCET chemistry generally, and given its multisite and concerted reactivity it may be appropriate to consider it to have some MS-CPET character when described overall.^86^

**Figure 7:**
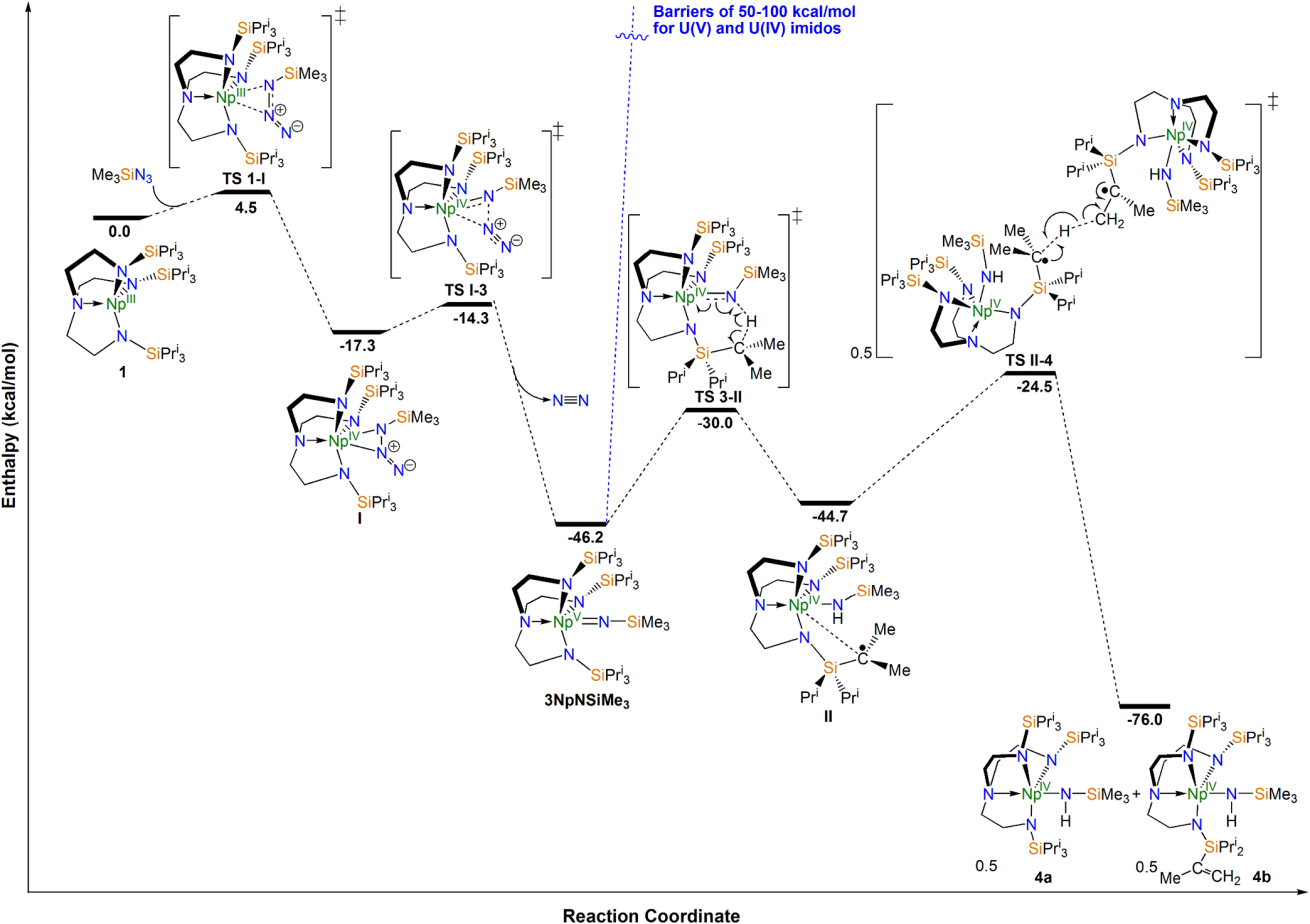
Overall computed reaction pathway accounting for the formation of **4a**/**4b**, via **3NpNSiMe**_**3**_, from the reaction between **1** and N_3_SiMe_3_ and showing how the analogous uranium-imido reactivity is thermodynamically unfeasible.

Radiological considerations of the neptunium chemistry reported here precluded detailed experimental mechanistic studies beyond the D-solvent reactions, but there can be confidence that the computed reaction profiles are representative of the observed reactivities because they successfully and independently reproduce the following key experimental observations: (i) the rapid reaction of **1** with N_3_SiMe_3_ to give a transient imido; (ii) the quick conversion of imido to amides; (iii) that there is not any D-incorporation into the products, i.e., no N–D bonds are formed, nor formation of dehydrogenated **IV**; (iv) formation of the vinyl group in **4b**, hence accounting for the formation of **4a** and **4b** as well as Np^IV^–N­(H)–R linkages; (v) that the U-imidos are robust and do not engage in analogous C–H activation reactions. When considering all the experimental and computational findings, including that the covalency of the Np^V^=NR and U^V^=NR linkages is similar, we conclude that the greater effective nuclear charge at Np compared to U decisively dictates and drives the observed reactivity.

In our previous report of neptunium­(III)-diphosphonioalkylidenes,^24^ we noted an apparent ‘diagonal relationship’ between uranium and neptunium, where—in terms of 5f-/6d-orbital bonding as a function of spatial reach and energy matching—the effects of increased effective nuclear charge of neptunium compared to uranium may be offset by the former adopting an oxidation state one unit lower than the latter, i.e., Np^III^ ∼ U^IV^. Here, we find that while the U^V^=NR linkages in **3UNR** are stable, the Np^V^=NSiMe_3_ linkage in putative **3NpNSiMe**_**3**_ is unstable, suggesting that perhaps a Np^IV^=NR linkage may be more stable. Likewise, the fact that we could not oxidize **3UNSiMe**_**3**_ to give a stable U^VI^=NSiMe_3_ linkage is also consistent with that premise since its diagonal analog Np^V^=NSiMe_3_ (**3NpNSiMe**_**3**_) is evidently unstable. While more experimental data are certainly needed to fully confirm this emerging trend, looking more widely a parallel can be drawn between the evidently reactive nature of Np^V^=NR and the aforementioned reactivity of nitride U^VI^≡N triple bonds.^62,103–105^

## Conclusions

To conclude, we have presented an internally consistent set of experimental and computational observations that evidence transient neptunium­(V)-mono­(imido) complexes. Although a neptunium­(III) precursor is readily oxidized by organoazides, the resulting imido complexes rapidly convert to amido-derivatives which exhibit quartet electronic ground states and slow relaxation of their magnetization that remains rare for transuranium elements. This is accomplished by C–H activation in the ancillary ligand periphery, where two HAT reactions involve H^•^ radical abstraction, electron transfer, then another H^•^ radical abstraction step. This overall three-step PCET sequence is highly unusual, not only in transuranium chemistry, but more widely, and provides complementary data to the few existing reports on PCET reactions involving uranium-nitridos and -imidos, actinyls, and transuranium M-N linkages. The preparation of an isostructural uranium­(IV)–imido that is 5f^2^ but stable like 5f^1^ uranium­(V)-imido analogs suggests that the highly reactive nature of the putative 5f^2^ neptunium­(V)–imido complexes is not a result of the 5f^n^-count but the immutable underlying effective nuclear charge of transuranium elements. Consequently, this work highlights a “diagonal relationship” between uranium and neptunium, and hence contrasting differences in the stabilities of uranium- and neptunium-imido complexes of the same metal oxidation state. It also highlights decisive differences between the ability of oxo and imido ligands to stabilize high oxidation state transuranium ions that echoes the now concluded but previously challenging search for isolable terminal uranium-nitrides.^62,109^ Thus, this work underscores the milestone that preparing an isolable high oxidation state transuranium–mono­(imido) complex will represent.^8,11^

## Supplementary Material



## Data Availability

All other data are provided in the Supporting Information or are available from the authors on reasonable request.

## Notes

Experimental and computational details and X-ray crystallographic, spectroscopic, magnetic, and quantum chemical calculations (PDF)

## ∇

College of Chemistry, Zhengzhou University, Zhengzhou 450001, China
