# Supplementary material for: Transient Triamidoamine Neptunium(V)–Mono(Imido) Complexes: C–H Activations and Hydrogen Atom Transfer Driven by Effective Nuclear Charge
Source: J Am Chem Soc. 2026 May 19;148(21):21505–17. doi: 10.1021/jacs.6c00505 (PMC13244476; doi:10.1021/jacs.6c00505)
Supplement: Supplementary file 1 [file ja6c00505_si_001.pdf]

– *Supporting Information* –

Transient Triamidoamine Neptunium(V)-Mono(imido) Complexes: C-H Activations and Hydrogen Atom Transfer Driven by Effective Nuclear Charge

Michał S. Dutkiewicz,[1,2] Iskander Douair,[3] Jingzhen Du,[1,4] Conrad A. P. Goodwin,[1,5] Leonardo Tacconi,[6] Mauro Perfetti,[6] Andrew J. Gaunt,[5] Samuel M. Greer,[5] Benjamin W. Stein,[5] Roberto Caciuffo,[2] Eric Colineau,[2] Jean-Christophe Griveau,[2] John A. Seed,[1] Attila Kovács,[2] Brian L. Scott,[7] Ashley J. Wooles,[1] Laurent Maron,[3*] Olaf Walter,[2*] and Stephen T. Liddle[1*]

[1] Department of Chemistry and Centre for Radiochemistry Research, The University of Manchester, Oxford Road, Manchester, M13 9PL, UK.

[2] European Commission, Joint Research Centre, Postfach 2340, 76125, Karlsruhe, Germany.

[3] LPCNO, CNRS & INSA, Université Paul Sabatier, 135 Avenue de Rangueil, Toulouse 31077, France.

[4] Present address: College of Chemistry, Zhengzhou University, Zhengzhou, 450001, China.

[5] Chemistry Division, Los Alamos National Laboratory, Los Alamos, New Mexico 87545, USA.

[6] Department of Chemistry Ugo Schiff, University of Florence, Via della Lastruccia 3, 50019, Sesto Fiorentino, Italy.

[7] Materials Physics & Applications Division, Los Alamos National Laboratory, Los Alamos, New Mexico, 87545, USA.

*Email: laurent.maron@irsamc.ups-tlse.fr; olaf.walter@ec.europa.eu; steve.liddle@manchester.ac.uk

Experimental and Computational Details

General

Caution! Compounds of the ^{237}Np radionuclide decay principally through α-emission (Q_α = 4.958 MeV), however, a long half-life ($t_{1/2}$ = 2.144(7) × 10^6 years) of the isotope commands its low specific activity (a = 26.04 MBq g^{-1}). ^{237}Np establishes a secular equilibrium (asymptotical concentration at 34.6 ppb) with the potent β-emitter ^{233}Pa ($t_{1/2}$ = 26.975(13) days, a = 777 TBq g^{-1}), which pertains also to the intense γ-ray emission. Handling of $^{237}Np/^{233}Pa$ compounds should be carried out in specifically equipped facilities, such as α-standard gloveboxes (as in this work), and within regulated radiological establishments under strict regulatory oversight.

Materials and Methods

For transuranium work argon N6.0 was purified by passage through Agilent Technologies Big Moisture and Oxygen Traps. For uranium work dinitrogen was dried and deoxygenated by passage through activated molecular sieves and copper oxide catalyst dispersed over a porous aluminium support. Solvents were pre-dried over activated 3 Å molecular sieves then distilled from NaK_2 or Na/Ph_2CO, or passed through activated alumina towers and degassed before use. Solvents were stored over potassium mirrors except for THF which was stored over activated 4 Å sieves. NMR solvents were dried over NaK_2 alloy in the presence of 1:1 mixture of benzophenone:tetraethylene glycol dimethyl ether for 48 h, or molten potassium, and vacuum-transferred prior to use. [Np($Tren^{TIPS}$)Cl] (**6**, $Tren^{TIPS}$ = $\{N(CH_2CH_2NSiPr^i_3)_3\}^{3-}$),[1] [Np($Tren^{TIPS}$)] (**1**),[1,2] [U($Tren^{TIPS}$)($NSiMe_3$)] (**3UNSiMe$_3$**),[3] KC_8,[4] LiN(H)Ad,[5] $AgBPh_4$,[6] and [$N(C_6H_4$-4-Br$)_3$][$B(C_6F_5)_4$][7] were prepared as described previously. Me_3SiN_3 was vacuum distilled from CaH_2, AdN_3 was fractionally sublimed *in vacuo*, and 2.2.2-cryptand was dried under vacuum for 24 hr before use. Unsealed transuranium compounds were manipulated in N_2-filled (<0.3% O_2, <50 ppm H_2O), negative-pressure, α-tight radiological gloveboxes. The glovebox for preparative chemistry was fitted with an automated dual vacuum/argon manifold and standard Schlenk techniques were used with silylated (hexamethyldisilazane)

glassware. Uranium compounds were manipulated using Schlenk line or positive-pressure glove box techniques (O_2 and H_2O < 0.1 ppm). All preparative experiments were conducted at least twice to ensure reproducibility, at the representative scale, unless stated otherwise.

SC-XRD measurements were performed on a Bruker Apex II Quazar diffractometer with monochromated MoKα-radiation (λ = 0.71073 Å) or a Rigaku FR-X diffractometer with mirror-monochromated CuKα-radiation (λ = 1.54184 Å) and specific collection parameters are detailed in the respective CIF files. Data integration and empirical absorption corrections were done using SAINT/SADABS[8,9] or CrysAlisPro.[10] The structures were solved either by direct or dual methods using SHELXS or SHELXT,[11,12] and all non-hydrogen atoms were refined by full-matrix least-squares on all unique F^2 values with anisotropic displacement parameters with exceptions noted in the respective CIF files. Except where noted, hydrogen atoms were refined with constrained geometries and riding thermal parameters. Refinements used SHELXL and Olex2.[13,14] ORTEP-3 and POV-Ray were employed for molecular graphics.[15,16] NMR spectra were recorded on a Bruker Ascent™ 400 MHz WB NMR/DNP spectrometer equipped with an inverse Z-gradient Bruker Triple Resonance Broad Band Probe (TBI) or a Bruker Avance II 400 MHz spectrometer. Transuranium samples were sealed under inert conditions in a silanized quartz capillary which was covered by and closed by a molten perfluorinated polymer. The sealed liner was then transferred into a standard borosilicate glass NMR tube of which the upper part was placed in a PVC bag. The bag, which was connected to the sample glovebox, was then welded. By this procedure, the sample can be measured applying standard NMR experiments. Alternatively, the relevant solution was loaded into a fresh FEP NMR liner that was protected from surface contamination with Parafilm while inside a transuranium glovebox. The liner was sealed with two PTFE plugs, brought out of the glovebox, and verified to be free of surface contamination before the parafilm was removed. The liner was then loaded into a J. Young tap appended 5 mm NMR tube, the headspace was then evacuated and refilled with He to provide an inert atmosphere headspace above the sample. Uranium samples were measured in 5mm

Youngs tap NMR tubes. Chemical shifts were calibrated against residual protio solvent resonances and are calibrated relative to tetramethylsilane (δ = 0 ppm). Transuranium and uranium FT-IR data were recorded on Bruker Alpha II spectrometers fitted with single reflection Platinum-ATR (diamond) modules with the spectrometers placed housed in a glovebox. UV/Vis/NIR measurements were performed using Agilent CARY 5000 or Perkin Elmer Lambda 750 spectrometers, for transuranium and uranium samples respectively, in cuvettes of 10 mm thickness with the sample compartment in a radiologically shielded glovebox for transuranium samples. For EPR spectroscopy, toluene solutions were loaded into a fresh FEP EPR liner that was protected from surface contamination with Parafilm while inside a transuranium glovebox. The liner was sealed with two PTFE plugs, brought out of the glovebox, and verified to be free of surface contamination before the parafilm was removed. The liner was then loaded into an FEP liner that was then inserted into a standard 4 mm quartz EPR tube. Samples were kept frozen once removed from the glovebox and loaded frozen into the EPR spectrometer. Spectra were recorded on a Magnettech MS5000 (currently marketed as ESR5000) X-band spectrometer. Temperature control was achieved using an Advanced Research Systems LTR helium flow cryostat in combination with a lakeshore temperature controller. Spectra were recorded using a microwave power of ~30 mW and a 1 mT modulation amplitude. Variable-temperature magnetic measurements were variously performed on Quantum Design MPMS3 superconducting quantum interference device (SQUID) and PPMS-14T alternating gradient field vibrating sample magnetometers using recrystallized samples in an applied field of $\mu_0 H$ = 7 T. Data reproducibility was carefully checked, and care was taken to ensure complete thermalization of the sample before each data point was recorded. Diamagnetic corrections were applied using tabulated Pascal constants and measurements were corrected for the effect of the blank PCTFE sample holder. The magnetic data were modelled with the CONDON 3.0 program.[17] Elemental microanalyses for uranium compounds were carried out by Mr Martin Jennings and Mrs Anne Davies at The University of Manchester Department of Chemistry.

Reaction of [Np(Tren^{TIPS})] (1) with N_3SiMe_3 and isolation of [Np(Tren^{TIPS}){N(H)SiMe$_3$}] (4a) and [Np(Tren$^{TIPS-2H}$){N(H)SiMe$_3$}] (4b, Tren$^{TIPS-2H}$ = {N(CH$_2$CH$_2$NSiPr$^i_3$)$_2$(NCH$_2$CH$_2$NSiPr$^i_2$C[Me] =CH$_2$)}$^{3-}$)

A 12-mL Schlenk tube equipped with a glass-coated AlNiCo stirrer bar was charged with **1** (55.90 mg, 65.8 μmol) dissolved in 2 mL of toluene, or n-hexane, and precooled to −74 °C with dry ice/methanol bath. N_3SiMe_3 (8.65 μL, 65.8 μmol) was added at once with a 10-μL Hamilton® GASTIGHT® syringe (N.I.S.T. traceable). The reaction mixture was allowed to warm for 1.5 hr to 20 °C. During this process, the color changed to purplish-brown at *ca.* −45 °C and then to dark brown at −15 °C, accompanied by gas evolution. Finally, the color faded to red-orange on storage above 10 °C. After removal of trace off-white turbidity by filtration, the volume of the clear solution was adjusted by slow isothermal evaporation of the solvent at 40 °C *in vacuo* to induce crystallization inside the resulting solution (~0.45 mL) on its withdrawal from the heating bath and followed by slow rotational motion of the Schlenk tube along its main axis while maintaining nearly horizontal alignment. The resulting intense red-orange needles of **4a/4b**, suitable for single crystal X-ray diffractometry, were isolated by direct absorption using silanized glass microfiber filter strips (Grade GF/C™) to remove the mother liquor. Yield: 33.0 mg, 53.5%. NMR (D_6-benzene, 298 K; for rationale of assignment see later)/ ppm ^{1}H,^{13}C: δ 3.4, −223.4 (m, 2H, NCH$_2$C$\underline{H}_a$H, **4b**); −0.1, −223.4 (m, 2H, NCH$_2$C$\underline{H}_b$H, **4b**); 47.7, −62.2 (m, 6H, NC*H*$_2$, **4a**); −2.0, −233.8 (m, 6H, NCH$_2$C*H*$_2$, **4a**); 52.4, 66.5 (m, 2H, NC$\underline{H}_a$H, **4b**); 49.9, 66.5 (m, 2H, NCH$\underline{H}_b$, **4b**); 45.0, 53.3 (m, 2H, NC$\underline{H_2}$, **4b**); −4.2, −240.0 (m, 2H, NCH$_2$C*H*$_2$, **4b**); −8.0, −7.10 (m, 2H, =C*H*$_2$, **4b**); 5.3, 9.9/11.8 (m, 6H, C*H*, **4a/b**); −8.5/−8.6, −7.0/−9.2 (br, 26H, C*H*$_3$, **4a/b**); 3.9, 24.8 (m, 1H, C*H*, **4a/b**); 1.0, 11.8/16.5/19.1 (m, 6H, C*H*, **4a/b**); −5.0, 10.4 (m, 3H, C*H*, **4a/b**); 5.5, 56.1 (m, 1H, C*H*, **4a/b**); −9.0/−9.2, −9.0/−6.9 (br, 58H, C*H*$_3$, **4a/b**); −10.4, −9.2 (br, 23H, C*H*$_3$, **4a/b**). FTIR (ATR) ν/cm^{-1}: 3220 (w), 2940 (m), 2888 (m/w), 2862 (m), 2085* (m), 1461 (m), 1400 (w), 1387 (w), 1380 (w), 1365 (w), 1337 (w), 1273 (m), 1255 (m/w), 1242 (m), 1156 (vw), 1136 (w), 1103 (w), 1046 (m/s), 1019 (m), 1011 (m), 989 (m/w), 969 (w), 925

(s), 880 (s), 869 (s), 824 (m), 786 (m/s), 725 (vs), 671 (s), 631 (s), 595 (m/w), 563 (m), 544 (m/s), 512 (m/s), 447 (m/s), 430 (m), 426 (m), 421 (m), 416 (m), 400 (m/s). *Residual Me_3SiN_3. UV/Vis/NIR (*n*-hexane, 7.87 mM, 293 K) λ_{max}/nm (cm^{-1}; ε/dm^3 mol^{-1} cm^{-1}): 525 (19,048; 34.8), 539 sh (18,553; 24.6), 546 sh (18,315; 22.4), 564 (17,731; 31.7), 578 (17,301; 15.4), 586 (17,065; 15.1), 645 sh (15,504; 27.4), 653 (15,314; 28.3), 669 (14,948; 26.4), 724 (13,812; 29.1), 755 (13,245; 52.1), 764 (13,089; 56.1), 777 (12,870; 38.8), 795 (12,579; 27.9), 856 (11,682; 39.0), 886 (11,286; 59.3), 938 (10,661; 67.2), 958 (10,438; 60.0), 1001 (9,990; 30.4), 1022 (9,785; 17.0), 1241 (8,058; 4.3), 1294 (7,728; 4.8), 1587 (6,301; 15.7). * Residual N_3SiMe_3.

Reaction of [Np(Tren^{TIPS})] (1) with N_3Ad and putative isolation of [Np(Tren^{TIPS}){N(H)Ad}] (5a) and [Np(Tren$^{TIPS-2H}$){N(H)SiMe$_3$}] (5b, Tren$^{TIPS-2H}$ = {N(CH$_2$CH$_2$NSiPr$^i_3$)$_2$(NCH$_2$CH$_2$NSiPr$^i_2$C-[Me]=CH$_2$)}$^{3-}$)

A 12-mL Schlenk tube equipped with a PTFE-coated stirrer bar was charged with **1** (31.8 mg, 37 μmol) and AdN_3 (7.39 mg, 42 μmol) and the solid reactants were cooled to −77 °C with dry ice/methanol bath. *n*-Hexane (3 mL) was carefully added down the cold side of the vessel and the stirred contents were allowed to react for 1 hr at this temperature. The wine red color of the resulting solution turned gradually to dark brown and gas evolution was observed. The stirred reaction mixture was allowed to warm for 1 hr to 20 °C, at which time it changed color to yellowish brown. After removal of the trace off-white turbidity by filtration, the volume of the clear solution was adjusted by slow isothermal evaporation of the solvent at 35 °C *in vacuo* to induce crystallization inside the resulting viscous solution (~0.20 mL) on its withdrawal from heating bath and followed by slow rotational motion of the Schlenk tube along its main axis while maintaining nearly horizontal alignment. The resulting dark red block crystals of **5a**/**5b** suitable for single-crystal X-ray diffraction analysis, were isolated by direct absorption using silanized glass microfiber filter strips (Grade GF/C™) to remove the mother liquor. Yield: 3.45 mg, 9.2%. Single-crystal X-ray diffractometry was

used for analysis, as it proved to be the only viable technique given the limitations in sample preparation and manipulation.

Attempted reaction of [Np(Tren^{TIPS})Cl] (6) with LiN(H)Ad

A 25-mL Schlenk tube equipped with a PTFE-coated stirrer bar was charged with **6** (56.0 mg, 63 μmol) and excess LiN(H)Ad (18.10 mg, 115 μmol) and the solid reactants were cooled to −77 °C with a dry ice/methanol bath. Toluene (4 mL) was carefully added down the cold side of the vessel and the stirred contents were allowed to react for 0.5 hr at this temperature. A red-orange supernatant with an off-white precipitate resulted. The stirred reaction mixture was allowed to warm for 1.5 hr to 20 °C indicating no visible changes in color or volume/density of the precipitate. After isolation of the supernatant by filtration, the volume of the clear red-orange solution was adjusted by slow isothermal evaporation of the solvent at 60 °C *in vacuo* to induce crystallization (~ 0.45 ml) on its withdrawal from heating bath. Large needle crystals of **6** were isolated and multiple specimens were examined by single-crystal X-ray diffraction studies which confirmed the identity of **6** by unit cell checking. The parent reagents were recombined in 3.5 mL of fresh toluene and heated to 80 °C in a closed vessel for 2.5 hr. No visible changes were observed in the reaction mixture, and crystallization attempts from the clear supernatant solution yielded only starting compound **6** (48.1 mg, 86% total recovery) as the sole identified Np-containing product.

Reaction of [Np(Tren^{TIPS})Cl] (1), N_3SiMe_3, KC_8, and 2.2.2-cryptand to give [Np(Tren^{TIPS})(μ-Cl)K(2.2.2-cryptand)] (7)

Complex **6** (31.0 mg, 35 μmol), N_3SiMe_3 (4.1 mg, 36 μmol) and 2.2.2.-cryptand (13.2 mg, 35 μmol) were combined in a 20 mL borosilicate vial with a PTFE-coated magnetic stirrer bar, THF added (1.5 mL) and the mixture cooled to –35 °C. KC_8 (10.4 mg, 77 μmol) was added as a solid to the cooled mixture resulting in a slow color change from orange-red to ruby-red with the mixture kept cool in an aluminum block. Consistent with a reduction reaction, the KC_8 turned from brown to black. The

mixture was allowed to warm to ambient temperature then stirred for 40 minutes. The mixture was filtered through a glass-fiber filter disc packed into a borosilicate pipette. Volatiles were removed from the filtrate *in vacuo* to afford a red oil, which persisted despite attempts to triturate with pentane and Et_2O/pentane. The oil was dissolved in toluene, filtered through a glass-fiber filter disc packed into a borosilicate pipette, and volatiles removed in vacuo to form a foam. The foamy material was dissolved in Et_2O (0.1 mL) and stored at −35 °C. After 12 days, ruby-red blocks, determined by single-crystal X-ray diffraction to be **7**, were isolated. Yield: 11.2 mg, 25%. ^{1}H NMR (D_6-benzene, 298 K): δ 0.22 (d, $^3J_{HH}$ = 7 Hz, 54H, SiCHCH_3CH_3), 1.66 (m, 9H, SiCHCH$_3$CH$_3$), 2.92 (br, 12H, NCH_2CH$_2$-cryptand), 3.99 (br, 12H, NCH$_2$CH_2-cryptand), 4.13 (br, 12H, OCH_2CH_2O), 6.79 (br, 6H, NCH_2CH$_2$), 10.81 (br, 6H, NCH$_2$CH_2) ppm. ^{29}Si{^{1}H} NMR (D_6-benzene, 298 K): 7.90 (s, *Si*(CHCH$_3$CH$_3$)$_3$) ppm. UV/Vis/NIR (toluene): λ_{max}/nm (cm^{-1}; ε/dm^3 mol^{-1} cm^{-1}): 792 (12,630; 28) 497 (20,130; 18) 468 (21,385; 20) 448 (22,325; 16) 431 (23,180; 17) 421 (23,750; 22) 410 (24,405; 35) 387 (25,835; 44) 380 (26,325; 25) 372 (26,850; 30) 342 (29,210; 26) 338 (29,545; 25) 301 (33,255; 564, br. sh.) 288 (34,745; 1,376) 262 (38,220; 1,986). ATR-IR was not attempted due to material handling limitations.

Reaction of [Np(Tren^{TIPS})] and N$_3$SiMe$_3$ in D$_6$-benzene

A 12-mL Schlenk tube equipped with a glass-coated AlNiCo stirrer bar was charged with **1** (45.62 mg, 53.7 μmol) and dissolved in 2 mL of D_6-benzene (≥ 99.98 at.% D). To the stirred resulting solution N_3SiMe_3 (7.10 μL, 54.0 μmol) was added at once with the aid of a 10-μl Hamilton® GASTIGHT® syringe (N.I.S.T. traceable). Immediate gas effervescence and change of color to dark brown were observed. The reaction mixture was allowed to react for further 15 hr, during which time the color gradually faded and turned to orange. After removal of the trace off-white turbidity by filtration, the volume of the clear solution was adjusted by slow isothermal evaporation of the solvent at 40 °C *in vacuo* to induce crystallization inside the resulting solution (~0.40 ml) on its withdrawal from the heating bath and followed by slow rotational motion of the Schlenk tube along its main axis

while maintaining nearly horizontal alignment. The resulting intense red-orange needles of **4a/4b** were isolated by direct absorption using silanized glass microfiber filter strips (Grade GF/C™) to remove the mother liquor. The sample was found to be identical, by ^{1}H and ^{13}C NMR spectroscopies and single-crystal X-ray diffractometry, to authentic **4a/4b** material. No ^{2}D NMR resonance indicating D-incorporation was detected in the 48.1 mg/ml solution in D_6-benzene. The representative procedure was employed to isolate pure **4a/4b** from C_6H_6. In one instance, **4a** was produced as pale orange crystalline material in low yield and characterized by single-crystal X-ray diffractometry. Attempts to characterize this material further were precluded by the low yield.

Preparation of [K(2.2.2-cryptand)][U(Tren^{TIPS})(NSiMe$_3$)] (8UNSiMe$_3$)

Benzene (40 mL) was added to a pre-cooled mixture of **3UNSiMe$_3$** (0.80 g, 0.85 mmol) and KC_8 (0.13 g, 1.00 mmol) at –78 °C in a Schlenk flask. The mixture was warmed up to room temperature and stirred for 24 hrs, during this time, the reaction turned into a dark red brown mixture. A solution of 2.2.2-cryptand (0.33 g, 0.88 mmol) in benzene (10 mL) was added to the mixture and the reaction was stirred at room temperature for 4 hours, resulting in a pink solution with oily product formed at the bottom of the Schlenk flask. Volatiles were removed *in vacuo* to afford an oily red residue which was extracted with THF (20 mL) and filtered. Volatiles were removed again from the filtrate *in vacuo* to afford an oily red residue which was washed with Et_2O (2 × 10 mL) to give **8UNSiMe$_3$** as **a** pink solid after drying *in vacuo*. Yield: 0.83 g, 72%. Red crystals of **8UNSiMe$_3$** suitable for a single crystal X-ray diffraction study were obtained by storing the Et_2O washings at –35 °C for 3 days. Anal. Calcd for $C_{54}H_{120}N_7KO_6Si_4U$: C, 47.94; H, 8.94; N, 7.25%. Found: C, 48.10; H, 9.30; N, 7.06%. ^{1}H NMR (D_8-THF, 298 K): δ –25.47 (br, 54H, SiCHCH_3CH_3), –23.50 (br, 9H, SiCHCH$_3$CH$_3$), 3.09 (t, 12H, NCH_2CH$_2$-cryptand), 4.09 (t, 12H, NCH$_2$CH_2-cryptand), 4.14 (br, 12H, OCH_2CH_2O), 19.33 (br, 6H, NCH_2CH$_2$), 59.45 (br, 9H, Si(CH_4)$_3$), 112.02 (br, 6H, NCH$_2$CH_2) ppm. ^{29}Si{^{1}H} NMR (D_8-THF, 298 K): not observed. ATR-IR ν/cm^{-1}: 2934 (w), 2880 (w), 2852 (s), 1479 (w), 1459 (w), 1445 (w), 1355 (m), 1300 (w), 1259 (w), 1221 (w), 1134 (s), 1105 (s), 1082 (s), 1039 (s), 1011 (m), 932 (s), 881 (m),

834 (w), 813 (m), 794 (m), 739 (vs), 669 (s), 625 (m), 564 (w), 539 (w), 508 (m), 440 (w). UV/Vis/NIR (THF) λ_{max}/nm (cm^{-1}; $\varepsilon/dm^3\ mol^{-1}\ cm^{-1}$): 484 (20,661; 72), 504 (19,841; 154), 534 (18,729; 115), 564 (17,731; 38), 582 (17,182; 34), 414 (16,287; 32), 638 (15,674; 33), 706 (14,164; 41), 754 (13,263; 42), 770 (12,987; 48), 788 (12,690; 46), 806 (12,407; 44), 880 (11,364; 32), 956 (10,460; 61), 1,022 (9,784; 49), 1,090 (9,174; 47), 1,118 (8,945; 33), 1,178 (8,489; 47), 1,300 (7,692; 100), 1,370 (7,299; 63), 1,458 (6,859; 41), 1,538 (6,502; 42), 1,712 (5,841; 231), 1,810 (5,525; 104), 1,948 (5,133; 57). Complex **8UNSiMe$_3$** is stable in THF or Et_2O, and is very soluble in THF and slightly soluble in Et_2O, but forms oils in benzene. A thermal stability test of the complex in D_8-THF at 80 °C for one week shows no signs of decomposition.

Attempted oxidation of [U(Tren^{TIPS})(NSiMe$_3$)] (3UNSiMe$_3$) with AgBPh$_4$

THF (5 mL) was added to a solid mixture of **3UNSiMe$_3$** (0.022 g, 0.023 mmol) and $AgBPh_4$ (0.010 g, 0.023 mmol) in a 20 mL glass scintillation vial at room temperature. The mixture was stirred for 24 hours, during which time a small amount of off-white solid precipitated from solution. The mixture was filtered to obtain a dark red, clear solution, before volatiles were removed *in vacuo* to afford a dark red oil. NMR spectroscopic analysis of the crude reaction mixture showed the presence of **3UNSiMe$_3$** as the main component. ^1H NMR (C_6D_6, 298 K): δ 21.45 (s, 6H, CH_2), 11.37 (s, 9H, CH(CH_3)$_2$), –2.93 (s, 54H, CH(CH_3)$_2$), –3.34 (s, 6H, CH_2) ppm.

Attempted oxidation of [U(Tren^{TIPS})(NSiMe$_3$)] (3UNSiMe$_3$) with [N(C$_6$H$_4$-4-Br)$_3$][B(C$_6$F$_5$)$_4$]

1,2-difluorobenzene (5 mL) was added to a solid mixture of **3UNSiMe$_3$** (0.030 g, 0.032 mmol) and $[N(C_6H_4\text{-}4\text{-}Br)_3][B(C_6F_5)_4]$ (0.037 g, 0.032 mmol) in a 20 mL glass scintillation vial at room temperature. The mixture was stirred for 24 hours, during which time a small amount of brown oil precipitated from solution. The mixture was filtered to obtain a dark red, clear solution, before volatiles were removed *in vacuo* to afford a dark red oil. NMR spectroscopic analysis of the crude reaction mixture showed the presence of **3UNSiMe$_3$** as the main component, in addition to H_3Tren^{TIPS}

and $N(C_6H_4Br)_3$. ^{1}H NMR (C_6D_6, 298 K): δ 21.61 (s, 6H, C*H*$_2$), 11.45 (s, 9H, C*H*(CH_3)$_2$), –2.96 (s, 54H, CH(C*H*$_3$)$_2$), –3.36 (s, 6H, C*H*$_2$) ppm.

NMR assignment of 4a/4b

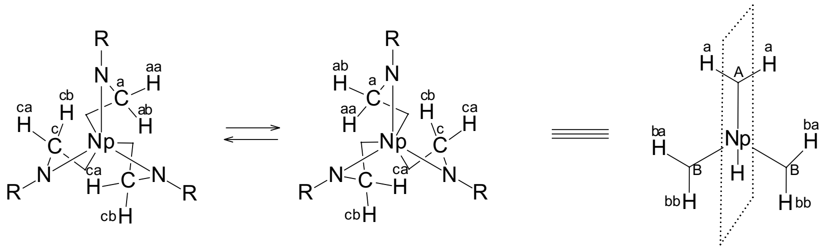

Figure S1. Reference frame for NMR assignment of **4a**/**4b**.

Complex **4a** would be expected to have three-fold symmetry arising from a C_3 rotation axis along the N-Np-N axis with a minimum of five groups of resonances arising from CH groups plus one for the NH group. In contrast for the vinyl complex **4b** due to the three-fold symmetry being broken by the vinyl group we expect additional resonances and a more complex ^{1}H NMR spectrum to be observed, and indeed this is the case. Assuming dynamic exchange of the Tren-backbone linkages (interconversion of left to right hand screw), Figure S1, and hindered rotation around the molecular three-fold axis, the symmetry of the molecule can be simplified to the view as shown on the right of Figure S1 with one N-donor ligand with its CH_2 neighbor *trans* to the H of the amido group and two in *cis* positions. This results in 3 proton (a, ba, bb) and two carbon (A, B) resonances, as the protons of the *cis*-positioned CH_2-groups split into two resonances for the protons closer or more distant to the NH functionalization but both are bonded to the same C atom. Then, each of these will show the same relative two proton intensity in the ^{1}H NMR spectrum. In the list of resonances there is only cross peak in the HH correlation between the resonances at 47.7/-62.2 (^{1}H/^{13}C) and –2.0/–233.8 2 (^{1}H/^{13}C) ppm with a relative intensity of 6H each. These two resonances are well separated and can only be assigned to the ethylene bridges in the backbone of **4a**, where the symmetry is 3-fold, which results in the two observed resonances with identical intensity corresponding to 6H each. The

correlated ^{13}C shift of these resonances differs significantly from 62.2 ppm for the one with the proton resonance at 47.7 ppm to −233.8 ppm for the CH_2 group having the proton resonance at −2.0 ppm. Close to each of these isolated resonances, each time three pairs of resonances, with a comparable combination of $^{1}H/^{13}C$ shift, but with one third of the relative intensity are observed. All the CH functionalities of the Pr^i groups then gives rise to a family of resonances with comparable ^{1}H and ^{13}C shifts in the range of 6 to –6 ppm for the ^{1}H and 10 to 57 ppm for the ^{13}C, respectively. The overall intensity of 17H corresponds to the number of CH groups for **4a**/**4b**; this is consistent with the transformation of one $CH\text{-}CH_3$ of an Pr^i group to a $C{=}CH_2$ by dehydrogenation. Accordingly, the methyl groups of the Pr^i substituents show resonances with comparable shifts: for the protons the shifts cover a range between −7 to −11 ppm whereas the carbon resonances are between 3 and −10 ppm. The overall experimentally found intensity of 113H covers the expected number of 105H for overall 35 Me-groups over two molecules, and reflecting the formation of one vinyl group. Interestingly the terminal vinyl ($=CH_2$) resonates at −8.0 ppm (^{1}H) and −7.1 ppm (^{13}C) with an intensity of 2H in total in a comparable range to the methyl groups listed here. In agreement with this assignment the resonances at 21.5 and 21.0 ppm with an intensity corresponding to 9H each were assigned to the methyl groups of the $SiMe_3$ substituents of the $NpN(H)SiMe_3$ unit of **4a**/**4b** ; the chemical shifts for both the ^{1}H and ^{13}C nuclei of these units is significantly different compared to the one of the Pr^i residues located at the other silyl groups. This assignment is further confirmed by the phase sensitive NOESY spectrum, in which only few cross peaks are observed, as the mixing time was with 170 ms, so not long enough to give the Nuclear Overhauser Effect a lot of time to evolve. Longer mixing times did not show better cross peak intensities resulting from fast relaxation due to the paramagnetic nature of the systems. However, the observed cross peaks confirm through space interactions between the methyl and methine groups of the Pr^i substituents and the $SiMe_3$ group of the $NpN(H)SiMe_3$ unit located at 21 ppm. Finally, there are a pair of resonances with a relative intensity of 1H each not showing any cross peaks in the CH correlation; these two resonances are nicely separated with chemical shifts at 93.8 and 91.6 ppm and are assigned to the NH atoms of the

two molecules being present in the experimentally confirmed 1:1 ratio of **4a/4b**. This confirms that no activation of the solvent has taken place since the same outcome is observed when the reaction is carried out in deuterated benzene, which would lead to a N-D linkage, which would not be observable by ^{1}H NMR spectroscopy. The ^{2}D spectrum of the complex supports this negative finding as there is only one deuterium resonance observed arising from the solvent.

CONDON analysis

The DC magnetometry data for **4a/4b** and **8UNSiMe₃** were modelled with a full Hamiltonian approach using the CONDON 3.0 program.[17] In particular, the following Hamiltonian was employed.

$$\hat{H} = \sum_{k=2,4,6} F^k \hat{f_k} + \sum_{i=1}^{N} \zeta(\kappa \hat{l_i} \cdot \hat{s_i}) + \sum_{i=1}^{N} \left(B_2^0 \widehat{C_2^0}(i) + B_4^0 \widehat{C_4^0}(i) + B_6^0 \widehat{C_6^0}(i) + B_4^3 \widehat{C_4^3}(i) \right) + \sum_{i=1}^{N} \mu_B (\kappa \hat{l_i} \cdot g_e \hat{s_i}) \cdot \boldsymbol{B}$$

The four terms of the Hamiltonian account for the inter-electronic repulsion, the spin-orbit coupling, the crystal field and the Zeeman interaction, respectively. F^k represents the inter-electronic Slater-Condon parameters, ζ is the spin-orbit coupling constant, κ is the orbital reduction factor and B_k^q are the crystal field parameters. $\hat{f_k}$, $\hat{l_i}$, $\hat{s_i}$ and $\widehat{C_k^q}$ are the Slater-Condon, orbital momentum, spin momentum and crystal field operators, respectively. μ_B is the Bohr magneton, g_e is the free-electron g factor and $\boldsymbol{B}$ is the applied magnetic field. Free-ion parameters (Slater-Condon integrals and spin-orbit coupling constant) were taken from the literature and kept fixed during the procedure.[18,19] Crystal field parameters and orbital reduction factor were obtained through a fitting procedure of the experimental DC magnetometry data. Including additional trigonal terms such as B_4^{-3}, B_6^3, B_6^6, B_6^{-3} and B_6^{-6} leads to overparametrization without significantly improving the quality of the fit. The thermal evolution of the real and imaginary components of the magnetic susceptibility of Np was fitted using a single component Debye model.[20] The temperature dependence of the extracted

relaxation time τ was fitted using a two components model which accounts for temperature independent quantum tunnelling and a temperature dependent Raman processes.

$$\frac{1}{\tau(T)} = \frac{1}{\tau_{QTM}} + CT^n$$

DFT calculations

Calculations on **3NpNSiMe$_3$**, **3NpNAd**, **4a**, **4b**, **5a**, and the anion component of **8UNSiMe$_3$** were performed using coordinates derived from the crystal structure as the starting points. No constraints were imposed on the structures during the geometry optimizations. The calculations were performed using the Amsterdam Density Functional (ADF) suite version 2017 with standard convergence criteria.[21,22] The DFT geometry optimizations employed Slater type orbital (STO) triple-ζ-plus polarization all-electron basis sets (from the Dirac and ZORA/TZP database of the ADF suite).[23-25] Scalar relativistic approaches (spin-orbit neglected) were used within the ZORA Hamiltonian for the inclusion of relativistic effects and the local density approximation (LDA) with the correlation potential due to Vosko *et al* was used in all of the calculations.[26] Generalized gradient approximation (GGA) corrections were performed using the functionals of Becke and Perdew.[27,28] This approach is justified based on the prior use of this functional/basis set combination and the fact that is has compared favorably to NMR chemical shift verified B3LYP hybrid functional calculations.[29-31] Analytical frequency calculations were carried out within the ADF program. The Quantum Theory of Atoms in Molecules analysis was carried out within the ADF program.[32,33] We quote Nalewajski-Mrozek bond orders since they reproduce expected bond multiplicities reliably in polar heavy atom structures whereas Mayer bond orders for polar bonds often do not always conform with chemical intuition.[34] The ADF-GUI (ADFview) was used to prepare the plots of the electron density.

CASSCF calculations

Multi-reference calculations were performed using the MOLCAS 8.2 code.[35,36] The multi-configurational natures of **4a** and **3NpNSiMe$_3$** were elucidated by single-point relativistic complete

active space self-consistent field (CASSCF)[37] calculations on the reference geometries optimized with the BP86 exchange-correlation functional (see above). Scalar relativistic effects were taken into account with the second-order Douglas-Kroll-Hess Hamiltonian.[38,39] Atomic natural orbital type all-electron basis sets developed for relativistic calculations (ANO-RCC) were used with the contraction schemes of [26s23p17d13f5g3h]/[9s8p6d4f2g] for Np,[40] [14s9p4d3f2g]/[4s3p2d1f] for N,[41] [17s12p5d4f2g]/[4s3p1d] for Si,[41] [14s9p4d3f2g]/[3s2p1d] for C,[41] and [8s4p3d1f]/[2s1p] for H[42] corresponding to valence triple-zeta plus polarization (VTZP) quality for Np and the coordinated N atoms, with VDZP quality for Si, C and H atoms. The large non-truncated complexes **3NpNSiMe$_3$** and **4a** were computed using a minimal active space consisting of the seven 5f orbitals occupied by two and three electrons, denoted as (2,7) and (3,7), respectively. Such minimal (n,7) active spaces (n = number of 5f electrons) has been successfully applied in previous SO-CASSCF studies of actinide complexes.[43-45] More sophisticated multireference calculations were performed on geometry-optimized truncated model structures with hydrogen atoms instead of the methyl groups in the parent molecules: [Np{N(CH$_2$CH$_2$NSiH$_3$}(NSiH$_3$)] and [Np{N(CH$_2$CH$_2$NSiH$_3$}(NHSiH$_3$)]. These truncated structures (possessing C_3 and C_1 symmetries, respectively) provided ground electronic state properties similar to the parent molecules (see in Table S1). The smaller sizes facilitated examination of the large active spaces (12,16) and (13,16), respectively. In these active spaces the populations of the formally 2e-orbitals proved to be very high (≥ 1.97 e) whereas those of the formally 0e-orbitals very low (≤ 0.03 e), confirming the relevance of the minimal active spaces. Additional calculations on the truncated structures included second-order perturbation theory (CASPT2)[46,47] accounting for dynamic electron correlation and determination of the spin-orbit (SO) ground state. The latter procedure was performed using the complete active space state interaction (CASSI) method,[48] which allows CASSCF wave functions for different electronic states to interact under the influence of a spin-orbit Hamiltonian. In the state-averaged calculations all the states of the high-spin and low-spin Np were considered, i.e. 21 and 28 roots for [Np{N(CH$_2$CH$_2$NSiH$_3$}(NSiH$_3$)] and 35 and 112 roots for [Np{N(CH$_2$CH$_2$NSiH$_3$}(NHSiH$_3$)], respectively.

Reaction profile calculations

All calculations were performed using Gaussian09 suite of programs[49] using the Becke's 3-parameter hybrid functional[50] combined with the non-local correlation functional provided by Perdew/Wang.[51] The Np, U and Si atoms were represented with a small-core Stuttgart-Dresden relativistic effective core potential associated with their adapted basis set.[52-54] Additionally, the Si basis set was augmented by a d-polarization function ($\alpha = 0.284$)[55] to represent the valence orbitals. All the other atoms C, N and H were described with a 6-31G (d,p), double –ζ quality basis set.[56,57] The nature of the extrema (minimum) was established with analytical frequencies calculations and geometry optimizations were computed without any symmetry constraints. Intrinsic Reaction Paths (IRPs)[58,59] were traced from the various transition structures to obtain the connected intermediates. The enthalpy energy was computed at T = 298 K in the gas phase.

Figures

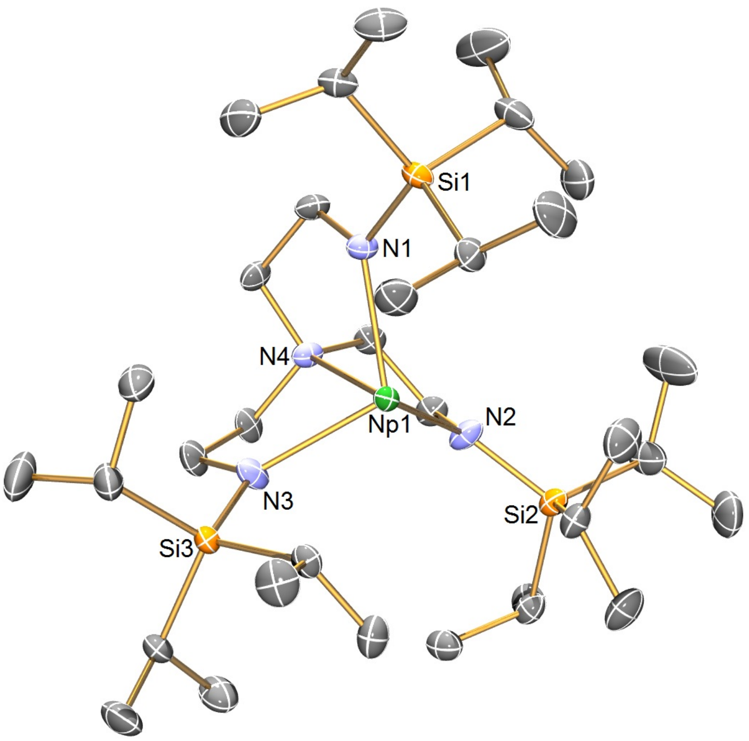

Figure S2. Molecular structure of **1** at 140 K with selective labelling and displacement ellipsoids at 40%. Hydrogen atoms and disordered components are omitted for clarity.

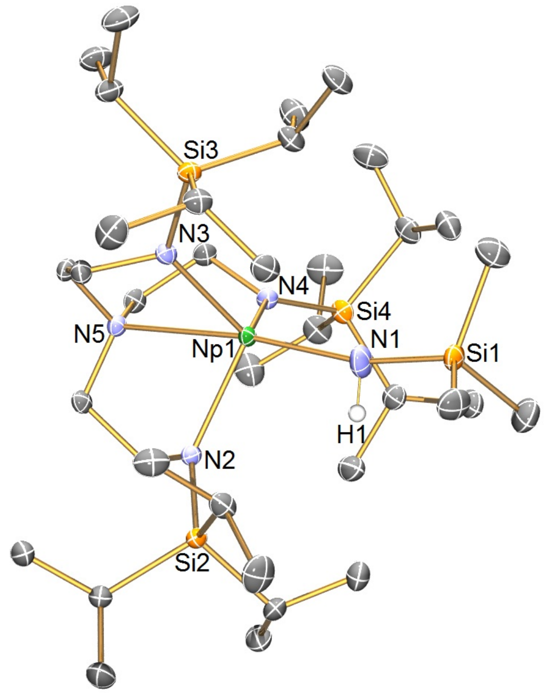

Figure S3. Molecular structure of **4a** from the **4a**/**4b** co-crystal at 150 K with selective labelling and displacement ellipsoids at 30%. Carbon-bound hydrogen atoms and disordered components are omitted for clarity.

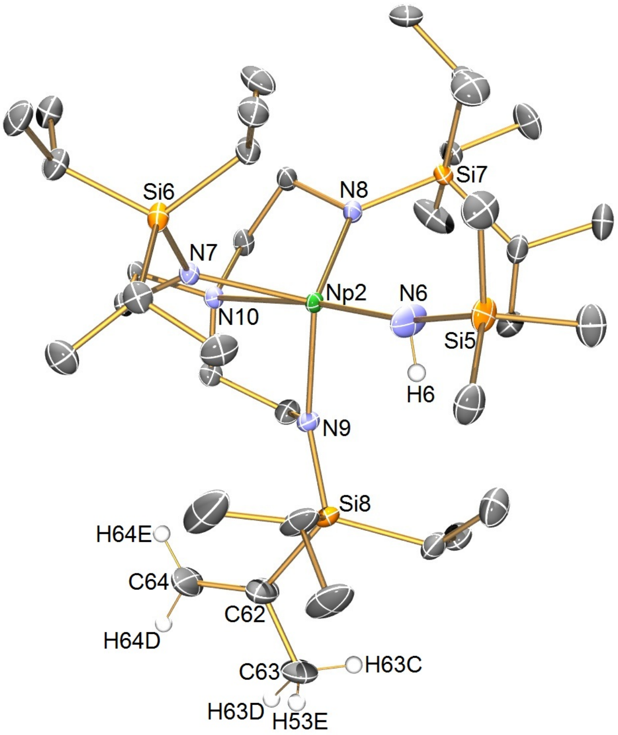

Figure S4. Molecular structure of **4b** from the **4a**/**4b** co-crystal at 150 K with selective labelling and displacement ellipsoids at 30%. Hydrogen atoms, except for the amido-bound and dehydrogenated *iso*-propyl group, and disordered components are omitted for clarity.

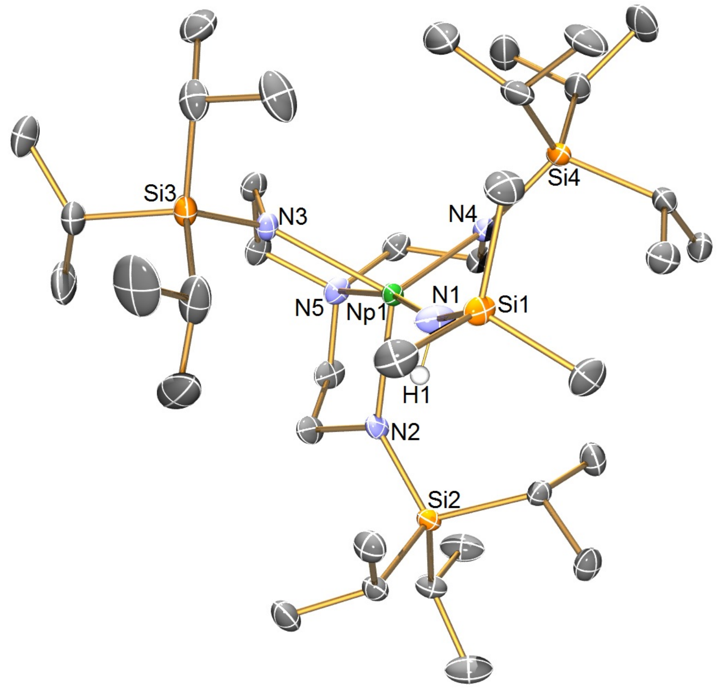

Figure S5. Molecular structure of **4a** from a pure crystal of **4a** at 110 K with selective labelling and displacement ellipsoids at 30%. Hydrogen atoms, except for the amido-bound and dehydrogenated *iso*-propyl group, and disordered components are omitted for clarity.

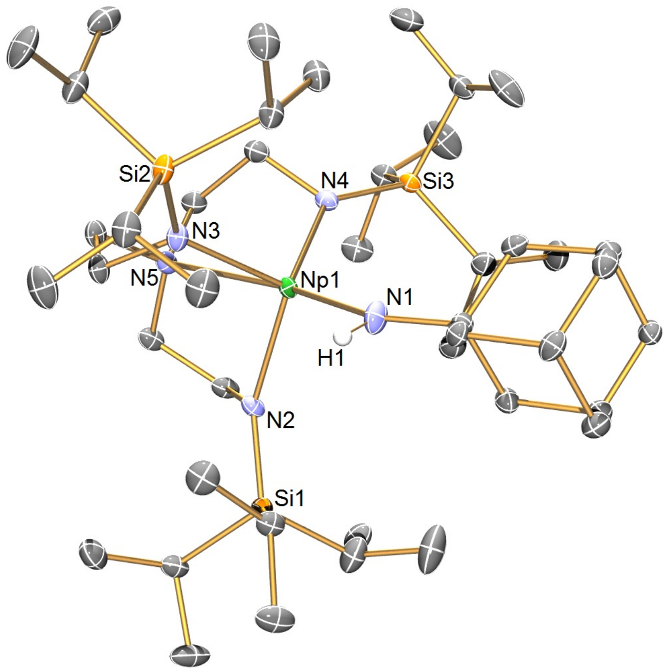

Figure S6. Molecular structure of **5a** at 150 K with selective labelling and displacement ellipsoids at 30%. Carbon-bound hydrogen atoms and disordered components are omitted for clarity. The other five molecules of **5a** in the asymmetric unit are apparently very similar so are not shown.

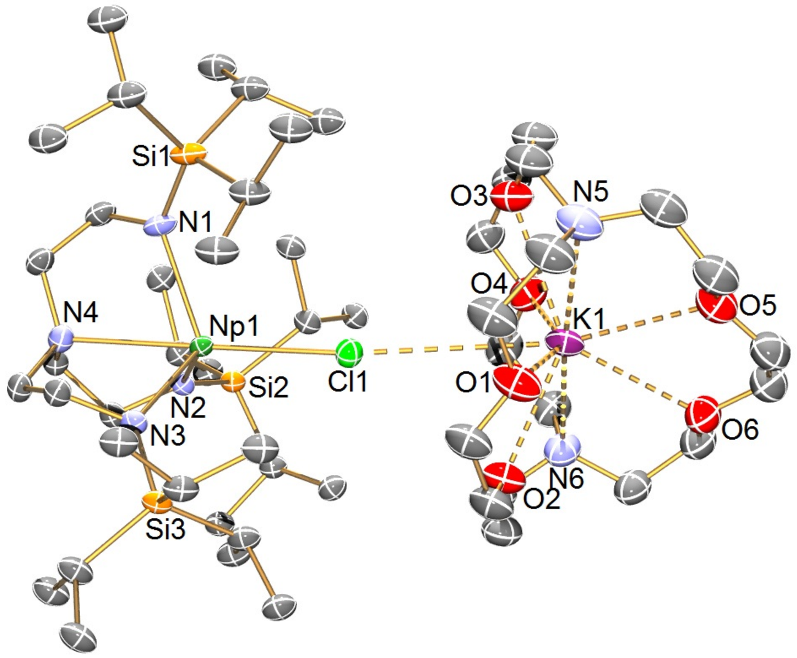

Figure S7. Molecular structure of **7** at 100 K with selective labelling and displacement ellipsoids at 20%. Hydrogen atoms and disordered components are omitted for clarity.

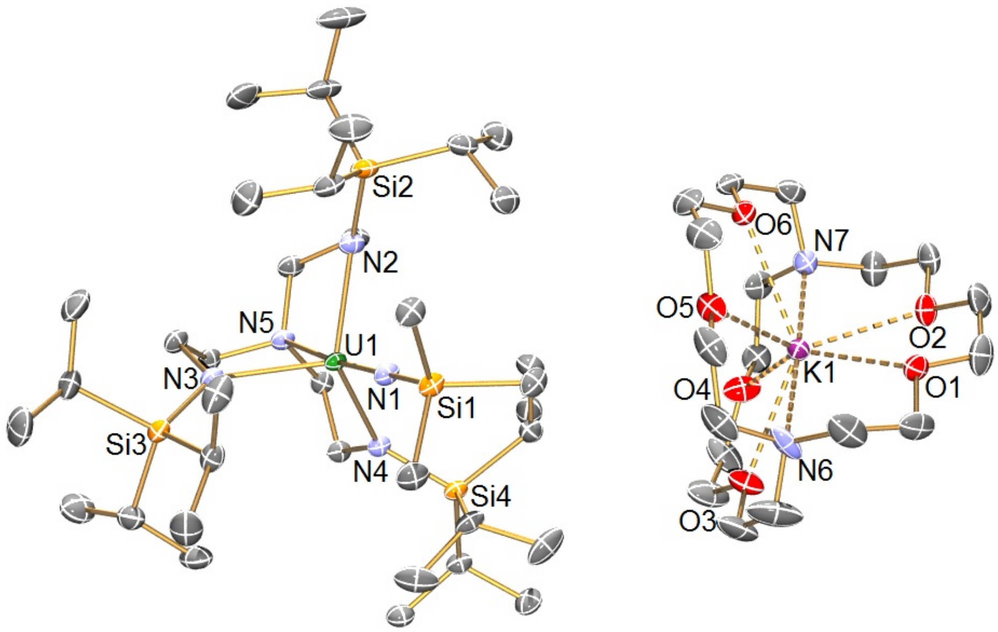

Figure S8. Molecular structure of **8UNSiMe$_3$** at 100 K with selective labelling and displacement ellipsoids at 40%. Hydrogen atoms and disordered components are omitted for clarity.

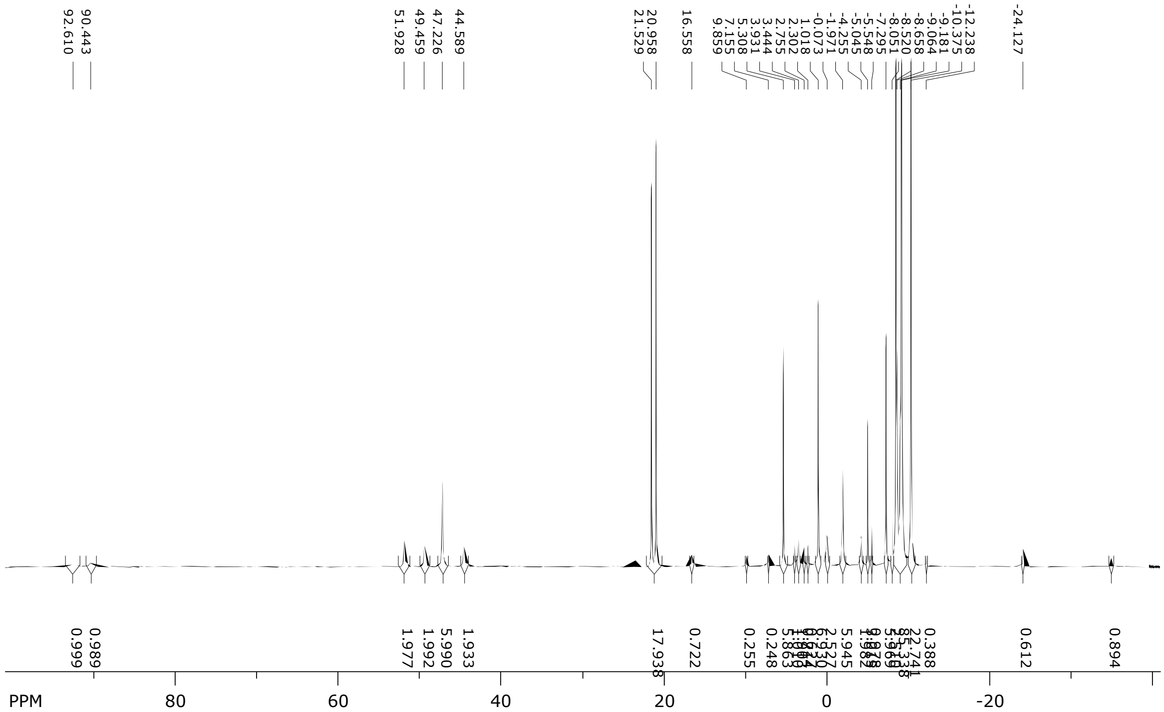

Figure S9. ^{1}H NMR (D_6-benzene, 298 K) spectrum of **4a**/**4b**.

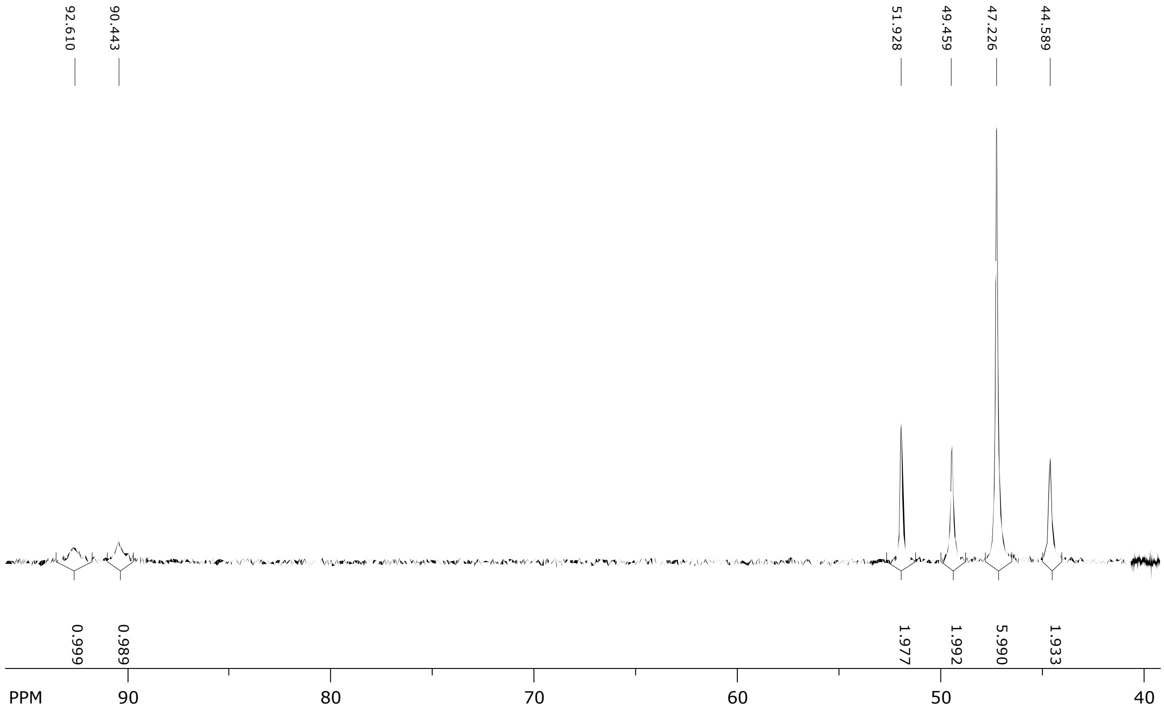

Figure S10. Zoom-in of the ^{1}H NMR (D_6-benzene, 298 K) spectrum of **4a**/**4b**.

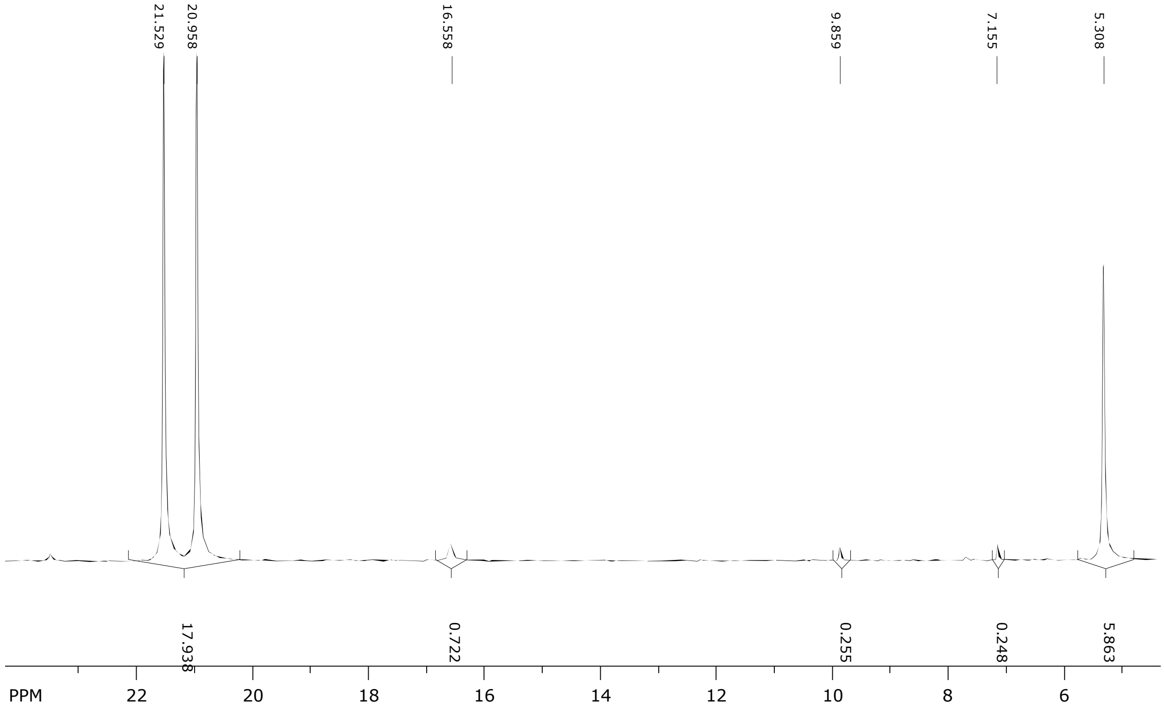

Figure S11. Zoom-in of the ^{1}H NMR (D_6-benzene, 298 K) spectrum of **4a**/**4b**.

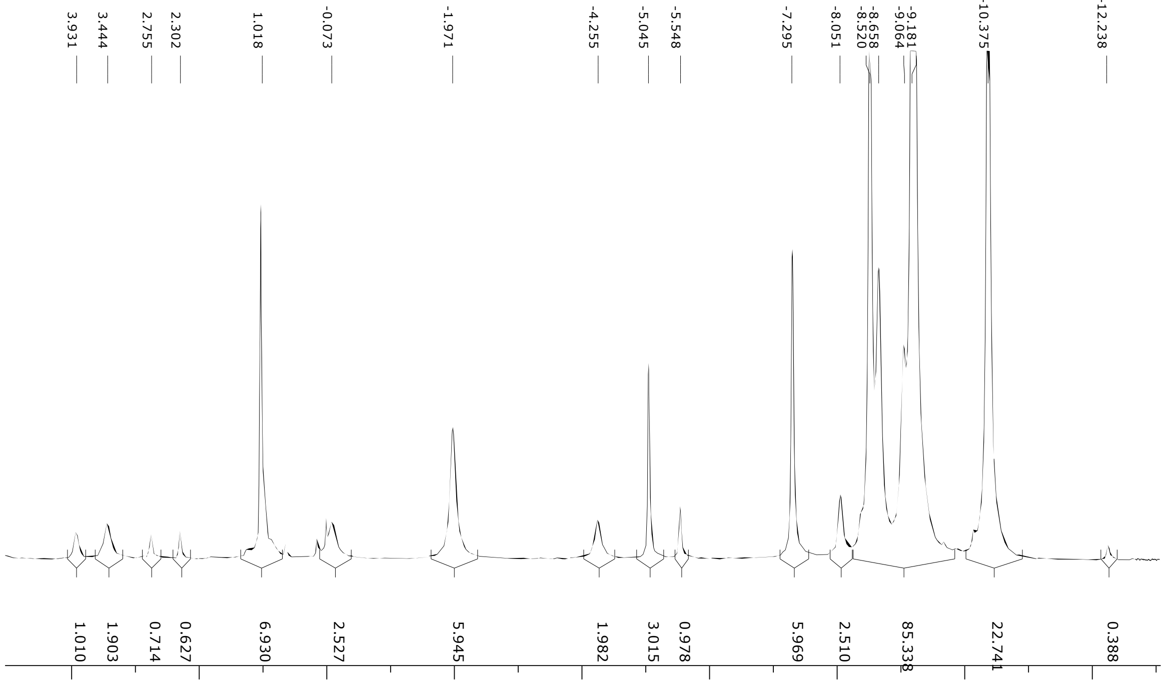

Figure S12. Zoom-in of the ^{1}H NMR (D_6-benzene, 298 K) spectrum of **4a**/**4b**.

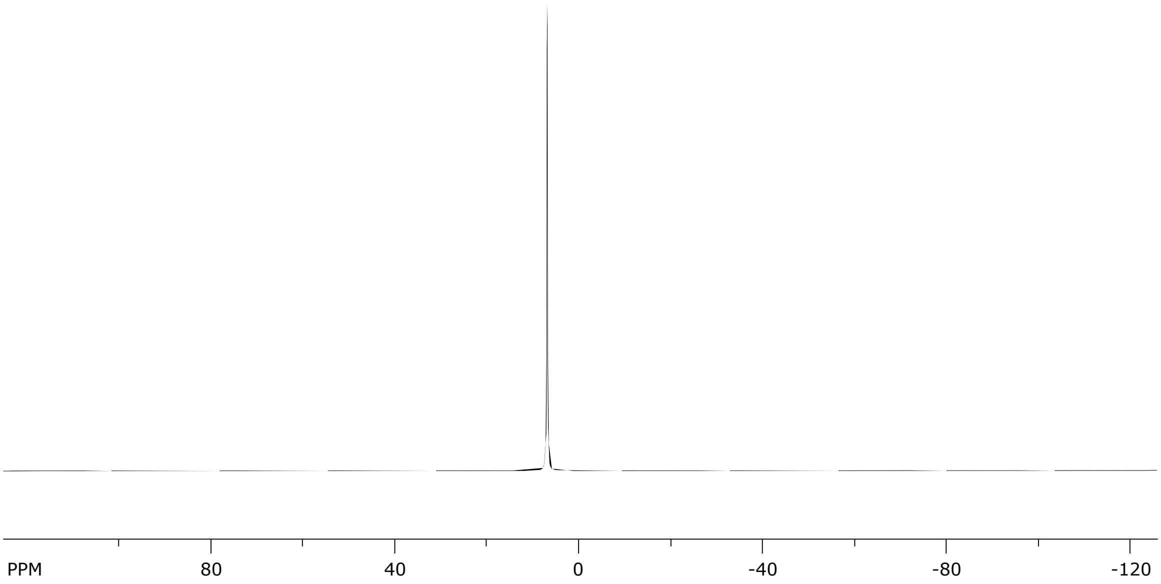

Figure S13. ^{2}H NMR spectrum of **4a**/**4b** prepared in D-solvent (D_6-benzene) showing only solvent resonance.

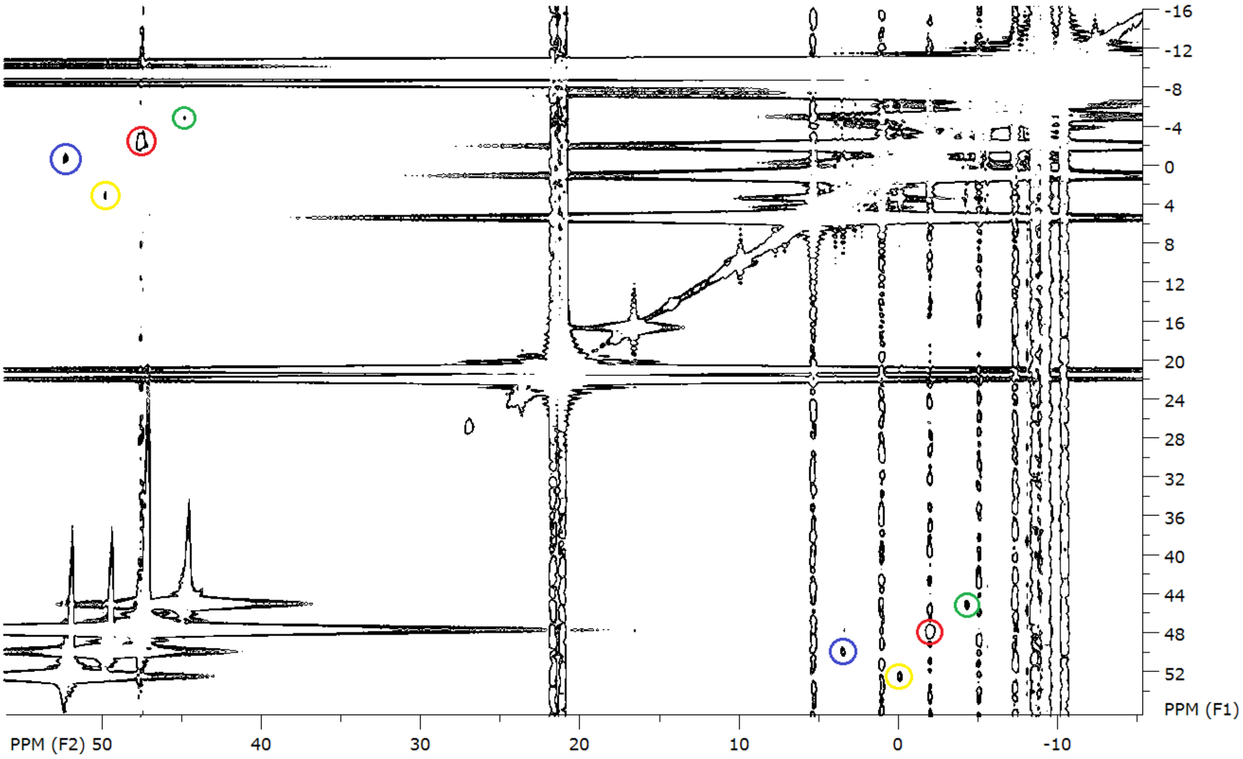

Figure S14. HH CORR of **4a**/**4b**, gradient COSY (D_6-benzene, 298 K), with four marked COSY resonances assigned as the Tren backbone carbons for **4b.**

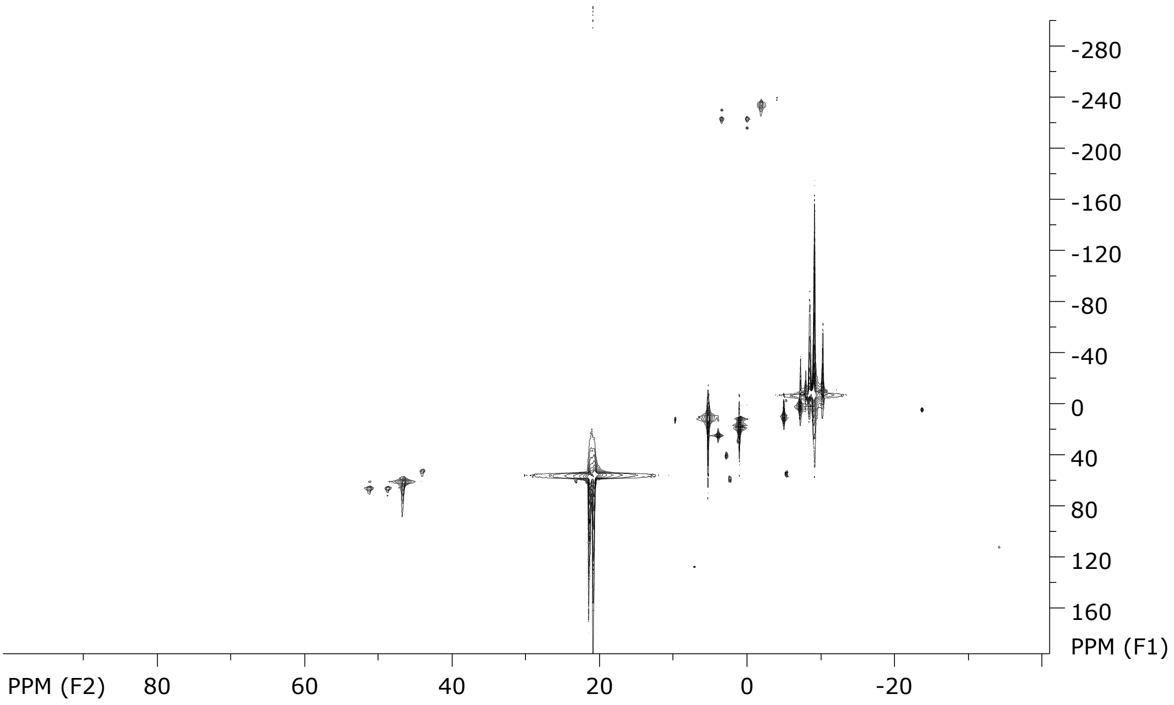

Figure S15. Gradient CH CORR spectrum (D_6-benzene, 298 K) of **4a**/**4b**.

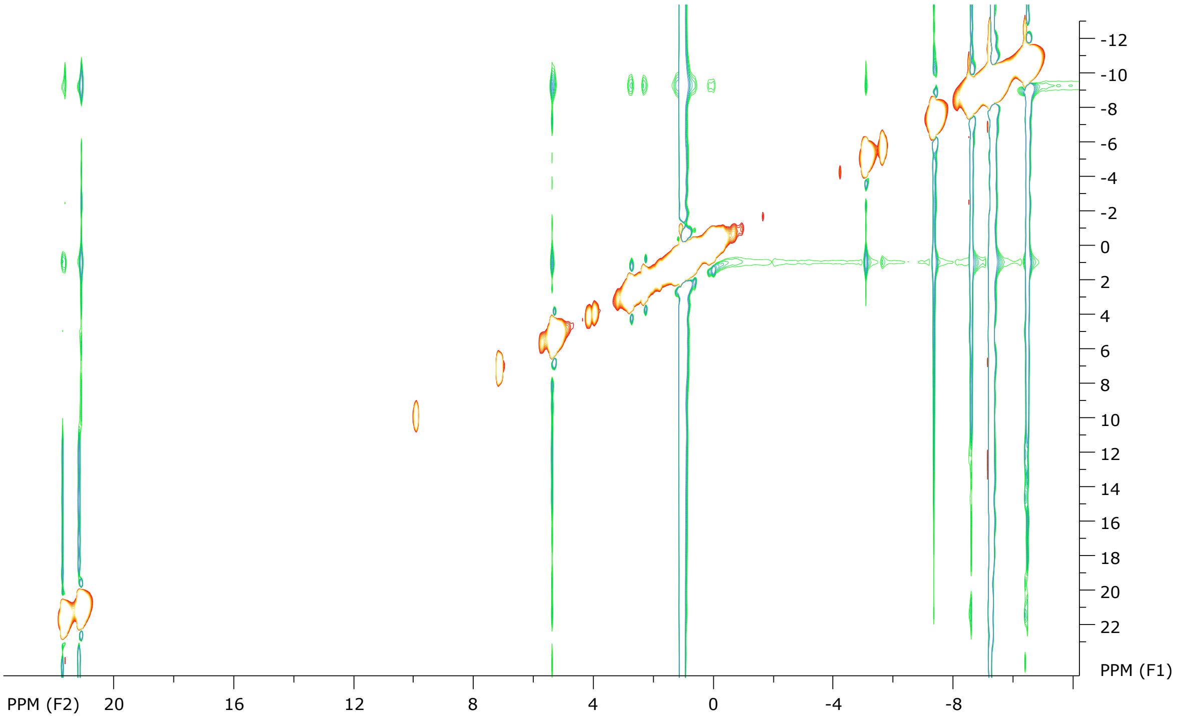

Figure S16. NOESY (D_6-benzene, 298 K) spectrum, phase sensitive, with 170 ms mixing time of **4a**/**4b**.

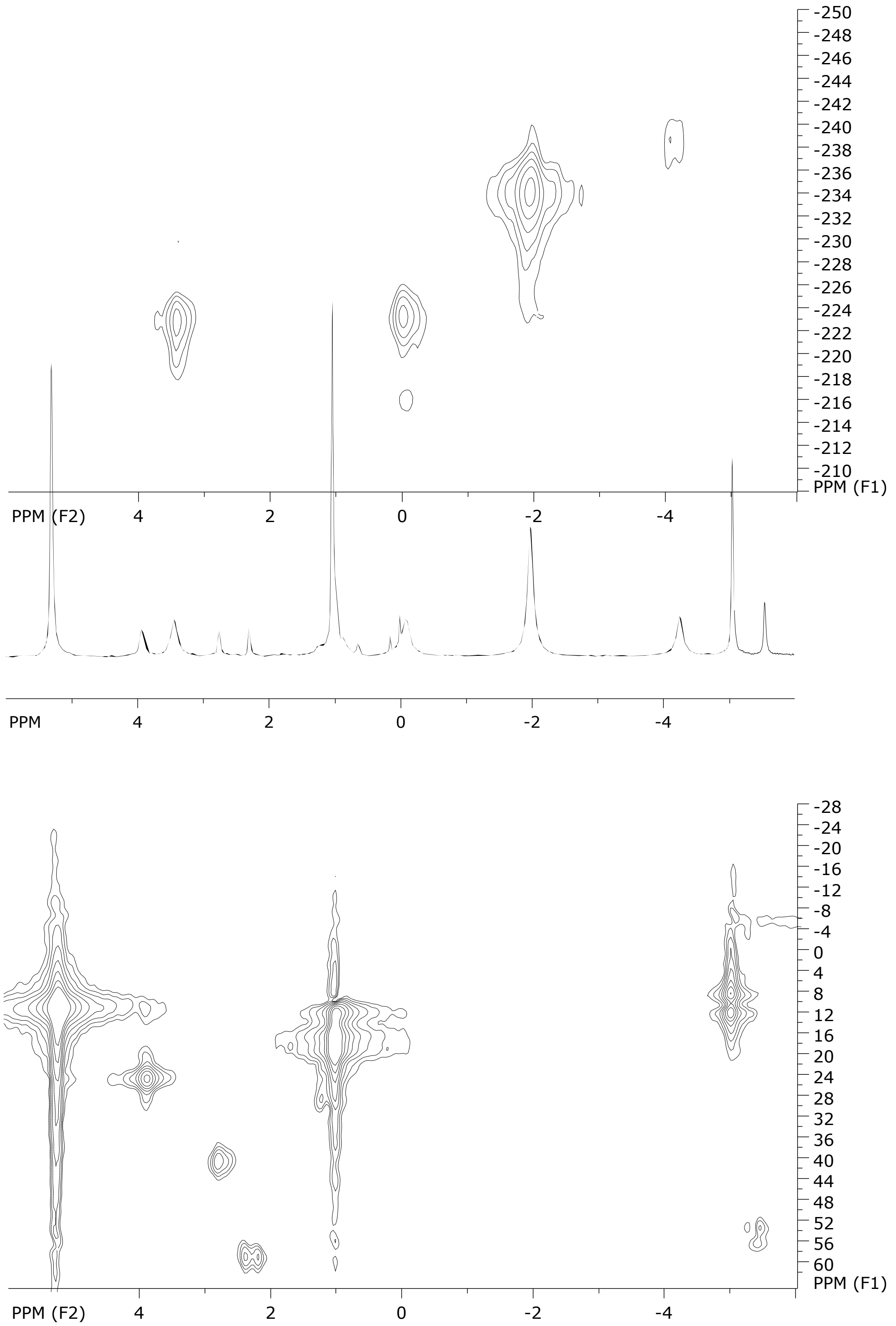

Figure S17. Parts of CH correlated (D_6-benzene, 298 K) spectrum of **4a**/**4b**, region from +6 to -6 ppm, with correlations at ^{13}C shift in the range of +30 ppm and -230 ppm.

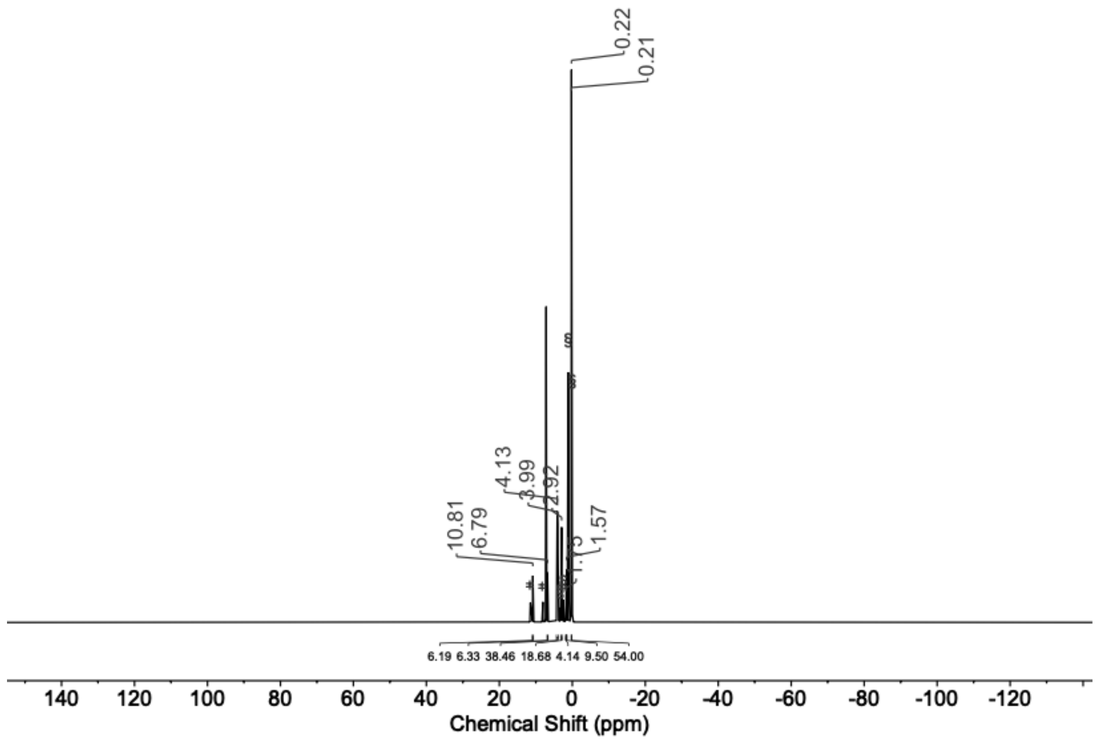

Figure S18. ^{1}H NMR spectrum of **7** in C_6D_6.

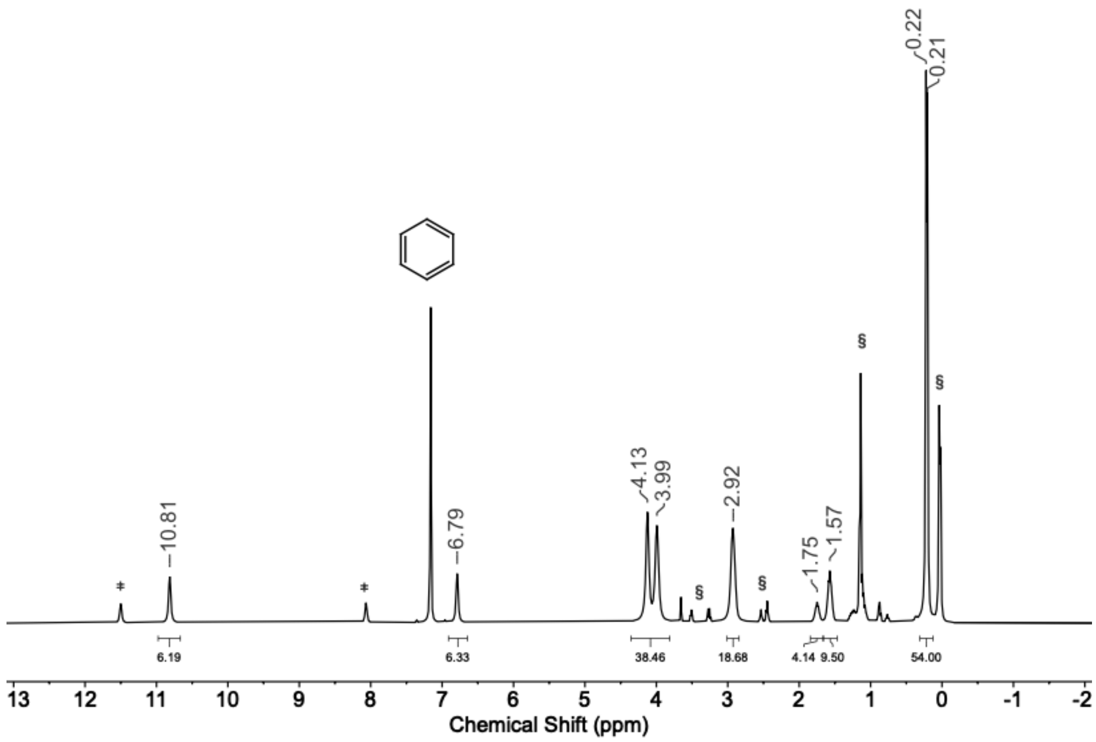

Figure S19. Zoom-in of the ^{1}H NMR (C_6D_6, 298K) spectrum of **7**. ‡ Symbol denotes an unknown impurity. § Denotes H_3Tren^{TIPS}.

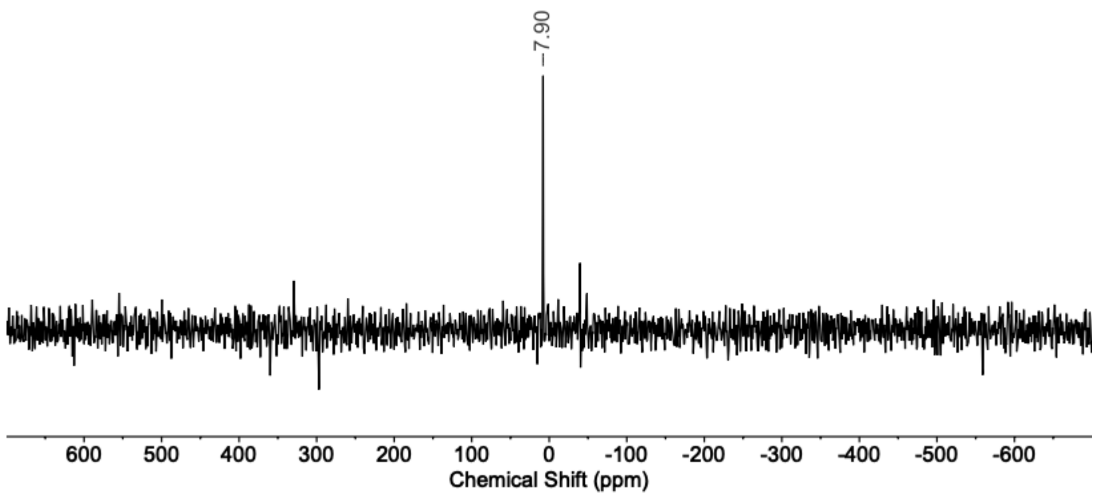

Figure S20. $^{29}Si\{^1H\}$ NMR (C_6D_6, 298 K) spectrum of **7**.

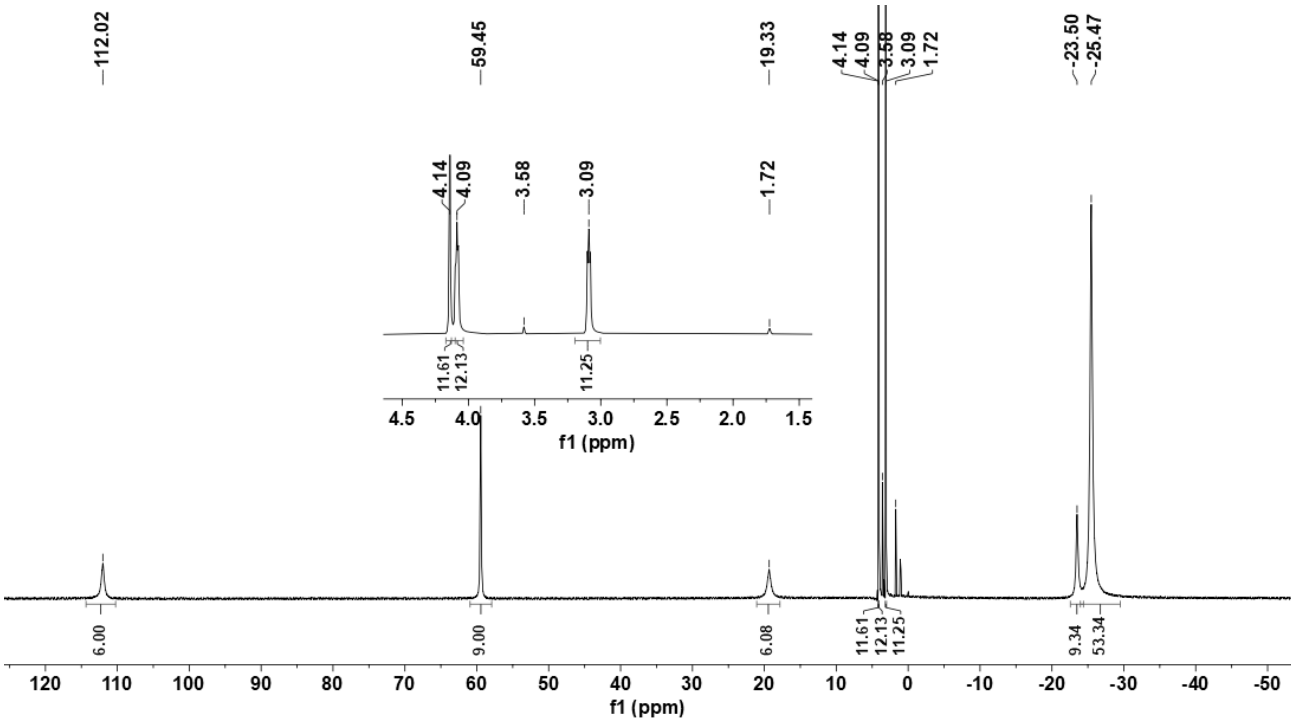

Figure S21. ^1H NMR (D_8-THF, 298 K) of **8UNSiMe$_3$**.

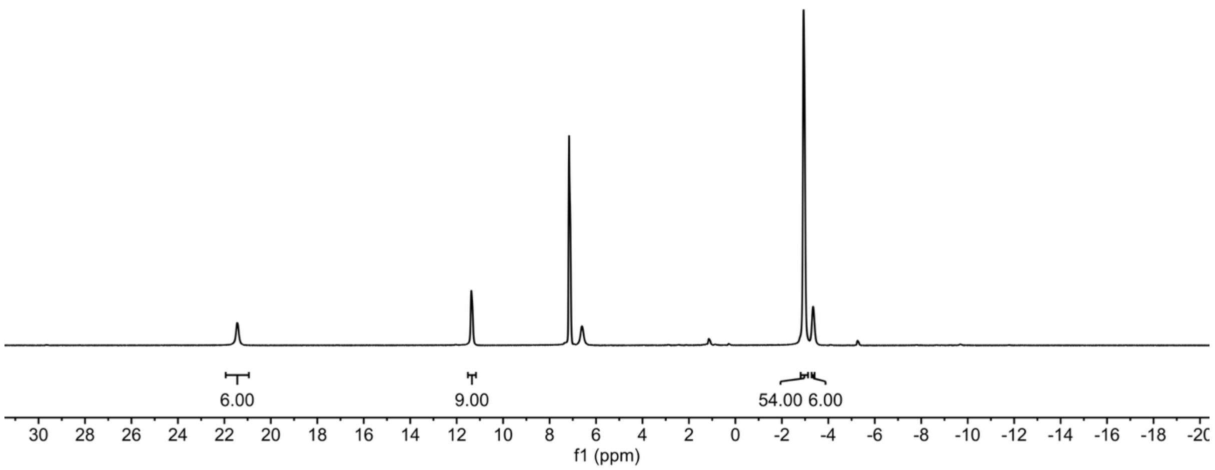

Figure 22. ^{1}H NMR (C_6D_6, 298 K) spectrum of the reaction mixture from the attempted oxidation of **3UNSiMe$_3$** with $AgBPh_4$.

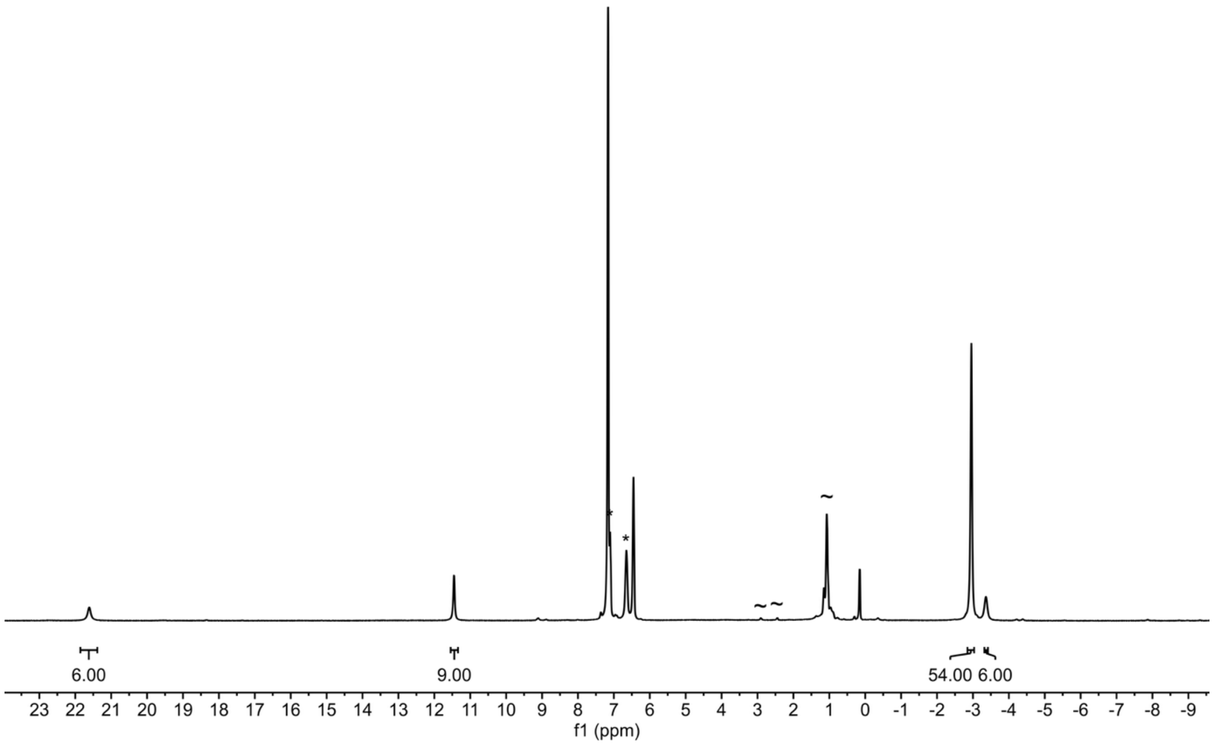

Figure 23. ^{1}H NMR (C_6D_6, 298 K) spectrum of the reaction mixture from the attempted oxidation of **3UNSiMe$_3$** with $[N(C_6H_4Br)_3][BArF_{20}]$. The asterisks (*) at ~7.10 and 6.65 ppm denotes $N(C_6H_4Br)_3$. ~ = H_3Tren^{TIPS} suggesting some decomposition.

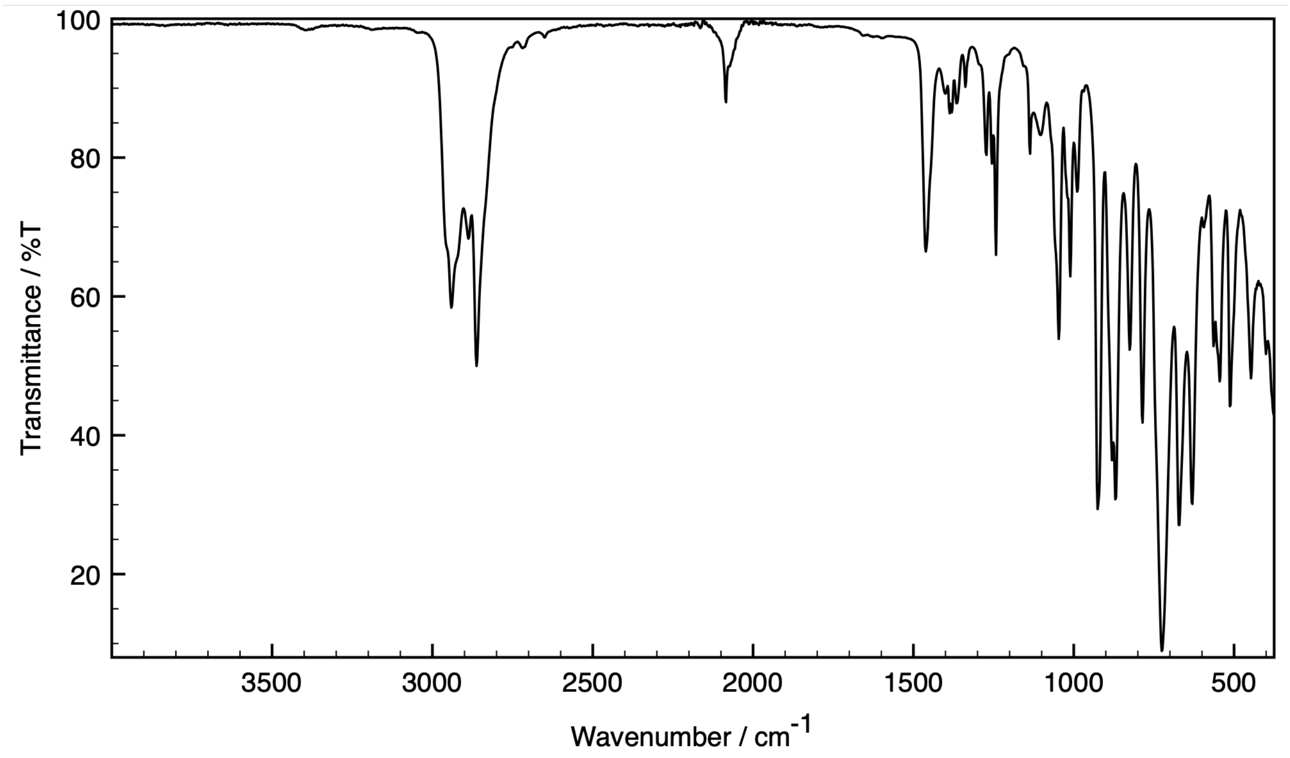

Figure S24. ATR-IR spectrum of **4a**/**4b**.

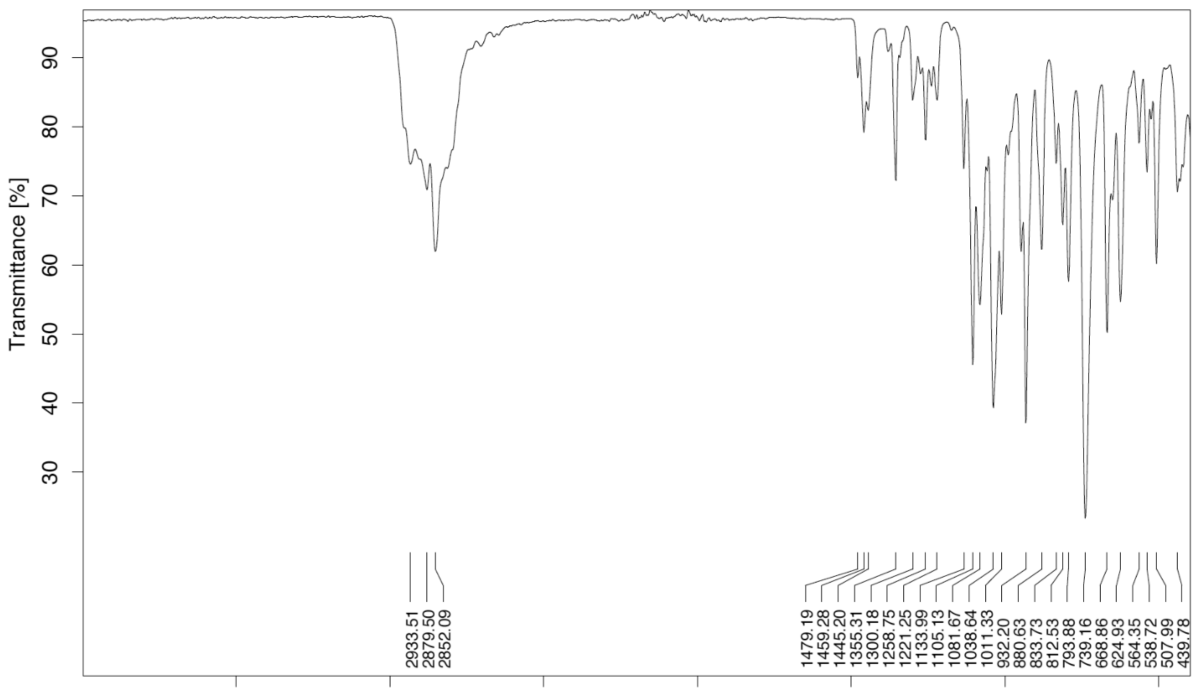

Figure S25. ATR-IR spectrum of **8UNSiMe$_3$**.

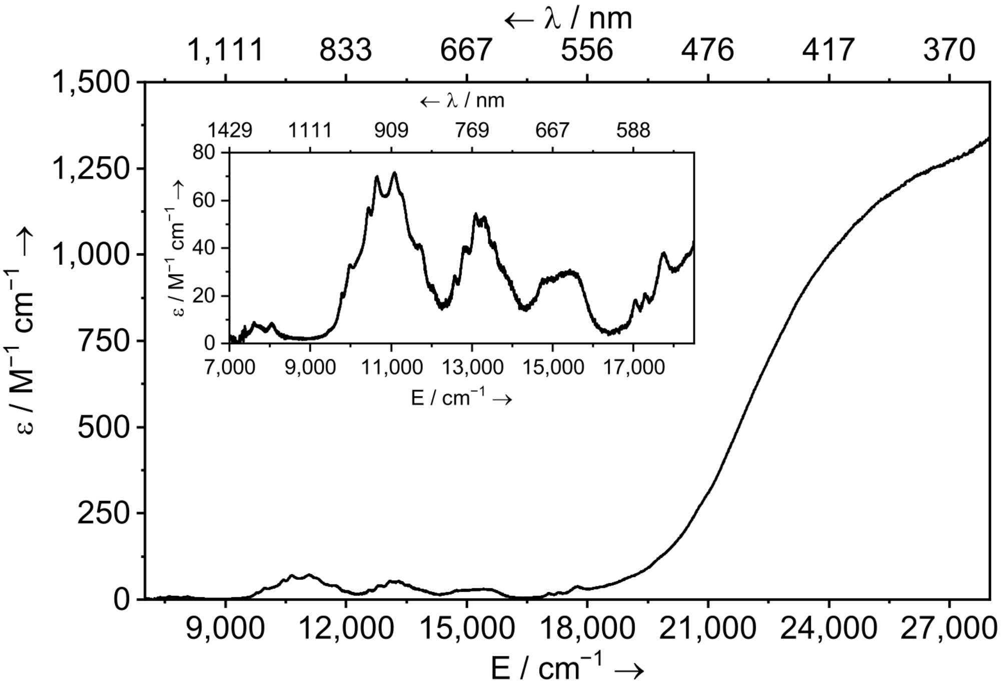

Figure S26. UV/Vis/NIR spectrum of a 7.87 mM solution of **4a**/**4b** in *n*-hexane.

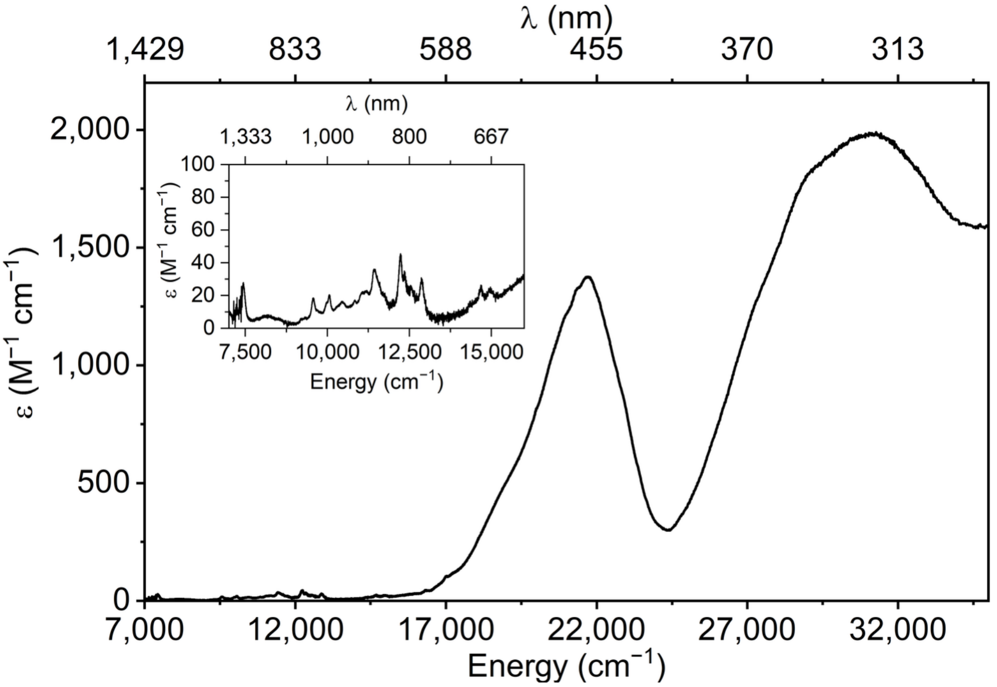

Figure S27. UV/Vis/NIR spectrum of a 0.5 mM solution of **7** in toluene.

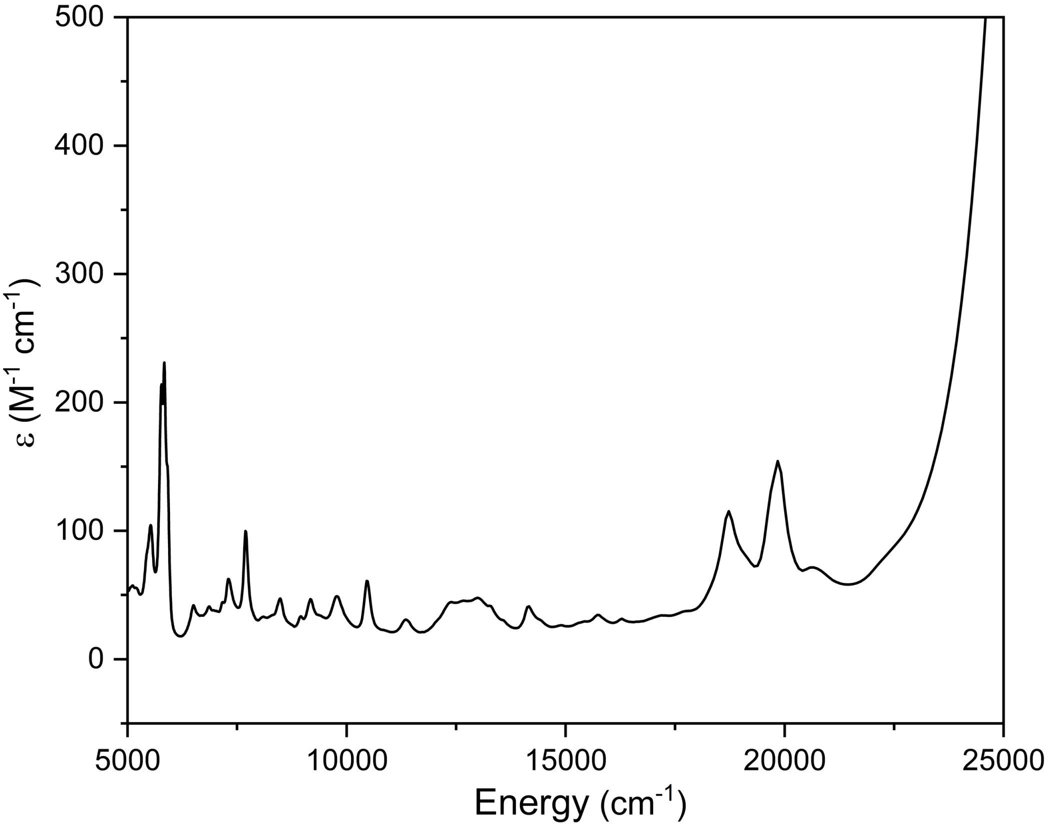

Figure S28. UV/Vis/NIR spectrum of a 25 mM solution of **8UNSiMe$_3$** in THF.

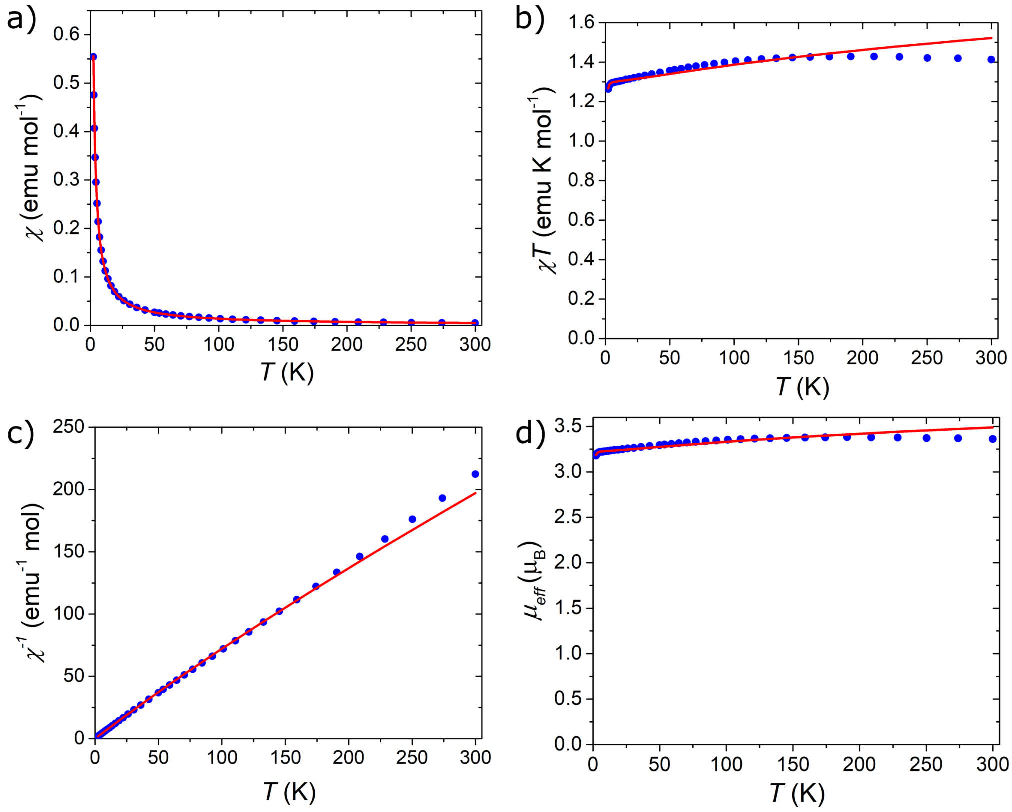

Figure S29. Variable-temperature SQUID magnetometry of **4a**/**4b** over the temperature range 1.8-300 K: a) χ vs T; b) χT vs T; c) 1/χ vs T d) μ_{eff} vs T. Lines represent the CONDON modelling.

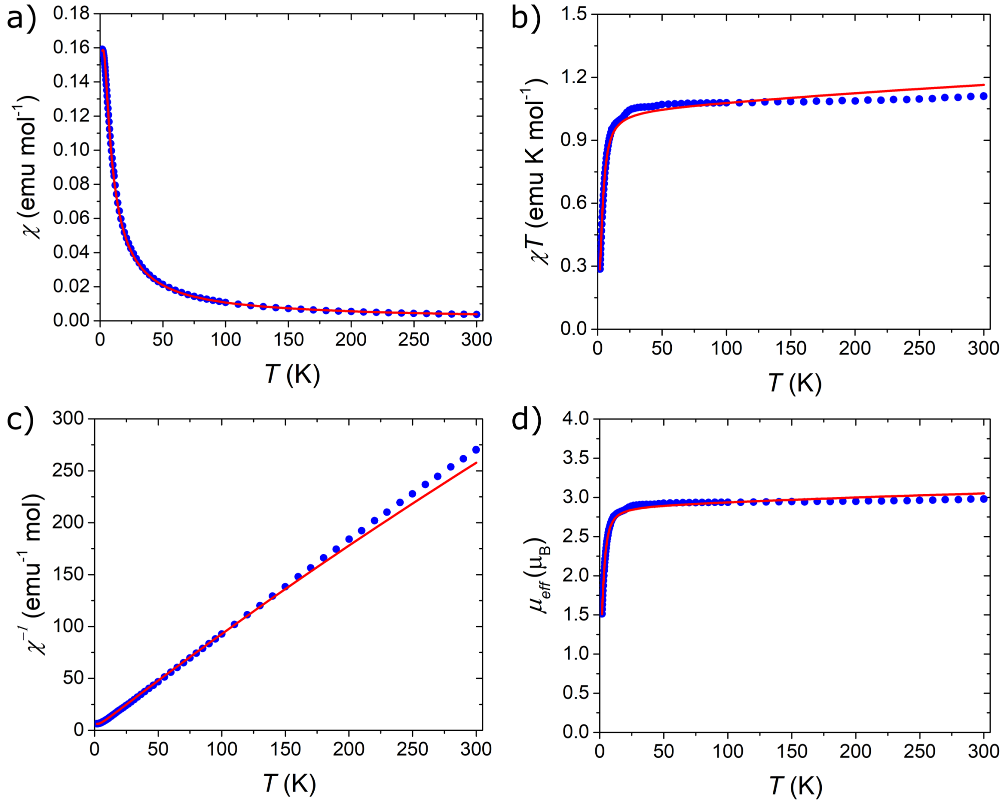

Figure S30. Variable-temperature SQUID magnetometry of **8UNSiMe$_3$** over the temperature range 1.8-300 K: a) χ vs T; b) χT vs T; c) 1/χ vs T d) μ_{eff} vs T. Lines represent the CONDON modelling.

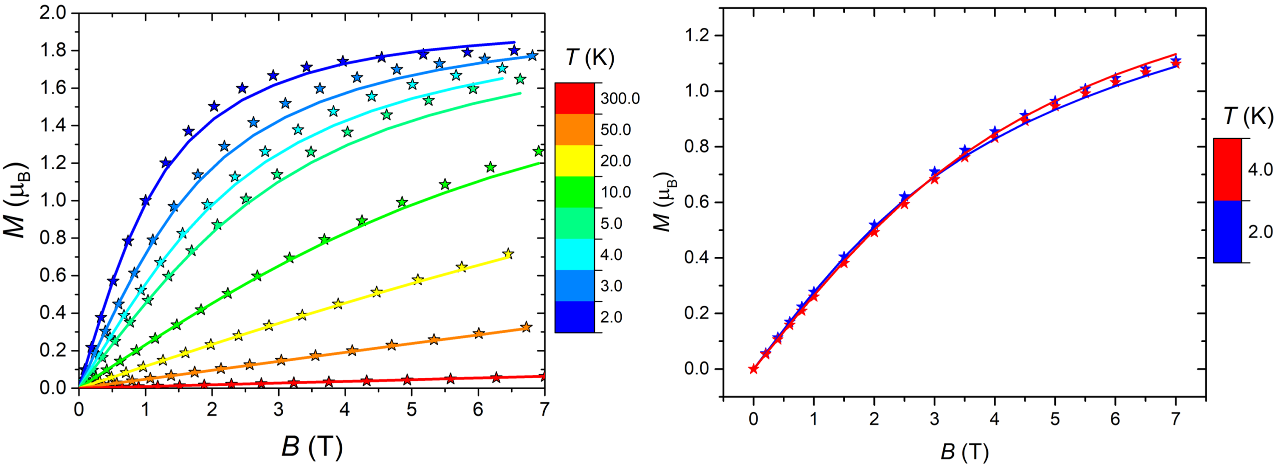

Figure S31. Magnetization vs Field data for **4a**/**4b** at 2, 3, 4, 5, 10, 20, 50, and 300 K (left) and for **8UNSiMe$_3$** at 2 and 4 K (right). Lines represent the CONDON modelling.

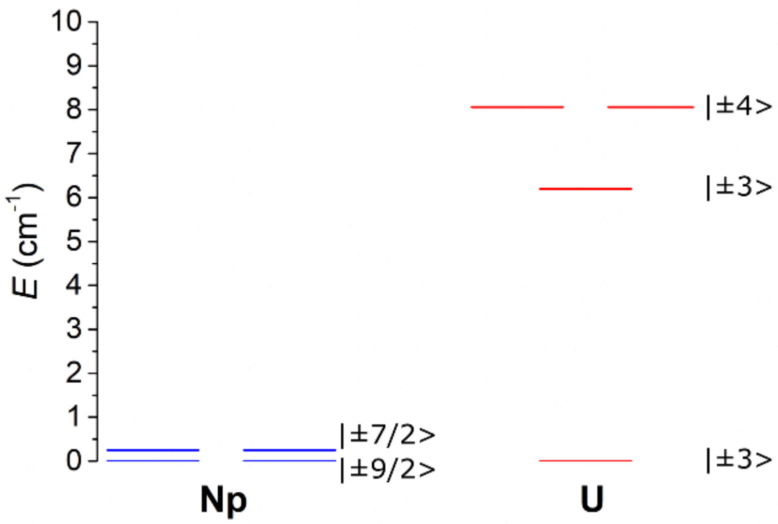

Figure S32. Zoomed-in energy level diagrams calculated from full Hamiltonian modelling for the $^4I_{9/2}$ ($5f^3$) and $^3H_4$ ($5f^2$) states of **4a** and **8UNSiMe$_3$**, respectively, demonstrating the pseudo-quartet ground state for both complexes. The $|\pm m_J>$ notation refers to the principal m_J component of each level.

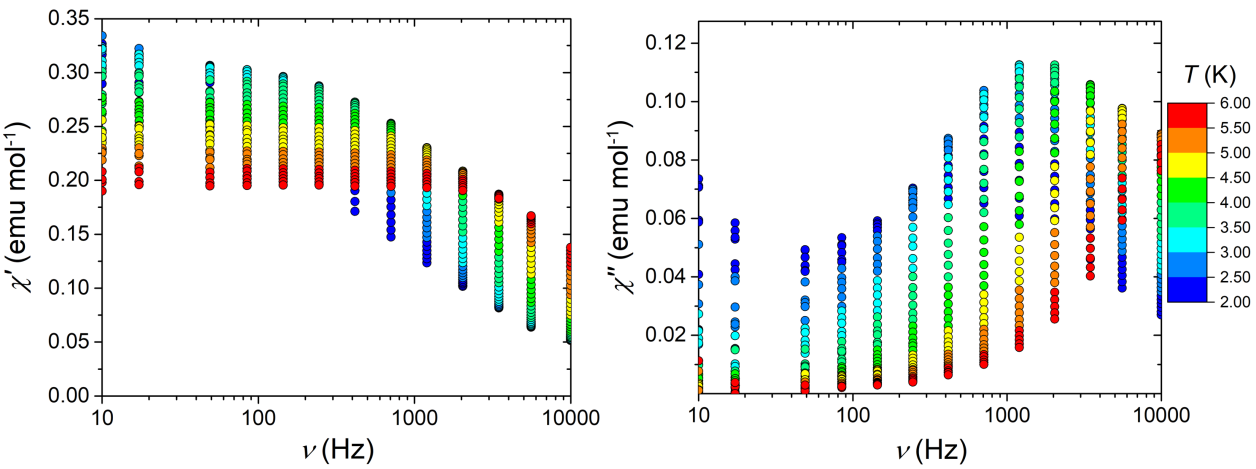

Figure S33. Frequency dependence of the real (left) and imaginary (right) components of the magnetic susceptibility for **4a**/**4b** as a function of temperature in an applied magnetic field of 4600 G.

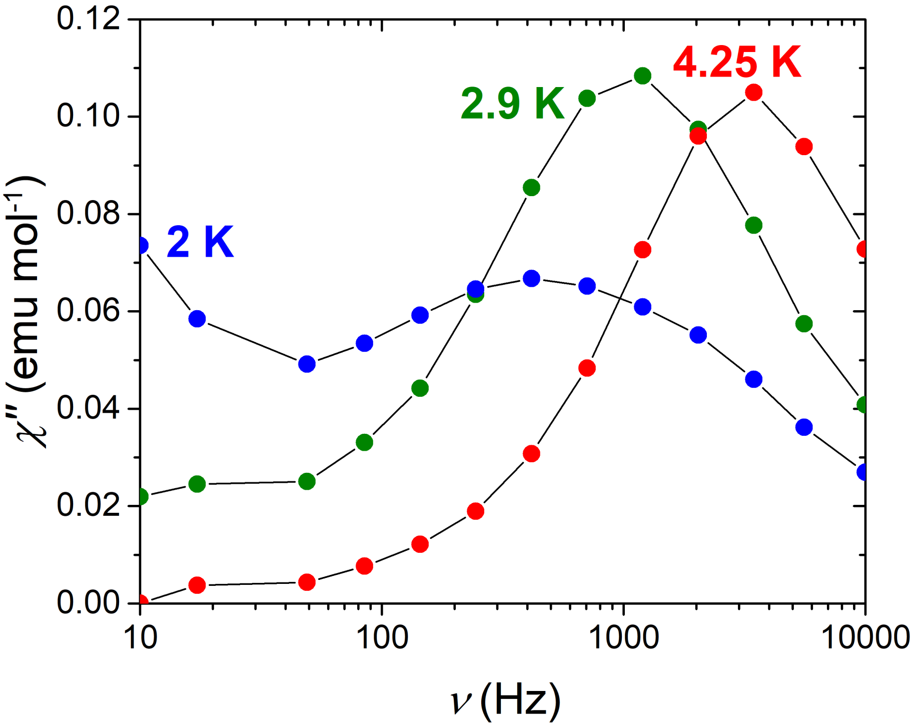

Figure S34. Selected χ" curves of **4a**/**4b** at different experimental temperatures with an applied magnetic field of 4600 Oe. At 2K two similar relaxation processes (two peaks) are evidently present, consistent with the 1:1 mix of co-crystallized **4a**/**4b**. The low frequency one rapidly disappears when the temperature is increased.

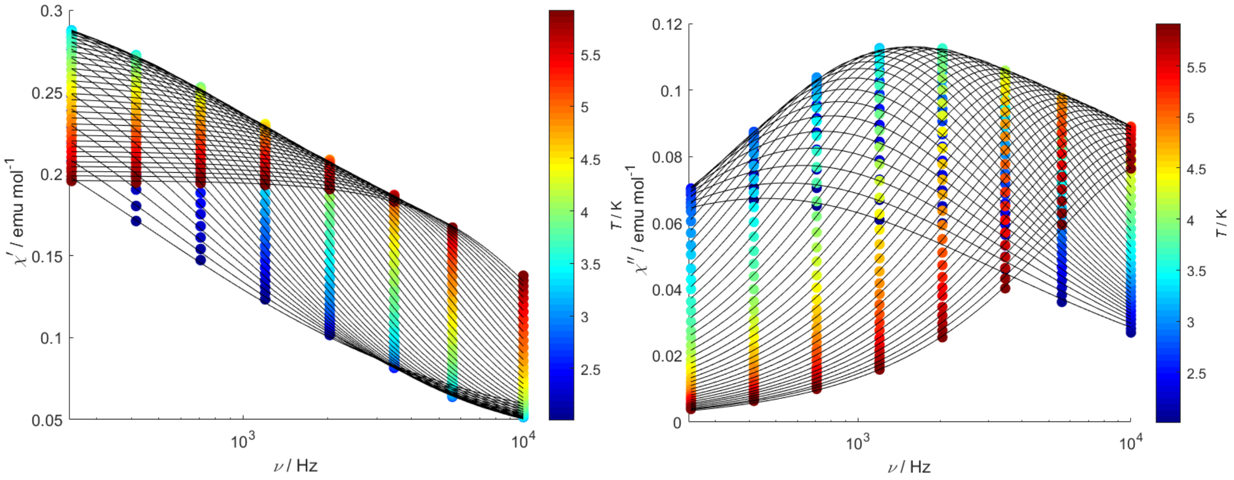

Figure S35. High frequency relaxation process at 4600 Oe of **4a**/**4b** fitted with a single component Debye model. Lines represent the best fit curves.

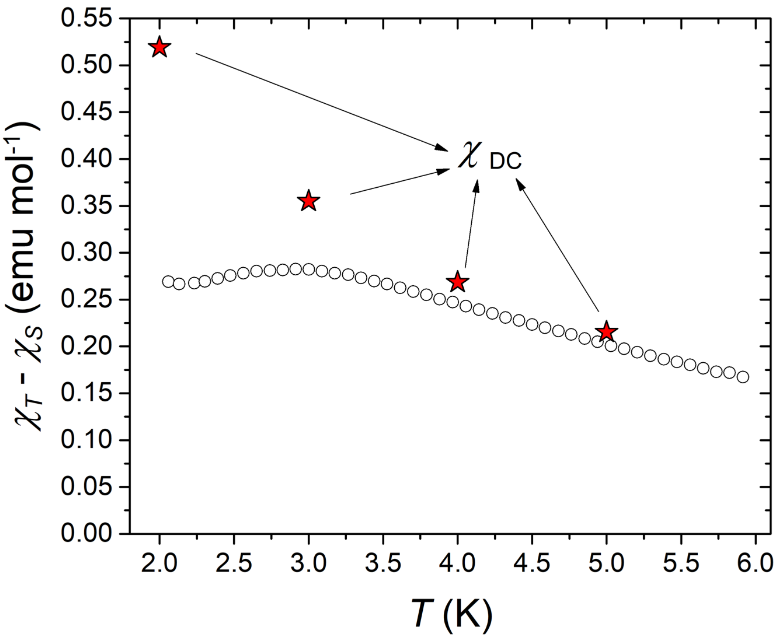

Figure S36. Comparison between the value of χ for **4a**/**4b** determined by DC (red stars) and AC (circles) magnetometry. The presence of two relaxation pathways at temperatures near 2 K is responsible for the difference observed between the two measurement techniques. Upon increasing the temperature, only one relaxation process remains, and the two measurement techniques give superimposable data values.

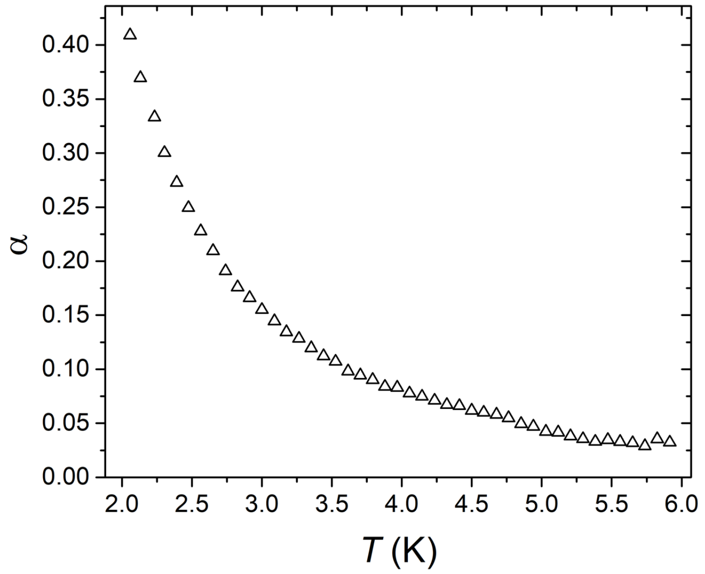

Figure S37. Temperature dependence of the parameter α of the Debye generalized model obtained through fitting of experimental AC magnetometry data on **4a**/**4b**. The resulting trend suggests a predominantly QTM process at low temperatures, which is replaced by a thermally activated process as soon as the temperature is increased.

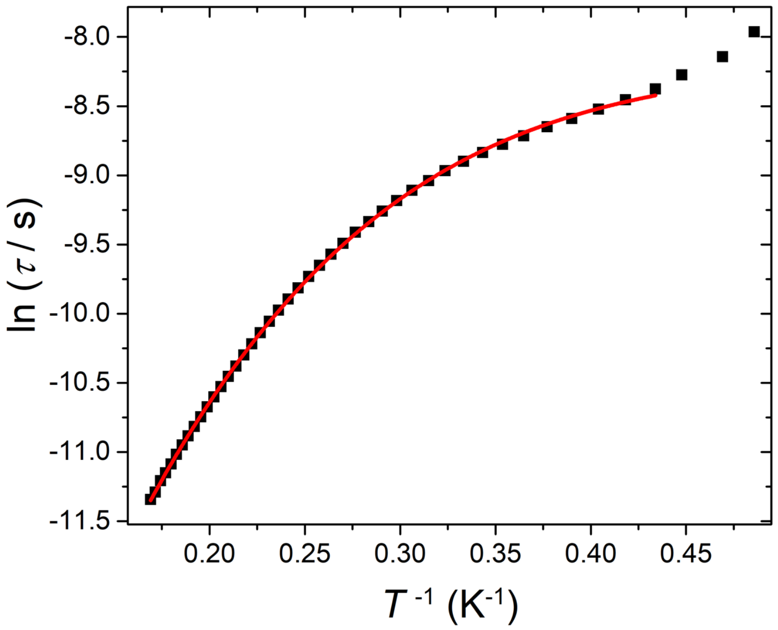

Figure S38. Thermal dependence of ln τ determined from the fit of real and imaginary components of magnetic susceptibility of **4a**/**4b** at 4600 Oe. The red line represents the best fit curve with parameters reported in Table S3.

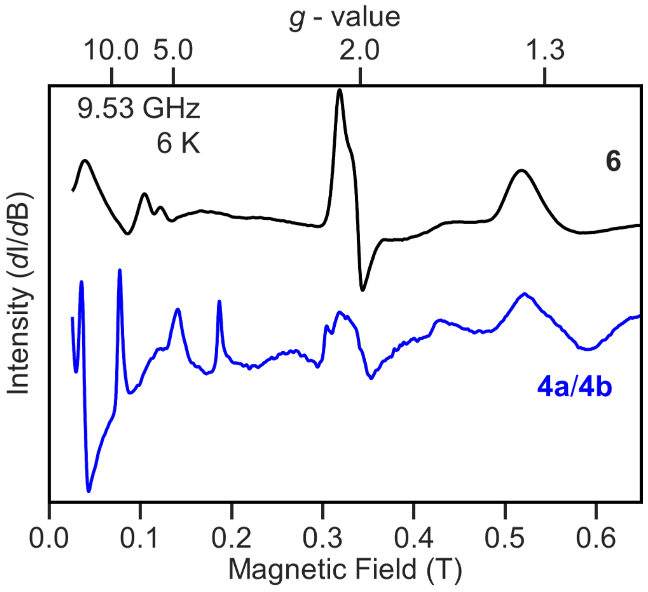

Figure S39. X-band (9.53 GHz) EPR spectra of frozen toluene solutions of **6** (black line) and **4a**/**4b** (blue line) at 6 K.

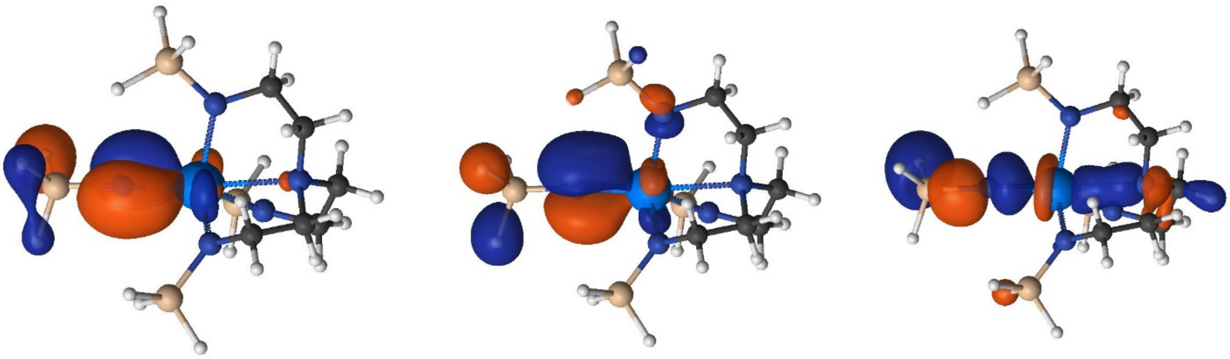

Figure S40. CASSCF-computed Np-N_{imido} π- and σ-bond combinations of the truncated model [Np{N($CH_2CH_2NSiH_3$}($NSiH_3$)] with a (12,16) active space.

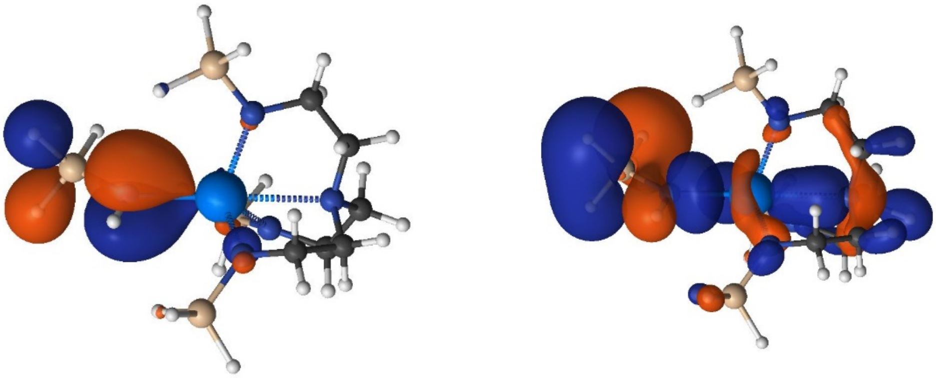

Figure S41. CASSCF-computed Np-N_{imido} π- and σ-bond combinations of the truncated model [Np{N($CH_2CH_2NSiH_3$}($NHSiH_3$)] with a (13,16) active space.

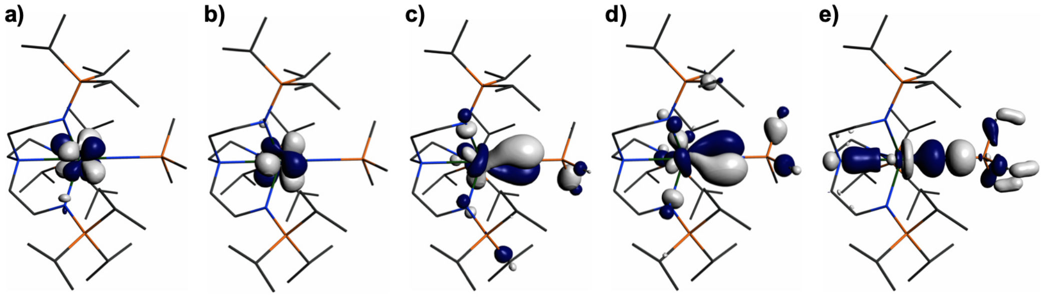

Figure S42. Selected frontier molecular orbitals of putative **3NpNSiMe$_3$**. a) HOMO (243a, −3.749 eV), b) HOMO−1 (242a, −3.754 eV), c) HOMO−5 (238a, −5.454 eV), d) HOMO−6 (237a, −5.462 eV), e) HOMO−19 (224a, −7.302 eV). Hydrogen atoms are omitted for clarity.

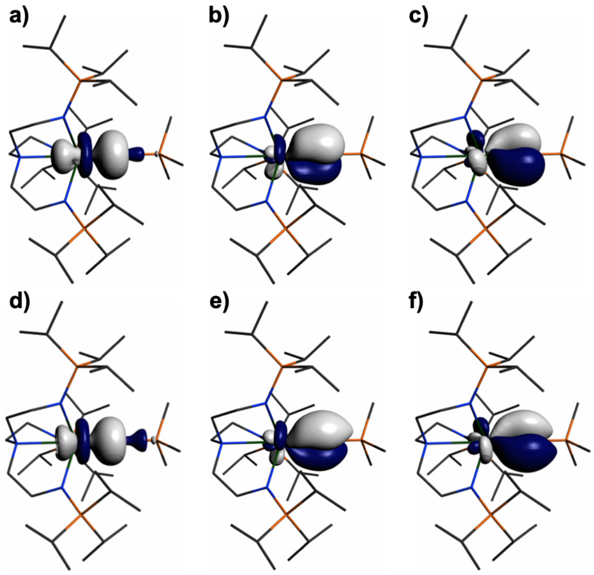

Figure S43. Selected NBOs and NLMOs of putative **3NpNSiMe$_3$**. Hydrogen atoms are omitted for clarity.

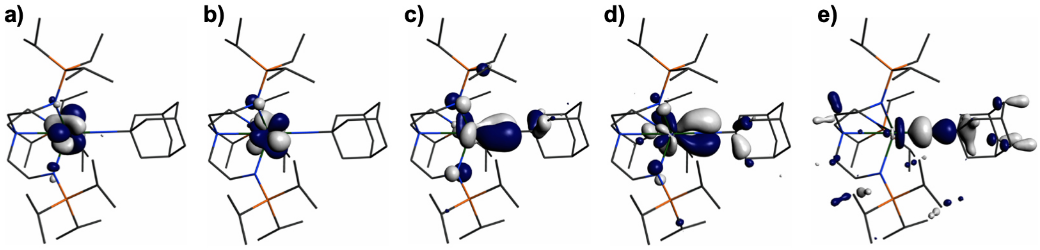

Figure S44. Selected frontier molecular orbitals of putative **3NpNAd**. a) HOMO (260a, −4.271 eV), b) HOMO−1 (259a, −4.289 eV), c) HOMO−5 (255a, −5.644 eV), d) HOMO−6 (254a, −5.668 eV), e) HOMO−50 (210a, −9.162 eV). Hydrogen atoms are omitted for clarity.

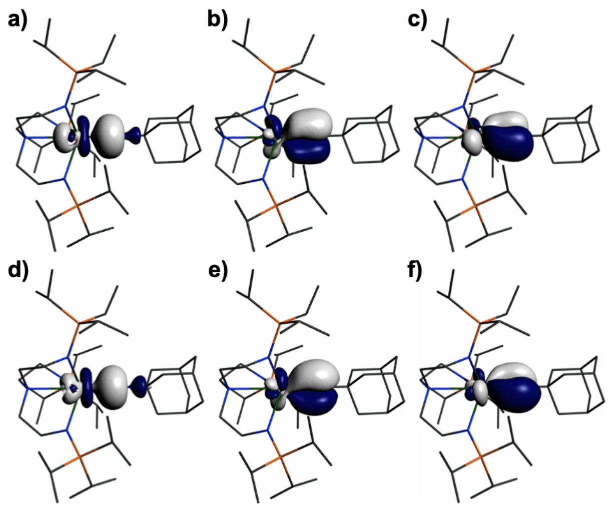

Figure S45. Selected NBOs and NLMOs of putative **3NpNAd**. Hydrogen atoms are omitted for clarity.

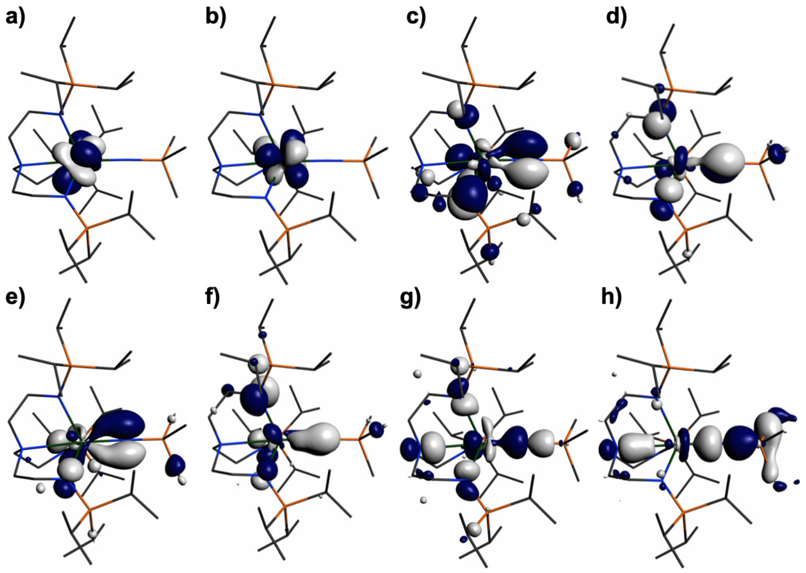

Figure S46. Selected frontier molecular orbitals of the anion of **8UNSiMe$_3$**. a) HOMO (243a, 0.708 eV), b) HOMO−1 (242a, 0.638 eV), c) HOMO−2 (241a, −1.696 eV), d) HOMO−3 (240a, −1.728 eV), e) HOMO−5 (238a, −2.069 eV), f) HOMO−6 (237a, −2.148 eV), g) HOMO−7 (236a, −2.963 eV), h) HOMO−19 (224a, −4.095 eV). Hydrogen atoms are omitted for clarity.

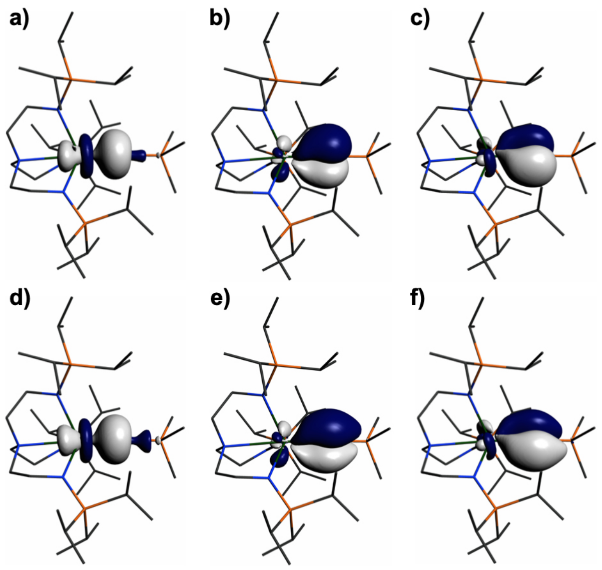

Figure S47. Selected NBOs and NLMOs of the anion of **8UNSiMe**$_3$. Hydrogen atoms are omitted for clarity.

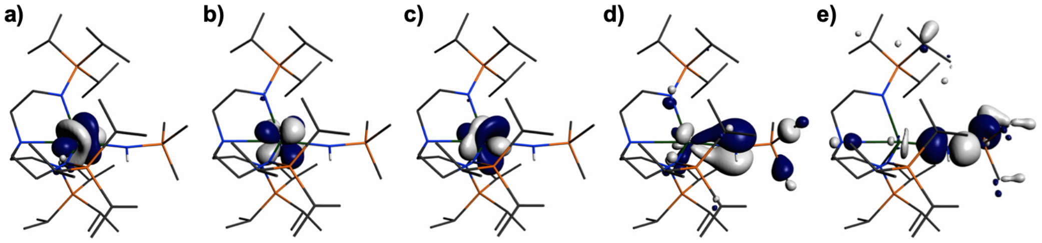

Figure S48. Selected frontier molecular orbitals of **4a**. a) HOMO (244a, −3.445 eV), b) HOMO−1 (243a, −3.495 eV), c) HOMO−2 (242a, −3.520 eV), d) HOMO−6 (238a, −5.290 eV), e) HOMO−19 (225a, −7.383 eV). Hydrogen atoms, except the N-H proton, are omitted for clarity.

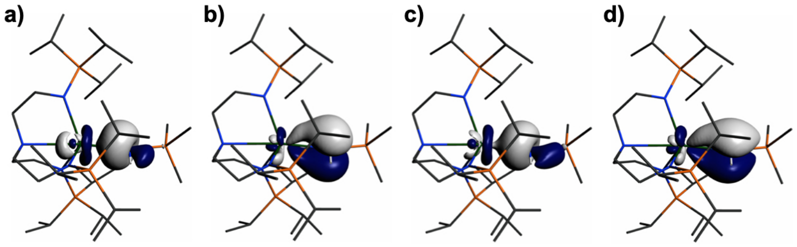

Figure S49. Selected NBOs and NLMOs of **4a**. Hydrogen atoms, except the N-H proton, are omitted for clarity.

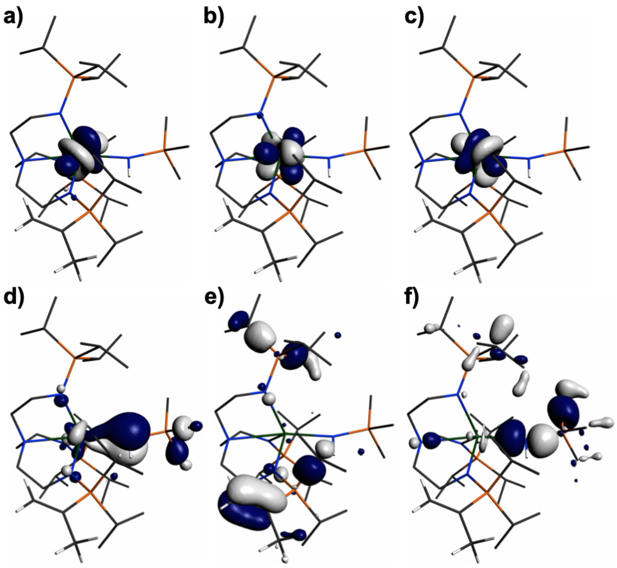

Figure S50. Selected frontier molecular orbitals of **4b**. a) HOMO (243a, −3.481 eV), b) HOMO−1 (242a, −3.530 eV), c) HOMO−2 (241a, −3.566 eV), d) HOMO−6 (237a, −5.336 eV), e) HOMO−9 (234a, −5.868 eV), f) HOMO−20 (223a, −7.417 eV). Hydrogen atoms, except the N-H proton and MeC=CH_2 group, are omitted for clarity.

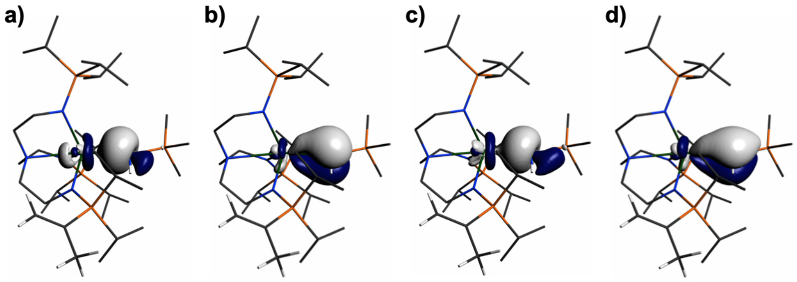

Figure S51. Selected NBOs and NLMOs of **4b**. Hydrogen atoms, except the N-H proton and MeC=CH_2 group, are omitted for clarity.

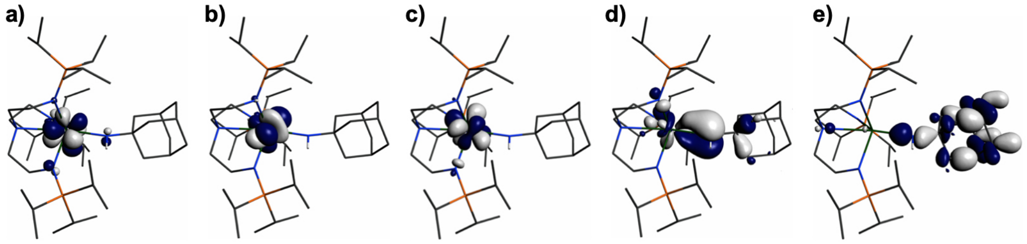

Figure S52. Selected frontier molecular orbitals of **5a**. a) HOMO (261a, –4.054 eV), b) HOMO–1 (260a, –4.128 eV), c) HOMO–2 (259a, –4.191 eV), d) HOMO–6 (255a, –5.511 eV), e) HOMO–19 (242a, –7.316 eV). Hydrogen atoms, except the N-H proton, are omitted for clarity.

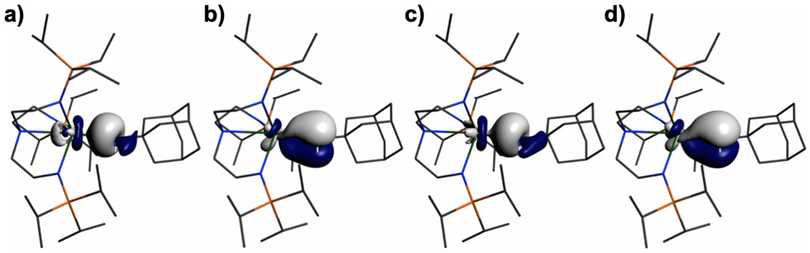

Figure S53. Selected NBOs and NLMOs of **5a**. Hydrogen atoms, except the N-H proton, are omitted for clarity.

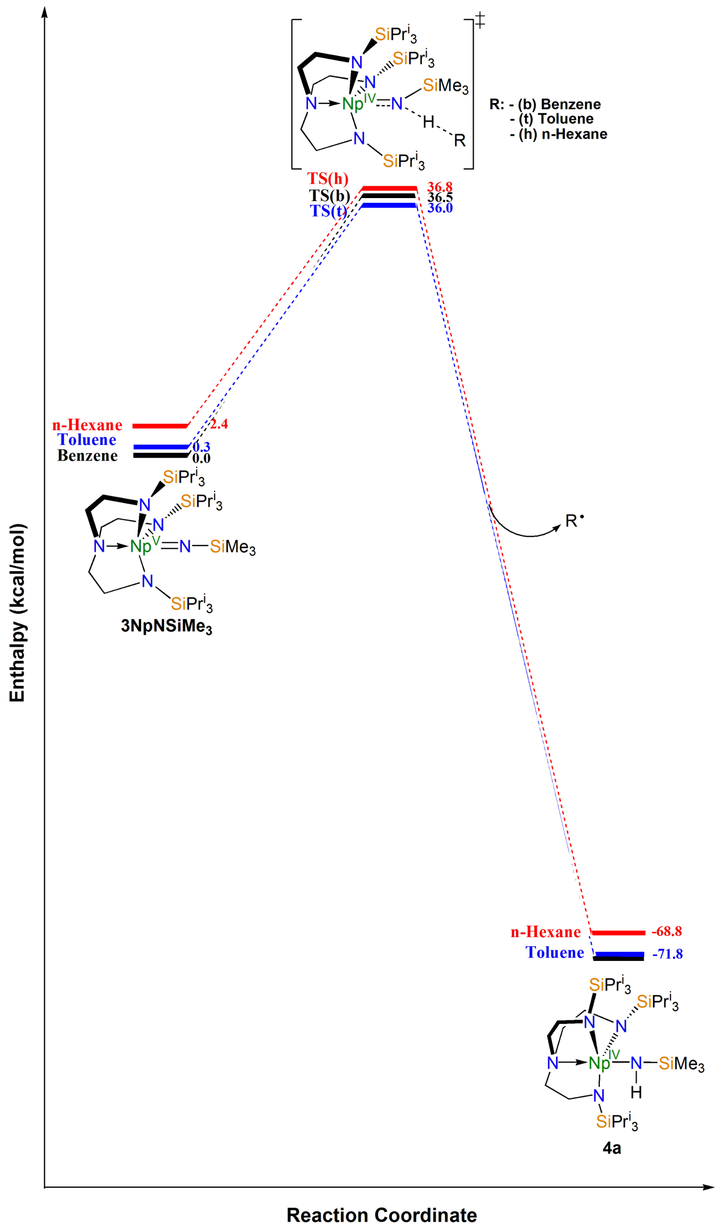

Figure S54. Computed reaction profile for the direct reaction of **3NpNSiMe₃** with the reaction solvents benzene, toluene, and hexane.

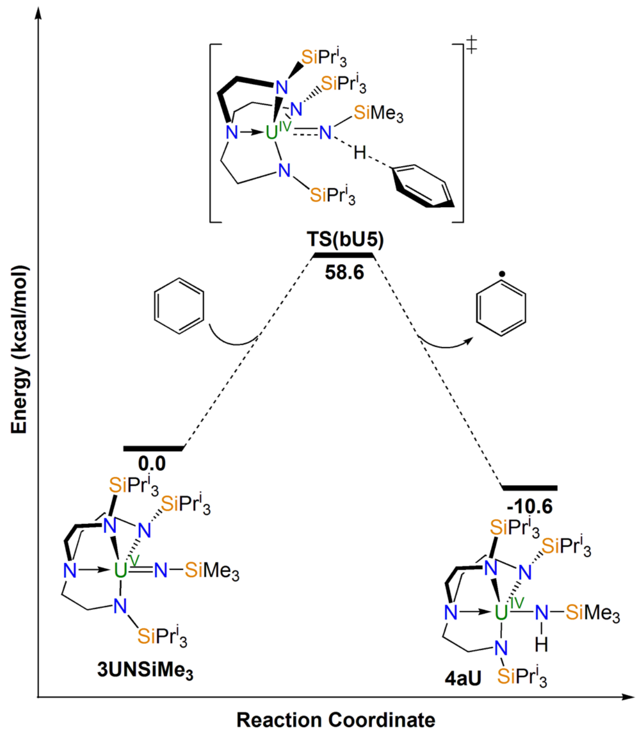

Figure S55. Computed reaction profile for the intermolecular reaction of **3UNSiMe3** with benzene.

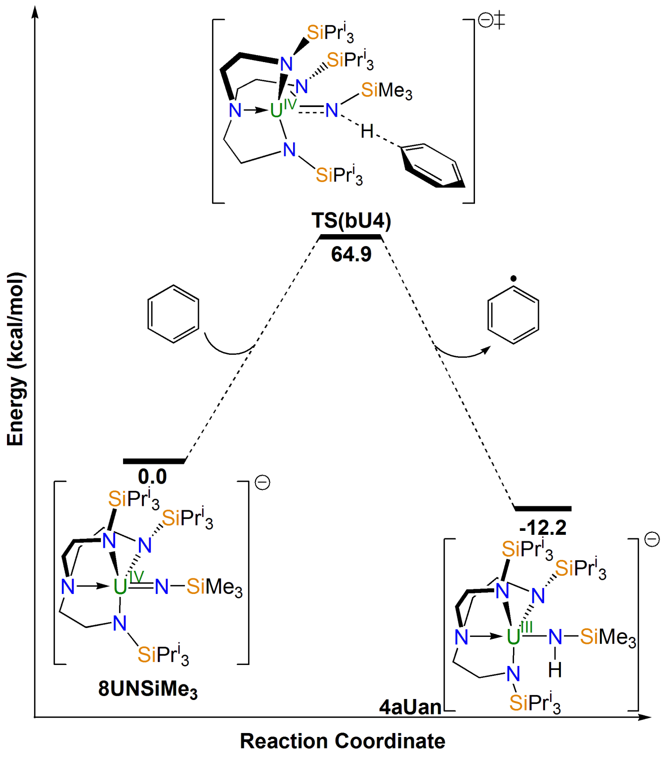

Figure S56. Computed reaction profile for the intermolecular reaction of **8UNSiMe3** with benzene.

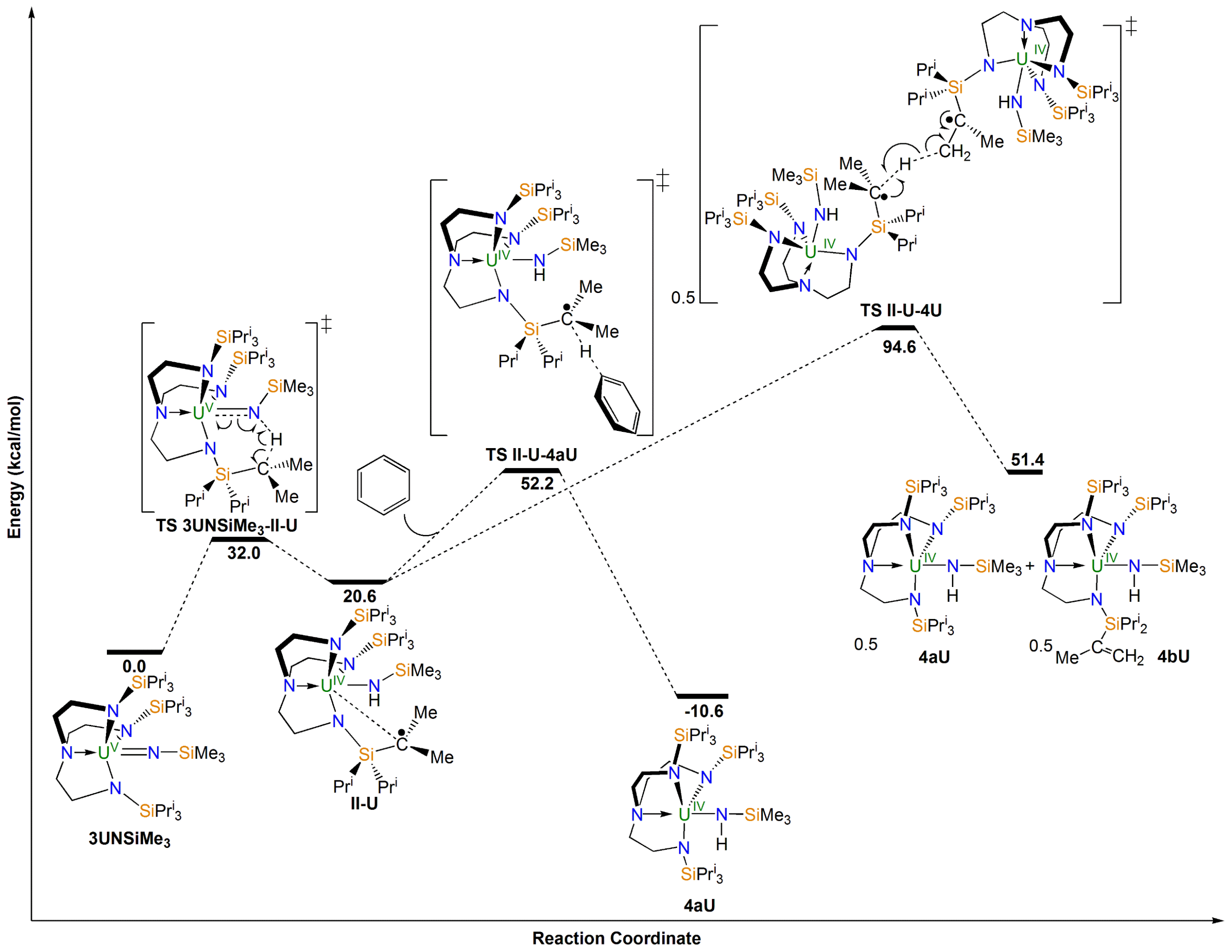

Figure S57. Computed reaction profile for the intramolecular-intermolecular reactions of **3UNSiMe₃**.

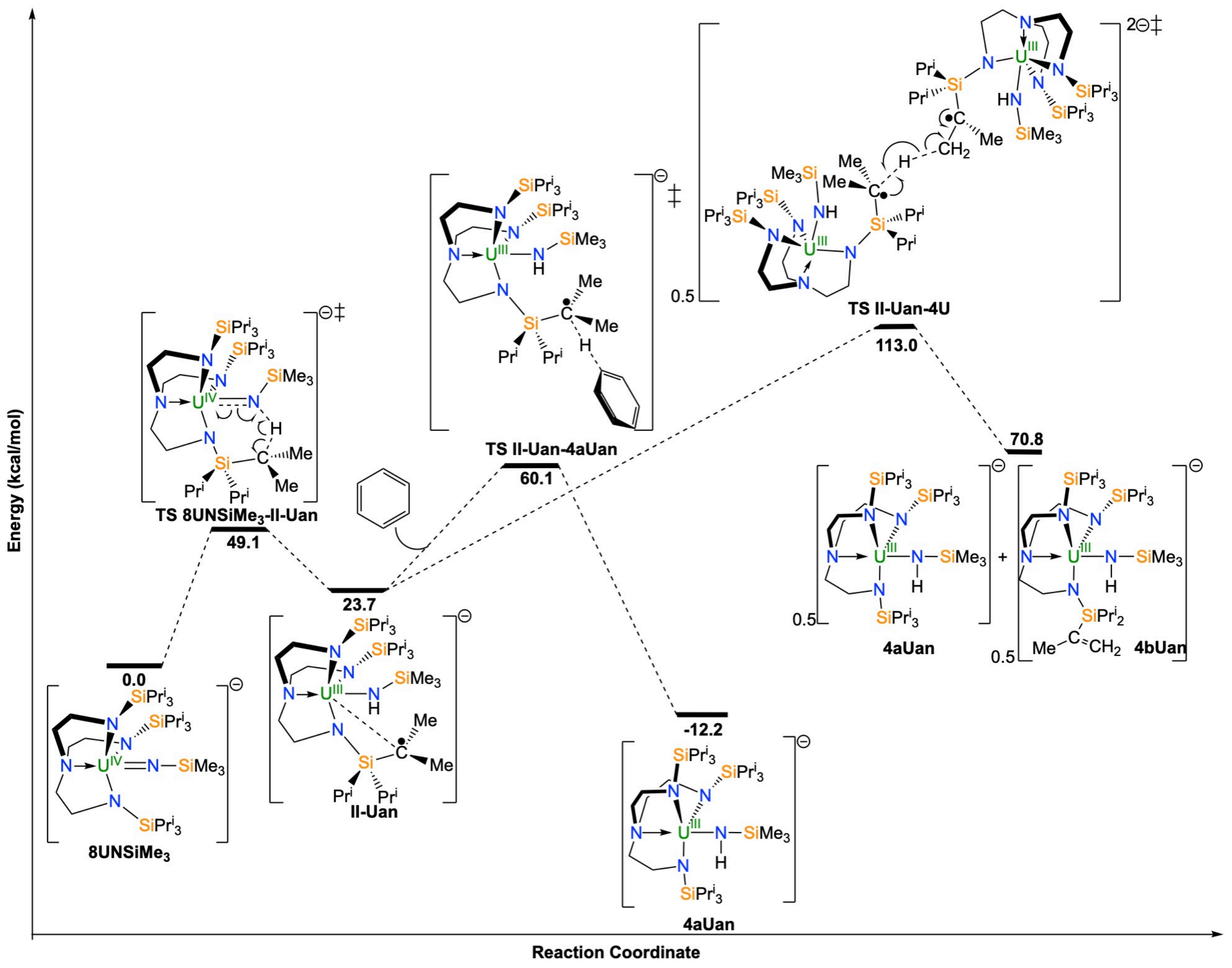

Figure S58. Computed reaction profile for the intramolecular-intermolecular reactions of **8UNSiMe₃**.

Tables

Table S1. Crystal field parameters (B_k^q) and orbital reduction factor (κ) determined by the CONDON analysis of the DC data of 4a and 8UNSiMe₃.

Complex	$B_2^0\ (cm^{-1})$	$B_4^0\ (cm^{-1})$	$B_6^0\ (cm^{-1})$	$B_4^3\ (cm^{-1})$	κ
**4a**	8398	6726	-6488	2246	1
**8UNSiMe$_3$**	4664	-2932	3378	536	0.93

Table S2. Slater-Condon integrals (F_2, F_4, F_6) and spin-orbit coupling (ζ) parameters used for the modelling of the magnetic data of 4a and 8UNSiMe$_3$.

Complex	$F^2(cm^{-1})$	$F^4(cm^{-1})$	$F^6(cm^{-1})$	$\zeta(cm^{-1})$
**4a**	45212	38018	28345	2129
**8UNSiMe$_3$**	42600	36500	29000	1740

Table S3. Best fit parameters of the thermal evolution of the magnetic relaxation dynamics of 4a at 4600 Oe.

$\tau_{QTM}\ (s)$	$C\ (K^{-n})$	n
$3.01(4) \cdot 10^{-4}$	32(2)	4.40(4)

Table S4. Composition of the spin-orbit-free SF-CASSCF ground states of the complexes

Complex	State	Active space	Main configuration	Main 5f of 1e orbitals
**3NpNSiMe$_3$**	^{3}H	(2,7)	64+36 %	$5f_\sigma$, $5f_\delta$
[Np{N(CH$_2$CH$_2$NSiH$_3$}(NSiH$_3$)]		(2,7)	82+18 %	$5f_\sigma$, $5f_\delta$
		(12,16)	76+17 %	$5f_\sigma$, $5f_\delta$
**4a**	^{4}I	(3,7)	88 %	strongly mixed
[Np{N(CH$_2$CH$_2$NSiH$_3$}(NHSiH$_3$)]		(3,7)	88 %	$5f_{\sigma\phi}$, $5f_\delta$, $5f_{\sigma\phi}$
		(13,16)	85 %	strongly mixed

Table S5. Spin-orbit characteristics of the SO-CASSCF ground states of the complexes

Complex	State	Active space	Composition (SF states)
[Np{N(CH$_2$CH$_2$NSiH$_3$}(NSiH$_3$)]	$^3H_4$	(2,7)	39% SF1 + 39% SF2
[Np{N(CH$_2$CH$_2$NSiH$_3$}(NHSiH$_3$)]	$^4I_{9/2}$	(3,7)	35% SF2 + 31% SF1

Table S6. Bonding characteristics from SF-CASSCF and SF-CASPT2 wavefunctions of the Np(V) complexes

Bond	Property	3NpNSiMe$_3$ (2,7)	[Np{N(CH$_2$CH$_2$NSiH$_3$}(NSiH$_3$)] (2,7)		[Np{N(CH$_2$CH$_2$NSiH$_3$}(NSiH$_3$)] (12,16)	
		**CASSCF**	**CASSCF**	**CASPT2**	**CASSCF**	**CASPT2**
Np	q_{Np}	3.14	3.09	3.00	3.02	2.95
Np=N	Wiberg	2.11	2.03	1.92	2.01	1.90
	DI	1.60	1.61	1.37	1.59	1.35
	ρ(BCP)	0.20	0.21	0.21	0.21	0.21
Np…N$_1$	Wiberg	0.84	0.82	0.79	0.90	0.89
	DI	0.61	0.65	0.59	0.64	0.62
	ρ(BCP)	0.10	0.11	0.11	0.11	0.11
Np…N$_2$	Wiberg	0.83	0.82	0.79	0.84	0.81
	DI	0.60	0.64	0.58	0.63	0.58
	ρ(BCP)	0.10	0.10	0.10	0.10	0.10
Np…N$_3$	Wiberg	0.84	0.81	0.78	0.86	0.85
	DI	0.60	0.64	0.58	0.63	0.61
	ρ(BCP)	0.10	0.10	0.10	0.10	0.11
Np…N$_4$	Wiberg	0.28	0.26	0.25	0.25	0.24
	DI	0.24	0.20	0.19	0.20	0.18
	ρ(BCP)	0.05	0.04	0.04	0.04	0.04

Table S7. Bonding characteristics from SF-CASSCF and SF-CASPT2 wavefunctions of the Np(IV) complexes

Bond	Property	4a (3,7)	[Np{N(CH$_2$CH$_2$NSiH$_3$}(NHSiH$_3$)] (2,7)		[Np{N(CH$_2$CH$_2$NSiH$_3$}(NHSiH$_3$)] (2,7)	
		**CASSCF**	**CASSCF**	**CASPT2**	**CASSCF**	**CASPT2**
Np	q_{Np}	2.88	2.86	2.82	2.81	2.78
Np-NH	Wiberg	0.89	0.82	0.80	0.81	0.79
	DI	0.63	0.62	0.57	0.61	0.57
	ρ(BCP)	0.10	0.10	0.10	0.10	0.10
Np…N$_1$	Wiberg	0.79	0.78	0.76	0.85	0.84
	DI	0.57	0.61	0.56	0.61	0.59
	ρ(BCP)	0.09	0.10	0.10	0.10	0.10
Np…N$_2$	Wiberg	0.81	0.80	0.77	0.82	0.80
	DI	0.59	0.63	0.57	0.63	0.58
	ρ(BCP)	0.09	0.10	0.10	0.10	0.10
Np…N$_3$	Wiberg	0.79	0.78	0.76	0.80	0.79
	DI	0.57	0.61	0.55	0.60	0.56
	ρ(BCP)	0.09	0.10	0.10	0.10	0.10
Np…N$_4$	Wiberg	0.24	0.22	0.21	0.21	0.21
	DI	0.21	0.17	0.16	0.17	0.16
	ρ(BCP)	0.05	0.04	0.04	0.04	0.04

Table S8. Computed bond, indices, charges, and spin density data for 3NpNSiMe₃, 3NpNAd, 8UNSiMe₃, 4a, 4b, and 5a

**Cmpd**	**M-N$_{imido/amido}$ bond lengths and indices**		**MDC$_q$ charges**		**MDC$_m$ spin density[b]**	
	**Dist.**	**BI[a]**	**M**	**N**	**M**	**N**
**3NpNSiMe$_3$**	1.9594	2.90	2.81	−1.25	2.76	−0.29
**3NpNAd**	1.9613	2.88	3.05	−1.18	2.84	−0.36
**8UNSiMe$_3$**	2.0400	2.65	2.49	−1.26	2.34	−0.16
**4a**	2.2554	1.63	2.03	−1.36	3.51	−0.09
**4b**	2.2507	1.65	2.05	−1.34	3.51	−0.10
**5a**	2.2142	1.73	2.37	−1.11	3.59	−0.13

[a] Nalewajski-Mrozek bond indices. [b] Positive value is accumulation of electron spin density, negative value is a deficit of electron spin density.

Table S9. NBO data for 3NpNSiMe₃, 3NpNAd, 8UNSiMe₃, 4a, 4b, and 5a

**Cmpd**	**M-N$_{imido/amido}$ σ-bond (%)**				**M-N$_{imido/amido}$ π-bond (%)**			
	**M**	**N**	**M s/p/d/f**	**N s/p**	**M**	**N**	**M s/p/d/f**	**N s/p**
**3NpNSiMe$_3$**	19	81	2/2/48/28	44/56	28	72	0/0/31/69	0/100
					28	72	0/0/31/69	0/100
**3NpNAd**	16	84	4/1/53/42	52/48	34	66	0/0/31/69	0/100
					34	66	0/0/31/69	0/100
**8UNSiMe$_3$**	15	85	1/2/32/65	47/53	16	84	0/0/31/69	0/100
					16	84	0/0/34/66	0/100
**4a**	10	90	3/1/49/47	35/65	13	87	0/0/33/67	0/100
**4b**	10	90	3/1/49/47	35/65	13	87	0/0/34/66	0/100
**5a**	11	89	5/0/47/48	37/63	17	83	0/0/42/58	0/100

Table S10. QTAIM data for the M-NR bonds in 3NpNR (R = SiMe₃, Ad), 8UNSiMe₃, 4a, 4b, and 5a

**Cmpd**	ρ[a]	$\nabla^2\rho$[b]	H[c]	ε[d]
**3NpNSiMe$_3$**	0.19	0.35	−0.14	0.01
**3NpNAd**	0.17	0.49	−0.12	0.04
**8UNSiMe$_3$**	0.16	0.33	−0.11	0.02
**4a**	0.10	0.24	−0.04	0.14
**4b**	0.10	0.24	−0.04	0.14
**5a**	0.10	0.28	−0.04	0.24

[a] Topological electron density. [b] Laplacian. [c] Electronic energy density. [d] Bond ellipticity.

Table S11. Computed properties for reaction profile complexes

Compound	M Spin Density	M Charge	M-N Dist. Å	Spin Contam.	I_{mag} Freq. cm^{-1}
**1**	4.146084	1.322172		0.0001	
**TS 1-I**	4.101198	1.518829	2.76525	0.0191	−389.24
**I**	3.168867	1.770175	2.42212	0.0778	
**TS I-3**	3.218100	1.774361	2.21881	0.0900	−224.96
**Np Benzene (intramolecular)**					
**$3NpSiMe_3$**	2.586150	1.705665	1.93341	0.0988	
**TS 3-II**	3.198446	1.733155	2.21582	0.0834	−1510.34
**II**	3.206684	1.719668	2.25409	0.0633	
**TS II-4a**	3.214346	1.700647	2.26530	0.0694	−1338.31
**4a**	3.215097	1.685047	2.26477	0.0905	
**Np Benzene (intermolecular)**					
**$3NpSiMe_3$**	2.586150	1.705665	1.93341	0.0988	
**TS(b)**	3.170247	1.743525	2.20768	0.0735	−1563.49
**4a**	3.215097	1.685047	2.26477	0.0905	
**Np Toluene (intramolecular)**					
**$3NpSiMe_3$**	2.586765	1.708303	1.93415	0.0089	
**TS 3-II**	3.197491	1.736582	2.21778	0.0830	−1506.30
**II**	3.205881	1.723573	2.25554	0.0631	
**TS II-4a**	3.213976	1.690055	2.26542	0.0872	−1624.08
**4a**	3.208661	1.713365	2.28208	0.0802	
**Np Toluene (intermolecular)**					
**$3NpSiMe_3$**	2.586765	1.708303	1.93415	0.0089	
**TS(t)**	3.181634	1.731698	2.21090	0.0859	−1631.61
**4a**	3.208661	1.713365	2.28208	0.0802	
**Np n-Hexane (intramolecular)**					
**$3NpSiMe_3$**	2.582767	1.698477	1.93027	0.0086	
**TS 3-II**	3.227079	1.691818	2.21915	0.0846	−1457.86
**II**	3.209579	1.705253	2.24736	0.0639	
**TS II-4a**	3.214905	1.671406	2.26009	0.0714	−1584.17
**4a**	3.210215	1.698486	2.27144	0.0705	
**Np n-Hexane (intermolecular)**					
**$3NpSiMe_3$**	2.582767	1.698477	1.93027	0.0086	
**TS(h)**	3.199810	1.717420	2.23562	0.0780	−1650.32
**4a**	3.210215	1.698486	2.27144	0.0705	
**Np Intermolecular**					
**II**	3.206684	1.719668	2.25409	0.0633	
**TS II-4**	2.81677<br>3.25096	1.509186<br>1.493671	2.22763<br>2.24901	0.0497	−1119.32
**4a**	3.242488	1.501591	2.22683	0.0304	
**4b**	3.258844	1.508199	2.23971	0.0295	
**Np Intramolecular-Intermolecular**					
**II**	3.206684	1.719668	2.25409	0.0633	
**TS B1**	3.202569	1.484415	2.23395	0.0248	−1567.30
**III**	3.064410	1.450064	2.22415	0.0254	
**TS B2**	2.90594<br>3.282405	1.452669<br>1.501295	2.23768<br>2.23284	0.0984	−1185.12
**4a**	3.242488	1.501591	2.22683	0.0304	
**IV**	3.285174	1.420247	2.23059	0.0507	
**U(V) Intermolecular**					
**$3UNSiMe_3$**	1.204437	1.827934	1.92543	0.0502	
**TS(bU5)**	2.049020	1.854957	2.22300	0.0104	−1481.40
**4aU**	2.163417	1.803975	2.25242	0.0090	

**U(IV) Intermolecular**					
**8UNSiMe$_3$**	2.177989	1.352894	2.02112	0.0132	
**TS(bU4)**	2.179505	1.589023	2.04206	0.0160	−1043.01
**9U**	3.127976	1.180736	2.38861	0.0087	
**U(V) Intramolecular-Intermolecular**					
**3UNSiMe$_3$**	1.204437	1.827934	1.92543	0.0502	
**TS 3UNSiMe$_3$-II-U**	1.397134	1.879772	2.00465	0.0528	−1160.05
**II-U**	2.170735	1.801884	2.25601	0.0098	
**TS II-U-4aU**	2.166609	1.790762	2.25302	0.0292	−1046.01
**4aU**	2.163417	1.803975	2.25242	0.0090	
**TS II-U-4U**	2.168263<br>2.154724	1.627944<br>1.616223	2.24009<br>2.24736	0.0184	−945.49
**4aU**	2.163417	1.803975	2.25242	0.0090	
**4bU**	2.173991	1.612497	2.24340	0.0128	
**U(IV) Intramolecular-Intermolecular**					
**8UNSiMe$_3$**	2.177989	1.352894	2.02112	0.0132	
**TS 8UNSiMe$_3$-II-Uan**	2.189822	1.400659	2.08832	0.0152	−1005.65
**II-Uan**	2.885159	1.426292	2.29041	0.0170	
**TS II-Uan-4aUan**	2.869753	1.604024	2.27663	0.0132	−1248.55
**4aUan**	3.127976	1.180736	2.38861	0.0082	
**TS II-Uan-4U**	3.134142<br>3.132974	1.186769<br>1.183185	2.35912<br>2.36722	0.0176	−958.72
**4aUan**	3.127976	1.180736	2.38861	0.0087	
**4bUan**	3.133192	1.193761	2.36842	0.0089	

Table S12. Final coordinates and energy for geometry optimized 3NpNSiMe$_3$

1.H	-0.765541	0.182237	-6.131247
2.H	0.622521	1.254754	-5.867487
3.H	1.522754	4.860161	-5.457983
4.H	0.021900	3.913111	-5.507422
5.C	-0.120470	0.626858	-5.354234
6.C	0.764572	4.360344	-4.830899
7.H	-1.436014	2.260641	-4.875722
8.H	0.418085	-0.202524	-4.870504
9.H	1.938496	2.592372	-4.568292
10.H	-2.685322	0.092252	-4.552275
11.H	0.255503	5.147304	-4.252348
12.C	-0.957361	1.422939	-4.333783
13.C	1.428114	3.317400	-3.904223
14.H	-0.917891	-2.396155	-3.993107
15.H	3.254166	4.506680	-3.710947
16.C	-2.077616	0.547184	-3.751545
17.H	0.240765	-3.703098	-3.700704
18.C	-0.230373	-2.819629	-3.243909
19.H	-1.765265	4.840292	-3.133607
20.H	2.563941	0.961338	-3.284180
21.C	2.524891	3.994715	-3.059686
22.H	-2.757631	1.118490	-3.105592
23.H	-1.668208	-0.270831	-3.141652
24.H	-2.771562	3.381983	-3.169695
25.H	-2.771607	-3.839609	-3.037165
26.H	2.199994	-4.389022	-2.966258
27.H	0.559192	-2.076063	-3.064885
28.Si	0.115468	2.244031	-2.956543
29.H	-1.651911	-5.163264	-2.667145
30.H	2.108416	4.753341	-2.381278
31.H	3.086389	3.278764	-2.444262
32.C	-2.167412	4.024262	-2.513574
33.H	0.398518	-6.234664	-2.357137
34.H	2.136376	-0.594755	-2.593489
35.C	-2.081222	-4.229890	-2.271149
36.C	2.188273	0.486041	-2.362269
37.H	3.393302	-5.331332	-2.055770
38.H	-3.978802	-0.896707	-2.261235
39.C	2.619351	-4.550016	-1.962631
40.H	-2.851273	4.481054	-1.779294
41.C	-0.983982	-3.194370	-1.953479
42.H	1.708936	-7.086885	-1.522415
43.H	3.128481	-3.622136	-1.669854
44.N	0.877041	1.011554	-1.932686
45.C	-1.042105	3.250771	-1.798309
46.C	0.920921	-6.325244	-1.391695
47.H	-1.497163	-2.279581	-1.590460
48.H	4.234946	0.362453	-1.567159
49.H	-2.683097	-4.494389	-1.390603
50.H	0.163054	5.036490	-1.383099
51.H	-5.399579	-1.126321	-1.214767
52.C	-4.298067	-1.168294	-1.245037

53.C	3.227372	0.708156	-1.264416
54.H	-4.021307	2.107217	-1.281449
55.H	-1.529919	2.469350	-1.178678
56.H	3.287576	1.784562	-1.058999
57.C	1.536249	-4.978018	-0.953094
58.H	-4.000729	-2.211636	-1.065615
59.H	0.200988	-6.720008	-0.660105
60.H	3.310651	-1.744725	-1.003797
61.C	-0.285876	4.190851	-0.840567
62.Si	0.179031	-3.664622	-0.496584
63.H	0.521835	3.674872	-0.301795
64.C	-4.286222	1.738265	-0.280266
65.H	-5.386651	1.709522	-0.215769
66.H	2.054044	-5.172288	0.006749
67.H	-0.966134	4.615640	-0.085678
68.C	3.240510	-1.387631	0.031152
69.N	2.806561	0.033153	-0.009358
70.Si	-3.586291	0.009352	0.059931
71.Np	0.125569	0.022026	0.009658
72.N	-1.833670	0.016586	0.033378
73.N	0.897767	-2.147551	0.079646
74.H	-1.312178	-5.394387	0.445094
75.H	-3.925628	2.471332	0.456055
76.H	4.245832	-1.469002	0.487792
77.H	-2.731268	-3.360712	0.538154
78.C	-0.873270	-4.484720	0.897159
79.H	2.591270	-3.267894	0.825292
80.C	2.204043	-2.236926	0.764591
81.H	2.132027	2.575550	0.771438
82.H	4.247405	1.222804	1.031322
83.C	3.247118	0.778353	1.198284
84.C	-2.032837	-3.586108	1.355038
85.H	-5.363714	-0.580170	1.697877
86.H	-1.613540	2.956164	1.483813
87.H	0.757751	-5.650732	1.825074
88.C	2.207012	1.828011	1.584215
89.C	-4.262485	-0.542346	1.743352
90.H	-1.664029	-2.623476	1.736831
91.H	2.136235	-1.890971	1.813680
92.N	0.905428	1.179041	1.845048
93.H	-3.903395	-1.543222	2.022555
94.C	-0.023098	-4.927944	2.104041
95.H	-2.612419	-4.064662	2.162907
96.H	3.330306	0.058380	2.021557
97.H	0.609199	4.286340	2.204979
98.H	-2.452718	4.118924	2.532894
99.H	0.471135	-4.068253	2.582288
100.H	2.593857	2.386815	2.453769
101.H	-3.987438	0.153882	2.548644
102.C	-1.939407	3.142313	2.517523
103.H	-0.652089	-5.406630	2.873834
104.H	-2.685127	2.374319	2.763720
105.H	-0.384316	5.281327	3.281988
106.H	-1.577949	-0.067441	2.760153
107.C	0.177230	4.333872	3.216293
108.H	0.361524	-1.598639	3.220883
109.C	-0.744317	3.127416	3.485653
110.Si	0.197943	1.443935	3.449416
111.H	1.008880	4.398242	3.933392
112.C	-1.052211	0.013928	3.733260
113.C	-0.370690	-1.341934	4.000016
114.H	-1.112277	-2.155839	4.032693
115.H	3.341743	0.662297	3.977819
116.H	-1.140006	3.232716	4.513999
117.H	2.303364	-0.498982	4.825760
118.H	-2.658151	1.232129	4.646259
119.C	2.676622	0.535625	4.842392
120.H	0.155714	-1.345092	4.966531
121.H	-2.882120	-0.520702	4.805358
122.C	-2.127247	0.282906	4.805735
123.H	1.999550	2.564898	4.716822
124.C	1.533554	1.570694	4.857606
125.H	3.303594	0.640365	5.744903
126.H	-1.691690	0.317889	5.815410
127.C	0.873943	1.593694	6.255253
128.H	0.460689	0.607546	6.518532
129.H	0.056606	2.325976	6.330313
130.H	1.617486	1.847835	7.029866

Energy: –698.97236557 eV

Table S13. Final coordinates and energy for geometry optimized 3NpNAd

1.C	0.706480	4.176584	-5.212057
2.C	-1.686528	1.240347	-4.934661
3.C	1.778345	1.108774	-4.506286
4.C	3.247834	1.534202	-4.319315
5.C	1.660883	-0.425984	-4.513572
6.C	0.588377	3.863629	-3.704519
7.C	-1.153479	1.138988	-3.490800
8.C	-0.577501	4.669891	-3.097014
9.C	-2.654799	-3.952599	-2.969200
10.C	-1.125335	-4.130640	-2.852037
11.C	-0.486137	-2.816891	-2.347687
12.C	-2.236481	1.575524	-2.489783
13.C	-0.818118	-5.269974	-1.856977
14.C	-3.234517	-3.593921	-1.584608
15.C	3.383521	-1.313313	-1.138430
16.C	2.220409	2.529390	-1.128960
17.C	-2.595463	-2.280149	-1.086949
18.C	-1.054477	-2.425296	-0.956363
19.C	-2.924279	-4.729174	-0.585142
20.C	-1.395175	-4.907089	-0.472027
21.C	1.781624	3.561613	-0.090237
22.C	-0.757452	-3.591190	0.027490

23.C	4.130901	-3.335012	0.176496	75.H	-1.946760	1.405804	-1.439806
24.C	-4.733044	0.741622	0.215961	76.H	4.395867	-0.892832	-1.220305
25.C	3.194590	-2.109208	0.164720	77.H	-3.391222	-5.670508	-0.923251
26.C	5.480625	0.700226	0.964512	78.H	2.675201	-0.476231	-1.242680
27.C	-1.245602	3.097898	0.986557	79.H	3.951188	-3.970929	-0.706568
28.C	-4.436923	1.755969	1.337559	80.H	2.997741	1.883485	-0.680840
29.C	0.032643	3.597834	1.658386	81.H	1.000716	4.189952	-0.536897
30.C	-5.705081	1.993249	2.182709	82.H	-5.521927	1.113766	-0.459374
31.C	4.967798	-0.191766	2.112686	83.H	-3.845832	0.526428	-0.392099
32.C	2.230286	2.551256	2.124079	84.H	-3.008307	-2.000611	-0.105482
33.C	6.097084	-1.138876	2.574476	85.H	-1.167343	-5.712145	0.246971
34.C	-2.808628	-0.600642	2.653235	86.H	5.838000	0.095233	0.117628
35.C	1.955735	1.174256	2.729877	87.H	0.334049	-3.706370	0.123189
36.C	1.794939	-3.224878	3.073727	88.H	5.189842	-3.034955	0.145563
37.C	3.007620	-2.295115	3.255058	89.H	-1.168040	3.286387	-0.101228
38.C	-1.448219	-1.172915	3.081869	90.H	2.614079	4.223929	0.216214
39.C	-3.910790	-1.041972	3.637255	91.H	-3.353642	-4.493247	0.403781
40.C	-3.038204	3.793216	3.878187	92.H	2.154469	-2.487864	0.156740
41.C	-2.878274	2.260840	3.985247	93.H	-5.089410	-0.214635	0.630885
42.C	2.953506	-1.611749	4.636073	94.H	4.707166	1.377291	0.574674
43.C	-1.754384	1.921904	4.983705	95.H	3.993159	-3.962970	1.068054
44.H	-0.158609	3.797570	-5.778517	96.H	-4.188731	2.717598	0.847692
45.H	1.611141	3.741935	-5.660593	97.H	6.330117	1.321243	1.296587
46.H	0.749087	5.267027	-5.378797	98.H	-1.144582	-3.327463	1.024092
47.H	-0.956717	0.887680	-5.677596	99.H	-6.547239	2.299314	1.538315
48.H	1.456633	1.467955	-5.502582	100.H	-2.091415	3.720596	1.323745
49.H	-1.948861	2.277385	-5.196381	101.H	6.376813	-1.852532	1.783447
50.H	2.389402	-0.872848	-5.211981	102.H	0.165992	4.691350	1.549189
51.H	3.884934	1.104737	-5.111381	103.H	3.210417	2.522749	1.632203
52.H	-2.600980	0.635930	-5.057620	104.H	-3.046487	-1.052513	1.673466
53.H	3.380305	2.626616	-4.351977	105.H	1.861596	-3.823053	2.154093
54.H	0.661994	-0.762747	-4.824824	106.H	7.005804	-0.565211	2.826154
55.H	-0.704885	-4.377701	-3.841388	107.H	-6.022140	1.078486	2.707904
56.H	-2.891383	-3.156752	-3.696545	108.H	-5.572366	2.777401	2.941522
57.H	-0.414403	5.754339	-3.222028	109.H	-0.656992	-1.004951	2.331771
58.H	-3.116745	-4.881624	-3.345746	110.H	-0.029273	3.370825	2.730371
59.H	-1.529576	4.430194	-3.594570	111.H	2.264098	3.343641	2.896210
60.H	3.646016	1.178497	-3.356936	112.H	4.754008	0.478651	2.968394
61.H	1.856082	-0.850770	-3.519532	113.H	0.857487	-2.653587	3.025683
62.H	1.517967	4.242564	-3.236317	114.H	5.821650	-1.722562	3.464042
63.H	-0.949793	0.071079	-3.288268	115.H	-4.912323	-0.732048	3.306671
64.H	-0.684815	-1.998323	-3.057292	116.H	3.914926	-2.929282	3.238986
65.H	-3.165659	1.004788	-2.648689	117.H	-3.810490	4.095331	3.156010
66.H	-2.490685	2.640174	-2.586809	118.H	0.978689	1.200092	3.247015
67.H	-1.260506	-6.215586	-2.215623	119.H	-2.097620	4.280867	3.578882
68.H	0.608272	-2.920968	-2.271079	120.H	-1.510777	-2.265108	3.216812
69.H	-0.714446	4.477101	-2.022516	121.H	2.700488	0.998523	3.524357
70.H	0.271530	-5.430032	-1.786196	122.H	-3.925133	-2.140281	3.740948
71.H	2.729249	3.062031	-1.949809	123.H	1.708407	-3.929479	3.918807
72.H	3.232710	-1.963135	-2.015298	124.H	-1.091465	-0.750551	4.031215
73.H	-4.326259	-3.456296	-1.661615	125.H	-3.821206	1.878110	4.424380
74.H	-2.816946	-1.452872	-1.780012	126.H	-3.752012	-0.624053	4.643968

127.H	-3.317806	4.222636	4.855444
128.H	2.046326	-0.997126	4.741069
129.H	-0.759797	2.177248	4.583922
130.H	3.821867	-0.961481	4.822223
131.H	2.928399	-2.361948	5.444904
132.H	-1.736914	0.855367	5.245992
133.H	-1.879722	2.487361	5.923744
134.N	1.061816	1.727148	-1.582372
135.N	-0.468560	-1.186202	-0.485843
136.N	1.204108	2.873347	1.096038
137.N	-1.436238	1.654702	1.264411
138.N	1.968630	0.133820	1.676913
139.Si	0.566426	1.966679	-3.267783
140.Si	3.264613	-1.070744	1.781142
141.Si	-2.822592	1.288741	2.305133
142.Np	0.249501	0.507220	0.194997

Energy: −776.69898461 eV

Table S14. Final coordinates and energy for geometry optimized anion of 8UNSiMe$_3$

1.C	-0.165393	3.723861	-5.532959
2.C	-1.205249	1.419001	-5.525574
3.C	-0.893518	2.667404	-4.676016
4.C	1.835224	0.353824	-4.014061
5.C	2.840339	2.649489	-3.635533
6.C	-1.003332	-2.822446	-3.444687
7.C	1.773832	1.640838	-3.170295
8.C	-2.698578	-4.401940	-2.442234
9.C	-1.218125	-3.972301	-2.445676
10.C	-2.336890	1.010961	-2.301434
11.C	-1.230238	4.791287	-2.031724
12.C	0.116816	4.047738	-2.097854
13.C	2.052207	-4.280506	-1.988547
14.C	4.461388	-0.765858	-1.309112
15.C	-3.232975	1.212578	-1.082289
16.C	1.424761	-3.362547	-0.920374
17.C	0.702287	3.898149	-0.683320
18.C	-0.374363	-6.403959	-0.030968
19.C	-3.367477	-1.071541	-0.181513
20.C	4.325350	1.987145	-0.038981
21.C	-0.981315	-5.061748	0.425950
22.C	2.200595	-3.482196	0.403944
23.C	-2.457090	-2.094463	0.502221
24.C	-3.268275	0.856793	1.333879
25.C	4.404121	-0.488319	1.720153
26.C	-0.758020	-4.850920	1.935077
27.C	-2.194124	1.794391	1.886900
28.C	2.016228	2.967151	2.932283
29.C	0.565213	-1.689180	3.574676
30.C	-0.103750	4.037656	3.784954
31.C	1.159386	-0.272111	3.667931
32.C	0.821244	2.809378	3.887784
33.C	2.148343	-0.190637	4.846490
34.C	-2.360272	-0.167785	5.019599
35.C	-1.456831	1.080605	5.033535
36.C	-0.885924	1.301297	6.449316
37.H	-0.764285	3.998737	-6.420914
38.H	-1.868685	1.671646	-6.373066
39.H	-0.286278	0.988062	-5.950580
40.H	0.800432	3.342027	-5.901418
41.H	0.040252	4.649534	-4.973772
42.H	-1.693857	0.626146	-4.942879
43.H	1.675692	0.560902	-5.084566
44.H	2.684189	2.958174	-4.682408
45.H	-1.867066	3.112917	-4.390362
46.H	-1.358650	-3.091587	-4.456553
47.H	2.819440	-0.135214	-3.920661
48.H	3.849830	2.205641	-3.577144
49.H	1.074696	-0.371780	-3.693034
50.H	-3.040154	-4.661444	-3.461318
51.H	0.054742	-2.536341	-3.530889
52.H	2.850549	3.562604	-3.021612
53.H	-1.547989	-1.919003	-3.128788
54.H	-1.631192	5.026973	-3.029607
55.H	-2.715808	1.708263	-3.072258
56.H	1.608884	-4.133188	-2.983885
57.H	-0.629806	-4.840102	-2.803007
58.H	0.816853	4.667427	-2.690259
59.H	-2.536251	0.000350	-2.715231
60.H	4.240280	-0.303374	-2.283481
61.H	-3.354485	-3.592200	-2.082999
62.H	2.027043	1.341713	-2.132929
63.H	3.134164	-4.081811	-2.079017
64.H	-2.878959	-5.280295	-1.803471
65.H	1.938278	-5.345640	-1.729719
66.H	-1.986279	4.185382	-1.504403
67.H	-1.137737	5.743243	-1.477313
68.H	4.098934	-1.804843	-1.349127
69.H	-4.306259	1.085387	-1.349300
70.H	5.558575	-0.793023	-1.190921
71.H	-0.580554	-6.618120	-1.091142
72.H	1.560366	-2.316474	-1.263560
73.H	-3.368518	-1.277121	-1.260364
74.H	4.018477	2.451954	-0.988653
75.H	-3.097204	2.242448	-0.722722
76.H	1.674552	3.385937	-0.677689
77.H	0.837615	4.877006	-0.191184
78.H	5.428488	1.992022	-0.007223
79.H	0.720047	-6.415053	0.100686
80.H	0.019955	3.313495	-0.040663
81.H	-4.415332	-1.157508	0.180632
82.H	-2.076610	-5.146964	0.280675
83.H	-0.778774	-7.245624	0.561693
84.H	3.267158	-3.246781	0.253267

85.H	-2.944702	-3.080662	0.381002
86.H	3.960403	2.625979	0.779421
87.H	2.146611	-4.502415	0.816882
88.H	1.819747	-2.783165	1.158443
89.H	-4.254479	1.363734	1.252773
90.H	-2.111556	2.671708	1.213625
91.H	5.505388	-0.511316	1.650816
92.H	-2.477738	-1.899456	1.593412
93.H	4.056270	-1.513830	1.918166
94.H	-3.381123	0.018155	2.033274
95.H	1.690344	2.940131	1.882598
96.H	0.311081	-4.774744	2.182147
97.H	-1.236395	-3.930081	2.294796
98.H	-1.170238	-5.695215	2.517836
99.H	4.138657	0.126235	2.594523
100.H	-2.587944	2.205983	2.835945
101.H	-0.527663	4.123558	2.772222
102.H	1.737383	-0.113224	2.735199
103.H	-0.111414	-1.799731	2.714558
104.H	2.537446	3.927720	3.096542
105.H	2.755720	2.164078	3.055757
106.H	1.365580	-2.438886	3.455342
107.H	0.449776	4.973076	3.985438
108.H	-2.770898	-0.372527	4.021090
109.H	-0.944240	3.993551	4.495006
110.H	0.004343	-1.959729	4.483385
111.H	2.958334	-0.930445	4.724230
112.H	1.217957	2.773210	4.921104
113.H	-2.113418	1.948954	4.825983
114.H	2.620325	0.798783	4.936170
115.H	-1.804146	-1.066134	5.329104
116.H	-3.210070	-0.055243	5.717574
117.H	1.652817	-0.407622	5.806661
118.H	-0.278689	2.216576	6.518222
119.H	-0.248645	0.458370	6.762403
120.H	-1.698805	1.386646	7.194976
121.N	-0.914385	1.189326	-1.992892
122.N	-1.080800	-2.055502	-0.020814
123.N	-2.863103	0.304411	0.022554
124.N	1.926173	0.150951	0.082768
125.N	-0.910513	1.092562	2.034835
126.Si	-0.014024	2.322463	-2.973683
127.Si	-0.480013	-3.548239	-0.694690
128.Si	3.637577	0.210103	0.113146
129.Si	-0.133568	1.148957	3.599743
130.U	-0.112406	0.122950	0.010907

Energy: −700.89674378 eV

Table S15. Final coordinates and energy for geometry optimized 4a

1.C	-1.308288	1.695944	-6.114306
2.C	-1.244411	0.478046	-5.171456
3.C	-2.674460	0.044690	-4.787197
4.C	1.649381	-1.636764	-4.777179
5.C	1.501061	1.705649	-4.171778
6.C	1.125377	3.200371	-4.123985
7.C	0.740591	-1.259533	-3.590311
8.C	-0.363138	-2.322787	-3.437092
9.C	2.711584	1.450653	-3.255983
10.C	-1.642839	2.022235	-2.097284
11.C	4.071669	-2.350831	-1.473324
12.C	-2.924407	1.488819	-1.456436
13.C	-1.665370	-5.029231	-0.827954
14.C	0.734640	-4.367611	-0.394354
15.C	1.637040	3.710334	-0.261116
16.C	4.573299	0.369210	-0.142273
17.C	-2.790715	-1.499478	-0.214496
18.C	-0.638933	-4.624488	0.249323
19.C	-3.487699	-0.214987	0.232713
20.C	-2.790761	2.031149	0.949038
21.C	2.103648	2.990734	1.017493
22.C	3.407123	3.630276	1.534923
23.C	4.133935	-2.128666	1.575154
24.C	-0.805542	5.249976	1.868618
25.C	-2.925384	-3.813223	2.126833
26.C	-1.846461	1.749790	2.116578
27.C	-2.759434	-5.141709	2.896885
28.C	0.111217	-2.826398	2.688091
29.C	-3.725997	-2.813642	2.983835
30.C	-0.090399	4.420164	2.952651
31.C	0.804826	-4.077833	3.262858
32.C	2.352050	0.717795	3.565105
33.C	0.859527	5.325226	3.766297
34.C	-0.371011	-1.916419	3.833340
35.C	1.564499	1.994024	3.907409
36.C	0.555057	1.733234	5.042594
37.H	-1.954834	1.477768	-6.982075
38.H	-0.319699	1.975353	-6.505181
39.H	-0.818963	-0.349739	-5.773964
40.H	-3.240606	-0.267063	-5.681480
41.H	-1.732641	2.581758	-5.615044
42.H	1.088980	-1.647483	-5.726163
43.H	1.797077	1.458484	-5.209391
44.H	2.494965	-0.946762	-4.903903
45.H	0.310786	3.456340	-4.814010
46.H	2.066538	-2.648250	-4.639610
47.H	1.992656	3.829226	-4.388391
48.H	-3.234053	0.877656	-4.334274
49.H	-1.014289	-2.357975	-4.323813
50.H	-2.691488	-0.793454	-4.077263
51.H	3.533856	2.152156	-3.476282
52.H	3.110280	0.432296	-3.352500
53.H	0.071271	-3.328291	-3.320446
54.H	0.811867	3.493163	-3.111256

55.H	-1.901251	2.403192	-3.101120
56.H	1.359451	-1.283873	-2.672994
57.H	-1.001550	-2.139186	-2.561068
58.H	3.851796	-1.871608	-2.439424
59.H	2.436020	1.593170	-2.200266
60.H	-3.287019	0.650538	-2.064337
61.H	-1.290570	-5.871442	-1.434643
62.H	5.148415	-2.586570	-1.450841
63.H	-1.867598	-4.198782	-1.521609
64.H	-1.300465	2.910695	-1.532845
65.H	3.522333	-3.305439	-1.443778
66.H	-3.722171	2.256824	-1.426854
67.H	-2.672966	-1.471758	-1.315294
68.H	4.314624	0.945462	-1.041387
69.H	1.070487	-5.239179	-0.981611
70.H	0.696847	-3.515148	-1.088399
71.H	2.416537	3.675395	-1.038274
72.H	-2.625191	-5.342895	-0.392593
73.H	0.728013	3.260053	-0.687306
74.H	5.645987	0.122720	-0.204064
75.H	-4.491823	-0.113143	-0.222457
76.H	1.418356	4.770768	-0.068599
77.H	-3.480155	-2.338627	-0.024328
78.H	1.435537	-1.899216	0.010171
79.H	1.514810	-4.162972	0.353265
80.H	4.438464	1.020915	0.732306
81.H	-2.500891	2.986234	0.491514
82.H	4.186231	3.613308	0.754761
83.H	-0.521273	-5.485520	0.934334
84.H	2.356451	1.947234	0.731069
85.H	-0.085200	5.719592	1.182412
86.H	-3.549802	-4.045499	1.241919
87.H	-3.615378	-0.252477	1.321702
88.H	-3.845904	2.117442	1.277256
89.H	-1.491082	4.646968	1.257145
90.H	5.213737	-2.350506	1.548702
91.H	3.602582	-3.084630	1.707030
92.H	3.250244	4.684026	1.812255
93.H	-2.267954	-5.920838	2.296614
94.H	-1.395321	6.064171	2.323388
95.H	3.810979	3.114897	2.417587
96.H	0.883460	-2.257575	2.133539
97.H	3.936177	-1.512542	2.465453
98.H	-3.932324	-1.874039	2.454242
99.H	1.215859	-4.732393	2.481463
100.H	-2.150212	0.805366	2.605658
101.H	-2.006892	2.534691	2.875116
102.H	-3.743432	-5.535214	3.205184
103.H	3.189836	0.922047	2.884064
104.H	1.672882	5.725986	3.141327
105.H	-4.698551	-3.245871	3.275809
106.H	1.713717	-0.032992	3.076376
107.H	-2.166048	-5.004691	3.814530
108.H	-0.852787	-1.003120	3.457084
109.H	-0.870616	4.078021	3.660762
110.H	0.113216	-4.683478	3.867761
111.H	-3.194989	-2.554567	3.911161
112.H	1.640891	-3.786183	3.920200
113.H	0.311587	6.191061	4.176469
114.H	2.290408	2.744835	4.273468
115.H	-1.091895	-2.436376	4.481899
116.H	1.321136	4.797620	4.613196
117.H	2.772646	0.252626	4.472843
118.H	0.472965	-1.606804	4.469375
119.H	-0.225538	1.023427	4.729356
120.H	0.055037	2.655536	5.374301
121.H	1.055356	1.296314	5.923897
122.N	-0.606889	0.966424	-2.128094
123.N	-2.653091	0.973734	-0.086307
124.N	1.854710	-0.950082	-0.004971
125.N	-1.486375	-1.667290	0.469470
126.N	-0.454990	1.677301	1.632487
127.Si	0.043137	0.529900	-3.711170
128.Si	3.592789	-1.239134	-0.011228
129.Si	-1.251164	-3.174390	1.373910
130.Si	0.734506	2.770568	2.351566
131.Np	-0.132456	0.116226	-0.034366

Energy: −703.00286806 eV

Table S16. Final coordinates and energy for geometry optimized 4b

1.C	-2.928223	-0.049558	-5.922134
2.C	-0.657269	0.945423	-5.462653
3.C	-1.635348	-0.184920	-5.088097
4.C	-2.514639	-3.235238	-3.645028
5.C	-3.147877	-1.955747	-3.066118
6.C	1.873448	-0.603326	-3.008900
7.C	0.592513	-1.436858	-2.975096
8.C	-4.353096	1.226996	-2.740729
9.C	-2.133479	2.400832	-2.503478
10.C	-2.869748	1.057294	-2.352667
11.C	-3.622943	-2.188440	-1.622876
12.C	2.973475	1.073881	-1.578260
13.C	1.936651	2.170304	-1.337669
14.C	3.172601	-1.325732	-1.044261
15.C	0.010080	5.207973	-0.655266
16.C	-0.772105	-4.153276	0.039647
17.C	2.735809	-4.922834	0.202712
18.C	2.987716	-1.375873	0.470906
19.C	4.089509	3.665018	0.853839
20.C	-0.102991	4.613644	0.763575
21.C	2.912990	4.263473	1.118136
22.C	-1.540830	4.131528	1.020972
23.C	-4.502710	1.430904	1.237794

24.C	2.903966	5.724722	1.527842	76.H	-0.321089	-5.157207	0.044427
25.C	-0.624606	-3.473883	1.413900	77.H	-4.765551	1.059349	0.236058
26.C	2.463529	-4.430909	1.637570	78.H	4.158783	2.609267	0.585704
27.C	-3.723037	-1.395165	2.132338	79.H	-1.826662	3.338034	0.315915
28.C	2.139425	-5.630360	2.553602	80.H	-2.265674	4.952111	0.883862
29.C	-1.346417	-4.308220	2.490680	81.H	5.040298	4.209415	0.910085
30.C	1.017484	2.538122	2.857526	82.H	2.253403	6.331164	0.878232
31.C	2.232138	1.681185	3.257314	83.H	3.658424	-2.157930	0.865711
32.C	-2.899313	1.046661	3.807854	84.H	3.353422	-0.428649	0.910665
33.C	1.247402	-2.508437	3.709383	85.H	3.916511	6.157107	1.497754
34.C	0.707597	3.575016	3.953962	86.H	-4.200412	2.485066	1.133735
35.C	2.664459	-2.133008	4.188043	87.H	-3.853506	-1.840438	1.136144
36.C	0.240333	-1.401056	4.063894	88.H	0.106628	5.423854	1.486851
37.H	-2.693777	-0.044970	-7.001863	89.H	-1.354341	1.513326	1.200649
38.H	-0.421158	0.915555	-6.542037	90.H	-1.158909	-2.499835	1.361247
39.H	-3.635144	-0.873593	-5.744689	91.H	-5.415832	1.407259	1.855475
40.H	-3.452922	0.891602	-5.703682	92.H	3.415090	-4.004428	2.012716
41.H	-1.162859	-1.135949	-5.400309	93.H	-1.678738	3.745153	2.041844
42.H	-1.083422	1.935627	-5.253687	94.H	2.950551	-6.377598	2.516903
43.H	0.295110	0.878854	-4.918137	95.H	-2.406502	-4.453303	2.225327
44.H	-2.233777	-3.121727	-4.702611	96.H	1.216433	-6.143831	2.234703
45.H	0.340148	-1.694420	-4.018259	97.H	2.514148	5.848966	2.551597
46.H	-4.464306	1.538249	-3.789063	98.H	-0.896853	-5.308368	2.589000
47.H	-3.213957	-4.087328	-3.581895	99.H	2.428247	0.888741	2.520988
48.H	-4.038535	-1.715914	-3.676985	100.H	-4.700331	-1.425852	2.640410
49.H	2.686456	-1.115346	-3.559960	101.H	-3.035318	-2.034241	2.703661
50.H	1.657086	0.337837	-3.530364	102.H	0.145228	1.861419	2.783350
51.H	-2.162724	2.766433	-3.538802	103.H	2.008626	-5.337714	3.603805
52.H	-1.609293	-3.519870	-3.087073	104.H	-1.315683	-3.837614	3.483327
53.H	-4.935100	0.304488	-2.607951	105.H	3.141539	2.296156	3.337154
54.H	0.806911	-2.404994	-2.484075	106.H	-0.175752	4.187133	3.722296
55.H	3.555336	1.252461	-2.502765	107.H	3.050841	-1.254323	3.650056
56.H	-4.827123	2.007396	-2.120438	108.H	0.428908	-0.485072	3.485985
57.H	1.227158	2.182732	-2.188118	109.H	-2.579499	2.101022	3.808800
58.H	-1.074951	2.333247	-2.207646	110.H	2.067498	1.194860	4.231888
59.H	-2.600451	3.176462	-1.873878	111.H	3.383170	-2.954551	4.047142
60.H	-4.254701	-3.091242	-1.547666	112.H	-0.796510	-1.703488	3.858530
61.H	2.841409	-2.285062	-1.463415	113.H	1.555783	4.259403	4.109789
62.H	-0.194500	4.443794	-1.419095	114.H	-3.843483	0.989694	4.374314
63.H	2.457014	3.141015	-1.375452	115.H	-2.139686	0.467993	4.354305
64.H	-2.841413	0.796234	-1.276570	116.H	0.943649	-3.420764	4.257024
65.H	4.231566	-1.175092	-1.333251	117.H	0.517324	3.073877	4.917620
66.H	-4.210166	-1.343680	-1.239197	118.H	2.663652	-1.879956	5.261353
67.H	-0.721585	6.020851	-0.803998	119.H	0.300224	-1.136104	5.132572
68.H	-2.768286	-2.331822	-0.945217	120.N	-0.487746	-0.704402	-2.278001
69.H	1.009529	5.620362	-0.860587	121.N	2.317423	-0.261280	-1.630997
70.H	3.675922	1.071485	-0.735958	122.N	1.239694	1.957209	-0.044842
71.H	-0.292901	-3.576734	-0.765640	123.N	1.566777	-1.607822	0.796293
72.H	3.084449	-4.119677	-0.458856	124.N	-1.622615	0.513707	1.142856
73.H	1.836007	-5.360168	-0.258527	125.Si	-1.981385	-0.425843	-3.192284
74.H	-1.832874	-4.278249	-0.228035	126.Si	1.253576	3.291067	1.107659
75.H	3.512727	-5.707530	0.204329	127.Si	1.186971	-2.982879	1.841823

128.Si	-3.132504	0.393406	2.042239
129.Np	0.134388	-0.060325	-0.141358

Energy: −695.07570595 eV

Table S17. Final coordinates and energy for geometry optimized 5a

1.C	0.669245	4.165443	-5.212886
2.C	-1.646730	1.152871	-4.944351
3.C	1.793210	1.114987	-4.470883
4.C	3.255674	1.564887	-4.280587
5.C	1.700541	-0.421685	-4.464990
6.C	0.557633	3.856214	-3.704237
7.C	-1.141655	1.099923	-3.487634
8.C	-0.609686	4.657299	-3.093473
9.C	-2.756008	-3.886862	-2.998795
10.C	-1.262339	-4.273128	-2.933999
11.C	-0.448767	-3.067036	-2.415017
12.C	-2.254908	1.552436	-2.527271
13.C	-1.080497	-5.469377	-1.974972
14.C	-3.243490	-3.486771	-1.589376
15.C	3.548914	-1.385883	-1.168587
16.C	2.185506	2.575066	-1.116131
17.C	-2.418000	-2.284873	-1.075289
18.C	-0.921366	-2.646970	-0.998033
19.C	-3.067664	-4.686643	-0.631990
20.C	-1.574831	-5.076035	-0.566231
21.C	1.742206	3.600750	-0.075492
22.C	-0.758741	-3.869340	-0.053853
23.C	4.390367	-3.333994	0.206702
24.C	-4.776326	0.834280	0.204620
25.C	3.384121	-2.165948	0.148777
26.C	5.524507	0.732669	0.968539
27.C	-1.275318	3.188301	1.028240
28.C	-4.487038	1.825586	1.348768
29.C	0.024339	3.630217	1.702195
30.C	-5.756842	2.036564	2.198039
31.C	5.020471	-0.170889	2.111554
32.C	2.218024	2.538502	2.105065
33.C	6.160777	-1.091661	2.597362
34.C	-2.845007	-0.542460	2.623593
35.C	1.971324	1.126669	2.640412
36.C	1.833127	-3.210930	3.031365
37.C	3.057339	-2.296558	3.221788
38.C	-1.487685	-1.133272	3.043170
39.C	-3.942923	-0.989654	3.611095
40.C	-3.092234	3.830564	3.912598
41.C	-2.912776	2.298538	3.990703
42.C	3.002093	-1.615422	4.604704
43.C	-1.777448	1.956226	4.975299
44.H	-0.197922	3.784944	-5.775412
45.H	1.572079	3.730234	-5.664628
46.H	0.710886	5.255315	-5.382915
47.H	-0.898469	0.788815	-5.662809
48.H	1.473303	1.459250	-5.472856
49.H	-1.914178	2.179724	-5.239550
50.H	2.446103	-0.864784	-5.147747
51.H	3.904203	1.135026	-5.063177
52.H	-2.552690	0.535836	-5.069154
53.H	3.371164	2.658665	-4.326384
54.H	0.711648	-0.776646	-4.788238
55.H	-0.905210	-4.546205	-3.940886
56.H	-2.902188	-3.050620	-3.704220
57.H	-0.445028	5.742941	-3.204819
58.H	-3.351345	-4.736072	-3.377036
59.H	-1.559751	4.424434	-3.598435
60.H	3.653113	1.228252	-3.311043
61.H	1.886085	-0.834554	-3.463563
62.H	1.485932	4.243746	-3.241073
63.H	-0.924395	0.039893	-3.252825
64.H	-0.555917	-2.206229	-3.095018
65.H	-3.158236	0.933456	-2.655113
66.H	-2.552107	2.595831	-2.704752
67.H	-1.643863	-6.343318	-2.345452
68.H	0.626891	-3.315292	-2.377877
69.H	-0.751088	4.450692	-2.022477
70.H	-0.017592	-5.764838	-1.934859
71.H	2.680311	3.114174	-1.941559
72.H	3.408869	-2.047031	-2.038484
73.H	-4.307492	-3.201186	-1.632052
74.H	-2.547997	-1.416597	-1.742196
75.H	-1.971381	1.484453	-1.463465
76.H	4.555055	-0.952126	-1.253159
77.H	-3.667523	-5.543022	-0.986118
78.H	2.831826	-0.557646	-1.268969
79.H	4.258054	-4.002659	-0.660641
80.H	2.971790	1.936170	-0.674589
81.H	0.951645	4.220009	-0.518055
82.H	-5.566332	1.216046	-0.463970
83.H	-3.886818	0.641641	-0.408541
84.H	0.839978	-1.834998	-0.436406
85.H	-2.769796	-1.983870	-0.075453
86.H	-1.441767	-5.925391	0.124851
87.H	5.913709	0.136582	0.128874
88.H	0.312090	-4.134011	0.008944
89.H	5.429389	-2.973057	0.179297
90.H	-1.212006	3.423039	-0.050120
91.H	2.567120	4.272415	0.233727
92.H	-3.437108	-4.429155	0.375543
93.H	2.380564	-2.635530	0.136820
94.H	-5.126045	-0.133808	0.597180
95.H	4.732563	1.380350	0.565231
96.H	4.274417	-3.944354	1.113497
97.H	-4.245110	2.798925	0.879050
98.H	6.348417	1.382251	1.310560

99.H	-1.079544	-3.582918	0.961183
100.H	-6.599928	2.356133	1.561363
101.H	-2.097914	3.815739	1.411983
102.H	6.481138	-1.791661	1.809745
103.H	0.183928	4.723808	1.631811
104.H	3.194938	2.553370	1.605938
105.H	-3.097560	-0.988798	1.644144
106.H	1.906785	-3.824889	2.122669
107.H	7.047201	-0.498059	2.879924
108.H	-6.071561	1.108002	2.699984
109.H	-5.627143	2.801765	2.976627
110.H	-0.697111	-0.991738	2.285617
111.H	-0.034490	3.366320	2.766093
112.H	2.244456	3.286227	2.920167
113.H	4.773903	0.495987	2.961669
114.H	0.904793	-2.626530	2.957767
115.H	5.875886	-1.691101	3.473893
116.H	-4.944985	-0.667667	3.294353
117.H	3.955351	-2.944127	3.217565
118.H	-3.861580	4.136927	3.188974
119.H	0.977669	1.109443	3.133518
120.H	-2.155411	4.335955	3.631372
121.H	-1.565100	-2.223461	3.186771
122.H	2.688507	0.939414	3.456040
123.H	-3.963702	-2.089277	3.698789
124.H	1.719814	-3.902060	3.884358
125.H	-1.115302	-0.708076	3.985284
126.H	-3.847749	1.897374	4.430308
127.H	-3.771772	-0.587685	4.622333
128.H	-3.386666	4.236708	4.895436
129.H	2.099620	-0.993611	4.710137
130.H	-0.787795	2.217352	4.567271
131.H	3.875035	-0.972874	4.795410
132.H	2.967923	-2.368097	5.410938
133.H	-1.753907	0.888052	5.230765
134.H	-1.894523	2.515082	5.920220
135.N	1.025831	1.763210	-1.550998
136.N	-0.141513	-1.507052	-0.503558
137.N	1.173955	2.897231	1.105742
138.N	-1.489562	1.737080	1.242553
139.N	2.049944	0.109722	1.560354
140.Si	0.559514	1.963541	-3.246362
141.Si	3.344709	-1.082383	1.742523
142.Si	-2.860194	1.355330	2.292819
143.Np	0.229114	0.545295	0.240270

Energy: −780.87822609 eV

Table S18. Final coordinates for 1

Np -0.019253 -0.003514 -0.046601
Si -0.725543 -3.361079 1.222037
Si -2.261104 2.745166 0.807318
Si 3.256645 0.741130 -1.445473
N -0.794959 -2.208244 -0.132975
N -1.816489 1.461128 -0.339449
N 1.547728 0.357592 -1.747808
N -1.102332 -0.434778 -2.325694
C -0.020784 -2.906837 4.041659
C -1.572048 1.309285 3.259097
C -0.801534 3.697628 3.178742
C 0.303535 -2.450361 2.614398
C -1.094838 2.445289 2.345842
C 1.819064 -2.490821 2.384656
C -4.544740 3.690054 2.347717
C 0.284189 -6.018601 1.851967
C -3.293815 -2.557747 2.280522
C -2.468472 -3.810386 1.976939
C -4.699883 1.291806 1.557965
C -4.159097 2.690579 1.251590
C 4.744956 0.787967 1.098755
C 2.857855 2.434728 0.904289
C 0.066312 -5.058569 0.676957
C -3.277039 -4.797165 1.126731
C 3.365750 1.050051 0.483352
C -1.910599 4.546851 0.147986
C 1.330361 -4.925194 -0.176098
C -0.490968 4.657166 -0.413482
C -2.938955 5.047953 -0.872388
C 4.021191 -2.027288 -1.219825
C -2.605388 1.398048 -1.565751
C -1.438043 -2.699788 -1.347331
C 4.483046 -0.717810 -1.863465
C 5.283763 2.716039 -2.112651
C -2.500963 0.020812 -2.218446
C 3.866155 2.270704 -2.490965
C -1.010106 -1.886614 -2.567702
C 2.900077 3.458321 -2.510099
C 1.166739 0.175215 -3.143881
C -0.341900 0.336838 -3.326721
C 4.738278 -0.914549 -3.361775
H 0.552154 -2.327152 4.778423
H 0.238841 -3.960784 4.193919
H -1.080072 -2.790953 4.289536
H -0.808240 1.046781 4.004185
H -0.116296 3.467504 4.006230
H -2.467912 1.604255 3.816389
H -1.716088 4.102965 3.626460
H -4.109584 3.416960 3.316608
H 2.346711 -1.824922 3.081711
H -1.834467 0.399503 2.706413
H -0.342994 4.497264 2.589504
H 1.061662 -5.654944 2.534567
H 2.213927 -3.498687 2.552114
H -2.250291 -4.307815 2.933900
H -0.021294 -1.395659 2.546552

H -4.337356 0.911826 2.519158
H -2.770485 -1.870545 2.955075
H -0.625215 -6.169885 2.444198
H -5.634268 3.721890 2.487897
H -0.130998 2.137218 1.894348
H -4.249981 -2.813636 2.757851
H -4.219218 4.710960 2.118441
H 4.723595 0.939166 2.186849
H 2.747316 2.507909 1.995205
H 0.609217 -7.007616 1.499885
H -5.797601 1.297798 1.609517
H 2.105220 -2.207174 1.364957
H -4.228115 -5.047892 1.617027
H -3.523823 -1.999502 1.366289
H -4.407941 0.566379 0.792300
H -1.978262 5.202536 1.028843
H 2.684242 0.293877 0.921090
H 2.181540 -4.555877 0.405107
H 5.498833 1.473820 0.696031
H 3.563748 3.218062 0.606863
H -2.744469 -5.739387 0.959186
H 5.099010 -0.231212 0.919127
H 1.895312 2.691287 0.446723
H 0.269362 4.422558 0.340191
H -4.648925 3.024586 0.324158
H -0.704110 -5.508723 0.032606
H -3.525708 -4.382118 0.143097
H 1.626466 -5.897126 -0.594960
H 3.945493 -1.947536 -0.129431
H 1.185798 -4.233833 -1.012292
H -0.279795 5.671322 -0.780146
H -3.958628 5.049979 -0.472577
H -0.339713 3.964035 -1.248970
H -3.683827 1.571875 -1.402505
H 5.317177 3.146171 -1.104420
H -2.712513 6.077905 -1.181612
H -2.539975 -2.684777 -1.272434
H 4.716598 -2.850376 -1.435378
H 2.835607 3.951489 -1.534580
H 3.033192 -2.323587 -1.590608
H -1.184900 -3.748890 -1.580356
H -2.945570 4.436561 -1.782186
H -3.027309 -0.696622 -1.582333
H 5.441275 -0.432309 -1.404190
H -2.301881 2.168636 -2.296494
H 6.005905 1.892017 -2.141049
H 5.650752 3.490917 -2.799959
H 1.885124 3.156733 -2.787721
H 0.039270 -2.111427 -2.778687
H -2.987653 0.014425 -3.209210
H -0.594390 1.391589 -3.186023
H 3.229018 4.218478 -3.232422
H -1.600215 -2.169060 -3.456837
H 3.913444 1.884464 -3.520348
H 1.474190 -0.811644 -3.534257
H 1.634793 0.907559 -3.825495
H 5.472207 -1.715006 -3.530762
H 3.827338 -1.202072 -3.899751
H 5.132664 -0.013178 -3.842483
H -0.648261 0.051950 -4.348222

Table S19. Final coordinates for TS 1-I

Np 0.055347 -0.080896 -0.001146
Si 0.003410 -3.854756 -0.390444
Si -4.399732 -0.153790 0.711231
Si 0.197982 2.346617 -2.983498
Si 0.353189 1.461347 3.487522
N -3.205371 -0.199695 -2.495008
N -2.870341 -0.162184 -1.395622
N -2.705136 -0.126301 -0.157040
N 0.792595 1.022477 -1.929990
N 2.722810 -0.161935 -0.110245
N 0.798942 -2.302948 0.019419
N 0.964258 1.055099 1.854586
C 0.031886 -1.252462 4.478810
C -1.954362 2.920194 2.536218
C -1.590707 -3.938471 2.003766
C 1.968521 -4.518698 -2.498476
C 0.567420 -6.424704 -1.639974
C 1.232695 -5.089659 -1.283068
C -1.236540 -3.047741 -2.900419
C -2.618623 -4.587460 -1.473133
C -1.570701 -3.467740 -1.464379
C 0.516880 -5.307338 2.098551
C -0.616488 -4.802497 1.199902
C -5.132466 1.580246 0.473047
C -5.441651 -1.453912 -0.200319
C -4.160815 -0.633596 2.522817
C -2.521904 3.408467 -3.284308
C 0.991779 4.924158 -4.066271
C 2.049031 4.379519 -1.847398
C 1.501971 3.800692 -3.154385
C 1.137758 1.691844 -5.723065
C -0.879008 0.451372 -4.912558
C -0.108937 1.770194 -4.833106
C -1.323252 4.086103 -1.184560
C -1.489664 2.981349 -2.234248
C 2.080351 0.480550 -2.402631
C 3.141848 0.578541 -1.317251
C 1.260102 2.277881 6.132217
C -1.754916 0.360718 5.204894
C -0.770058 -0.006718 4.089407
C 0.070743 4.314189 3.070029
C -0.743974 3.076527 3.456687

C 3.233653 0.488981 1.110289
C 3.136563 -1.574812 -0.185884
C 2.154319 -2.460561 0.565838
C 2.291714 1.604468 1.539745
C 1.793103 1.863903 4.754153
C 2.879486 0.794647 4.913620
H 2.076506 2.646597 6.768625
H 0.510169 3.074136 6.077836
H 0.805728 1.432420 6.661338
H -1.234229 0.601395 6.138266
H 3.720781 1.182395 5.504769
H 2.280180 2.753849 4.328097
H -2.429154 -0.477908 5.426892
H 0.574321 -1.096052 5.417685
H -2.379500 1.225173 4.956273
H 2.505745 -0.091912 5.435854
H -1.117754 3.220519 4.480548
H 3.284515 0.457467 3.955604
H -0.627182 -2.116569 4.635252
H 0.894028 4.505171 3.767036
H 0.765552 -1.527371 3.713857
H -1.363267 -0.268057 3.198054
H -0.556119 5.216648 3.055164
H -2.615156 2.109890 2.861785
H 0.120378 -5.871471 2.954501
H -3.656248 0.144287 3.097970
H 2.763606 2.131156 2.381350
H 1.105550 -4.479004 2.508906
H -2.556644 3.838830 2.503331
H 0.506148 4.208952 2.069033
H 3.262031 -0.259110 1.908002
H -1.949400 -4.459988 2.902097
H -3.607447 -1.570816 2.625644
H 2.193942 -2.205602 1.639045
H -1.103774 -3.013366 2.334506
H 1.207653 -5.971631 1.567893
H -1.645624 2.703645 1.506349
H -5.152258 -0.782546 2.967348
H 4.264132 0.849819 0.949499
H 2.241233 2.353662 0.732850
H 2.537390 -3.491275 0.505861
H -2.472549 -3.658217 1.416333
H 4.169454 -1.693896 0.183315
H -4.543753 2.343556 0.989062
H -1.166385 -5.681759 0.832853
H -2.278122 4.293455 -0.682616
H 1.996555 -5.311460 -0.522686
H -6.155266 1.613093 0.865313
H -0.593869 3.827302 -0.410229
H 3.124571 -1.870908 -1.238524
H 0.061617 -6.887586 -0.785615
H -5.035150 -2.461019 -0.068899
H 3.258537 1.632143 -1.051262
H -1.903735 2.104916 -1.716643
H -5.174399 1.839937 -0.589788
H -6.459551 -1.445995 0.206800
H -0.995995 5.026790 -1.640798
H -2.967009 -4.852832 -0.470030
H 4.123488 0.215155 -1.665633
H -2.032202 -2.603503 -0.961638
H 2.444478 -3.557571 -2.283799
H 1.310825 -7.146607 -2.005698
H -3.458926 3.721028 -2.802392
H -5.515807 -1.249842 -1.272799
H 2.755190 -5.206520 -2.838874
H 1.986425 -0.571841 -2.719595
H -0.175286 -6.304561 -2.437157
H 2.350902 3.597692 -1.143579
H 1.311162 5.006183 -1.337337
H -2.231455 -5.502469 -1.934283
H -0.469266 -2.267673 -2.939080
H 1.294223 -4.361411 -3.346480
H -3.499941 -4.287482 -2.056518
H -2.770436 2.603057 -3.981922
H -0.280109 -0.375723 -4.515359
H -1.812788 0.470672 -4.343822
H 2.480320 1.007643 -3.282744
H -2.170308 4.262641 -3.875419
H -0.869807 -3.898565 -3.485661
H 2.928581 5.011248 -2.035393
H -2.124369 -2.661827 -3.416671
H 0.166546 5.475949 -3.601064
H -1.131471 0.200022 -5.952563
H 2.350386 3.325013 -3.669795
H 1.806589 0.878867 -5.417898
H -0.750983 2.560027 -5.251205
H 0.631944 4.555891 -5.032750
H 1.784784 5.656356 -4.273770
H 1.722418 2.617182 -5.729067
H 0.852482 1.486467 -6.764532

Table S20. Final coordinates for I

Np -0.008144 -0.017491 -0.241783
Si 3.450221 -1.559428 -0.512454
Si 0.688192 0.291763 3.916900
Si -3.219879 -2.042379 -0.165088
Si -0.415631 3.738212 -0.304669
N -0.368384 -2.266335 2.745423
N -0.137779 -1.380716 1.967835
N 0.320824 -0.121838 2.155624
N -1.683692 -1.424899 -0.901183
N -0.176273 -0.045060 -2.910526
N 2.013086 -0.627283 -1.113343
N -0.579052 2.078509 -1.008774

C 2.481716 3.805515 -0.042313
C -2.044301 3.188387 2.015211
C 4.537699 0.624348 1.020390
C 3.087785 -3.732939 -2.486249
C 5.389056 -3.494995 -1.501593
C 4.114817 -2.748278 -1.918272
C 2.089476 -3.737946 0.865294
C 4.189101 -2.907564 1.963775
C 2.977978 -2.513044 1.108707
C 5.531773 0.365847 -1.279181
C 4.929939 -0.367367 -0.076384
C -0.937767 0.309809 4.913108
C 1.893395 -0.986973 4.660029
C 1.529430 1.993294 4.001640
C -4.119669 -2.071266 2.611894
C -6.035326 -2.307335 -0.833626
C -4.920866 -0.323491 -1.906694
C -4.718899 -1.752014 -1.394782
C -3.469512 -4.832239 -1.145560
C -1.916086 -4.481628 0.783028
C -3.193168 -3.984173 0.102730
C -4.343145 0.137378 1.438818
C -3.517033 -1.149567 1.544731
C -1.426697 -2.035423 -2.218620
C -1.266414 -0.965803 -3.284250
C -0.304655 6.529993 -1.089749
C 1.208792 4.927744 1.808504
C 1.190795 3.787648 0.782972
C -3.253091 4.223434 0.063222
C -1.933847 4.158634 0.838215
C -0.466944 1.334584 -3.344273
C 1.128295 -0.509368 -3.415134
C 2.234317 -0.076672 -2.466263
C -1.304104 2.044204 -2.292632
C -0.417226 5.121399 -1.689851
C 0.599777 4.957888 -2.824166
H -0.467715 7.291535 -1.864469
H -1.036016 6.716037 -0.296624
H 0.691112 6.712920 -0.671144
H 1.261501 5.906693 1.319875
H 0.438337 5.719959 -3.599130
H -1.417863 5.056012 -2.142064
H 2.089296 4.850942 2.459970
H 2.620314 4.770499 -0.542049
H 0.325776 4.937337 2.455015
H 1.629029 5.076215 -2.471621
H -1.732889 5.159789 1.246686
H 0.535552 3.982514 -3.313672
H 3.359973 3.653775 0.598176
H -3.237229 4.983677 -0.725215
H 2.495927 3.028517 -0.813810
H 1.170017 2.841330 1.345138
H -4.090922 4.467642 0.730606
H -1.147767 3.195786 2.643119
H 6.416332 0.945462 -0.980682
H 0.868018 2.811415 3.708623
H -1.552843 3.041404 -2.680176
H 4.822157 1.076026 -1.718778
H -2.897222 3.437901 2.660736
H -3.490051 3.262800 -0.409303
H 0.482932 1.867019 -3.443589
H 5.371711 1.293050 1.274976
H 2.435230 2.031800 3.389598
H 2.280231 1.024100 -2.444683
H 3.700501 1.253066 0.697684
H 5.849826 -0.318631 -2.073511
H -2.192615 2.158722 1.671315
H 1.829328 2.173273 5.041265
H -0.954307 1.332645 -4.333309
H -2.271429 1.528494 -2.192564
H 3.189257 -0.390785 -2.909023
H 4.230806 0.120891 1.943114
H 1.298194 -0.144957 -4.441797
H -1.645861 1.041692 4.512371
H 5.709119 -1.030111 0.328079
H -4.367137 0.658144 2.405369
H 4.400656 -2.072543 -2.737863
H -0.729267 0.575216 5.956347
H -3.942854 0.839059 0.700511
H 1.109932 -1.601420 -3.450658
H 6.172594 -2.825087 -1.131755
H 2.872277 -0.939081 4.173806
H -2.196239 -0.394115 -3.339426
H -2.518123 -0.867328 1.899521
H -1.418187 -0.672901 4.905735
H 2.039749 -0.762755 5.723650
H -5.383625 -0.069005 1.165840
H 4.805865 -2.049906 2.249936
H -1.082020 -1.396885 -4.281528
H 2.394091 -1.787608 1.690658
H 2.140805 -3.248876 -2.741568
H 5.811105 -4.047514 -2.352433
H -4.246470 -1.528624 3.558350
H 1.514106 -2.008655 4.579727
H 3.468913 -4.213233 -3.397865
H -0.523800 -2.667603 -2.195096
H 5.185949 -4.231233 -0.715813
H -3.984745 0.132678 -2.242756
H -5.343006 0.332083 -1.139187
H 4.841662 -3.619979 1.446846
H 1.240871 -3.520039 0.209635
H 2.858453 -4.533025 -1.775718
H 3.858318 -3.393239 2.891249
H -3.485564 -2.937943 2.818917

H -1.045137 -4.340688 0.133200
H -1.696703 -3.961534 1.719070
H -2.234118 -2.706100 -2.546674
H -5.110157 -2.442060 2.321286
H 2.659210 -4.556679 0.411231
H -5.618014 -0.307969 -2.756058
H 1.677960 -4.113608 1.809733
H -6.384751 -1.724536 0.026168
H -1.983760 -5.556006 1.005518
H -4.458830 -2.365543 -2.270253
H -2.642181 -4.784644 -1.863220
H -4.030450 -4.153686 0.796287
H -5.954555 -3.349663 -0.508228
H -6.831553 -2.265683 -1.589845
H -4.383103 -4.543325 -1.674517
H -3.580666 -5.889798 -0.868477

Table S21. Final coordinates for TS I-3

Np -0.008872 -0.018112 -0.196275
Si 3.447398 -1.603091 -0.479251
Si -0.418513 3.729647 -0.282945
Si -3.264309 -2.044133 -0.168373
Si 0.835256 0.365559 3.725462
N 1.992588 -0.721360 -1.091974
N -0.198926 -0.074356 -2.893924
N -0.580326 2.075000 -0.989909
N -1.706807 -1.442891 -0.863403
N -0.364074 -2.288798 2.769791
N -0.219210 -1.653399 1.853801
N 0.489815 0.073330 1.963830
C 5.488383 -3.443846 -1.446281
C 4.156840 -3.040945 1.959906
C 5.463961 0.423905 -1.114242
C 2.093482 -3.853916 0.779207
C 2.962278 -2.630754 1.090342
C 4.392478 0.567764 1.159944
C 4.862876 -0.371560 0.048280
C 2.008836 -0.945235 4.473914
C -4.932379 -0.308685 -1.926120
C -2.078113 -4.498586 0.915875
C -4.367344 0.166415 1.398878
C -3.432625 -4.835662 -1.155596
C -3.274608 -3.985995 0.112413
C -4.287511 -2.046755 2.573231
C -3.598112 -1.152265 1.535317
C 1.266618 4.942016 1.765487
C -3.239120 4.223194 0.194326
C 1.099682 -0.553385 -3.394941
C 2.211269 -0.152579 -2.438047
C 3.249001 -3.692117 -2.564962
C 4.218427 -2.711168 -1.898247
C -1.969649 3.146179 2.086303
C -1.894089 4.133768 0.920872
C 0.513070 4.985244 -2.814719
C -0.420372 6.529906 -1.070763
C -0.490391 5.116129 -1.664238
C 2.485322 3.817031 -0.121434
C 1.223697 3.796007 0.747454
C -1.438799 -2.056139 -2.177936
C -1.294444 -0.990662 -3.250458
C -0.480557 1.307572 -3.322351
C -1.310948 2.025401 -2.269000
C -6.059192 -2.321642 -0.927291
C -4.730832 -1.745160 -1.435378
C -0.790110 0.387620 4.746226
C 1.720286 2.041629 3.933593
H -0.627903 7.282567 -1.843665
H -1.142396 6.690508 -0.263544
H 0.575776 6.752548 -0.672472
H 1.253786 5.919861 1.271796
H 0.283625 5.706465 -3.611389
H -1.495273 5.013300 -2.100178
H 2.188651 4.899636 2.360394
H 2.604934 4.781061 -0.627969
H 0.426369 4.924124 2.467120
H 1.537136 5.189960 -2.487331
H -1.670047 5.126825 1.337081
H 0.512289 3.990685 -3.269167
H 3.385527 3.667005 0.488468
H -3.245544 5.001925 -0.575952
H 2.472928 3.038040 -0.890894
H 1.227971 2.849833 1.310772
H -4.052941 4.455192 0.895099
H -1.044363 3.121823 2.670589
H 6.302845 1.045682 -0.771613
H 1.089315 2.888976 3.658508
H -1.564287 3.017994 -2.665446
H 4.730004 1.102778 -1.563331
H -2.786275 3.402308 2.775013
H -3.497905 3.276143 -0.293967
H 0.473412 1.833367 -3.422371
H 5.193527 1.251757 1.473276
H 2.651753 2.090251 3.363038
H 2.271634 0.948203 -2.405087
H 3.548295 1.178919 0.822669
H 5.847807 -0.221981 -1.911680
H -2.155391 2.126132 1.732982
H 1.973256 2.149801 4.995695
H -0.970440 1.313636 -4.310740
H -2.276634 1.507907 -2.159601
H 3.161113 -0.471333 -2.889244
H 4.059422 0.021224 2.048087
H 1.283329 -0.180323 -4.416459
H -1.656633 0.087640 4.150554

H 5.659058 -1.011507 0.456415
H -4.402868 0.693760 2.361385
H 4.531474 -1.993824 -2.671593
H -0.986127 1.392750 5.134394
H -3.916225 0.846949 0.670532
H 1.062837 -1.644501 -3.444279
H 6.222930 -2.773742 -0.986964
H 2.979779 -0.947203 3.970492
H -2.224508 -0.418524 -3.295147
H -2.604165 -0.905081 1.929893
H -0.719087 -0.295578 5.599459
H 2.181934 -0.691894 5.527164
H -5.404998 -0.003795 1.092080
H 4.767903 -2.188018 2.271873
H -1.125485 -1.427739 -4.248427
H 2.358631 -1.925395 1.677049
H 2.307189 -3.216866 -2.853734
H 5.982251 -3.930207 -2.299020
H -4.430463 -1.500834 3.515572
H 1.599199 -1.958197 4.442599
H 3.691525 -4.126168 -3.472355
H -0.523661 -2.669798 -2.150848
H 5.261119 -4.232937 -0.720621
H -3.990721 0.173300 -2.205648
H -5.404721 0.321628 -1.166856
H 4.819099 -3.743639 1.442312
H 1.250506 -3.614412 0.122919
H 2.997675 -4.526872 -1.902738
H 3.812309 -3.543169 2.873827
H -3.711844 -2.947159 2.806682
H -1.135978 -4.314081 0.388078
H -1.996167 -4.028716 1.899388
H -2.232589 -2.744342 -2.503131
H -5.280809 -2.368311 2.238017
H 2.677720 -4.641692 0.290159
H -5.586541 -0.286820 -2.808789
H 1.682010 -4.289341 1.699506
H -6.432710 -1.766561 -0.059107
H -2.151999 -5.582792 1.079632
H -4.438089 -2.338941 -2.314206
H -2.525687 -4.819374 -1.770641
H -4.176838 -4.147084 0.721250
H -5.983285 -3.374012 -0.634854
H -6.835448 -2.257569 -1.702511
H -4.268587 -4.521942 -1.788872
H -3.609705 -5.886923 -0.888937

Table S22. Final coordinates for 3NpNSiMe3

Np 0.133608 -0.034625 0.030370
Si 0.232850 1.358527 3.498763
Si -3.581740 0.129515 -0.024681
Si 0.205466 -3.725351 -0.504297
Si 0.059474 2.326916 -2.948700
N 0.963095 1.047140 1.877864
N 0.955344 -2.179051 0.049936
N -1.786525 0.154013 -0.094463
N 2.809987 -0.004428 -0.020135
N 0.809649 1.117555 -1.841788
C 0.962756 1.763368 6.275582
C 1.591652 1.502023 4.900137
C -2.138285 0.317944 4.852692
C 2.604733 0.357537 4.989958
C -0.382524 -1.376930 4.261492
C -1.047115 -0.060365 3.844654
C -0.718759 3.056832 3.527343
C 0.202172 4.276459 3.427224
C -1.796906 3.107190 2.443110
C 0.088687 -5.016699 2.097673
C -4.231081 -0.352291 1.708307
C 2.248553 1.718019 1.636739
C -1.904511 -3.617889 1.457497
C 3.280736 0.693525 1.192402
C 2.276272 -2.298254 0.681796
C -0.802511 -4.545619 0.944508
C 3.268507 -1.406959 -0.054118
C -4.331072 1.834907 -0.451417
C -0.524670 4.414322 -1.007255
C 1.547102 -5.052730 -1.012026
C 3.175082 0.729779 -1.249216
C -4.299674 -1.146805 -1.254794
C 0.897151 -6.373317 -1.447624
C -1.196070 3.382106 -1.919849
C -1.032442 -3.300747 -1.936133
C 2.588183 -4.612486 -2.045230
C 2.094737 0.568110 -2.311552
C -2.127624 -4.353708 -2.150161
C -2.284539 4.055848 -2.764174
C 2.420415 4.127632 -2.913049
C -0.334826 -2.995382 -3.266585
C -1.998390 0.530841 -3.868056
C 1.422105 3.420890 -3.834301
C -0.911280 1.463727 -4.401910
C 0.798978 4.426058 -4.811792
C 0.003673 0.725947 -5.384944
H 1.736345 2.007168 7.016705
H 0.252099 2.596471 6.265937
H 0.431482 0.881290 6.651145
H -1.722093 0.497585 5.850367
H 3.405482 0.602812 5.701533
H 2.156895 2.408542 4.637169
H -2.870559 -0.494220 4.953006
H 0.056945 -1.297916 5.261853
H -2.689513 1.218113 4.564785
H 2.143633 -0.572013 5.337584

H -1.218182 3.095000 4.506617
H 3.081115 0.143067 4.029460
H -1.114867 -2.193529 4.300311
H 0.933613 4.319385 4.241394
H 0.414235 -1.679368 3.573565
H -1.542375 -0.224707 2.874447
H -0.377662 5.209061 3.466812
H -2.519815 2.290213 2.535977
H -0.510248 -5.478500 2.894659
H -3.958570 0.382712 2.470489
H 2.646257 2.230468 2.524518
H 0.634210 -4.181227 2.552451
H -2.359220 4.050357 2.485253
H 0.757128 4.287543 2.482038
H 3.392000 -0.050403 1.985284
H -2.492204 -4.096752 2.252857
H -3.863271 -1.332092 2.026705
H 2.243995 -2.003258 1.743070
H -1.480488 -2.698250 1.875590
H 0.827410 -5.760110 1.779330
H -1.352665 3.030785 1.445611
H -5.326507 -0.401915 1.675245
H 4.270629 1.144482 1.013986
H 2.161632 2.500920 0.865565
H 2.673466 -3.323550 0.676544
H -2.602625 -3.324532 0.666677
H 4.288703 -1.486092 0.355623
H -3.943599 2.626189 0.197030
H -1.283705 -5.431573 0.505085
H -1.249384 4.847056 -0.305032
H 2.090079 -5.262656 -0.078395
H -5.418297 1.792239 -0.311939
H 0.289738 3.982684 -0.415801
H 3.303938 -1.723343 -1.100040
H 0.171710 -6.750593 -0.719155
H -4.041354 -2.171288 -0.971021
H 3.250239 1.790301 -0.996517
H -1.682521 2.634027 -1.276570
H -4.140616 2.117350 -1.490346
H -5.394013 -1.069817 -1.253159
H -0.109384 5.249020 -1.583701
H -2.704024 -4.560284 -1.243073
H 4.162858 0.399076 -1.609151
H -1.529667 -2.376764 -1.599600
H 3.114487 -3.701149 -1.748683
H 1.658795 -7.153882 -1.579921
H -3.013867 4.566085 -2.120705
H -3.953154 -0.975420 -2.277436
H 3.348289 -5.393642 -2.184893
H 2.012917 -0.502162 -2.569805
H 0.378376 -6.270731 -2.407392
H 2.888986 3.444620 -2.198876
H 1.942949 4.922834 -2.332602
H -1.712075 -5.307332 -2.495858
H 0.472521 -2.263250 -3.158551
H 2.139843 -4.424323 -3.025606
H -2.838317 -4.019266 -2.917472
H -2.840900 3.346452 -3.384673
H -1.563597 -0.271241 -3.262482
H -2.715485 1.058369 -3.231345
H 2.459338 1.051551 -3.228646
H -1.867511 4.814963 -3.435848
H 0.097578 -3.901591 -3.705683
H 3.226672 4.594656 -3.495749
H -1.046808 -2.596736 -4.000194
H 0.236465 5.207348 -4.288103
H -2.563410 0.063289 -4.686807
H 1.995305 2.706783 -4.444423
H 0.556469 -0.082883 -4.892571
H -1.404884 2.279446 -4.951526
H 0.117488 3.952125 -5.525840
H 1.578376 4.934185 -5.396563
H 0.738180 1.389280 -5.855122
H -0.578290 0.265889 -6.195764

Table S23. Final coordinates for TS 3-II Benzene

Np 0.142450 0.038341 0.000381
Si 0.039776 2.168715 -3.018372
Si 0.084090 -3.743799 -0.387934
Si 0.376184 1.388235 3.541034
Si -3.643757 0.751999 -0.072742
N 0.869493 -2.165569 0.015914
N 1.018974 1.051607 1.890358
N 2.823121 -0.013820 -0.077582
N 0.882940 1.050319 -1.904758
N -1.874170 0.895811 -0.328048
C 0.552265 1.022647 -5.670625
C -2.725427 3.112993 -2.954825
C -1.690631 3.236511 2.717933
C 2.797887 0.308029 4.913443
C -4.545326 -0.040161 -1.568605
C 0.457713 4.619523 -4.489556
C -1.246071 3.969315 -1.109431
C -1.510969 2.838103 -2.086914
C 1.957734 4.354660 -2.476943
C 1.209018 3.627035 -3.594800
C -1.351735 0.037738 -4.362548
C -0.563673 1.312881 -4.661141
C -1.812527 -3.613634 1.787272
C -0.294393 -1.308634 4.362807
C 1.233967 1.698056 6.299150
C 1.797649 1.467276 4.890294
C -1.959035 0.442515 5.037227

C -0.939331 0.024825 3.970409
C -0.721850 -3.382036 -3.146804
C -2.453180 -4.434222 -1.675073
C -1.294080 -3.430847 -1.725549
C 0.710838 -6.421587 -1.312899
C 2.341363 -4.671658 -2.093942
C 1.382839 -5.083937 -0.972686
C 0.260629 -4.959392 2.252810
C -0.754090 -4.536574 1.185657
C 2.218347 -2.304349 0.596048
C 3.213936 -1.433031 -0.157672
C 0.493043 4.289469 3.377666
C -0.480001 3.136648 3.646009
C 2.328483 1.673360 1.630220
C 3.320304 0.623206 1.155163
C 3.240440 0.738725 -1.273816
C 2.203706 0.608969 -2.379855
C -3.988503 -0.377931 1.433478
C -4.551442 2.393642 0.316991
H 2.045543 1.903391 7.010785
H 0.542369 2.545296 6.347415
H 0.702954 0.814769 6.671879
H -1.483539 0.602377 6.011137
H 3.609366 0.511954 5.626174
H 2.366279 2.372779 4.631417
H -2.714485 -0.342262 5.178917
H 0.206304 -1.230716 5.334083
H -2.490794 1.363585 4.782023
H 2.331307 -0.632152 5.223110
H -0.835798 3.231609 4.682763
H 3.261871 0.133318 3.939399
H -1.047703 -2.100920 4.457946
H 1.340342 4.298436 4.072273
H 0.450153 -1.643101 3.632555
H -1.497277 -0.131873 3.033916
H -0.012603 5.260156 3.475369
H -2.463314 2.501917 2.966728
H -0.243158 -5.416213 3.115868
H -3.699291 0.100593 2.373577
H 2.760751 2.168215 2.511895
H 0.826289 -4.100430 2.631930
H -2.155229 4.231103 2.775760
H 0.899324 4.242585 2.360331
H 3.399331 -0.148214 1.925242
H -2.296028 -4.068744 2.662492
H -3.484192 -1.345460 1.368526
H 2.229794 -2.023185 1.661780
H -1.365672 -2.670149 2.119878
H 0.984530 -5.691688 1.878764
H -1.406477 3.060336 1.676161
H -5.066380 -0.574987 1.482408
H 4.328559 1.040450 0.995481
H 2.254170 2.468067 0.868789
H 2.600132 -3.335130 0.570156
H -2.605684 -3.373513 1.071731
H 4.243850 -1.570892 0.212410
H -4.130186 2.894746 1.192900
H -1.262532 -5.443198 0.826113
H -2.115971 4.146680 -0.465405
H -5.602219 2.167234 0.537246
H -0.387980 3.768496 -0.461647
H 3.196688 -1.727141 -1.210881
H 0.065704 -6.787889 -0.507320
H -4.156796 -1.040765 -1.785529
H 3.316006 1.794065 -0.997725
H -1.724902 1.829274 -1.303082
H -4.539208 3.100824 -0.516369
H -5.615364 -0.138300 -1.348718
H -1.050893 4.911962 -1.640504
H -2.961822 -4.453585 -0.707065
H 4.238491 0.409718 -1.608430
H -1.709593 -2.436044 -1.497998
H 2.856175 -3.730040 -1.882786
H 1.466879 -7.197051 -1.497807
H -3.590801 3.421383 -2.356595
H -4.450454 0.562960 -2.476594
H 3.114622 -5.438140 -2.242538
H 2.178272 -0.435632 -2.731673
H 0.100476 -6.353772 -2.220910
H 2.485190 3.661111 -1.815218
H 1.282734 4.948326 -1.852335
H -2.112773 -5.454518 -1.884508
H 0.134790 -2.706316 -3.233580
H 1.824592 -4.550366 -3.050678
H -3.209258 -4.187886 -2.432790
H -3.025814 2.255128 -3.561786
H -0.698569 -0.719087 -3.918909
H -2.173223 0.202258 -3.657516
H 2.570888 1.194038 -3.237187
H -2.523810 3.942660 -3.648370
H -0.390584 -4.375249 -3.469658
H 2.704089 5.046861 -2.890864
H -1.481472 -3.051132 -3.866206
H -0.300924 5.176044 -3.925867
H -1.780574 -0.393647 -5.277526
H 1.964499 3.131835 -4.223168
H 1.280606 0.303746 -5.276830
H -1.247010 2.039737 -5.125008
H -0.050527 4.128814 -5.326733
H 1.144777 5.362232 -4.917786
H 1.101944 1.922959 -5.964990
H 0.140355 0.583826 -6.589986
H 1.995932 -5.264236 -0.077501

Table S24. Final coordinates for II Benzene

Np 0.173643 0.075124 -0.003188
Si 0.211843 2.269822 -3.077444
Si 0.056966 -3.685279 -0.497699
Si 0.400842 1.445939 3.547202
Si -3.775382 -0.002302 0.036131
N 0.843993 -2.126068 -0.040866
N 1.015881 1.157635 1.878582
N 2.838866 -0.005278 -0.061648
N 0.917859 1.079823 -1.920751
N -2.042850 0.026344 -0.410275
C 0.381847 0.621994 -5.454487
C -2.253588 3.935855 -2.961886
C -1.780604 3.166000 2.757960
C 2.906184 0.498740 4.873963
C -4.719926 -1.325341 -0.977047
C 1.040393 4.362501 -4.892306
C -0.834958 3.972874 -0.879706
C -1.071026 3.376252 -2.230500
C 2.272778 4.293304 -2.701663
C 1.602138 3.455024 -3.792090
C -1.831090 0.569632 -4.249377
C -0.619765 1.425970 -4.620290
C -1.860358 -3.609486 1.652955
C -0.135996 -1.301772 4.317844
C 1.284355 1.786728 6.289620
C 1.840805 1.598490 4.871295
C -1.842316 0.368493 5.093533
C -0.841262 0.018107 3.985655
C -0.736261 -3.041023 -3.216787
C -2.386455 -4.417955 -1.917422
C -1.310987 -3.328333 -1.825534
C 0.724408 -6.331342 -1.490725
C 2.320789 -4.534166 -2.222482
C 1.375439 -4.994860 -1.109478
C 0.190472 -4.997031 2.105455
C -0.801729 -4.524986 1.038052
C 2.161618 -2.277071 0.604331
C 3.214789 -1.430742 -0.099163
C 0.357459 4.340097 3.360731
C -0.549522 3.143045 3.664355
C 2.343804 1.738093 1.608168
C 3.321849 0.658110 1.163869
C 3.263663 0.704726 -1.282036
C 2.213987 0.534087 -2.369724
C -4.029243 -0.387692 1.886582
C -4.648380 1.666464 -0.313822
H 2.091974 2.044652 6.988482
H 0.537848 2.584904 6.352049
H 0.821076 0.868220 6.667400
H -1.344510 0.503398 6.060023
H 3.680835 0.715678 5.622896
H 2.351753 2.537001 4.609906
H -2.574389 -0.439904 5.222592
H 0.393232 -1.235768 5.275100
H -2.404494 1.284568 4.888987
H 2.488165 -0.481547 5.122369
H -0.890821 3.231016 4.706152
H 3.413081 0.401881 3.910996
H -0.857239 -2.123887 4.410194
H 1.210645 4.407879 4.044629
H 0.596432 -1.588308 3.555557
H -1.422928 -0.136514 3.063726
H -0.196177 5.285654 3.443121
H -2.517029 2.403839 3.031648
H -0.330735 -5.490066 2.937932
H -3.719483 0.442467 2.527844
H 2.782199 2.246402 2.478943
H 0.750818 -4.157714 2.533793
H -2.290551 4.138433 2.797156
H 0.754836 4.290347 2.339935
H 3.376189 -0.098289 1.951163
H -2.389475 -4.102260 2.480629
H -3.501902 -1.290964 2.204716
H 2.129277 -1.988647 1.669123
H -1.399594 -2.700962 2.056725
H 0.919835 -5.714931 1.713927
H -1.499317 2.987262 1.715042
H -5.098494 -0.553956 2.065412
H 4.339135 1.056205 1.011510
H 2.293431 2.514151 0.826390
H 2.521688 -3.315840 0.605622
H -2.612481 -3.295269 0.921908
H 4.217183 -1.576007 0.336067
H -4.169136 2.493066 0.220532
H -1.312798 -5.413601 0.639867
H -1.738050 3.911700 -0.256407
H -5.694000 1.619065 0.013913
H -0.011902 3.500319 -0.334439
H 3.260713 -1.740687 -1.146295
H 0.063644 -6.720638 -0.708769
H -4.412040 -2.339585 -0.704947
H 3.346424 1.769268 -1.046834
H -2.006309 0.146143 -1.430863
H -4.647839 1.904374 -1.382815
H -5.798102 -1.244495 -0.793463
H -0.598946 5.048555 -0.949722
H -2.870437 -4.618603 -0.956562
H 4.257996 0.355303 -1.607025
H -1.797538 -2.410206 -1.463872
H 2.823899 -3.593324 -1.983024
H 1.490677 -7.096570 -1.677016
H -3.183461 3.779258 -2.393043
H -4.556922 -1.201750 -2.053274

H 3.102433 -5.284762 -2.405131
H 2.131325 -0.535280 -2.622394
H 0.134066 -6.246554 -2.410429
H 2.689688 3.677187 -1.898786
H 1.561953 4.989412 -2.241896
H -1.976356 -5.368135 -2.278628
H 0.055598 -2.284301 -3.194525
H 1.793100 -4.385565 -3.169758
H -3.175170 -4.122996 -2.622629
H -2.400642 3.510971 -3.958561
H -1.539772 -0.271004 -3.610015
H -2.596934 1.145613 -3.718913
H 2.595447 1.015978 -3.280539
H -2.165031 5.028399 -3.084407
H -0.315031 -3.946228 -3.668833
H 3.093501 4.895930 -3.114205
H -1.517885 -2.682980 -3.899784
H 0.224292 4.993686 -4.519767
H -2.305246 0.140915 -5.143229
H 2.372069 2.820394 -4.253544
H 0.764817 -0.239311 -4.895598
H -0.976128 2.261625 -5.240424
H 0.656044 3.797527 -5.747884
H 1.818089 5.037747 -5.274857
H 1.243607 1.222266 -5.767801
H -0.088759 0.228943 -6.366015
H 1.994140 -5.191381 -0.221324

Table S25. Final coordinates for TS II-4a Benzene

Np 0.050220 0.157113 -0.033469
Si 0.229131 1.554159 3.498782
Si 0.064870 2.232624 -3.249950
Si 0.120205 -3.620084 -0.489948
Si -3.899699 -0.219363 0.205161
N 2.719917 0.158544 -0.082305
N 0.860038 1.284009 1.829615
N 0.777375 1.123268 -2.014080
N 0.795322 -2.011341 -0.028552
N -2.181536 -0.127420 -0.297865
C 0.631820 6.276282 -1.035241
C 0.944177 7.612511 -0.774565
C 0.458137 8.618378 -1.610324
C -0.339992 8.287263 -2.705310
C -0.653584 6.950397 -2.963682
C -0.165124 5.944555 -2.130548
C -1.322465 3.319720 -0.906481
C 2.489664 3.904780 -3.502679
C -2.082798 0.394669 -3.964497
C 0.797271 4.059672 -5.356173
C 1.435878 3.140896 -4.306283
C -0.141450 0.462177 -5.555604
C -1.003503 1.278773 -4.587177
C 1.100327 2.139525 6.202106
C -1.870369 0.256780 5.083673
C -2.269361 -4.519547 -1.902710
C 2.492227 -4.182745 -2.174939
C 0.333695 -4.992711 2.085557
C 0.981132 -6.149907 -1.605664
C 1.541366 -4.802862 -1.125519
C -1.765733 -3.663105 1.676348
C -0.681108 -4.528988 1.035893
C -0.157259 4.430279 3.354480
C 0.022411 -1.220047 4.347600
C -0.832723 -0.001093 3.983953
C -2.144985 3.028293 2.714186
C -0.917218 3.127089 3.618705
C 2.152019 1.926672 1.532810
C 3.179562 0.881062 1.118739
C 2.107209 -2.105470 0.643298
C 3.145418 -1.253991 -0.072553
C 3.122370 0.839880 -1.325016
C 2.092398 0.583026 -2.414177
C 2.817785 0.907824 4.850434
C 1.661218 1.910356 4.791004
C -2.346353 3.926492 -3.136606
C -1.097690 3.510486 -2.386305
C -0.664910 -3.114764 -3.235723
C -1.249487 -3.375733 -1.843160
C -4.115137 -0.681566 2.042370
C -4.821644 -1.527813 -0.847706
C -4.829749 1.434178 -0.065692
H -0.470270 4.773081 -2.330447
H 1.015793 5.497207 -0.380935
H 1.564935 7.868240 0.080976
H 0.700230 9.658540 -1.407785
H -0.720957 9.069884 -3.357279
H -1.282658 6.702031 -3.815396
H 1.891159 2.493495 6.877678
H 0.299296 2.885302 6.227022
H 0.705125 1.214131 6.636380
H -1.393301 0.495319 6.040558
H 3.583774 1.248118 5.561215
H 2.087414 2.870894 4.464972
H -2.489377 -0.635193 5.248125
H 0.516840 -1.082896 5.315955
H -2.548680 1.081813 4.846251
H 2.490185 -0.081600 5.183789
H -1.268349 3.147468 4.660853
H 3.313432 0.778784 3.885155
H -0.595123 -2.123431 4.433301
H 0.672290 4.581851 4.053777
H 0.801132 -1.419983 3.604417
H -1.390014 -0.252903 3.067593

H -0.820580 5.301133 3.447352
H -2.740121 2.131799 2.915300
H -0.168567 -5.514410 2.912279
H -3.808813 0.124194 2.714992
H 2.565265 2.487942 2.382773
H 0.877973 -4.149661 2.526831
H -2.808136 3.894721 2.842981
H 0.257598 4.452406 2.339846
H 3.271817 0.154892 1.930804
H -2.277766 -4.192733 2.491879
H -3.569429 -1.588326 2.315675
H 2.046178 -1.780767 1.695165
H -1.332154 -2.749814 2.098466
H 1.075559 -5.686506 1.674720
H -1.855158 2.999804 1.659523
H -5.179784 -0.868987 2.228430
H 4.175905 1.321091 0.946528
H 2.056733 2.670220 0.722980
H 2.491756 -3.133986 0.686714
H -2.525978 -3.352614 0.952622
H 4.144597 -1.350679 0.382788
H -4.386432 2.255647 0.505934
H -1.162859 -5.426991 0.622122
H -1.924167 2.418050 -0.713281
H -5.873560 1.332809 0.255630
H -0.379344 3.225111 -0.355949
H 3.217179 -1.598989 -1.106735
H 0.335268 -6.626835 -0.860052
H -4.471353 -2.543353 -0.638255
H 3.151055 1.915565 -1.128469
H -2.198686 -0.165355 -1.324788
H -4.838493 1.726428 -1.121206
H -5.896786 -1.495329 -0.634385
H -1.871138 4.164204 -0.469683
H -2.761646 -4.696631 -0.941585
H 4.135210 0.525135 -1.627952
H -1.787032 -2.472123 -1.520581
H 3.502525 -4.044299 -1.773963
H 1.795916 -6.853927 -1.823020
H -3.106143 3.132380 -3.118657
H -4.693593 -1.343796 -1.920304
H 2.592363 -4.833995 -3.051794
H 2.042506 -0.500294 -2.610782
H 0.398469 -6.043490 -2.527225
H 3.000931 3.269946 -2.773417
H 2.049828 4.745066 -2.957487
H -1.809148 -5.464807 -2.211151
H 0.026999 -2.265978 -3.244148
H 2.159215 -3.208520 -2.545268
H -3.057616 -4.295874 -2.634220
H -2.154577 4.159418 -4.189064
H -1.631181 -0.419472 -3.386496
H -2.748762 0.954609 -3.299676
H 2.477099 1.022436 -3.344113
H -2.809745 4.809027 -2.676061
H -0.117551 -3.985733 -3.614255
H 3.258575 4.320699 -4.168419
H -1.459268 -2.896670 -3.961945
H 0.276854 4.902805 -4.889566
H -2.711177 -0.072964 -4.735033
H 1.953325 2.342152 -4.857108
H 0.426633 -0.316546 -5.032822
H -1.506628 2.066832 -5.165616
H 0.081558 3.535718 -5.998552
H 1.567108 4.487492 -6.012846
H 0.575230 1.081618 -6.104261
H -0.766228 -0.046438 -6.302754
H 2.136312 -5.026899 -0.230326

Table S26. Final coordinates for 4a Benzene

Np -0.095286 -0.510805 -0.149206
Si -0.117991 -4.302531 -0.506063
Si -4.050704 -0.791158 0.069369
Si 0.090504 0.992161 3.348144
Si -0.048805 1.559766 -3.358765
N -2.327690 -0.773553 -0.425855
N 0.618876 -2.695809 -0.131515
N 0.730951 0.633416 1.699707
N 2.573730 -0.548683 -0.204321
N 0.642470 0.430511 -2.129256
C 0.705681 3.471114 -5.369105
C -0.145211 -0.169913 -5.683719
C 0.633374 -6.967288 -1.378514
C -1.998958 -4.180349 1.671278
C -0.244909 -1.758790 4.235310
C -2.256825 2.494613 2.530931
C 2.605395 0.259436 4.770315
C -0.182822 3.851273 2.968364
C -0.989933 2.615414 3.377671
C 0.993325 1.721744 6.016433
C 1.532472 1.344018 4.629975
C -2.428321 3.269910 -3.273932
C -2.144659 -0.296317 -4.165613
C -1.059210 0.616165 -4.736998
C -0.959869 -3.881593 -3.260143
C 0.092950 -5.495779 2.152956
C -0.926924 -5.096291 1.081836
C -2.551853 -5.203993 -1.838461
C -1.507273 -4.080950 -1.842562
C 2.169295 -5.156917 -2.202615
C 1.243736 -5.594791 -1.064202
C -5.008952 -2.115556 -0.929641
C -4.929228 0.877424 -0.273071
C -4.288996 -1.166622 1.924323

C 2.979096 -1.965950 -0.175528
C 1.927966 -2.797493 0.544777
C -2.027404 -0.168766 5.006969
C -1.037776 -0.495980 3.881843
C 2.032578 1.250094 1.386642
C 3.043844 0.183957 0.986359
C 2.985687 0.108082 -1.457547
C 1.943088 -0.137164 -2.538111
C 2.256090 3.360646 -3.395456
C 1.339419 2.551560 -4.316485
C -1.412765 2.677655 -1.040680
C -1.136929 2.936976 -2.518886
C 0.595794 9.179364 -0.404533
C -0.253742 10.047050 -1.092964
C -1.024468 9.589591 -2.163199
C -0.952532 8.246444 -2.560753
C -0.097600 7.439521 -1.845152
C 0.684786 7.831773 -0.783054
H -0.498977 3.831323 -2.570698
H 1.340821 7.143601 -0.256493
H 1.192232 9.544454 0.428363
H -0.315970 11.089402 -0.792561
H -1.684219 10.272925 -2.692952
H -1.546607 7.874938 -3.391590
H 1.811030 2.052153 6.671767
H 0.260046 2.533867 5.982676
H 0.517467 0.867726 6.511572
H -1.512061 0.014847 5.956314
H 3.442976 0.620755 5.382913
H 2.032060 2.240943 4.234295
H -2.713494 -1.009606 5.175482
H 0.287245 -1.638145 5.185411
H -2.640812 0.712683 4.797065
H 2.215716 -0.637610 5.261849
H -1.297881 2.744781 4.425364
H 3.019705 -0.054350 3.808901
H -0.911685 -2.622391 4.353753
H 0.688266 4.014404 3.612689
H 0.497197 -2.014502 3.471649
H -1.632962 -0.719645 2.982427
H -0.797203 4.760729 3.019182
H -2.908087 1.683024 2.869668
H -0.404224 -5.963574 3.014256
H -3.990234 -0.329699 2.561493
H 2.460395 1.816664 2.225590
H 0.638870 -4.625570 2.535360
H -2.847777 3.420417 2.562730
H 0.179817 3.766787 1.937004
H 3.127344 -0.532083 1.808281
H -2.491288 -4.641961 2.538827
H -3.749346 -2.060591 2.246532
H 1.870160 -2.459280 1.593324
H -1.559651 -3.234822 2.007591
H 0.833579 -6.212926 1.781948
H -2.015734 2.304328 1.480902
H -5.356309 -1.344290 2.104944
H 4.046105 0.607698 0.807747
H 1.945732 1.983180 0.566314
H 2.302407 -3.829062 0.601567
H -2.776771 -3.932249 0.942349
H 3.975399 -2.072502 0.283703
H -4.459520 1.707155 0.264562
H -1.420514 -6.015584 0.733511
H -2.013740 1.772092 -0.890151
H -5.974731 0.820072 0.053905
H -0.482274 2.581507 -0.469076
H 3.051238 -2.319370 -1.207149
H -0.009219 -7.339659 -0.573565
H -4.700510 -3.131107 -0.662461
H 3.041936 1.184278 -1.273032
H -2.338700 -0.837624 -1.451874
H -4.931249 1.126402 -1.339589
H -6.084433 -2.032449 -0.732296
H -1.971989 3.509935 -0.592951
H -3.028788 -5.336773 -0.862730
H 3.989307 -0.233772 -1.761851
H -2.017245 -3.155465 -1.538328
H 2.636348 -4.186322 -2.014242
H 1.422136 -7.716103 -1.535620
H -3.142664 2.439674 -3.254338
H -4.861827 -1.993271 -2.008499
H 2.978771 -5.885661 -2.348924
H 1.870786 -1.220403 -2.727449
H 0.033882 -6.942671 -2.295578
H 2.766941 2.733348 -2.659422
H 1.700400 4.126210 -2.840048
H -2.117184 -6.169378 -2.121385
H -0.188330 -3.105944 -3.308340
H 1.634697 -5.078244 -3.154333
H -3.349993 -4.990778 -2.562487
H -2.246002 3.517653 -4.325120
H -1.701800 -1.099048 -3.565142
H -2.856338 0.246408 -3.534666
H 2.328522 0.290420 -3.474239
H -2.930292 4.134881 -2.819621
H -0.521835 -4.806651 -3.652249
H 3.030449 3.886040 -3.971245
H -1.761119 -3.594697 -3.954075
H 0.076973 4.241297 -4.906173
H -2.723131 -0.778344 -4.965946
H 1.961263 1.823517 -4.855533
H 0.409191 -0.951458 -5.150435
H -1.555215 1.404223 -5.322134
H 0.086347 2.925757 -6.089021

H 1.480500 3.998197 -5.942627
H 0.589200 0.470225 -6.184125
H -0.726980 -0.670475 -6.470054
H 1.872458 -5.731407 -0.171955

Table S27. Final coordinates for TS(b) Benzene

Np -0.318016 -0.268908 0.711889
Si 1.690468 -3.231987 -0.739762
Si -0.877581 -0.177523 4.475341
Si -1.116079 3.045880 -1.167183
Si -3.141544 -1.616387 -1.390168
N 1.414083 -1.763387 0.282614
N 2.049407 0.932930 1.209895
N -0.166866 0.396432 2.907674
N -0.139457 1.629508 -0.593658
N -2.128341 -1.370791 0.093487
C 0.270305 -4.505233 -2.958780
C 4.285061 -2.274067 -1.886328
C -2.106187 2.434761 4.931872
C -3.284482 2.258738 0.611276
C -0.889705 4.793737 1.185988
C 0.425527 3.838879 -3.595260
C -0.837099 5.994116 -1.005453
C -0.480576 4.686075 -0.286156
C -1.656183 2.535885 -4.053008
C -0.975847 3.493592 -3.075027
C -0.208260 -4.880593 0.677852
C 3.791718 -4.723902 -2.070530
C 3.581631 -3.460821 -1.223019
C 2.185638 -5.129356 1.411779
C 1.251515 -4.867191 0.226889
C 2.570315 -1.459384 1.150023
C 3.090742 -0.055601 0.882555
C -3.381906 -3.456428 -1.848898
C -4.895399 -0.857204 -1.278955
C 1.084564 -2.170873 -3.383690
C 0.565499 -3.142917 -2.317963
C 1.824879 -0.547243 5.695442
C 0.298788 0.968882 6.977228
C 0.361096 -0.213953 6.004335
C -0.299135 -2.985468 4.095965
C -2.312952 -2.469278 5.495897
C -1.465710 -2.013039 4.301279
C -3.641025 0.625225 4.146529
C -2.398999 0.931559 4.984783
C 1.290012 1.831062 -0.927116
C 2.109790 2.106470 0.323684
C 2.094052 1.302132 2.632519
C 0.688303 1.577421 3.132059
C -3.898804 3.875104 -1.214644
C -2.990431 2.703675 -0.822372
C -2.282729 -0.733746 -2.843270
C -3.766859 -2.649823 1.590672
C -3.645099 -4.024749 1.786896
C -4.660918 -4.726540 2.441741
C -5.798599 -4.055711 2.890336
C -5.921553 -2.681770 2.684356
C -4.906051 -1.977820 2.031057
H -5.009251 -0.909585 1.865912
H -6.809209 -2.156124 3.028195
H -6.589828 -4.603277 3.395968
H -4.564186 -5.798679 2.595869
H -2.766225 -4.553100 1.431624
H 0.887321 0.752556 7.879916
H -0.721014 1.192102 7.305241
H 0.717570 1.884431 6.542132
H -2.615856 0.659140 6.029054
H -1.255937 2.732294 5.552481
H -0.050522 -1.077907 6.549244
H -2.975996 3.012257 5.274175
H -1.732928 -2.472342 6.427570
H 2.346710 -0.871534 6.606491
H -3.192957 -1.840333 5.660478
H -4.491479 1.245860 4.462170
H -2.673473 -3.494378 5.342050
H -1.893486 2.753832 3.905565
H 2.362586 0.332667 5.326885
H -3.954977 -0.419397 4.216504
H 1.940969 -1.343870 4.954668
H 0.336026 -3.042932 4.987882
H 0.755916 1.866729 4.191676
H -3.461092 0.843498 3.088131
H -2.106980 -2.058158 3.411244
H -0.666364 -4.001765 3.904028
H -4.881372 0.150599 -0.854016
H -4.341831 1.986328 0.729857
H 0.289510 2.462385 2.617376
H 0.342198 -2.703884 3.253458
H 2.496302 0.453398 3.189038
H -1.212179 -0.932785 -2.903463
H -2.698487 1.377844 0.899436
H 2.769668 2.158780 2.791889
H -3.074718 3.048828 1.338064
H -5.311404 -0.785707 -2.291652
H -5.572237 -1.474355 -0.682252
H -2.419420 0.343600 -2.756829
H 2.305749 -1.539528 2.217832
H -4.956225 3.594099 -1.118330
H -0.407579 -4.061341 1.375523
H 2.123867 -4.328755 2.158446
H -2.740876 -1.056831 -3.785668
H -3.241277 1.861514 -1.482043
H -1.949972 5.053179 1.286295

H 1.667341 2.958256 0.847547
H -3.740830 4.745089 -0.567266
H -0.458898 -5.820384 1.190054
H 4.016898 0.161575 1.440648
H 1.919014 -6.063882 1.924842
H -0.729291 3.862802 1.737272
H 3.407146 -2.158606 1.016248
H -0.902700 -4.763346 -0.160406
H -3.745579 4.200659 -2.248940
H -0.321802 5.582555 1.698566
H 3.234555 -5.218348 1.108422
H 3.156819 2.362706 0.090232
H -3.976972 -3.517918 -2.768546
H 3.316958 0.031312 -0.182719
H -1.920474 6.146609 -1.069372
H -3.919740 -4.001125 -1.068112
H -2.696449 2.326304 -3.783689
H -2.432083 -3.965966 -2.032749
H 1.390581 -5.683539 -0.496707
H 1.454043 2.659374 -1.627270
H -0.386698 -2.749639 -1.936011
H 1.703966 0.946265 -1.436933
H 0.615935 4.596729 -0.317328
H 4.093609 -3.641159 -0.266548
H -1.126788 1.579793 -4.117706
H -0.428490 6.853687 -0.455673
H -0.437099 6.047070 -2.021921
H -1.557334 4.428220 -3.095509
H 4.218963 -1.359050 -1.293343
H -0.157717 -5.223613 -2.253164
H 1.323601 -1.182052 -2.976990
H -1.668150 2.964879 -5.065003
H 3.343531 -5.617455 -1.624082
H 1.030073 2.937320 -3.746991
H -0.444241 -4.395745 -3.785687
H 5.353125 -2.489889 -2.029590
H 0.343930 -2.028829 -4.182238
H 0.985159 4.504534 -2.928829
H 3.870159 -2.050706 -2.873623
H 1.172582 -4.962255 -3.379798
H 1.990243 -2.556044 -3.865075
H 4.863851 -4.928380 -2.197711
H 3.370775 -4.610776 -3.075988
H 0.360362 4.340605 -4.570689
H -2.895494 -2.031701 0.986332

Table S28. Final coordinates for TS 3-II Toluene

Np 0.141278 0.035983 0.000866
Si 0.037909 2.169920 -3.017575
Si 0.086081 -3.745520 -0.386873
Si 0.376522 1.387758 3.540755
Si -3.644646 0.754225 -0.074333
N 0.871935 -2.167474 0.015687
N 1.019519 1.050567 1.890554
N 2.822921 -0.014052 -0.078634
N 0.879917 1.050724 -1.904171
N -1.875017 0.899545 -0.326812
C 0.549717 1.023260 -5.669630
C -2.726863 3.115548 -2.954058
C -1.688102 3.239081 2.717136
C 2.797216 0.305813 4.913018
C -4.543942 -0.038074 -1.571602
C 0.456847 4.619520 -4.489621
C -1.246541 3.972531 -1.109560
C -1.512157 2.840926 -2.086423
C 1.957387 4.354952 -2.477473
C 1.208190 3.627056 -3.594846
C -1.352985 0.037869 -4.360184
C -0.566072 1.313437 -4.659969
C -1.811463 -3.614051 1.787741
C -0.295395 -1.308390 4.363729
C 1.233293 1.695530 6.299229
C 1.797523 1.465555 4.890448
C -1.960590 0.443508 5.035096
C -0.939746 0.024910 3.969764
C -0.719052 -3.382042 -3.145679
C -2.450960 -4.435067 -1.675166
C -1.291752 -3.431797 -1.724639
C 0.711390 -6.423644 -1.310967
C 2.343192 -4.674725 -2.091910
C 1.384210 -5.086409 -0.970836
C 0.261447 -4.959670 2.254390
C -0.752956 -4.537346 1.186726
C 2.221372 -2.305507 0.594471
C 3.215380 -1.432736 -0.159607
C 0.496003 4.288874 3.379495
C -0.478460 3.136858 3.646250
C 2.328801 1.672590 1.630156
C 3.320620 0.623020 1.153833
C 3.238086 0.739424 -1.274984
C 2.200305 0.608358 -2.379840
C -3.991197 -0.377501 1.430143
C -4.553901 2.394995 0.315650
H 2.044492 1.900980 7.011267
H 0.541231 2.542404 6.347503
H 0.702496 0.811834 6.671322
H -1.485967 0.604618 6.009233
H 3.609019 0.509177 5.625550
H 2.366636 2.370964 4.632370
H -2.716011 -0.341275 5.176973
H 0.204718 -1.229735 5.335239
H -2.492231 1.364169 4.778225
H 2.330166 -0.634162 5.222645

H -0.835055 3.231382 4.682768
H 3.260713 0.131094 3.938725
H -1.048995 -2.100415 4.458977
H 1.343382 4.295787 4.074011
H 0.449593 -1.643650 3.634252
H -1.496543 -0.132342 3.032664
H -0.008462 5.260054 3.478485
H -2.461510 2.504479 2.963603
H -0.242428 -5.417681 3.116766
H -3.698805 0.097759 2.370909
H 2.761583 2.166590 2.512092
H 0.825738 -4.100171 2.634386
H -2.152154 4.233860 2.776353
H 0.902093 4.242747 2.362032
H 3.400718 -0.148546 1.923683
H -2.294902 -4.068582 2.663297
H -3.490443 -1.346706 1.362104
H 2.233676 -2.025035 1.660405
H -1.364670 -2.670355 2.119901
H 0.986586 -5.690754 1.880384
H -1.402924 3.064848 1.675311
H -5.069694 -0.570856 1.480147
H 4.328490 1.040888 0.993433
H 2.254001 2.467973 0.869472
H 2.604037 -3.335938 0.567440
H -2.604642 -3.374414 1.072054
H 4.245649 -1.569471 0.209853
H -4.133488 2.895690 1.192238
H -1.261396 -5.444075 0.827440
H -2.116533 4.151134 -0.465939
H -5.604753 2.167925 0.534853
H -0.388929 3.771053 -0.461312
H 3.197904 -1.726563 -1.212920
H 0.067173 -6.790067 -0.504698
H -4.154423 -1.038448 -1.787926
H 3.312107 1.794772 -0.998591
H -1.725712 1.832972 -1.302021
H -4.541166 3.102731 -0.517238
H -5.614219 -0.136812 -1.353146
H -1.050043 4.914641 -1.641093
H -2.960394 -4.454280 -0.707558
H 4.236303 0.412065 -1.610648
H -1.707239 -2.437063 -1.496481
H 2.860036 -3.734475 -1.879579
H 1.466975 -7.199218 -1.497288
H -3.592367 3.422948 -2.355508
H -4.448011 0.565218 -2.479371
H 3.114780 -5.442688 -2.241635
H 2.174359 -0.436878 -2.729830
H 0.099842 -6.355050 -2.218131
H 2.485528 3.661660 -1.816019
H 1.282562 4.948463 -1.852529
H -2.110312 -5.455359 -1.884202
H 0.137678 -2.706301 -3.231758
H 1.826425 -4.551169 -3.048363
H -3.206318 -4.188728 -2.433606
H -3.026717 2.257812 -3.561484
H -0.699839 -0.716878 -3.912849
H -2.176574 0.203270 -3.657821
H 2.566636 1.191776 -3.238631
H -2.525836 3.945778 -3.647110
H -0.387773 -4.375121 -3.468949
H 2.703257 5.047334 -2.891989
H -1.478438 -3.050604 -3.865088
H -0.301442 5.176285 -3.925703
H -1.778797 -0.396489 -5.275185
H 1.963271 3.131260 -4.223190
H 1.278176 0.304477 -5.275821
H -1.250129 2.039540 -5.123910
H -0.051909 4.128668 -5.326411
H 1.143968 5.361929 -4.918298
H 1.099269 1.923624 -5.964056
H 0.137688 0.584313 -6.588884
H 1.997013 -5.266722 -0.075460

Table S29. Final coordinates for II Toluene

Np 0.173317 0.074565 -0.004152
Si 0.212320 2.269960 -3.077690
Si 0.057886 -3.685906 -0.496900
Si 0.400201 1.445555 3.546787
Si -3.776287 -0.001042 0.034924
N 0.845775 -2.127158 -0.040680
N 1.015843 1.157567 1.878677
N 2.839385 -0.005680 -0.061840
N 0.918783 1.080118 -1.921539
N -2.044377 0.026300 -0.412750
C 0.380833 0.623174 -5.455203
C -2.252798 3.936322 -2.959914
C -1.783208 3.164027 2.758323
C 2.905465 0.499973 4.874694
C -4.722701 -1.323304 -0.977597
C 1.039344 4.363233 -4.892441
C -0.832412 3.972713 -0.878840
C -1.069808 3.376318 -2.229524
C 2.273878 4.292884 -2.703029
C 1.602089 3.455180 -3.793203
C -1.831469 0.570120 -4.249009
C -0.620457 1.426765 -4.620237
C -1.859958 -3.609352 1.653536
C -0.134247 -1.302596 4.316925
C 1.283168 1.788468 6.289342
C 1.839764 1.599426 4.871200
C -1.842151 0.365989 5.092910
C -0.840795 0.016679 3.984983

C -0.735155 -3.041304 -3.215927
C -2.385943 -4.417710 -1.916700
C -1.310041 -3.328521 -1.824702
C 0.722989 -6.332150 -1.490685
C 2.321095 -4.536117 -2.221480
C 1.375215 -4.996457 -1.108766
C 0.191194 -4.996150 2.106663
C -0.801181 -4.524975 1.039030
C 2.163710 -2.278150 0.603750
C 3.216122 -1.430913 -0.099749
C 0.354694 4.339306 3.359297
C -0.551519 3.141913 3.663955
C 2.343868 1.737676 1.608182
C 3.321915 0.657616 1.163929
C 3.264396 0.704884 -1.281877
C 2.215207 0.534757 -2.370130
C -4.029050 -0.386809 1.885500
C -4.648145 1.668754 -0.313170
H 2.090718 2.046791 6.988145
H 0.536609 2.586652 6.351172
H 0.819832 0.870157 6.667535
H -1.344364 0.501465 6.059331
H 3.680965 0.718649 5.622249
H 2.350470 2.537871 4.609096
H -2.573338 -0.443219 5.222033
H 0.394657 -1.236334 5.274341
H -2.405323 1.281450 4.888315
H 2.487948 -0.479893 5.125643
H -0.892196 3.229974 4.705940
H 3.411129 0.401328 3.911252
H -0.854682 -2.125492 4.408748
H 1.208363 4.407646 4.042537
H 0.598772 -1.588045 3.554771
H -1.422255 -0.138302 3.062986
H -0.199321 5.284649 3.441670
H -2.518933 2.401382 3.032555
H -0.329815 -5.488674 2.939574
H -3.717241 0.442588 2.526764
H 2.782399 2.246009 2.478886
H 0.751447 -4.156363 2.534219
H -2.293725 4.136155 2.797843
H 0.751372 4.289243 2.338223
H 3.376123 -0.098901 1.951130
H -2.388946 -4.101802 2.481486
H -3.502814 -1.291044 2.202787
H 2.131753 -1.990324 1.668724
H -1.399367 -2.700564 2.056970
H 0.920649 -5.714145 1.715483
H -1.502502 2.985590 1.715178
H -5.098416 -0.551369 2.065198
H 4.339196 1.055797 1.011847
H 2.293696 2.513663 0.826277
H 2.524264 -3.316775 0.604183
H -2.612182 -3.295614 0.922374
H 4.218686 -1.575642 0.335245
H -4.167591 2.494251 0.221796
H -1.312066 -5.413893 0.641268
H -1.735304 3.912649 -0.255114
H -5.693677 1.622196 0.014968
H -0.009583 3.499190 -0.334034
H 3.262021 -1.740560 -1.146991
H 0.061682 -6.721082 -0.709000
H -4.415032 -2.337690 -0.705679
H 3.347114 1.769326 -1.046179
H -2.008182 0.142846 -1.433672
H -4.647538 1.907703 -1.381940
H -5.800672 -1.241865 -0.793098
H -0.595133 5.048103 -0.949088
H -2.869729 -4.618407 -0.955750
H 4.258804 0.355611 -1.606724
H -1.796382 -2.410265 -1.463052
H 2.825277 -3.596008 -1.981373
H 1.488558 -7.098040 -1.677169
H -3.182303 3.779630 -2.390482
H -4.560407 -1.199755 -2.053942
H 3.101854 -5.287528 -2.404611
H 2.132885 -0.534500 -2.623482
H 0.132872 -6.246330 -2.410446
H 2.691108 3.676362 -1.900608
H 1.563622 4.989161 -2.242617
H -1.976239 -5.367909 -2.278293
H 0.056988 -2.284854 -3.193599
H 1.793534 -4.386319 -3.168656
H -3.174699 -4.122162 -2.621625
H -2.400510 3.511695 -3.956614
H -1.539540 -0.271196 -3.610803
H -2.596707 1.145572 -3.717096
H 2.597045 1.017276 -3.280465
H -2.164151 5.028890 -3.082122
H -0.314133 -3.946643 -3.667908
H 3.094535 4.895290 -3.116047
H -1.516638 -2.682975 -3.898950
H 0.223643 4.994251 -4.518726
H -2.306512 0.142334 -5.142843
H 2.371407 2.820522 -4.255640
H 0.764155 -0.238226 -4.896653
H -0.977252 2.262687 -5.239764
H 0.654087 3.798651 -5.747864
H 1.816699 5.038627 -5.275443
H 1.242381 1.223679 -5.768650
H -0.090170 0.230332 -6.366624
H 1.993571 -5.193770 -0.220558

Table S30. Final coordinates for TS II-4a Toluene

Np -4.755503 -5.123509 -0.294619
Si -7.938902 -5.295053 -2.650916
Si -4.875541 -1.372895 -0.926920
Si -2.689026 -7.198158 -2.778716
Si -6.434160 -6.498060 2.845982
N -6.775240 -5.019395 -1.315376
N -4.079340 -2.936181 -0.484259
N -3.088921 -6.504947 -1.160186
N -2.515315 -4.783645 1.116986
N -5.179084 -5.563705 1.941970
C -3.621942 -6.586652 -5.493976
C -3.042636 -10.006314 -2.134838
C -6.412494 -8.166289 0.445017
C -8.655192 -4.857300 1.887242
C -0.487997 -8.202900 -4.369670
C -5.092207 -8.805681 -2.961086
C -3.571699 -8.912626 -3.068083
C 0.220315 -6.484211 -2.689727
C -0.780754 -7.605433 -2.985828
C -3.186022 -0.608719 -3.191985
C -3.954571 -0.026860 1.557454
C -7.625048 -0.519070 -1.422164
C -4.728538 1.462668 -0.316134
C -4.061541 0.126613 0.037956
C -7.144872 -1.359588 0.893480
C -6.782744 -1.493905 -0.589651
C -2.132387 -3.366776 0.978664
C -2.612451 -2.818703 -0.356879
C -4.493611 -7.705572 4.722557
C -6.927372 -7.781805 5.376384
C -5.839835 -6.982875 4.645101
C -8.908800 -6.936988 -2.455631
C -9.243077 -3.893111 -2.685584
C -7.096320 -5.350474 -4.360106
C -5.174185 -2.061131 -3.709354
C -4.630815 -0.947357 -2.813420
C -2.380346 -4.753611 -4.313440
C -3.318666 -5.951368 -4.131691
C -2.173192 -6.908201 -0.076543
C -1.502253 -5.688543 0.542143
C -2.830460 -5.126866 2.515697
C -4.281732 -4.784332 2.817441
C -7.836698 -4.329910 4.198044
C -8.053837 -5.441273 3.165419
C -8.404029 -8.489807 1.965107
C -6.945819 -8.083134 1.858033
C -5.813689 -10.408016 2.714618
C -8.310241 -13.143123 1.945830
C -7.868234 -13.591307 0.698873
C -6.746951 -12.996720 0.116812
C -6.078023 -11.966998 0.770482
C -6.507722 -11.499726 2.029797
C -7.641991 -12.114398 2.600943
H -7.991993 -11.777562 3.574280
H -9.178812 -13.602606 2.411287
H -8.389068 -14.397262 0.189169
H -6.389030 -13.341792 -0.850105
H -5.200223 -11.518057 0.312124
H -4.776783 -10.260339 2.406252
H -6.338818 -9.186501 2.405206
H 0.549366 -8.560942 -4.421360
H -1.134398 -9.051656 -4.614416
H -0.605806 -7.457740 -5.164489
H -2.714393 -6.976468 -5.968176
H 1.249517 -6.862981 -2.761785
H -0.591028 -8.404110 -2.253152
H -4.047840 -5.842963 -6.180769
H -1.451996 -5.053338 -4.812131
H -4.335234 -7.413766 -5.431581
H 0.135660 -5.657213 -3.401255
H -3.326840 -9.203125 -4.100150
H 0.104084 -6.065052 -1.687237
H -2.842208 -3.981699 -4.942191
H -1.968929 -10.182988 -2.261034
H -2.108931 -4.286975 -3.361013
H -4.270287 -5.567869 -3.731717
H -3.549261 -10.963402 -2.321301
H -5.511838 -8.095592 -3.680787
H -3.111741 -0.341068 -4.255302
H -6.461488 -6.232121 -4.480742
H -1.381854 -7.595168 -0.408656
H -2.516944 -1.461900 -3.031577
H -5.577629 -9.774804 -3.140941
H -3.212128 -9.751320 -1.081607
H -0.980769 -5.146965 -0.250753
H -5.095123 -1.796289 -4.773102
H -6.495919 -4.459016 -4.559858
H -2.092825 -3.356002 -1.167562
H -4.611976 -2.989541 -3.560260
H -2.787854 0.236324 -2.619882
H -5.391972 -8.475401 -1.962245
H -7.875211 -5.404269 -5.130314
H -0.756338 -5.966120 1.305386
H -2.706732 -7.456749 0.718367
H -2.258515 -1.780715 -0.433189
H -6.225742 -2.283091 -3.501707
H -1.043817 -3.247757 1.104893
H -8.246077 -7.808445 -2.453938
H -5.240279 -0.047868 -2.985330
H -6.818349 -7.363580 -0.191084
H -9.609094 -7.055334 -3.291537
H -5.317752 -8.104172 0.422489

H -2.620285 -2.804770 1.778848
H -4.782526 1.636083 -1.396281
H -8.799604 -2.936073 -2.976534
H -2.699303 -6.205816 2.636783
H -7.345098 -4.743537 -0.506118
H -9.494876 -6.959296 -1.530682
H -10.031695 -4.128633 -3.410192
H -6.693608 -9.114243 -0.030757
H -7.463309 -0.631415 -2.498524
H -2.130700 -4.622994 3.203508
H -7.040230 -2.511899 -0.913894
H -3.480277 -0.968097 1.848102
H -4.168810 2.302892 0.117561
H -9.058149 -7.806051 1.406919
H -9.722631 -3.759541 -1.709428
H -3.357232 0.787777 1.990405
H -4.427484 -3.700290 2.684512
H -5.748564 1.524931 0.079846
H -3.669189 -7.089260 4.354465
H -4.487937 -8.632425 4.142062
H -7.414034 0.526280 -1.169962
H -6.552761 -2.022101 1.533672
H -4.935067 0.004058 2.042682
H -8.695334 -0.682318 -1.236691
H -8.762346 -8.507052 2.999408
H -7.988803 -4.104558 1.451489
H -8.849130 -5.620898 1.126786
H -4.455156 -4.961904 3.887271
H -8.563798 -9.489552 1.541715
H -6.988197 -0.335569 1.250556
H -4.255715 -7.973517 5.761486
H -8.204182 -1.594122 1.063432
H -7.124455 -8.744922 4.893204
H -9.609828 -4.352775 2.090849
H -5.733674 -6.030972 5.184311
H -7.075520 -3.614068 3.864790
H -8.781999 -6.152935 3.580214
H -7.878353 -7.241702 5.434103
H -6.618243 -7.999131 6.408012
H -7.522367 -4.714949 5.173832
H -8.761631 -3.759142 4.359633
H -3.033515 0.179611 -0.350129
H -5.919652 -10.415087 3.800064

Table S31. Final coordinates for 4a Toluene

Np 0.136483 0.011199 0.020932
Si 0.342412 -0.395121 3.755773
Si -3.735456 -0.971502 0.119387
Si -0.675120 3.359326 -1.456833
Si 0.832774 -2.873154 -2.296751
N 0.946130 0.210831 2.167007
N -1.952281 -0.907869 0.004236
N 2.747009 0.627828 -0.020921
N 1.317118 -1.627001 -1.071433
N 0.450016 1.999178 -1.085855
C 2.110643 0.094824 6.115328
C 1.753874 -0.915315 5.019910
C -0.169848 2.310188 4.744496
C -0.812543 0.925914 4.605453
C -1.514229 -2.385939 4.735140
C 3.034013 -1.471914 4.385565
C -2.143823 1.057266 3.859768
C -0.691209 -2.017525 3.493436
C 0.190516 -3.206706 3.095463
C 2.100571 1.116406 2.296514
C 3.220715 0.662938 1.374568
C -1.387659 3.998184 1.287514
C -4.287278 -2.282892 1.404193
C -4.492265 0.699314 0.637890
C -1.968579 3.431540 -0.012169
C -3.274405 4.153896 -0.366637
C 2.719931 -1.748051 -0.635471
C -0.795385 -4.476366 -0.496571
C 1.580202 -5.243767 -0.770726
C 3.436850 -0.417180 -0.795183
C 2.867360 1.945977 -0.668606
C 1.245241 5.450792 -0.590498
C 0.385112 -4.580055 -1.463411
C -4.543843 -1.469150 -1.545221
C 1.776933 2.116486 -1.714580
C 0.276252 5.049811 -1.707009
C -0.682866 6.207762 -2.015234
C -2.499984 1.792952 -3.055181
C -1.650628 3.064041 -3.115020
C -0.725328 -2.226485 -3.257275
C 2.309808 -3.298526 -3.512569
C -1.582054 -3.332209 -3.887942
C 3.005667 -2.110636 -4.182333
C -0.377703 -1.172718 -4.315577
C -0.739690 3.050186 -4.346402
C 1.889509 -4.327560 -4.571202
H 2.782558 -0.363436 6.854842
H 1.231007 0.452644 6.659720
H 2.634036 0.971414 5.715674
H -1.018523 0.536192 5.613884
H 0.755241 2.297940 5.326742
H 1.262764 -1.759086 5.530543
H -0.856277 3.011359 5.239494
H -0.871690 -2.577985 5.604199
H 3.642292 -1.991761 5.138723
H -2.227120 -1.607675 5.024696
H -2.810879 1.772111 4.361090
H -2.089804 -3.304824 4.560339
H 0.064617 2.734315 3.762066

H 3.660292 -0.671838 3.976289
H -2.683660 0.109183 3.777067
H 2.836790 -2.182776 3.578153
H 0.867586 -3.490709 3.909796
H 2.504798 1.159811 3.318932
H -1.982412 1.430717 2.841825
H -1.389039 -1.819286 2.667317
H -0.420195 -4.090670 2.869240
H -4.016916 -1.991374 2.424304
H -2.079282 3.851217 2.126846
H 1.829592 2.156436 2.052666
H 0.807527 -2.996726 2.215349
H 3.507306 -0.352368 1.661426
H -4.054962 1.085921 1.562762
H -0.435959 3.527685 1.555354
H 4.115399 1.302409 1.462711
H -1.212356 5.076716 1.207786
H -5.375437 -2.415446 1.372813
H -3.830716 -3.258965 1.205369
H -5.564088 0.552868 0.818781
H -1.658039 -1.883529 -0.135724
H 2.787321 -2.070662 0.415944
H -3.994714 4.082184 0.459129
H -0.559703 -3.813707 0.343704
H 1.951329 -4.637222 0.063670
H -4.395981 1.461030 -0.140639
H -2.225770 2.376132 0.174101
H 0.717853 5.715115 0.331587
H 2.729794 2.713304 0.098356
H -3.107495 5.220928 -0.551470
H -1.047896 -5.456040 -0.068000
H 4.497964 -0.481607 -0.501357
H 1.298373 -6.220557 -0.353968
H 1.952892 4.655247 -0.341799
H 3.286744 -2.500593 -1.202091
H -1.700960 -4.095607 -0.981161
H -3.760541 3.744128 -1.257416
H 1.834468 6.330239 -0.885348
H 2.420209 -5.416893 -1.452051
H 3.875752 2.077339 -1.096007
H -5.636802 -1.465770 -1.452358
H 3.403468 -0.131859 -1.849739
H -1.286862 6.477478 -1.141411
H -4.245355 -2.475754 -1.856855
H -3.223892 1.813964 -2.233843
H -4.273659 -0.775319 -2.347680
H 0.080201 -5.228973 -2.297673
H 1.939028 3.084141 -2.209667
H -1.340800 -1.734596 -2.488953
H 1.915427 1.362756 -2.506262
H 0.876690 4.891831 -2.615245
H 3.059024 -3.792414 -2.877390
H -1.875992 0.903854 -2.914405
H -0.122112 7.107611 -2.302872
H -1.370821 5.981792 -2.836617
H -2.332742 3.921669 -3.211216
H 3.357709 -1.370578 -3.459259
H -1.925334 -4.073775 -3.160568
H 0.249948 -0.368640 -3.917201
H -3.068008 1.648398 -3.984493
H 1.422741 -5.218418 -4.137747
H -0.012341 2.231296 -4.299037
H -2.476252 -2.901240 -4.357817
H 3.880509 -2.448398 -4.755101
H -1.287163 -0.711581 -4.721361
H -0.177996 3.983553 -4.462971
H 2.344746 -1.591387 -4.882491
H -1.041930 -3.873186 -4.673065
H 0.155167 -1.618457 -5.162991
H 2.762125 -4.667472 -5.145940
H 1.182275 -3.901419 -5.291943
H -1.322283 2.907513 -5.266931

Table S32. Final coordinates for TS(t) Toluene

Np -0.135743 -0.006255 0.537595
Si -3.005282 -1.253595 -1.511405
Si 1.750241 -3.037253 -0.870621
Si -0.546607 0.184838 4.368093
Si -0.948092 3.326179 -1.325158
N -1.935350 -1.144128 -0.057999
N 1.563233 -1.529715 0.109906
N 0.091552 0.671904 2.747184
N 2.269129 1.168972 0.938358
N 0.020968 1.893297 -0.786792
C -4.355056 -4.706076 0.944722
C -5.484711 -5.447200 0.614115
C -6.751795 -4.864329 0.676224
C -6.876837 -3.534222 1.081154
C -5.746400 -2.793662 1.412622
C -4.458437 -3.359858 1.345579
C -3.268025 -2.585370 1.727350
C 0.187579 -4.320812 -2.981561
C 4.351550 -2.201231 -2.099360
C -2.050449 2.665349 4.496380
C -1.669854 -2.140356 5.681255
C 2.159103 0.164328 5.625281
C 0.272499 -2.600988 4.161222
C -0.950892 -1.709674 4.396479
C 0.534746 1.766988 6.666096
C 0.676743 0.456429 5.883812
C -0.147444 -4.539799 0.696953
C 3.732620 -4.626618 -2.258935
C 3.612474 -3.352106 -1.411057

C 2.265334 -4.910087 1.303754
C 1.282592 -4.624422 0.164247
C 2.758430 -1.230032 0.923179
C 3.287846 0.159851 0.606019
C -3.310653 -3.056886 -2.093489
C -4.724725 -0.462138 -1.232388
C -2.158116 -0.332935 -2.950766
C 1.118052 -2.051434 -3.529988
C 0.575396 -2.949963 -2.413679
C -3.080561 2.537105 0.491604
C -0.655915 6.271145 -1.173336
C -1.549673 2.833646 -4.203960
C -3.737409 4.141471 -1.328331
C -2.815198 2.976674 -0.949446
C -0.697511 5.088860 1.023241
C -0.297859 4.965113 -0.450486
C -3.406137 0.611499 4.039859
C -2.185442 1.165951 4.778329
C 0.945211 1.870861 2.862752
C 2.343133 1.570705 2.351579
C 2.319956 2.322428 0.024038
C 1.439122 2.043071 -1.184283
C -0.849510 3.785684 -3.233710
C 0.535965 4.145065 -3.786673
H -2.449772 -3.208550 2.091317
H -3.491400 -1.765413 2.411101
H -5.854002 -1.763609 1.744353
H -7.859899 -3.074909 1.145595
H -7.634461 -5.443873 0.419743
H -5.378208 -6.485877 0.311791
H -3.373751 -5.172540 0.905818
H 1.132728 1.729605 7.587690
H -0.498438 1.970048 6.963806
H 0.891861 2.632612 6.095115
H -2.350438 1.029769 5.857800
H -1.232467 3.133269 5.051971
H 0.311508 -0.337337 6.553939
H -2.972985 3.200048 4.761637
H -1.014009 -2.061445 6.556997
H 2.694597 0.020490 6.573876
H -2.565572 -1.547443 5.892555
H -4.305925 1.197374 4.273472
H -1.983792 -3.190737 5.616947
H -1.868463 2.842525 3.430998
H 2.648681 1.001280 5.116397
H -3.622408 -0.428615 4.304020
H 2.322062 -0.732858 5.021181
H 0.981336 -2.539664 4.995360
H 1.028954 2.246092 3.892842
H -3.270681 0.659811 2.952675
H -1.645327 -1.873098 3.565053
H -0.019858 -3.655946 4.074605
H -5.334707 -1.064735 -0.554055
H -4.132950 2.257429 0.633679
H 0.534747 2.710630 2.285059
H 0.813718 -2.332889 3.248000
H 2.748329 0.733373 2.923880
H -1.639109 0.565193 -2.610769
H -2.483799 1.661706 0.776199
H 3.027473 2.426322 2.476876
H -2.862829 3.334029 1.208120
H -4.658155 0.549631 -0.823454
H -5.256799 -0.405882 -2.190082
H -2.909720 -0.039849 -3.692060
H -2.633918 -1.919982 0.797893
H 2.539158 -1.285752 2.002384
H -4.791285 3.856877 -1.206306
H -0.246801 -3.706319 1.399871
H 2.290081 -4.088124 2.029037
H -1.427348 -0.971710 -3.452724
H -3.069111 2.130993 -1.604310
H -1.758421 5.344120 1.127119
H 1.922971 3.197156 0.546564
H -3.568699 5.016824 -0.690721
H -0.430248 -5.458054 1.231142
H 4.233186 0.377799 1.130860
H 1.975736 -5.814170 1.857673
H -0.527327 4.169069 1.589305
H 3.576929 -1.946388 0.766221
H -0.878200 -4.373880 -0.100959
H -3.609014 4.460656 -2.368025
H -0.130554 5.888843 1.519466
H 3.289015 -5.067864 0.947029
H 3.362004 2.546570 -0.259143
H -2.704728 -3.287456 -2.975123
H 3.482415 0.216514 -0.467986
H -1.739626 6.425879 -1.228147
H -4.363475 -3.196997 -2.362133
H -2.570179 2.588692 -3.893593
H -3.075031 -3.791001 -1.320224
H 1.333531 -5.465072 -0.543065
H 1.597312 2.846555 -1.914211
H -0.340558 -2.487992 -2.019031
H 1.806143 1.132345 -1.685503
H 0.798230 4.871309 -0.487486
H 4.144812 -3.554458 -0.470498
H -1.001214 1.892572 -4.313255
H -0.240736 7.131836 -0.630239
H -0.264783 6.319335 -2.193203
H -1.437711 4.716354 -3.230635
H 4.376235 -1.292262 -1.493176
H -0.288096 -4.965310 -2.236534
H 1.392126 -1.053417 -3.171246
H -1.614405 3.285138 -5.204106

H 3.255817 -5.494712 -1.791867
H 1.134210 3.247329 -3.980556
H -0.521508 -4.209143 -3.812937
H 5.395316 -2.480715 -2.301051
H 0.377003 -1.920184 -4.330274
H 1.117438 4.792053 -3.120603
H 3.900717 -1.937987 -3.060772
H 1.053069 -4.862033 -3.378945
H 2.005011 -2.489606 -4.001314
H 4.788374 -4.884561 -2.420586
H 3.283665 -4.496569 -3.250146
H 0.442250 4.671476 -4.746738

Table S33. Final coordinates for TS 3-II n-Hexane

Np 0.246808 0.066689 -0.058595
Si 0.127348 2.053782 -3.043086
Si 0.159584 -3.723068 -0.269227
Si 0.250810 1.600892 3.437752
Si -3.669222 0.366083 -0.187624
N 0.968802 -2.127805 -0.011295
N 0.998479 1.115695 1.871098
N 2.905887 0.026688 -0.043773
N 1.092253 1.046587 -1.914939
N -1.895934 0.540109 -0.388990
C 0.900472 1.066613 -5.684654
C -2.734433 2.609985 -3.058856
C -0.497748 4.198389 2.411720
C 1.769745 -0.470337 4.910976
C -4.574804 -0.304797 -1.735545
C 0.290854 4.586559 -4.442246
C -1.442095 3.684062 -1.186250
C -1.528386 2.507023 -2.142526
C 1.753834 4.427138 -2.384411
C 1.119290 3.658514 -3.545957
C -0.827875 -0.269054 -4.438753
C -0.279451 1.133066 -4.708041
C -0.426209 -3.947833 2.547065
C -1.571109 -0.581234 4.005836
C 0.741774 1.378542 6.292076
C 1.356755 1.003914 4.937763
C -2.532476 1.716811 4.327256
C -1.546478 0.869594 3.513998
C -1.783195 -3.260183 -2.381639
C -2.657290 -4.488721 -0.374602
C -1.668674 -3.422697 -0.862959
C 0.531711 -6.162358 -1.808441
C 1.563190 -4.091986 -2.838247
C 1.185524 -4.802108 -1.535587
C 1.405960 -5.477141 1.766849
C 0.098543 -4.779160 1.375129
C 2.363114 -2.331163 0.422921
C 3.314842 -1.369162 -0.268894
C 1.433463 4.251366 4.016098
C 0.126962 3.541808 3.643181
C 2.388343 1.601406 1.778082
C 3.329710 0.515225 1.280518
C 3.394848 0.912780 -1.113816
C 2.482897 0.838706 -2.327468
C -4.040373 -0.867201 1.228641
C -4.548346 1.991246 0.322415
H 1.438021 1.163987 7.114599
H 0.484162 2.441354 6.356099
H -0.171938 0.808119 6.494387
H -2.243884 1.777048 5.383390
H 2.553502 -0.671934 5.654290
H 2.281271 1.592287 4.837267
H -3.537902 1.274802 4.298636
H -1.291112 -0.649596 5.062880
H -2.619898 2.741047 3.952243
H 0.931222 -1.133970 5.143490
H -0.565949 3.677187 4.486792
H 2.157687 -0.770843 3.932413
H -2.575657 -1.015169 3.918526
H 1.895024 3.843440 4.921626
H -0.887340 -1.223586 3.443831
H -1.887039 0.879561 2.467667
H 1.253903 5.319716 4.201223
H -1.438969 3.723780 2.116085
H 1.249170 -6.121978 2.642565
H -3.852382 -0.415538 2.205566
H 2.790902 1.939259 2.745250
H 2.187229 -4.759527 2.040794
H -0.702414 5.264114 2.583853
H 2.175037 4.191852 3.211100
H 3.289720 -0.321240 1.982991
H -0.558252 -4.560889 3.449185
H -3.463507 -1.792911 1.156019
H 2.461813 -2.222420 1.515523
H 0.276646 -3.145129 2.796885
H 1.805908 -6.112552 0.969550
H 0.180686 4.130159 1.554451
H -5.102394 -1.137282 1.187083
H 4.374572 0.868357 1.242528
H 2.466172 2.483228 1.119611
H 2.740486 -3.341820 0.203508
H -1.391780 -3.478769 2.328481
H 4.355814 -1.524856 0.063645
H -4.103593 2.416008 1.227708
H -0.637497 -5.568622 1.163158
H -2.348750 3.763745 -0.575521
H -5.601125 1.773099 0.539872
H -0.591336 3.607829 -0.505104
H 3.279032 -1.563255 -1.344688

H 0.312909 -6.714075 -0.887491
H -5.652674 -0.342307 -1.536187
H 3.374594 1.937848 -0.732349
H -1.710002 1.492536 -1.346632
H -4.528242 2.757028 -0.458068
H -4.424480 0.320453 -2.619209
H -1.346918 4.630851 -1.735775
H -2.694768 -4.566387 0.716183
H 4.440756 0.672725 -1.369344
H -1.965873 -2.464535 -0.411586
H 1.966650 -3.090952 -2.652913
H 1.187687 -6.798828 -2.418230
H -3.656971 2.795275 -2.496154
H -4.245309 -1.319495 -1.978785
H 2.323715 -4.660391 -3.391214
H 2.611181 -0.130662 -2.836242
H -0.410074 -6.059442 -2.358828
H 2.308947 3.766810 -1.710325
H 1.005596 4.950989 -1.781398
H -2.406673 -5.481838 -0.765611
H -1.106804 -2.494834 -2.769204
H 0.702814 -3.976928 -3.504735
H -3.674300 -4.257422 -0.718437
H -2.884658 1.715807 -3.669207
H -0.048952 -0.905964 -4.004386
H -1.669692 -0.264423 -3.737966
H 2.841180 1.592008 -3.045934
H -2.618828 3.457396 -3.750579
H -1.557954 -4.196668 -2.902913
H 2.455456 5.186739 -2.755336
H -2.802344 -2.973895 -2.671330
H -0.549872 5.031267 -3.897547
H -1.168507 -0.753156 -5.364015
H 1.944613 3.273708 -4.163426
H 1.743743 0.504616 -5.266247
H -1.072338 1.727096 -5.185968
H -0.120313 4.068296 -5.315489
H 0.901974 5.418358 -4.817861
H 1.272113 2.056705 -5.968818
H 0.607470 0.554928 -6.611626
H 2.125229 -5.006395 -1.001342

Table S34. Final coordinates for II n-Hexane

Np 0.175472 0.076660 -0.000551
Si 0.211317 2.266386 -3.074758
Si 0.052482 -3.681761 -0.499524
Si 0.400539 1.446347 3.543600
Si -3.769920 -0.002689 0.041923
N 0.836908 -2.121417 -0.040895
N 1.015675 1.159781 1.873716
N 2.837306 -0.004008 -0.061472
N 0.916103 1.073985 -1.918977
N -2.035362 0.027957 -0.401113
C 0.382838 0.617525 -5.450344
C -2.250757 3.936113 -2.959180
C -1.785148 3.161198 2.755425
C 2.910157 0.506116 4.868898
C -4.710770 -1.322896 -0.978097
C 1.044743 4.356320 -4.890311
C -0.835308 3.965202 -0.874384
C -1.070772 3.372265 -2.226853
C 2.268721 4.292067 -2.694695
C 1.603405 3.451201 -3.786451
C -1.831914 0.568098 -4.247684
C -0.619287 1.423005 -4.618071
C -1.862620 -3.606466 1.652268
C -0.126551 -1.302873 4.314858
C 1.283554 1.787791 6.285260
C 1.840976 1.602280 4.866825
C -1.839307 0.361820 5.088729
C -0.836578 0.014176 3.981352
C -0.738356 -3.036715 -3.218489
C -2.388771 -4.415583 -1.920862
C -1.314188 -3.325120 -1.827976
C 0.725172 -6.326821 -1.489445
C 2.319400 -4.527962 -2.222739
C 1.374268 -4.988888 -1.109608
C 0.185253 -4.999891 2.101397
C -0.806008 -4.522842 1.035243
C 2.151951 -2.271628 0.610201
C 3.209699 -1.430657 -0.093303
C 0.351447 4.339782 3.355608
C -0.553116 3.141170 3.660599
C 2.342271 1.743111 1.603450
C 3.321418 0.663175 1.161579
C 3.262210 0.700253 -1.285047
C 2.210371 0.526078 -2.370115
C -4.027021 -0.391940 1.890731
C -4.637898 1.668167 -0.309327
H 2.090156 2.047866 6.984286
H 0.534242 2.583235 6.347799
H 0.823368 0.867401 6.662048
H -1.341965 0.499749 6.054931
H 3.681301 0.723095 5.621247
H 2.348719 2.542765 4.606221
H -2.568052 -0.449310 5.218272
H 0.402016 -1.234076 5.272237
H -2.404790 1.275497 4.882935
H 2.494744 -0.476536 5.111862
H -0.893583 3.228828 4.702672
H 3.420504 0.415291 3.907243
H -0.845050 -2.127188 4.407483
H 1.204491 4.409784 4.039348
H 0.607022 -1.587294 3.552926

H -1.417420 -0.143483 3.059282
H -0.204254 5.284036 3.437213
H -2.520658 2.399338 3.031966
H -0.337359 -5.492802 2.932891
H -3.712948 0.434969 2.533822
H 2.778931 2.253400 2.473886
H 0.747975 -4.162948 2.531097
H -2.295204 4.133516 2.793028
H 0.748488 4.289368 2.334804
H 3.376217 -0.090830 1.951138
H -2.392307 -4.100026 2.478978
H -3.503335 -1.298122 2.206097
H 2.115993 -1.977829 1.673393
H -1.399598 -2.699714 2.057347
H 0.912201 -5.719114 1.708017
H -1.504670 2.979728 1.712917
H -5.096944 -0.553519 2.068763
H 4.338412 1.061223 1.007200
H 2.290322 2.517890 0.820614
H 2.509386 -3.311159 0.618124
H -2.614012 -3.289132 0.921880
H 4.210523 -1.577041 0.345113
H -4.160526 2.492200 0.230261
H -1.318991 -5.409354 0.634917
H -1.739792 3.906089 -0.253216
H -5.685740 1.621560 0.010823
H -0.015038 3.488500 -0.328456
H 3.257388 -1.744219 -1.139195
H 0.065273 -6.715839 -0.706738
H -4.398737 -2.337575 -0.713031
H 3.345922 1.765675 -1.054379
H -1.998512 0.161350 -1.420236
H -4.629290 1.908633 -1.377621
H -5.788871 -1.246531 -0.793051
H -0.594802 5.040002 -0.942131
H -2.873435 -4.616214 -0.960371
H 4.255943 0.348427 -1.609360
H -1.801263 -2.407444 -1.465938
H 2.822001 -3.587077 -1.982901
H 1.492528 -7.090868 -1.675327
H -3.181132 3.784789 -2.389966
H -4.548876 -1.191540 -2.053462
H 3.101000 -5.278419 -2.405434
H 2.125752 -0.543977 -2.618836
H 0.134290 -6.243358 -2.408840
H 2.682231 3.677680 -1.888815
H 1.554983 4.987981 -2.239367
H -1.977505 -5.365463 -2.281357
H 0.051411 -2.277946 -3.194655
H 1.791364 -4.379123 -3.169667
H -3.176758 -4.121058 -2.626860
H -2.399449 3.510218 -3.955121
H -1.541808 -0.271449 -3.606436
H -2.598034 1.145783 -3.719526
H 2.589578 1.004601 -3.283563
H -2.156679 5.027928 -3.083607
H -0.314318 -3.940957 -3.669727
H 3.090538 4.894685 -3.104746
H -1.520033 -2.680691 -3.902300
H 0.226194 4.986404 -4.521469
H -2.304714 0.138255 -5.141546
H 2.375827 2.816703 -4.243885
H 0.764770 -0.242887 -4.889530
H -0.973883 2.258592 -5.239258
H 0.664645 3.789361 -5.746371
H 1.822918 5.032195 -5.270381
H 1.244913 1.217377 -5.763476
H -0.087081 0.223378 -6.361593
H 1.993006 -5.183750 -0.221105

Table S35. Final coordinates for TS II-4a n-Hexane

Np -4.752982 -5.056702 -0.269526
Si -4.875515 -1.328121 -1.000065
Si -6.420875 -6.415702 2.875236
Si -2.718274 -7.242062 -2.664965
Si -7.928040 -5.287185 -2.625447
N -5.193281 -5.457115 1.957409
N -3.098842 -6.462239 -1.080187
N -2.527821 -4.682589 1.148100
N -4.081748 -2.878288 -0.512055
N -6.770073 -4.999021 -1.287372
C -6.944989 -16.503738 1.601964
C -6.064009 -15.358854 2.095063
C -6.705821 -13.982193 1.914334
C -5.835125 -12.829180 2.417560
C -6.481935 -11.446338 2.223008
C -5.629482 -10.318033 2.747877
C -5.180069 -8.767706 -2.909782
C 0.202147 -6.610114 -2.576564
C -4.713731 1.524547 -0.475017
C -7.150133 -1.290044 0.813125
C -7.876468 -4.276894 4.216860
C -6.866800 -7.733859 5.385147
C -8.328748 -8.467766 2.024619
C -4.431430 -7.527024 4.763524
C -5.811988 -6.870752 4.678151
C -6.357751 -8.093547 0.491857
C -6.871850 -8.040230 1.916979
C -5.157272 -2.098280 -3.761034
C -7.623923 -0.486803 -1.518499
C -6.782925 -1.446008 -0.666716
C -3.179729 -0.619191 -3.278766
C -4.624071 -0.955576 -2.896387

C -3.970841 0.086516 1.451429
C -4.061831 0.194768 -0.073070
C -2.857732 -4.985338 2.552363
C -4.316027 -4.644346 2.823565
C -2.345713 -4.845041 -4.264921
C -0.524577 -8.400571 -4.171963
C -0.822279 -7.721410 -2.827787
C -3.540610 -6.715494 -5.432121
C -3.289899 -6.037833 -4.079156
C -8.664234 -4.816532 1.899491
C -8.063089 -5.391924 3.182088
C -8.914751 -6.915563 -2.400347
C -7.074714 -5.384914 -4.327374
C -3.269082 -9.934171 -1.764486
C -3.661157 -8.937931 -2.860583
C -2.191576 -6.841423 0.019961
C -1.515654 -5.608318 0.605436
C -2.141780 -3.270953 0.970099
C -2.615259 -2.760841 -0.383148
C -9.221751 -3.876120 -2.696974
H -7.174277 -16.399912 0.534859
H -7.899101 -16.533682 2.141332
H -6.457153 -17.474801 1.740967
H -5.827060 -15.511777 3.156944
H -5.102473 -15.383032 1.564183
H -6.935882 -13.827411 0.850258
H -7.672563 -13.962611 2.437961
H -5.613788 -12.976271 3.483980
H -4.865621 -12.847754 1.900413
H -6.684212 -11.303291 1.152706
H -7.458393 -11.440368 2.725724
H -4.666189 -10.201072 2.243069
H -6.236116 -9.047985 2.463684
H 0.505175 -8.782749 -4.188764
H -1.186255 -9.247848 -4.377255
H -0.615471 -7.698323 -5.008056
H -2.611266 -7.095394 -5.870538
H 1.222463 -7.017367 -2.599793
H -0.664514 -8.484723 -2.051367
H -3.963187 -5.999583 -6.149573
H -1.402460 -5.159205 -4.725242
H -4.235163 -7.557944 -5.367589
H 0.154281 -5.828343 -3.340553
H -3.340577 -9.353619 -3.826851
H 0.072700 -6.124188 -1.606442
H -2.787307 -4.092611 -4.930695
H -2.194862 -10.149329 -1.756253
H -2.103616 -4.350266 -3.318918
H -4.255687 -5.643750 -3.727238
H -3.789164 -10.892751 -1.897641
H -5.505020 -8.174188 -3.769526
H -3.102594 -0.382296 -4.349019
H -6.438857 -6.268911 -4.421038
H -1.405130 -7.544472 -0.287402
H -2.506300 -1.463259 -3.090938
H -5.687395 -9.739922 -2.978969
H -3.539787 -9.557314 -0.771055
H -0.986504 -5.092345 -0.199719
H -5.077561 -1.863666 -4.831694
H -6.473017 -4.498846 -4.545525
H -2.093656 -3.322621 -1.176008
H -4.588334 -3.017635 -3.583710
H -2.789439 0.244525 -2.729708
H -5.555402 -8.271937 -2.009325
H -7.848511 -5.458014 -5.100939
H -0.775798 -5.867793 1.380888
H -2.734064 -7.362579 0.827003
H -2.258423 -1.726411 -0.487980
H -6.207550 -2.321597 -3.548515
H -1.053639 -3.149658 1.098406
H -8.258889 -7.791440 -2.373013
H -5.238282 -0.065282 -3.096739
H -6.856679 -7.354330 -0.151634
H -9.610112 -7.047463 -3.238112
H -5.275450 -7.912872 0.445947
H -2.633521 -2.685313 1.750815
H -4.759290 1.662591 -1.560520
H -8.767013 -2.926073 -2.992548
H -2.722216 -6.059140 2.706377
H -7.343355 -4.719614 -0.481571
H -9.507242 -6.909827 -1.479216
H -10.000656 -4.115921 -3.430422
H -6.535218 -9.076405 0.036433
H -7.457572 -0.617420 -2.592146
H -2.169694 -4.456614 3.233472
H -7.037059 -2.469998 -0.974879
H -3.511189 -0.851424 1.774360
H -4.149599 2.373405 -0.064751
H -9.002010 -7.758738 1.525185
H -9.714536 -3.727554 -1.729765
H -3.367737 0.906624 1.865307
H -4.467958 -3.565972 2.656278
H -5.735698 1.608498 -0.088384
H -3.628742 -6.838068 4.487773
H -4.346467 -8.403575 4.114643
H -7.415885 0.563118 -1.283432
H -6.552548 -1.936030 1.464984
H -4.955364 0.143192 1.926032
H -8.694511 -0.648832 -1.334474
H -8.664240 -8.554972 3.063280
H -8.006596 -4.051493 1.471507
H -8.838050 -5.581329 1.135203
H -4.506318 -4.793152 3.895073
H -8.490570 -9.441163 1.544355

H -7.004324 -0.258647 1.153327
H -4.223931 -7.861862 5.789551
H -8.207483 -1.532114 0.984263
H -6.995885 -8.703878 4.893076
H -9.629153 -4.329282 2.096173
H -5.759817 -5.921184 5.229012
H -7.126362 -3.546806 3.889687
H -8.781581 -6.116608 3.591094
H -7.848435 -7.249519 5.425723
H -6.566273 -7.937879 6.421909
H -7.563445 -4.656896 5.194913
H -8.813467 -3.723889 4.370331
H -3.029384 0.227732 -0.451882
H -5.514939 -10.309062 3.833935

Table S36. Final coordinates for 4a n-Hexane

Np 0.140343 0.009395 0.017744
Si 0.332351 -0.380400 3.748490
Si -3.715883 -0.987635 0.118405
Si -0.676263 3.355946 -1.455705
Si 0.830941 -2.875236 -2.290627
N 0.935029 0.228435 2.159587
N -1.930923 -0.922919 0.008934
N 2.745646 0.627201 -0.024250
N 1.316294 -1.632102 -1.061293
N 0.446452 1.991091 -1.092052
C 2.102754 0.093318 6.110356
C 1.744762 -0.909357 5.008499
C -0.167781 2.323553 4.745052
C -0.815685 0.941954 4.603618
C -1.523846 -2.372625 4.724954
C 3.024188 -1.464478 4.371527
C -2.147936 1.078344 3.860699
C -0.705726 -1.998393 3.481765
C 0.172771 -3.185736 3.071839
C 2.092119 1.130734 2.288783
C 3.214354 0.669442 1.372598
C -1.379053 3.994973 1.288210
C -4.268068 -2.299826 1.401879
C -4.473932 0.682601 0.636934
C -1.965504 3.425715 -0.007747
C -3.273936 4.145630 -0.357724
C 2.719993 -1.752059 -0.628165
C -0.797667 -4.485773 -0.495987
C 1.577953 -5.251315 -0.772396
C 3.436843 -0.421406 -0.792257
C 2.864928 1.942182 -0.678686
C 1.264239 5.434637 -0.596320
C 0.383145 -4.584719 -1.462899
C -4.519635 -1.484068 -1.548516
C 1.772222 2.107718 -1.723592
C 0.274372 5.047954 -1.699433
C -0.691045 6.209820 -1.972166
C -2.499227 1.793103 -3.056466
C -1.651093 3.065095 -3.114880
C -0.726193 -2.224521 -3.249693
C 2.307691 -3.298934 -3.507617
C -1.585324 -3.327875 -3.881455
C 3.005231 -2.111178 -4.175968
C -0.377707 -1.170342 -4.307332
C -0.739183 3.052223 -4.345671
C 1.883944 -4.324761 -4.568183
H 2.772299 -0.371189 6.847919
H 1.223417 0.450684 6.655349
H 2.629348 0.970563 5.716471
H -1.020772 0.550225 5.611437
H 0.758990 2.305623 5.324390
H 1.252125 -1.755504 5.513634
H -0.850184 3.025335 5.244435
H -0.878238 -2.572101 5.589997
H 3.630725 -1.990612 5.121543
H -2.233163 -1.594116 5.022519
H -2.810198 1.797389 4.362130
H -2.102640 -3.288687 4.546741
H 0.064927 2.749547 3.763153
H 3.652412 -0.662727 3.968590
H -2.692029 0.132406 3.781594
H 2.825694 -2.169294 3.559207
H 0.855711 -3.474412 3.879581
H 2.493128 1.176774 3.312340
H -1.986261 1.448102 2.841522
H -1.406245 -1.794030 2.659567
H -0.439911 -4.068046 2.845015
H -4.000206 -2.007410 2.422204
H -2.070049 3.856133 2.129222
H 1.824453 2.170401 2.040216
H 0.782978 -2.970113 2.188231
H 3.496873 -0.345085 1.665927
H -4.037643 1.068300 1.562471
H -0.430092 3.519082 1.556017
H 4.110532 1.306991 1.460455
H -1.197302 5.071924 1.202550
H -5.355938 -2.432884 1.367653
H -3.810349 -3.275419 1.204339
H -5.545849 0.535501 0.815918
H -1.632256 -1.899761 -0.112015
H 2.789319 -2.072961 0.423508
H -3.991975 4.069655 0.469435
H -0.559863 -3.830373 0.349115
H 1.949166 -4.647202 0.063661
H -4.376770 1.445082 -0.140519
H -2.220823 2.370300 0.181264
H 0.756239 5.672428 0.343563

H 2.728721 2.712808 0.085013
H -3.109928 5.213369 -0.540076
H -1.052204 -5.468082 -0.074982
H 4.497662 -0.484286 -0.496801
H 1.295379 -6.229012 -0.358605
H 1.985494 4.641584 -0.382123
H 3.285494 -2.505681 -1.194578
H -1.702147 -4.099567 -0.978210
H -3.760555 3.736789 -1.248596
H 1.837425 6.326428 -0.885254
H 2.417566 -5.422970 -1.454467
H 3.872620 2.071468 -1.108432
H -5.612681 -1.476969 -1.458401
H 3.404526 -0.140640 -1.848037
H -1.272603 6.470041 -1.080447
H -4.223418 -2.491811 -1.857893
H -3.225221 1.813580 -2.237055
H -4.244804 -0.791547 -2.350264
H 0.078412 -5.230166 -2.299906
H 1.931787 3.073798 -2.222748
H -1.339945 -1.732413 -2.480106
H 1.909787 1.351330 -2.512786
H 0.857424 4.902930 -2.620998
H 3.056621 -3.795913 -2.874494
H -1.873866 0.905394 -2.913247
H -0.136596 7.112216 -2.263473
H -1.399562 5.993342 -2.778386
H -2.333347 3.922576 -3.211184
H 3.362826 -1.375277 -3.451575
H -1.927354 -4.071427 -3.155643
H 0.249718 -0.366582 -3.908200
H -3.064464 1.647448 -3.987165
H 1.417107 -5.216067 -4.135997
H -0.011032 2.234261 -4.297055
H -2.480128 -2.894444 -4.347561
H 3.876304 -2.450239 -4.753466
H -1.287011 -0.708935 -4.712841
H -0.179099 3.986421 -4.462544
H 2.343384 -1.586812 -4.871267
H -1.047323 -3.866814 -4.669356
H 0.155022 -1.615719 -5.154962
H 2.754838 -4.663816 -5.145774
H 1.175629 -3.895572 -5.285920
H -1.321221 2.908105 -5.266167

Table S37. Final coordinates for TS(h) n-Hexane

Np 1.961525 -0.141128 -0.088755
Si 5.608485 0.694474 -0.874398
Si 0.836210 -3.356271 1.650303
Si -0.740890 2.665445 -0.294465
Si 1.294496 -0.564886 -3.800796
N 1.152115 -1.581584 1.521788
N 2.405525 0.868838 2.366580
N 4.202266 0.272201 0.174631
N 0.836313 1.881468 0.126482
N 1.905161 -0.983043 -2.159023
C 1.387423 -8.709761 -5.772756
C 2.391229 -7.712540 -5.199757
C 1.727436 -6.494429 -4.556129
C 2.724182 -5.481540 -3.989047
C 2.039237 -4.252244 -3.367646
C 3.024635 -3.266354 -2.790639
C 6.193140 -2.057194 -1.553623
C 5.986382 0.864167 -3.761509
C 2.259987 -3.530050 4.267760
C -2.028815 -2.878023 1.752034
C -1.856612 0.051625 -0.881113
C -0.181534 4.328179 -2.690418
C -2.325987 1.905322 2.072885
C 0.171322 5.481019 -0.498925
C -0.620094 4.386962 -1.226175
C -2.775522 4.207406 1.202866
C -1.710675 3.113780 1.359977
C 1.704141 -5.818418 0.406056
C -1.137355 -3.728583 -0.433963
C -0.981941 -3.779209 1.089209
C 3.517992 -4.151737 0.885054
C 2.047569 -4.326112 0.494375
C 0.729248 -0.872635 2.744754
C 1.903174 -0.113327 3.341628
C 5.905766 3.449535 0.227946
C 4.476288 2.595186 -2.760142
C 4.966956 1.146935 -2.650194
C 7.930491 2.447465 -0.868579
C 6.653698 2.145610 -0.071883
C 7.680315 -1.103856 0.226548
C 6.872594 -0.789119 -1.037545
C -0.122532 -4.315841 4.312075
C 1.121884 -4.132357 3.435938
C 1.594216 2.759470 1.047944
C 1.673747 2.145330 2.436437
C 3.861584 1.064716 2.490195
C 4.590807 0.082319 1.587522
C -3.139964 2.013883 -1.794845
C -1.750738 1.472208 -1.436379
C 2.450771 -1.081646 -5.240921
C -0.424253 -1.317539 -4.182553
C 1.167011 1.332601 -3.933577
H 0.761178 -8.245789 -6.543740
H 0.718134 -9.094483 -4.994288
H 1.890506 -9.569026 -6.229492
H 3.024240 -8.216101 -4.456369
H 3.068822 -7.376597 -5.996741

H 1.087795 -5.996173 -5.298722
H 1.054260 -6.829504 -3.754165
H 3.356307 -5.969969 -3.234905
H 3.403818 -5.150549 -4.786643
H 1.430739 -3.768230 -4.141262
H 1.339141 -4.586595 -2.591909
H 3.552274 -3.615097 -1.901143
H 3.728087 -2.857999 -3.518546
H 0.120485 -4.910358 5.203913
H -0.930720 -4.836770 3.789502
H -0.520299 -3.358954 4.670714
H -1.162131 -4.814210 1.417382
H -2.001536 -2.925799 2.844685
H 1.446157 -5.145337 3.150429
H -3.043486 -3.159777 1.438986
H 1.843201 -6.325047 1.369264
H 2.545425 -4.212478 5.080110
H 0.672320 -6.002438 0.090290
H -2.179446 -3.914806 -0.728402
H 2.360548 -6.328634 -0.311221
H -1.881375 -1.832310 1.463263
H 1.956047 -2.591414 4.742799
H -0.520892 -4.474861 -0.944302
H 3.160534 -3.329064 3.680290
H 3.726926 -4.601777 1.862602
H 0.328791 -1.544701 3.517531
H -0.858758 -2.748185 -0.836442
H 1.904033 -3.890683 -0.502644
H 4.183033 -4.647249 0.164679
H -1.205509 -0.861059 -3.568354
H -2.339774 -0.623930 -1.598168
H -0.085239 -0.163919 2.534087
H 3.814475 -3.098538 0.938276
H 2.703946 -0.828014 3.547071
H 0.941838 1.798144 -2.972644
H -0.875935 -0.381369 -0.646423
H 1.641631 0.381997 4.292037
H -2.448337 0.018124 0.037716
H -0.680389 -1.144390 -5.234853
H -0.450892 -2.396434 -4.002819
H 0.384279 1.614650 -4.646488
H 2.424143 -2.139894 -2.351536
H 4.374029 -0.943825 1.930146
H -3.631853 1.363880 -2.530901
H 5.461689 -2.428339 -0.827965
H 7.045197 -1.513771 1.020275
H 2.111454 1.751029 -4.292626
H -1.171810 1.419829 -2.368623
H -3.210189 1.534381 1.541967
H 0.656266 1.942411 2.781826
H -3.797016 2.055871 -0.918565
H 6.921320 -2.861909 -1.726754
H 4.174704 0.972350 3.542982
H 8.448815 -1.860734 0.016314
H -1.625802 1.069907 2.166251
H 5.669226 0.208944 1.756802
H 5.658997 -1.889459 -2.493512
H -3.100653 3.019381 -2.227283
H -2.655544 2.172319 3.086564
H 8.196549 -0.227829 0.633691
H 2.146562 2.822483 3.167781
H 2.107713 -0.563794 -6.145380
H 4.104234 2.078552 2.163692
H -3.568261 3.911331 0.506849
H 2.438861 -2.153642 -5.455037
H -0.762510 3.609012 -3.275685
H 3.486143 -0.775625 -5.061602
H 7.584901 -0.452865 -1.805451
H 1.155985 3.760802 1.153245
H 4.103487 0.483301 -2.803789
H 2.614188 2.937159 0.675246
H -0.935920 3.522237 2.026052
H 6.972711 1.733873 0.897424
H 0.875348 4.059253 -2.789734
H -3.259859 4.411529 2.168119
H -2.362871 5.155532 0.846319
H -1.674644 4.703157 -1.227065
H 4.936149 3.281411 0.705658
H 6.281776 -0.188140 -3.809352
H 3.736540 2.852703 -1.995243
H -0.306581 5.310129 -3.168296
H 8.539374 1.555787 -1.049319
H 1.248462 5.281589 -0.528577
H 5.569886 1.129461 -4.742811
H 6.495495 4.090818 0.897511
H 4.013881 2.778546 -3.738661
H -0.116710 5.596287 0.552210
H 5.717398 4.029002 -0.680803
H 6.901434 1.455142 -3.637751
H 5.306354 3.304721 -2.671705
H 8.563627 3.169050 -0.334073
H 7.699972 2.891825 -1.844185
H 0.016802 6.455266 -0.983247

Table S38. Final coordinates for TS II-4a

C -4.829749000 1.434178000 -0.065692000
Si -3.899699000 -0.219363000 0.205161000
C -4.821644000 -1.527813000 -0.847706000
C -4.115137000 -0.681566000 2.042370000
N -2.181536000 -0.125422220 -0.297865000
Np 0.050220000 0.157113000 -0.033469000
N 0.795322000 -2.011341000 -0.028552000
Si 0.120205000 -3.620084000 -0.489948000
C -1.249487000 -3.375733000 -1.843160000

C -0.664910000 -3.114764000 -3.235723000
N 0.779372780 1.121270220 -2.014080000
Si 0.064870000 2.226630661 -3.251947780
C -1.125658916 3.446557048 -2.390300559
C -2.342357441 3.928489780 -3.138603780
N 0.860038000 1.284009000 1.829615000
Si 0.229131000 1.554159000 3.498782000
C 1.661218000 1.910356000 4.791004000
C 2.817785000 0.907824000 4.850434000
C 2.094395780 0.581028220 -2.414177000
C 3.122370000 0.839880000 -1.325016000
N 2.719917000 0.158544000 -0.082305000
C 3.145418000 -1.253991000 -0.072553000
C 2.107209000 -2.105470000 0.643298000
C 3.179562000 0.881062000 1.118739000
C 2.152019000 1.926672000 1.532810000
C -0.917218000 3.127089000 3.618705000
C -2.144985000 3.028293000 2.714186000
C -0.832723000 -0.001093000 3.983953000
C 0.022411000 -1.220047000 4.347600000
C -0.157259000 4.430279000 3.354480000
C -0.681108000 -4.528988000 1.035893000
C -1.765733000 -3.663105000 1.676348000
C 1.541366000 -4.802862000 -1.125519000
C 0.981132000 -6.149907000 -1.605664000
C 0.333695000 -4.992711000 2.085557000
C 2.492227000 -4.182745000 -2.174939000
C -2.269361000 -4.519547000 -1.902710000
C -1.870369000 0.256780000 5.083673000
C 1.100327000 2.139525000 6.202106000
C -1.001505220 1.276775220 -4.591172559
C -0.141450000 0.458181441 -5.559599559
C 1.433880220 3.140896000 -4.306283000
C 0.797271000 4.059672000 -5.356173000
C -2.082798000 0.394669000 -3.966494780
C 2.489664000 3.904780000 -3.502679000
C -1.316471661 3.323715559 -0.906481000
H 2.136312000 -5.026899000 -0.230326000
H -0.764230220 -0.048435780 -6.306749559
H 0.577227780 1.077622441 -6.106258780
H 1.567108000 4.485494220 -6.012846000
H 0.081558000 3.535718000 -5.998552000
H -1.502632441 2.064834220 -5.167613780
H 0.426633000 -0.320541559 -5.036817559
H 1.951327220 2.342152000 -4.855110220
H -2.713174780 -0.07296400 -4.735033000
H 0.276854000 4.904802780 -4.891563780
H -1.459268000 -2.896670000 -3.961945000
H 3.258575000 4.318701220 -4.168419000
H -0.117551000 -3.985733000 -3.614255000
H -2.781776084 4.821013678 -2.672065441
H 2.479096780 1.022436000 -3.344113000
H -2.744766441 0.958604559 -3.301673780
H -1.631181000 -0.419472000 -3.386496000
H -2.142590322 4.157420220 -4.189064000
H -3.057616000 -4.295874000 -2.634220000
H 2.159215000 -3.208520000 -2.545268000
H 0.026999000 -2.265978000 -3.244148000
H -1.809148000 -5.464807000 -2.211151000
H 2.051825780 4.747063780 -2.957487000
H 2.998933220 3.269946000 -2.771419220
H 0.398469000 -6.043490000 -2.527225000
H 2.044503780 -0.502291780 -2.610782000
H 2.592363000 -4.833995000 -3.051794000
H -4.693593000 -1.343796000 -1.920304000
H -3.132114137 3.160348916 -3.128645899
H 1.795916000 -6.853927000 -1.823020000
H 3.502525000 -4.044299000 -1.773963000
H -1.787032000 -2.472123000 -1.520581000
H 4.137207780 0.525135000 -1.625954220
H -2.761646000 -4.696631000 -0.941585000
H -1.855155762 4.18218401 -0.483667458
H -5.896786000 -1.495329000 -0.634385000
H -4.838493000 1.726428000 -1.121206000
H -2.198686000 -0.161359441 -1.324788000
H 3.151055000 1.915565000 -1.128469000
H -4.471353000 -2.543353000 -0.638255000
H 0.335268000 -6.626835000 -0.860052000
H 3.217179000 -1.598989000 -1.106735000
H -0.367357322 3.233102119 -0.365937899
H -5.873560000 1.332809000 0.255630000
H -1.924167000 2.430036678 -0.673325405
H -1.162859000-5.426991000 0.622122000
H -4.386432000 2.255647000 0.505934000
H 4.144597000 -1.350679000 0.382788000
H -2.525978000 -3.352614000 0.952622000
H 2.491756000 -3.133986000 0.686714000
H 2.056733000 2.670220000 0.722980000
H 4.175905000 1.321091000 0.946528000
H -5.179784000 -0.868987000 2.228430000
H -1.855158000 3.001801780 1.659523000
H 1.075559000 -5.686506000 1.674720000
H -1.332154000 -2.749814000 2.098466000
H 2.046178000 -1.780767000 1.695165000
H -3.569429000 -1.588326000 2.315675000
H -2.277766000 -4.192733000 2.491879000
H 3.271817000 0.154892000 1.930804000
H 0.257598000 4.452406000 2.339846000
H -2.808136000 3.894721000 2.842981000
H 0.877973000 -4.149661000 2.526831000
H 2.565265000 2.487942000 2.382773000
H -3.808813000 0.124194000 2.714992000
H -0.168567000 -5.514410000 2.912279000
H -2.740121000 2.131799000 2.915300000

H	-0.820580000	5.301133000	3.447352000
H	-1.390014000	-0.252903000	3.067593000
H	0.801132000	-1.419983000	3.604417000
H	0.672290000	4.581851000	4.053777000
H	-0.595123000	-2.123431000	4.433301000
H	3.313432000	0.778784000	3.885155000
H	-1.268349000	3.147468000	4.660853000
H	2.490185000	-0.081600000	5.183789000
H	-2.548680000	1.081813000	4.846251000
H	0.516840000	-1.082896000	5.315955000
H	-2.489377000	-0.635193000	5.248125000
H	2.087414000	2.870894000	4.464972000
H	3.583774000	1.248118000	5.561215000
H	-1.393301000	0.495319000	6.040558000
H	0.705125000	1.214131000	6.636380000
H	0.299296000	2.885302000	6.227022000
H	1.891159000	2.493495000	6.877678000
C	-0.173115119	5.950548339	-2.124554661
C	-0.651586220	6.962383678	-2.959686441
C	-0.335996441	8.297251899	-2.705310000
C	0.462132559	8.626369119	-1.610324000
C	0.948172559	7.620502119	-0.774565000
C	0.631820000	6.286270899	-1.033243220
H	-1.282658000	6.710022119	-3.809402661
H	-0.714963661	9.079872899	-3.357279000
H	0.704225559	9.664533339	-1.407785000
H	1.568930559	7.876231119	0.080976000
H	1.009799661	5.505198119	-0.380935000
H	-0.442301084	4.936898939	-2.300480304

Table S39. Final coordinates for TS II-4

Np	0.446635765	-0.752465876	0.352137569
Np	-5.381280765	8.664717876	-3.783530569
Si	-2.660477235	0.015370124	2.648658569
Si	-1.054574235	-4.045142876	-0.413145431
Si	-0.045423235	2.003413124	-2.273620431
Si	2.471841765	-0.028199876	3.442595569
Si	-2.939021765	7.441234876	-6.568485569
Si	-8.051468765	8.886765876	-6.406366569
Si	-5.202951765	5.563988876	-1.661847569
Si	-2.918496765	11.294131876	-2.975157569
N	-1.248703235	0.086262124	1.568851569
N	0.335447765	-2.955066876	-0.182261431
N	0.550519765	0.447846124	-1.594037431
N	2.650175765	-1.470025876	-0.945270431
N	2.261751765	-0.373283876	1.701976569
N	-3.854849765	7.670280876	-5.065500569
N	-7.263173765	9.365660876	-4.884363569
N	-5.976722765	7.036951876	-2.309977569
N	-7.106921765	9.677366876	-2.026597569
N	-4.393499765	10.353270876	-2.616860569
C	3.884988765	2.537922124	3.361226569
C	0.662773765	-0.067092876	5.734003569
C	-1.585475235	0.418297124	-4.202213431
C	-0.997149235	3.569779124	0.028807569
C	2.351068765	3.292942124	-1.201050431
C	-2.704009235	2.068054124	-1.085462431
C	-1.420015235	2.861217124	-1.251946431
C	1.141395765	4.584790124	-2.981938431
C	1.483192765	3.183130124	-2.460530431
C	-0.067265235	-6.625860876	0.615703569
C	-3.880719235	-3.786638876	0.257773569
C	-0.800666235	-3.954521876	-3.351912431
C	-2.968508235	-2.090884876	-1.363010431
C	-2.639929235	-2.984452876	-0.159328431
C	-2.158226235	-5.846996876	-2.394554431
C	-0.978656235	-4.892901876	-2.152769431
C	1.626848765	-3.632093876	-0.391885431
C	2.479808765	-2.864952876	-1.390990431
C	-4.316915235	0.208022124	1.737776569
C	-2.565706235	1.436174124	3.905765569
C	-2.741021235	-1.629518876	3.580146569
C	-0.928169235	-4.984564876	2.310072569
C	-1.070189235	-5.491944876	0.871787569
C	4.451231765	-2.210829876	3.736471569
C	1.034916765	-2.263763876	4.563736569
C	0.959961765	-0.741344876	4.387500569
C	0.117796765	1.922176124	-5.254189431
C	-0.814227235	1.736581124	-4.046422431
C	1.410556765	-0.179270876	-2.624013431
C	2.782592765	-0.542382876	-2.081613431
C	3.770399765	-1.338315876	0.000385569
C	3.519565765	-0.182902876	0.955351569
C	1.383891765	2.611741124	3.142478569
C	2.566017765	1.874518124	3.778802569
C	4.147598765	-0.753033876	4.102250569
C	4.302686765	-0.541214876	5.616345569
C	-1.362837765	10.720960876	-0.578117569
C	-1.199983765	11.472942876	-5.330947569
C	-7.475620765	4.093564876	-2.783745569
C	-2.455090765	5.046238876	-2.609672569
C	-5.559807765	6.178439876	1.205667431
C	-4.260488765	4.309053876	-4.117957569
C	-3.808178765	4.992508876	-2.846788569
C	-3.456187765	6.993974876	0.172538431
C	-4.508003765	5.877196876	0.124772431
C	-9.126782765	11.437771876	-7.427584569
C	-7.139730765	7.286882876	-8.671703569
C	-10.169825765	7.363929876	-5.016618569
C	-7.295521765	6.093414876	-6.462777569
C	-7.024138765	7.438937876	-7.148535569
C	-10.634494765	8.023842876	-7.399627569
C	-9.907327765	8.413320876	-6.103395569
C	-7.997978765	10.438504876	-4.188184569

C	-8.335961765	10.029102876	-2.762553569
C	-3.318168765	5.791570876	-7.432802569
C	-1.072355765	7.459044876	-6.215652569
C	-3.324225765	8.809284876	-7.817526569
C	-6.715685765	10.950872876	-7.923217569
C	-8.097758765	10.330689876	-7.697289569
C	-4.344891765	13.872585876	-2.671132569
C	-3.608168765	12.131677876	-5.650131569
C	-2.644529765	11.222556876	-4.875581569
C	-7.081579765	3.593872876	-0.359225569
C	-6.418858765	4.030871876	-1.671460569
C	-7.286492765	7.257787876	-1.663694569
C	-7.354595765	8.632559876	-1.018415569
C	-6.479337765	10.868942876	-1.430234569
C	-4.979233765	10.662666876	-1.303481569
C	-1.179342765	9.066793876	-2.464086569
C	-1.368530765	10.542916876	-2.101976569
C	-3.060339765	13.113406876	-2.317449569
C	-1.829636765	13.953004876	-2.694563569
H	5.310972765	-0.822469876	5.948742569
H	4.141509765	0.500354124	5.914295569
H	3.597867765	-1.158598876	6.184191569
H	1.471357765	-0.220932876	6.457168569
H	5.467333765	-2.488102876	4.048487569
H	4.924686765	-0.136276876	3.626078569
H	-0.248687235	-0.484107876	6.182927569
H	1.808453765	-2.543710876	5.287432569
H	0.513271765	1.012876124	5.638662569
H	3.764157765	-2.905756876	4.228190569
H	2.478526765	1.978232124	4.870849569
H	4.377706765	-2.393154876	2.660569569
H	0.088573765	-2.669367876	4.945374569
H	4.753201765	2.103229124	3.867087569
H	1.259571765	-2.780739876	3.625054569
H	0.117429765	-0.520715876	3.715553569
H	3.875651765	3.610181124	3.599628569
H	0.417873765	2.191344124	3.440647569
H	-0.224893235	-7.448288876	1.326319569
H	-1.787553235	-1.892055876	4.044023569
H	4.397825765	-0.112952876	1.612064569
H	0.967202765	-6.290105876	0.749805569
H	1.380900765	3.676300124	3.413030569
H	4.055144765	2.455859124	2.281316569
H	3.828444765	-2.261061876	0.584337569
H	-1.032461235	-5.803911876	3.033875569
H	-3.044107235	-2.450559876	2.924370569
H	2.175966765	-3.738566876	0.557335569
H	0.056280765	-4.531674876	2.469650569
H	-0.151261235	-7.048820876	-0.390675431
H	1.443354765	2.554908124	2.049213569
H	-3.492434235	-1.554992876	4.374398569
H	4.723872765	-1.224300876	-0.542828431
H	3.520644765	0.762778124	0.388919569
H	1.519547765	-4.656768876	-0.777384431
H	-1.675718235	-4.227357876	2.564865569
H	3.461519765	-3.342526876	-1.548201431
H	-1.710554235	1.326738124	4.578743569
H	-2.076825235	-5.925959876	0.768209569
H	-1.858757235	4.033471124	0.527268569
H	-3.473651235	1.463099124	4.519028569
H	-0.533020235	2.900228124	0.764663569
H	1.961597765	-2.855720876	-2.353238431
H	-2.287289235	-6.573118876	-1.585181431
H	-4.415649235	1.191141124	1.268761569
H	3.270744765	0.369115124	-1.726171431
H	-1.110241235	1.088584124	1.389043569
H	-2.479560235	2.406852124	3.404890569
H	-4.440071235	-0.550376876	0.958995569
H	-0.265703235	4.362048124	-0.159678431
H	-3.726746235	-4.345400876	1.186081569
H	3.425987765	-0.979237876	-2.864375431
H	-2.389693235	-2.317704876	0.680811569
H	-0.005498235	-3.221594876	-3.187052431
H	-2.020035235	-6.415794876	-3.323736431
H	-3.504363235	2.712696124	-0.692009431
H	-5.143410235	0.093500124	2.449121569
H	-0.551626235	-4.519737876	-4.259944431
H	0.933796765	-1.081981876	-3.036020431
H	-3.101231235	-5.297702876	-2.496764431
H	2.557654765	2.312881124	-0.761845431
H	1.865920765	3.895143124	-0.425847431
H	-4.175076235	-4.507853876	-0.512974431
H	-2.105580235	-1.513363876	-1.713179431
H	-1.714680235	-3.392432876	-3.565891431
H	-4.739230235	-3.120972876	0.416387569
H	-2.589543235	1.230773124	-0.387898431
H	-0.907538235	-0.441394876	-4.209814431
H	-2.307544235	0.249651124	-3.399476431
H	1.579855765	0.471355124	-3.493982431
H	-3.062680235	1.659139124	-2.034501431
H	-3.326120235	-2.685697876	-2.210498431
H	3.312562765	3.775096124	-1.422654431
H	-3.762181235	-1.374247876	-1.117602431
H	0.555786765	5.152544124	-2.250284431
H	-2.137482235	0.400600124	-5.151529431
H	2.098069765	2.693761124	-3.232277431
H	0.841685765	1.103705124	-5.342474431
H	-1.556246235	2.547982124	-4.095872431
H	0.568422765	4.564517124	-3.914686431
H	2.052402765	5.165976124	-3.177350431
H	0.684962765	2.856591124	-5.222486431
H	-0.460951235	1.924980124	-6.187611431
H	-0.072307235	-5.516223876	-2.099838431
H	-1.853020765	14.927906876	-2.189563569

H -0.887378765 13.467351876 -2.419853569
H -1.795072765 14.154403876 -3.771090569
H -0.873709765 12.494031876 -5.104867569
H -4.374358765 14.845603876 -2.162279569
H -3.040170765 13.011671876 -1.221702569
H -1.106773765 11.341870876 -6.417194569
H -3.351595765 13.187910876 -5.512739569
H -0.486680765 10.791095876 -4.858107569
H -4.418704765 14.070385876 -3.744599569
H -0.508487765 11.100604876 -2.503280569
H -5.246268765 13.325573876 -2.380831569
H -3.564925765 11.935410876 -6.729323569
H -1.396495765 11.773303876 -0.277330569
H -4.649022765 12.003249876 -5.333707569
H -2.884629765 10.177669876 -5.129978569
H -0.455284765 10.287619876 -0.136493569
H -1.121693765 8.903001876 -3.544156569
H -9.165567765 12.142893876 -8.268868569
H -3.224143765 9.808925876 -7.388061569
H -4.561201765 11.571711876 -0.848278569
H -8.866464765 12.024422876 -6.539358569
H -0.261088765 8.661712876 -2.017346569
H -2.215589765 10.214829876 -0.110910569
H -6.654852765 11.712382876 -2.104321569
H -6.743883765 11.708754876 -8.717741569
H -4.333225765 8.709140876 -8.226592569
H -7.418335765 11.376076876 -4.173023569
H -6.353830765 11.443757876 -7.014576569
H -10.140853765 11.05076687 -7.284131569
H -2.017318765 8.467101876 -2.092484569
H -2.623431765 8.734508876 -8.656709569
H -6.948143765 11.111055876 -0.461104569
H -4.781495765 9.862836876 -0.572128569
H -8.946085765 10.700652876 -4.680349569
H -5.963610765 10.208898876 -8.207791569
H -8.892328765 10.817316876 -2.227759569
H -0.734619765 8.447972876 -5.891986569
H -8.398208765 9.835917876 -8.633952569
H -1.780055765 4.915435876 -3.459055569
H -0.510837765 7.192583876 -7.118445569
H -2.053850765 5.678423876 -1.821298569
H -8.976703765 9.144595876 -2.801401569
H -10.529652765 8.777948876 -8.186506569
H -2.992870765 4.929888876 -6.843038569
H -6.567667765 8.700295876 -0.262800569
H -3.484870765 6.983676876 -4.390814569
H -0.803119765 6.740799876 -5.434035569
H -4.388053765 5.679643876 -7.631526569
H -1.894017546 3.901548362 -2.041637741
H -6.838664765 8.191898876 -9.208241569
H -8.319766765 8.802779876 -0.511817569
H -5.981270765 7.709314876 -6.924028569
H -9.609854765 7.568821876 -4.099581569
H -11.709506765 7.886129876 -7.221634569
H -3.409250765 4.043592876 -4.754586569
H -2.792869765 5.751109876 -8.394324569
H -11.236020765 7.326188876 -4.755852569
H -8.105758765 7.158675876 -2.392171569
H -10.256064765 7.076290876 -7.800364569
H -6.355258765 5.434835876 1.265402431
H -6.029541765 7.154058876 1.044036431
H -8.164462765 7.050048876 -8.979849569
H -7.249321765 6.160838876 -5.370185569
H -9.886183765 6.359958876 -5.346150569
H -6.502109765 6.468139876 -9.030607569
H -4.925128765 4.949292876 -4.708913569
H -8.259610765 4.820562876 -2.548916569
H -7.056551765 4.371460876 -3.753836569
H -7.508157765 6.514760876 -0.882459569
H -4.814178765 3.383764876 -3.916362569
H -8.286908765 5.707471876 -6.724044569
H -5.080907765 6.224948876 2.192770431
H -6.566401765 5.335895876 -6.777944569
H -3.851156765 7.920631876 -0.258551569
H -7.968050765 3.119164876 -2.902973569
H -4.020796765 4.929258876 0.400338431
H -7.817667765 4.322961876 -0.000427569
H -5.723452765 3.219891876 -1.935590569
H -2.538122765 6.754238876 -0.366907569
H -3.166891765 7.212050876 1.209241431
H -6.356337765 3.425260876 0.442645431
H -7.622517765 2.649360876 -0.506608569
H -10.363919765 9.352643876 -5.753883569

Table S40. Final coordinates for 4b

Np -1.667013 0.016510 -1.666849
Si -4.237894 2.712787 -2.212610
Si -2.630525 -3.541520 -2.300058
Si 1.825609 0.676422 -0.582227
Si -2.797772 0.165158 2.075127
N -3.042559 1.498729 -2.749688
N -0.888230 0.200361 -4.199910
N -1.692741 -2.061479 -2.619139
N 0.478591 0.762773 -1.746156
N -2.603335 -0.226674 0.353172
C -5.287503 5.294926 -2.963825
C -4.328475 4.200947 -3.456994
C -6.028450 1.985646 -2.154813
C -6.107572 0.750196 -1.253598
C -3.097263 1.277341 -4.206813
C -1.704303 1.261706 -4.814473
C -1.138970 -1.106051 -4.832182
C -0.883449 -2.230035 -3.841701
C -1.529386 -5.155174 -2.338806

C -1.613467 -6.071489 -3.564807
C -3.759802 3.277897 -0.441504
C -2.552736 4.224395 -0.419891
C -2.988786 4.816992 -3.876804
C 2.422053 2.431735 -0.023955
C 1.260354 3.308538 0.453215
C -1.838485 1.731775 2.529788
C -4.631604 0.422249 2.496704
C -2.172742 -1.209742 3.229752
C 0.543361 0.551286 -4.203716
C 0.871937 1.425573 -3.002371
C 3.371487 -0.170524 -1.387315
C 4.564268 -0.256134 -0.423374
C 1.182341 -0.271460 0.964388
C 1.166167 -1.793604 0.771268
C 3.125837 -1.527938 -2.056317
C 1.904947 0.081677 2.272208
C 3.280158 3.203586 -1.036184
C -4.151023 -3.672747 -3.494227
C -5.057612 -2.438937 -3.386965
C -3.281438 -3.601599 -0.496748
C -2.268618 -3.734293 0.616456
C -3.840531 -3.910662 -4.980158
C -4.586329 -3.715780 -0.200506
C -0.059373 -4.897967 -1.979186
C -4.913806 3.872666 0.377470
C -6.627974 1.676199 -3.532588
H 3.662461 0.528309 -2.187031
H -1.032282 -6.987810 -3.394251
H -2.640157 -6.378310 -3.786431
H -5.953048 -2.552792 -4.012062
H -5.392825 -2.232953 -2.368139
H -1.970867 -5.724351 -1.506003
H -1.200689 -5.599974 -4.464257
H -4.713009 -4.546870 -3.129426
H 0.455319 -5.841249 -1.753006
H -4.530135 -1.543715 -3.735360
H 0.645512 -2.290929 1.599396
H -4.773351 -4.020680 -5.549337
H 2.182893 -2.200037 0.744684
H -1.589499 -4.576120 0.440845
H -1.086968 -3.176939 -4.366828
H 0.057445 -4.241050 -1.112904
H 0.477980 -4.432305 -2.811371
H -1.648918 -2.837036 0.704997
H 1.470078 -0.468340 3.117055
H 2.983025 -2.323930 -1.319575
H 0.670653 -2.094953 -0.158288
H 2.967393 -0.184769 2.231418
H -3.308411 -3.059592 -5.419305
H -3.241600 -4.804215 -5.162845
H 4.367806 -0.954624 0.398189
H 0.189384 -2.265534 -3.599094
H 3.980566 -1.818875 -2.681418
H -2.229670 -0.867059 4.269671
H -2.757542 -3.902246 1.582239
H 5.462456 -0.619542 -0.940383
H 2.235153 -1.517632 -2.691391
H 0.135654 0.052241 1.071765
H -0.530914 -1.218612 -5.745803
H 1.844727 1.147519 2.512239
H -1.131545 -1.474369 3.022180
H -5.237002 -0.433208 2.178125
H -3.274461 -0.984970 0.162097
H -2.191044 -1.138636 -5.129442
H -2.775324 -2.118970 3.148474
H 4.815773 0.711737 0.022624
H 1.123136 -0.372201 -4.127967
H -5.373650 -3.674833 -0.946601
H -4.759073 0.532528 3.579796
H -4.921536 -3.881410 0.825009
H 3.064396 2.224697 0.846131
H -5.037248 1.319201 2.019898
H 0.818999 1.038557 -5.154258
H 0.661986 2.828262 1.233653
H 1.947683 1.649512 -3.053870
H -3.611826 0.335410 -4.459975
H -1.735304 1.138630 -5.910437
H -2.121353 2.043941 3.541467
H -5.490417 -0.059837 -1.656008
H 4.149512 2.632525 -1.378142
H 0.582032 3.542113 -0.374258
H 0.370966 2.401106 -3.106950
H -0.759195 1.556203 2.534790
H 1.622110 4.262741 0.859116
H -1.225104 2.221110 -4.601579
H -6.056293 0.900202 -4.054570
H -7.136795 0.373364 -1.180240
H 2.703416 3.493097 -1.921818
H -3.667946 2.054620 -4.733765
H -2.049489 2.562876 1.853314
H 3.659057 4.133387 -0.590969
H -5.754254 0.947984 -0.237575
H -7.656587 1.302704 -3.437679
H -3.462836 2.336810 0.045902
H -1.708747 3.836504 -0.999672
H -6.663488 2.556257 -4.183361
H -2.197364 4.397603 0.604554
H -2.289204 4.067812 -4.258195
H -6.644970 2.775710 -1.698729
H -2.494494 5.322831 -3.042122
H -5.769911 3.194985 0.449436
H -2.811053 5.206520 -0.831083
H -4.587311 4.097285 1.401665

H -4.783887 3.778741 -4.365717
H -3.132328 5.565832 -4.667551
H -5.278066 4.811838 -0.053224
H -4.886844 5.814479 -2.086419
H -6.270436 4.897192 -2.690600
H -5.447056 6.055984 -3.739450

Table S41. Final coordinates for TS B1

Np 0.167886 -0.125593 0.106375
Si 0.323099 1.635311 3.386701
Si 0.052860 2.021758 -3.060736
Si 0.134228 -3.860355 -0.171622
Si -3.743815 0.050847 0.211561
N 1.027911 1.100636 1.830848
N 2.799645 -0.118704 -0.062842
N 0.687212 0.807353 -1.888194
N 0.898851 -2.296509 0.191469
N -2.002363 0.398738 0.181374
C 1.047004 1.814654 6.188608
C 1.618562 1.428143 4.815986
C -0.149379 3.509239 3.381958
C -1.157142 3.847661 2.279084
C 2.387346 1.630119 1.615601
C 3.335456 0.538696 1.140825
C 3.097178 0.633575 -1.307989
C 2.003917 0.400522 -2.318979
C -0.113934 3.789938 -2.295592
C -1.490796 4.090174 -1.688999
C -1.287189 0.633001 3.702154
C -1.011660 -0.810928 4.138757
C 2.309269 0.061430 4.894985
C -0.136565 -4.955659 1.399624
C -0.877986 -4.206524 2.510460
C -4.140696 -1.335153 1.443261
C -4.666291 1.618982 0.756539
C -4.403117 -0.468875 -1.486829
C 3.225716 -1.527722 -0.158640
C 2.296011 -2.426535 0.646848
C 1.256492 -4.923917 -1.341408
C 0.628822 -6.284941 -1.680351
C -1.586952 -3.477061 -0.951383
C -1.498337 -3.182924 -2.454281
C 1.758663 -4.236225 -2.617168
C -2.658763 -4.546387 -0.696571
C 1.130174 -5.607943 1.970654
C -1.648455 1.600631 -3.842481
C -2.011172 2.607214 -4.945545
C 1.531626 1.978375 -4.291430
C 2.310978 3.237036 -4.615772
C -1.802052 0.165652 -4.351186
C 1.451700 1.033655 -5.479169
C 0.986576 4.098451 -1.274960
C -2.279858 1.293503 4.669920
C 1.039997 4.476613 3.325858
H 2.144823 -5.134293 -0.725739
H -1.519417 5.101288 -1.261943
H -2.297624 4.023935 -2.424522
H -3.031003 2.435312 -5.314186
H -1.958527 3.647836 -4.608444
H 0.024058 4.467147 -3.153237
H -1.736295 3.395296 -0.877941
H -2.361940 1.731425 -3.015380
H 0.923564 5.137016 -0.924230
H -1.339640 2.510503 -5.806513
H -2.452595 -2.807179 -2.844008
H -2.842260 -0.039135 -4.636624
H -1.263556 -4.092165 -3.018065
H 1.787310 3.843851 -5.369897
H 2.159014 1.238993 -3.383286
H 1.993440 3.949934 -1.679435
H 0.894187 3.448830 -0.397876
H 2.474790 3.881807 -3.748003
H -3.616110 -4.251816 -1.145922
H 0.966805 -4.145626 -3.366584
H -0.728078 -2.442837 -2.692923
H -2.378664 -5.507266 -1.143432
H -1.186251 -0.017881 -5.237583
H -1.518531 -0.563970 -3.589860
H -0.263082 -6.172039 -2.307533
H 2.060012 -0.578857 -2.816305
H 2.571570 -4.812231 -3.079021
H -5.473158 -0.698815 -1.429851
H 3.295049 2.996715 -5.042210
H 1.333302 -6.913641 -2.241238
H 2.133512 -3.226806 -2.423929
H -1.925903 -2.554116 -0.451201
H 4.090184 0.354639 -1.695523
H -2.837824 -4.721482 0.368158
H 0.796051 1.425218 -6.269472
H -3.889482 -1.360554 -1.858012
H -4.467237 2.458002 0.081089
H -1.910782 1.402915 0.381369
H 3.121766 1.698271 -1.053402
H -4.266374 0.327054 -2.225091
H 0.332953 -6.846184 -0.787977
H 3.167588 -1.825365 -1.208650
H 1.070568 0.045299 -5.202840
H -5.748451 1.447433 0.758730
H 2.441420 0.889616 -5.935101
H -0.791006 -5.768623 1.048634
H -4.377971 1.922027 1.768569
H 4.275890 -1.634685 0.161014
H -1.797536 -3.725113 2.160674
H 2.684804 -3.451839 0.556828

H 2.386835 2.451149 0.881289
H 4.348319 0.932545 0.951188
H -5.217567 -1.537711 1.458687
H -0.707884 3.719210 1.288906
H 1.662678 -6.217107 1.233156
H -0.245094 -3.421013 2.934335
H 2.388572 -2.180140 1.717338
H -3.634829 -2.269466 1.181197
H -1.153425 -4.881002 3.332243
H 3.417901 -0.218496 1.925686
H 1.590116 4.384982 2.382520
H -1.490556 4.891901 2.346930
H 1.833214 -4.858279 2.351775
H 2.827051 2.064683 2.524788
H -3.834524 -1.057806 2.456599
H 0.882254 -6.265737 2.814624
H -2.052615 3.217974 2.320116
H 0.700522 5.518900 3.395396
H -1.772046 0.588434 2.715351
H -0.268642 -1.302551 3.502461
H 1.752051 4.314505 4.141906
H -1.925303 -1.418048 4.106857
H 2.716490 -0.252930 3.929918
H -0.651225 3.663758 4.349691
H 1.619155 -0.722000 5.222673
H -2.586755 2.292061 4.344838
H -0.637432 -0.851983 5.167772
H -3.190845 0.686828 4.760100
H 2.400472 2.168457 4.587703
H 3.138151 0.081682 5.615247
H -1.864080 1.392121 5.678663
H 0.285155 1.101344 6.522494
H 0.590093 2.809839 6.190192
H 1.835527 1.817867 6.953064

Table S42. Final coordinates for III

C	1.014589000	1.586729000	6.249066000
C	1.552456000	1.214153000	4.861357000
Si	0.278700000	1.634100000	3.437848000
C	-0.088629000	3.539708000	3.638363000
C	-0.987403000	4.072491000	2.521878000
N	0.956058000	1.204310000	1.823943000
C	2.304029000	1.765180000	1.613356000
C	3.282417000	0.672995000	1.210314000
N	2.810778000	-0.011242000	-0.007478000
C	3.242668000	0.695975000	-1.226711000
C	2.220178000	0.511814000	-2.337754000
N	0.890800000	1.003470000	-1.922507000
Si	0.194606000	2.128012000	-3.146240000
C	-0.929181198	1.284552577	-4.493998556
C	-0.163273469	0.501516765	-5.566397610
C	-1.405762000	0.693488000	3.628671000
C	-1.217949000	-0.794874000	3.940469000
C	2.103362000	-0.214726000	4.868120000
Np	0.114880000	0.038785000	0.019458000
N	0.855063000	-2.130491000	0.224123000
Si	0.127271000	-3.726053000	-0.204050000
C	-0.047039000	-4.936035000	1.319902000
C	-0.846163000	-4.318072000	2.468942000
N	-2.121879000	0.299400000	-0.192013000
Si	-3.830111000	-0.036906000	0.211982000
C	-3.981329000	-1.290600000	1.641087000
C	-4.764604000	1.545864000	0.753543000
C	-4.807663000	-0.738026000	-1.277800000
C	3.215248000	-1.428151000	-0.024936000
C	2.223022000	-2.275741000	0.758308000
C	1.276971000	-4.673897000	-1.467689000
C	0.724298000	-6.053219000	-1.848695000
C	-1.659334000	-3.404637000	-0.893693000
C	-1.657398000	-2.892464000	-2.336289000
C	1.666970000	-3.889404000	-2.723795000
C	-2.609230000	-4.601431000	-0.759187000
C	1.260278000	-5.535496000	1.850556000
C	1.631387560	3.031388568	-4.120956298
C	1.060941094	4.003004982	-5.161369038
C	-0.837097284	3.474322099	-2.287822959
C	-1.860265482	4.080551743	-3.199961630
C	2.644353530	3.746730339	-3.223887719
C	-0.001653165	4.287217563	-1.346049995
C	-2.019333313	0.406008642	-3.877714137
C	-2.374745000	1.328724000	4.633118000
C	1.145281000	4.434710000	3.796383000
H	2.202919000	-4.843090000	-0.897107000
H	-0.843242467	0.164011896	-6.360761136
H	0.622489736	1.095447964	-6.045012117
H	1.866402850	4.479190582	-5.737026335
H	0.396202258	3.512115616	-5.880329401
H	-1.417538795	2.132733722	-4.997156526
H	0.307350906	-0.397129638	-5.151857733
H	2.170643824	2.243402816	-4.667244253
H	-2.744475872	0.078229580	-4.635298663
H	0.489411217	4.808858060	-4.684721559
H	-2.658333000	-2.564179000	-2.641947000
H	3.501392890	4.107403919	-3.809191822
H	-1.347925000	-3.675222000	-3.037827000
H	-1.407809439	4.850630205	-3.848863638
H	2.419294031	0.073711762	-3.327070799
H	-2.577286340	0.914473034	-3.084240998
H	-1.582877097	-0.495821359	-3.436594901
H	-2.343054148	3.357101242	-3.862777925
H	-3.609104000	-4.342417000	-1.132514000
H	0.831326000	-3.800902000	-3.425218000
H	-0.985289000	-2.040571000	-2.477225000
H	-2.266324000	-5.464046000	-1.342092000

H 2.201591642 4.621514817 -2.734921058
H 3.034220170 3.096041400 -2.434573170
H -0.189610000 -5.967773000 -2.448373000
H 2.483482000 -4.390768000 -3.261501000
H -5.876466000 -0.780740000 -1.035099000
H -2.649556685 4.595555312 -2.633740271
H 1.450301000 -6.612868000 -2.454568000
H 2.001425000 -2.874070000 -2.489593000
H -2.060279000 -2.597348000 -0.262854000
H 4.246479000 0.358372000 -1.535619000
H -2.728804000 -4.932005000 0.277031000
H 0.290905309 5.248647084 -1.801337954
H -4.485036000 -1.751454000 -1.535131000
H -4.848330000 2.273070000 -0.061410000
H -2.134996000 0.987052000 -0.961357000
H 3.310368000 1.762826000 -0.996963000
H -4.696374000 -0.112140000 -2.169835000
H 0.487654000 -6.671533000 -0.976231000
H 3.217778000 -1.769510000 -1.063801000
H 0.917377082 3.777695913 -1.045178300
H -5.783915000 1.286641000 1.064812000
H -0.554905289 4.546310517 -0.432702999
H -0.639980000 -5.767076000 0.909477000
H -4.274184000 2.041375000 1.597776000
H 4.242161000 -1.542225000 0.361867000
H -1.803083000 -3.899401000 2.138933000
H 2.594281000 -3.311065000 0.723325000
H 2.294361000 2.550554000 0.839673000
H 4.302720000 1.065594000 1.063465000
H -5.019253000 -1.639183000 1.698634000
H -0.453675000 4.082437000 1.566220000
H 1.866383000 -6.001733000 1.066017000
H -0.283769000 -3.509094000 2.946573000
H 2.262689000 -1.994115000 1.822412000
H -3.348331000 -2.171422000 1.498343000
H -1.067325000 -5.062319000 3.246420000
H 3.325210000 -0.061689000 2.018342000
H 1.746821000 4.464341000 2.880106000
H -1.311272000 5.102459000 2.725791000
H 1.881275000 -4.779077000 2.343593000
H 2.715909000 2.253253000 2.509244000
H -3.732616000 -0.837415000 2.604515000
H 1.053671000 -6.311796000 2.600306000
H -1.888391000 3.465766000 2.381715000
H 0.847825000 5.470857000 4.010840000
H -1.871888000 0.773090000 2.634038000
H -0.522449000 -1.280311000 3.247139000
H 1.802175000 4.115733000 4.612957000
H -2.170995000 -1.335436000 3.883863000
H 2.425143000 -0.540817000 3.874433000
H -0.654511000 3.594653000 4.580499000
H 1.360351000 -0.937685000 5.219272000
H -2.631544000 2.361026000 4.375987000
H -0.829573000 -0.944838000 4.953819000
H -3.315500000 0.762622000 4.673800000
H 2.403426000 1.881621000 4.659001000
H 2.969787000 -0.297627000 5.538860000
H -1.966435000 1.334488000 5.650499000
H 0.179640000 0.940044000 6.543374000
H 0.662448000 2.622372000 6.302700000
H 1.792993000 1.470511000 7.016122000
H -1.534852075 3.075403308 -1.566433986

Table S43. Final coordinates for TS B2

Np -0.447933 -4.114551 -4.785221
Np 6.019120 -5.761149 -9.781857
Si -2.739659 -3.362604 -1.650126
Si -1.341952 -0.671875 -6.101041
Si 2.609979 -4.006185 -2.086552
Si -2.797203 -6.994549 -5.467197
Si 6.519070 -8.775123 -12.250570
Si 4.262923 -3.798489 -12.453440
Si 4.382914 -8.277108 -7.567004
Si 9.737596 -5.319460 -9.589929
N -1.674539 -4.007605 -2.925758
N -0.656224 -2.316713 -6.222140
N 1.740041 -4.285499 -3.637912
N 0.850480 -4.622464 -6.929223
N -1.333603 -6.001337 -5.781412
N 6.607717 -7.568076 -10.949060
N 5.191506 -3.960921 -10.940781
N 4.675562 -6.600328 -8.106492
N 5.743260 -3.797035 -8.109229
N 8.067326 -5.184092 -8.959039
C -1.623493 -9.655391 -5.055403
C -5.064557 -6.785006 -3.649135
C 1.884762 -1.381223 -1.031980
C 1.236376 -6.272219 -1.033479
C 4.915138 -5.219225 -0.806599
C 1.775765 -4.535736 0.700050
C 1.434186 -4.771168 -0.778314
C 5.290686 -4.396415 -3.144988
C 4.262951 -4.993564 -2.177627
C -2.897846 -0.386738 -8.587964
C -2.845848 0.671132 -3.991950
C 1.319742 0.530675 -6.536750
C -0.417594 0.233729 -3.529341
C -1.670673 -0.283798 -4.244202
C -0.675406 2.069107 -6.760275
C -0.161190 0.630117 -6.919991
C -0.271511 -2.584062 -7.621258
C 1.022244 -3.380340 -7.688842
C -1.914909 -2.019016 -0.600099
C -3.202631 -4.795259 -0.493117

C -4.329821 -2.635641 -2.378526
C -3.961164 -1.670665 -6.708905
C -3.015219 -0.520649 -7.063427
C -3.654915 -6.733966 -8.277526
C -4.967165 -5.076677 -5.489140
C -4.134685 -5.958146 -4.549675
C 3.448638 -1.382170 -2.988811
C 3.009733 -2.151378 -1.733017
C 2.148222 -4.016155 -4.893666
C 1.821716 -4.971685 -5.927855
C 0.266041 -5.735639 -7.675763
C -0.456692 -6.697151 -6.739246
C -1.612313 -8.080039 -3.093265
C -2.371820 -8.517647 -4.349016
C -3.481339 -7.732473 -7.126352
C -4.770190 -8.539178 -6.905597
C 10.980905 -6.797259 -7.401508
C 10.849332 -6.196026 -12.143294
C 6.956691 -8.118600 -6.252658
C 2.616095 -9.715059 -5.769677
C 2.400350 -8.580019 -9.610699
C 3.733632 -7.685487 -4.818467
C 3.151571 -8.313152 -6.088612
C 3.444744 -10.759452 -8.947367
C 3.674224 -9.246568 -9.079518
C 6.280053 -2.427747 -14.010361
C 2.719770 -5.702524 -14.047963
C 2.325731 -1.842960 -11.313543
C 1.947924 -5.356328 -11.684623
C 3.166076 -5.372413 -12.616612
C 2.426925 -2.031398 -13.813644
C 3.232676 -2.154593 -12.510130
C 5.574039 -2.692605 -10.294972
C 5.106671 -2.686437 -8.845028
C 4.799714 -8.785746 -13.038861
C 6.871705 -10.520708 -11.591787
C 7.816890 -8.440941 -13.596062
C 6.306181 -4.946060 -14.114580
C 5.424696 -3.701375 -13.991544
C 10.296443 -2.408663 -9.281004
C 9.430035 -4.127425 -12.202251
C 9.649600 -5.477824 -11.507553
C 6.771884 -9.972283 -7.926582
C 6.001231 -9.117814 -6.915219
C 3.933275 -5.434172 -7.659068
C 4.851477 -4.370774 -7.074642
C 7.061871 -3.417235 -7.573771
C 7.989081 -4.622938 -7.596095
C 9.947520 -8.184549 -9.218991
C 10.666182 -6.870716 -8.901497
C 10.843674 -3.819669 -9.033651
C 12.269438 -3.927123 -9.598576
H -5.067695 -9.059731 -7.825735
H -4.658722 -9.299398 -6.124495
H -5.609149 -7.895672 -6.619823
H -5.646817 -7.516619 -4.220807
H -3.909793 -7.252995 -9.211360
H -2.720370 -8.460106 -7.445939
H -5.787457 -6.132264 -3.141439
H -5.636786 -5.674388 -6.117164
H -4.523231 -7.335064 -2.872954
H -4.458595 -6.019526 -8.076814
H -3.344801 -8.919720 -4.029582
H -2.745920 -6.151775 -8.460497
H -5.603289 -4.385901 -4.920071
H -2.181922 -10.055207 -5.907732
H -4.342509 -4.473399 -6.152592
H -3.558910 -5.283364 -3.901663
H -1.443424 -10.491595 -4.366210
H -2.172123 -7.345442 -2.502260
H -3.890438 -0.268945 -9.043198
H -4.905415 -3.401137 -2.906472
H -0.998371 -7.395083 -7.391557
H -2.446070 -1.275733 -9.041847
H -1.397155 -8.929778 -2.431624
H -0.644074 -9.333835 -5.425835
H -0.459810 -5.297055 -8.366742
H -4.949630 -1.535594 -7.167922
H -4.119420 -1.826293 -3.083368
H -1.059892 -3.136570 -8.158359
H -3.558993 -2.622760 -7.071383
H -2.300513 0.479122 -8.891090
H -0.651156 -7.624724 -3.361057
H -4.960445 -2.224944 -1.581938
H 1.026236 -6.261507 -8.272385
H 0.282713 -7.330207 -6.222593
H -0.108388 -1.668831 -8.209693
H -4.113139 -1.776077 -5.629321
H 1.312625 -3.601600 -8.724976
H -3.746896 -5.582268 -1.024973
H -3.465443 0.412885 -6.693210
H 0.444501 -6.681518 -0.392703
H -3.846172 -4.440044 0.319375
H 0.965421 -6.477932 -2.072744
H 1.825454 -2.795359 -7.234239
H -1.714135 2.179446 -7.089936
H -1.036383 -2.403556 -0.073688
H 1.530440 -5.922150 -5.465142
H -1.552824 -4.998938 -2.671613
H -2.313761 -5.243945 -0.036371
H -1.599863 -1.164550 -1.205070
H 2.149166 -6.839271 -0.814388
H -3.791496 0.292530 -4.391252
H 2.930399 -5.418309 -6.671398

H -1.933650 -1.253803 -3.793470
H 1.699823 -0.491528 -6.634334
H -0.070949 2.769467 -7.352066
H 0.953462 -4.879134 1.341031
H -2.622688 -1.650599 0.151468
H 1.935447 1.177755 -7.175989
H 2.688842 -3.099145 -5.147331
H -0.625377 2.408940 -5.719896
H 4.289229 -5.819813 -0.141445
H 5.133269 -4.274530 -0.293566
H -2.669858 1.653882 -4.443998
H 0.441548 -0.428886 -3.668083
H 1.494839 0.842669 -5.502642
H -2.988025 0.835079 -2.915719
H 1.957777 -3.485397 0.942498
H 0.959846 -1.399131 -1.614617
H 1.650703 -1.782179 -0.043477
H 0.477303 -4.270700 -0.990169
H 2.665994 -5.094505 1.004883
H -0.129476 1.224398 -3.896413
H 5.870700 -5.749071 -0.916837
H -0.586419 0.336335 -2.449995
H 5.709221 -3.461536 -2.754376
H 2.161206 -0.326864 -0.897963
H 3.965113 -5.974371 -2.571689
H 2.623801 -1.298924 -3.704999
H 3.871857 -2.186633 -1.047780
H 4.865247 -4.183330 -4.130558
H 6.132921 -5.085484 -3.292811
H 4.291628 -1.849300 -3.505872
H 3.750180 -0.358540 -2.730351
H -0.224999 0.403670 -7.994871
H 12.913817 -3.146609 -9.172483
H 12.740783 -4.890564 -9.380432
H 12.283317 -3.791007 -10.685643
H 11.781997 -5.643473 -11.985440
H 10.961238 -1.654029 -8.839289
H 10.935236 -3.936845 -7.943124
H 10.711501 -6.288210 -13.228344
H 10.319016 -3.492370 -12.124381
H 10.998909 -7.204650 -11.747484
H 10.216883 -2.180036 -10.347612
H 11.624940 -6.879834 -9.441610
H 9.305941 -2.261810 -8.844107
H 9.228683 -4.262856 -13.271884
H 11.590144 -5.925277 -7.142461
H 8.587435 -3.573240 -11.776223
H 8.759381 -6.097068 -11.699385
H 11.534475 -7.687122 -7.072836
H 9.752360 -8.308397 -10.290334
H 6.947794 -2.414839 -14.882099
H 8.830697 -8.492782 -13.184854
H 8.963884 -4.305370 -7.200545
H 6.915668 -2.358020 -13.120454
H 10.538486 -9.052416 -8.896784
H 10.065150 -6.753627 -6.800863
H 7.474070 -2.644273 -8.226502
H 6.985805 -4.871151 -14.974529
H 7.690486 -7.455055 -14.051772
H 6.664966 -2.539494 -10.318934
H 6.925369 -5.086487 -13.222511
H 5.672613 -1.517639 -14.056068
H 8.989541 -8.241423 -8.693071
H 7.745972 -9.193107 -14.390451
H 6.963121 -2.985886 -6.564056
H 7.618182 -5.368466 -6.876149
H 5.149596 -1.809906 -10.792499
H 5.717703 -5.859179 -14.246734
H 5.306528 -1.726652 -8.340452
H 7.842482 -10.576451 -11.087913
H 4.764730 -3.672943 -14.871435
H 1.990687 -9.694734 -4.867239
H 6.891707 -11.233989 -12.424037
H 2.005695 -10.120080 -6.579995
H 4.025599 -2.852320 -8.834765
H 3.039211 -2.190669 -14.706637
H 4.011636 -8.894864 -12.287711
H 5.477263 -4.852156 -6.317443
H 7.500343 -7.748409 -10.467730
H 6.107235 -10.851935 -10.883540
H 4.600078 -7.880110 -13.617144
H 3.424531 -10.430980 -5.577840
H 3.558961 -5.797107 -14.743392
H 4.295142 -3.557855 -6.583471
H 3.821010 -6.195243 -12.284374
H 2.860599 -1.872600 -10.360391
H 1.979107 -1.032487 -13.899771
H 2.972642 -7.617972 -4.029081
H 4.723955 -9.635769 -13.726211
H 1.888822 -0.839844 -11.409683
H 3.317568 -4.998033 -8.467308
H 1.601639 -2.751747 -13.846367
H 2.542117 -7.507390 -9.773858
H 1.565428 -8.704039 -8.909051
H 2.053987 -4.929958 -14.447324
H 2.219318 -5.103877 -10.653525
H 1.490557 -2.547108 -11.243738
H 2.165069 -6.649332 -14.074935
H 4.124943 -6.677873 -4.987844
H 7.418777 -7.470630 -7.005190
H 6.459834 -7.469260 -5.525644
H 2.298639 -7.703095 -6.412862
H 4.551780 -8.294984 -4.417828
H 1.204794 -4.624619 -12.020094

H	2.079801	-9.022910	-10.563589
H	1.449214	-6.333343	-11.668033
H	2.537377	-10.980633	-8.376836
H	7.770329	-8.641260	-5.731880
H	4.465343	-9.094176	-9.830080
H	7.026465	-9.400886	-8.823953
H	5.634312	-9.792709	-6.125574
H	4.267129	-11.285788	-8.455726
H	3.310138	-11.216362	-9.936812
H	6.205209	-10.845276	-8.258268
H	7.710533	-10.341764	-7.492276
H	3.998580	-1.364599	-12.544713

Table S44. Final coordinates for IV

C	1.014589000	1.586729000	6.249066000
C	1.552456000	1.214153000	4.861357000
Si	0.278700000	1.634100000	3.437848000
C	-0.088629000	3.539708000	3.638363000
C	-0.987403000	4.072491000	2.521878000
N	0.956058000	1.204310000	1.823943000
C	2.304029000	1.765180000	1.613356000
C	3.282417000	0.672995000	1.210314000
N	2.810778000	-0.011242000	-0.007478000
C	3.237753586	0.685160194	-1.196383847
C	2.139121376	0.476934972	-2.381908630
N	0.890800000	1.003470000	-1.922507000
Si	0.225671156	2.192416490	-3.101834400
C	-0.979248499	1.443281370	-4.435153487
C	-0.287316189	0.643992604	-5.545183490
C	-1.405762000	0.693488000	3.628671000
C	-1.217949000	-0.794874000	3.940469000
C	2.103362000	-0.214726000	4.868120000
Np	0.114880000	0.038785000	0.019458000
N	0.855063000	-2.130491000	0.224123000
Si	0.127271000	-3.726053000	-0.204050000
C	-0.047039000	-4.936035000	1.319902000
C	-0.846163000	-4.318072000	2.468942000
N	-2.121879000	0.299400000	-0.192013000
Si	-3.830111000	-0.036906000	0.211982000
C	-3.981329000	-1.290600000	1.641087000
C	-4.764604000	1.545864000	0.753543000
C	-4.807663000	-0.738026000	-1.277800000
C	3.215248000	-1.428151000	-0.024936000
C	2.223022000	-2.275741000	0.758308000
C	1.276971000	-4.673897000	-1.467689000
C	0.724298000	-6.053219000	-1.848695000
C	-1.659334000	-3.404637000	-0.893693000
C	-1.657398000	-2.892464000	-2.336289000
C	1.666970000	-3.889404000	-2.723795000
C	-2.609230000	-4.601431000	-0.759187000
C	1.260278000	-5.535496000	1.850556000
C	1.683806150	3.037316124	-4.097067682
C	1.140595826	4.062478665	-5.099998689
C	-0.705915430	3.573528170	-2.186127392
C	-1.717889959	4.255971098	-3.055934093
C	2.758844876	3.674995647	-3.213776958
C	0.198581933	4.317348396	-1.251077542
C	-2.099683184	0.612275138	-3.807353898
C	-2.374745000	1.328724000	4.633118000
C	1.145281000	4.434710000	3.796383000
H	2.202919000	-4.843090000	-0.897107000
H	-1.006174404	0.362768321	-6.327127577
H	0.517157284	1.204517773	-6.032916918
H	1.955555727	4.506605594	-5.687786337
H	0.430420061	3.625576451	-5.810295058
H	-1.432944253	2.328395736	-4.906115334
H	0.143482366	-0.288489373	-5.163364245
H	2.163407924	2.233526491	-4.675041521
H	-2.862096282	0.342406727	-4.551062488
H	0.627903437	4.887478131	-4.590331347
H	-2.658333000	-2.564179000	-2.641947000
H	3.618586216	4.001194546	-3.815127019
H	-1.347925000	-3.675222000	-3.037827000
H	-1.240851723	5.014602728	-3.700700482
H	2.528669610	0.088624635	-3.341769644
H	-2.606869965	1.132409537	-2.987799739
H	-1.702449224	-0.322110309	-3.398078224
H	-2.257913916	3.575607003	-3.720215501
H	-3.609104000	-4.342417000	-1.132514000
H	0.831326000	-3.800902000	-3.425218000
H	-0.985289000	-2.040571000	-2.477225000
H	-2.266324000	-5.464046000	-1.342092000
H	2.378846847	4.561423599	-2.693948763
H	3.132947925	2.985894517	-2.449842346
H	-0.189610000	-5.967773000	-2.448373000
H	2.483482000	-4.390768000	-3.261501000
H	-5.876466000	-0.780740000	-1.035099000
H	-2.461724320	4.800590735	-2.456838275
H	1.450301000	-6.612868000	-2.454568000
H	2.001425000	-2.874070000	-2.489593000
H	-2.060279000	-2.597348000	-0.262854000
H	4.283556080	1.009573598	-1.408057136
H	-2.728804000	-4.932005000	0.277031000
H	0.531905191	5.271367937	-1.693758727
H	-4.485036000	-1.751454000	-1.535131000
H	-4.848330000	2.273070000	-0.061410000
H	-2.134996000	0.987052000	-0.961357000
H	-4.696374000	-0.112140000	-2.169835000
H	0.487654000	-6.671533000	-0.976231000
H	3.217778000	-1.769510000	-1.063801000
H	1.095553224	3.751184965	-0.987156802
H	-5.783915000	1.286641000	1.064812000
H	-0.314583823	4.585550661	-0.317188760
H	-0.639980000	-5.767076000	0.909477000

H -4.274184000 2.041375000 1.597776000
H 4.242161000 -1.542225000 0.361867000
H -1.803083000 -3.899401000 2.138933000
H 2.594281000 -3.311065000 0.723325000
H 2.294361000 2.550554000 0.839673000
H 4.302720000 1.065594000 1.063465000
H -5.019253000 -1.639183000 1.698634000
H -0.453675000 4.082437000 1.566220000
H 1.866383000 -6.001733000 1.066017000
H -0.283769000 -3.509094000 2.946573000
H 2.262689000 -1.994115000 1.822412000
H -3.348331000 -2.171422000 1.498343000
H -1.067325000 -5.062319000 3.246420000
H 3.325210000 -0.061689000 2.018342000
H 1.746821000 4.464341000 2.880106000
H -1.311272000 5.102459000 2.725791000
H 1.881275000 -4.779077000 2.343593000
H 2.715909000 2.253253000 2.509244000
H -3.732616000 -0.837415000 2.604515000
H 1.053671000 -6.311796000 2.600306000
H -1.888391000 3.465766000 2.381715000
H 0.847825000 5.470857000 4.010840000
H -1.871888000 0.773090000 2.634038000
H -0.522449000 -1.280311000 3.247139000
H 1.802175000 4.115733000 4.612957000
H -2.170995000 -1.335436000 3.883863000
H 2.425143000 -0.540817000 3.874433000
H -0.654511000 3.594653000 4.580499000
H 1.360351000 -0.937685000 5.219272000
H -2.631544000 2.361026000 4.375987000
H -0.829573000 -0.944838000 4.953819000
H -3.315500000 0.762622000 4.673800000
H 2.403426000 1.881621000 4.659001000
H 2.969787000 -0.297627000 5.538860000
H -1.966435000 1.334488000 5.650499000
H 0.179640000 0.940044000 6.543374000
H 0.662448000 2.622372000 6.302700000
H 1.792993000 1.470511000 7.016122000
H -1.405145773 3.197254760 -1.454078727

Table S45. Final coordinates for TS(b)

C -4.906051000 -1.977820000 2.031057000
C -5.921553000 -2.681770000 2.684356000
C -5.798599000 -4.055711000 2.890336000
C -4.660918000 -4.726540000 2.441741000
C -3.645099000 -4.024749000 1.786896000
C -3.766859000 -2.649823000 1.590672000
H -2.895494000 -2.031701000 0.986332000
N -2.128341000 -1.370791000 0.093487000
Si -3.141544000 -1.616387000 -1.390168000
C -2.282729000 -0.733746000 -2.843270000
Np -0.318016000 -0.268908000 0.711889000
N -0.139457000 1.629508000 -0.593658000
Si -1.116079000 3.045880000 -1.167183000
C -2.990431000 2.703675000 -0.822372000
C -3.898804000 3.875104000 -1.214644000
N -0.166866000 0.396432000 2.907674000
C 0.688303000 1.577421000 3.132059000
C 2.094052000 1.302132000 2.632519000
N 2.049407000 0.932930000 1.209895000
C 2.109790000 2.106470000 0.323684000
C 1.290012000 1.831062000 -0.927116000
Si -0.877581000 -0.177523000 4.475341000
C -2.398999000 0.931559000 4.984783000
C -3.641025000 0.625225000 4.146529000
C -1.465710000 -2.013039000 4.301279000
C -2.312952000 -2.469278000 5.495897000
C -0.299135000 -2.985468000 4.095965000
C 0.361096000 -0.213953000 6.004335000
C 0.298788000 0.968882000 6.977228000
C 1.824879000 -0.547243000 5.695442000
N 1.414083000 -1.763387000 0.282614000
Si 1.690468000 -3.231987000 -0.739762000
C 0.565499000 -3.142917000 -2.317963000
C 1.084564000 -2.170873000 -3.383690000
C -4.895399000 -0.857204000 -1.278955000
C -3.381906000 -3.456428000 -1.848898000
C 3.090742000 -0.055601000 0.882555000
C 2.570315000 -1.459384000 1.150023000
C 1.251515000 -4.867191000 0.226889000
C 2.185638000 -5.129356000 1.411779000
C 3.581631000 -3.460821000 -1.223019000
C 3.791718000 -4.723902000 -2.070530000
C -0.208260000 -4.880593000 0.677852000
C -0.975847000 3.493592000 -3.075027000
C -1.656183000 2.535885000 -4.053008000
C -0.480576000 4.686075000 -0.286156000
C -0.837099000 5.994116000 -1.005453000
C 0.425527000 3.838879000 -3.595260000
C -0.889705000 4.793737000 1.185988000
C -3.284482000 2.258738000 0.611276000
C -2.106187000 2.434761000 4.931872000
C 4.285061000 -2.274067000 -1.886328000
C 0.270305000 -4.505233000 -2.958780000
H 0.360362000 4.340605000 -4.570689000
H 3.370775000 -4.610776000 -3.075988000
H 4.863851000 -4.928380000 -2.197711000
H 1.990243000 -2.556044000 -3.865075000
H 1.172582000 -4.962255000 -3.379798000
H 3.870159000 -2.050706000 -2.873623000
H 0.985159000 4.504534000 -2.928829000
H 0.343930000 -2.028829000 -4.182238000
H 5.353125000 -2.489889000 -2.029590000
H -0.444241000 -4.395745000 -3.785687000

H 1.030073000 2.937320000 -3.746991000
H 3.343531000 -5.617455000 -1.624082000
H -1.668150000 2.964879000 -5.065003000
H 1.323601000 -1.182052000 -2.976990000
H -0.157717000 -5.223613000 -2.253164000
H 4.218963000 -1.359050000 -1.293343000
H -1.557334000 4.428220000 -3.095509000
H -0.437099000 6.047070000 -2.021921000
H -0.428490000 6.853687000 -0.455673000
H -1.126788000 1.579793000 -4.117706000
H 4.093609000 -3.641159000 -0.266548000
H 0.615935000 4.596729000 -0.317328000
H 1.703966000 0.946265000 -1.436933000
H -0.386698000 -2.749639000 -1.936011000
H 1.454043000 2.659374000 -1.627270000
H 1.390581000 -5.683539000 -0.496707000
H -2.432083000 -3.965966000 -2.032749000
H -2.696449000 2.326304000 -3.783689000
H -3.919740000 -4.001125000 -1.068112000
H -1.920474000 6.146609000 -1.069372000
H 3.316958000 0.031312000 -0.182719000
H -3.976972000 -3.517918000 -2.768546000
H 3.156819000 2.362706000 0.090232000
H 3.234555000 -5.218348000 1.108422000
H -0.321802000 5.582555000 1.698566000
H -3.745579000 4.200659000 -2.248940000
H -0.902700000 -4.763346000 -0.160406000
H 3.407146000 -2.158606000 1.016248000
H -0.729291000 3.862802000 1.737272000
H 1.919014000 -6.063882000 1.924842000
H 4.016898000 0.161575000 1.440648000
H -0.458898000 -5.820384000 1.190054000
H -3.740830000 4.745089000 -0.567266000
H 1.667341000 2.958256000 0.847547000
H -1.949972000 5.053179000 1.286295000
H -3.241277000 1.861514000 -1.482043000
H -2.740876000 -1.056831000 -3.785668000
H 2.123867000 -4.328755000 2.158446000
H -0.407579000 -4.061341000 1.375523000
H -4.956225000 3.594099000 -1.118330000
H 2.305749000 -1.539528000 2.217832000
H -2.419420000 0.343600000 -2.756829000
H -5.572237000 -1.474355000 -0.682252000
H -5.311404000 -0.785707000 -2.291652000
H -3.074718000 3.048828000 1.338064000
H 2.769668000 2.158780000 2.791889000
H -2.698487000 1.377844000 0.899436000
H -1.212179000 -0.932785000 -2.903463000
H 2.496302000 0.453398000 3.189038000
H 0.342198000 -2.703884000 3.253458000
H 0.289510000 2.462385000 2.617376000
H -4.341831000 1.986328000 0.729857000
H -4.881372000 0.150599000 -0.854016000
H -0.666364000 -4.001765000 3.904028000
H -2.106980000 -2.058158000 3.411244000
H -3.461092000 0.843498000 3.088131000
H 0.755916000 1.866729000 4.191676000
H 0.336026000 -3.042932000 4.987882000
H 1.940969000 -1.343870000 4.954668000
H -3.954977000 -0.419397000 4.216504000
H 2.362586000 0.332667000 5.326885000
H -1.893486000 2.753832000 3.905565000
H -2.673473000 -3.494378000 5.342050000
H -4.491479000 1.245860000 4.462170000
H -3.192957000 -1.840333000 5.660478000
H 2.346710000 -0.871534000 6.606491000
H -1.732928000 -2.472342000 6.427570000
H -2.975996000 3.012257000 5.274175000
H -0.050522000 -1.077907000 6.549244000
H -1.255937000 2.732294000 5.552481000
H -2.615856000 0.659140000 6.029054000
H 0.717570000 1.884431000 6.542132000
H -0.721014000 1.192102000 7.305241000
H 0.887321000 0.752556000 7.879916000
H -2.766225000 -4.553100000 1.431624000
H -4.564186000 -5.798679000 2.595869000
H -6.589828000 -4.603277000 3.395968000
H -6.809209000 -2.156124000 3.028195000
H -5.009251000 -0.909585000 1.865912000

Table S46. Final coordinates for TS(t)

C 0.535965000 4.145065000 -3.786673000
C -0.849510000 3.785684000 -3.233710000
Si -0.948092000 3.326179000 -1.325158000
N 0.020968000 1.893297000 -0.786792000
C 1.439122000 2.043071000 -1.184283000
C 2.319956000 2.322428000 0.024038000
N 2.269129000 1.168972000 0.938358000
C 2.343133000 1.570705000 2.351579000
C 0.945211000 1.870861000 2.862752000
N 0.091552000 0.671904000 2.747184000
Si -0.546607000 0.184838000 4.368093000
C -2.185442000 1.165951000 4.778329000
C -3.406137000 0.611499000 4.039859000
C -0.297859000 4.965113000 0.450486000
C -0.697511000 5.088860000 1.023241000
C -2.815198000 2.976674000 0.949446000
C -3.737409000 4.141471000 1.328331000
C -1.549673000 2.833646000 4.203960000
C -0.655915000 6.271145000 1.173336000
C -3.080561000 2.537105000 0.491604000
Np -0.135743000 -0.006255000 0.537595000
N 1.563233000 -1.529715000 0.109906000
Si 1.750241000 -3.037253000 -0.870621000

C 0.575396000 -2.949963000 -2.413679000
C 1.118052000 -2.051434000 -3.529988000
N -1.935350000 -1.144128000 -0.057999000
Si -3.005282000 -1.253595000 -1.511405000
C -2.158116000 -0.332935000 -2.950766000
C -4.724725000 -0.462138000 -1.232388000
C -3.310653000 -3.056886000 -2.093489000
C 3.287846000 0.159851000 0.606019000
C 2.758430000 -1.230032000 0.923179000
C 1.282592000 -4.624422000 0.164247000
C 2.265334000 -4.910087000 1.303754000
C 3.612474000 -3.352106000 -1.411057000
C 3.732620000 -4.626618000 -2.258935000
C -0.147444000 -4.539799000 0.696953000
C 0.676743000 0.456429000 5.883812000
C 0.534746000 1.766988000 6.666096000
C -0.950892000 -1.709674000 4.396479000
C 0.272499000 -2.600988000 4.161222000
C 2.159103000 0.164328000 5.625281000
C -1.669854000 -2.140356000 5.681255000
C -2.050449000 2.665349000 4.496380000
C 4.351550000 -2.201231000 -2.099360000
C 0.187579000 -4.320812000 -2.981561000
C -3.268025000 -2.585370000 1.727350000
C -4.458437000 -3.359858000 1.345579000
C -5.746400000 -2.793662000 1.412622000
C -6.876837000 -3.534222000 1.081154000
C -6.751795000 -4.864329000 0.676224000
C -5.484711000 -5.447200000 0.614115000
C -4.355056000 -4.706076000 0.944722000
H 0.442250000 4.671476000 -4.746738000
H 3.283665000 -4.496569000 -3.250146000
H 4.788374000 -4.884561000 -2.420586000
H 2.005011000 -2.489606000 -4.001314000
H 1.053069000 -4.862033000 -3.378945000
H 3.900717000 -1.937987000 -3.060772000
H 1.117438000 4.792053000 -3.120603000
H 0.377003000 -1.920184000 -4.330274000
H 5.395316000 -2.480715000 -2.301051000
H -0.521508000 -4.209143000 -3.812937000
H 1.134210000 3.247329000 -3.980556000
H 3.255817000 -5.494712000 -1.791867000
H -1.614405000 3.285138000 -5.204106000
H 1.392126000 -1.053417000 -3.171246000
H -0.288096000 -4.965310000 -2.236534000
H 4.376235000 -1.292262000 -1.493176000
H -1.437711000 4.716354000 -3.230635000
H -0.264783000 6.319335000 -2.193203000
H -0.240736000 7.131836000 -0.630239000
H -1.001214000 1.892572000 -4.313255000
H 4.144812000 -3.554458000 -0.470498000
H 0.798230000 4.871309000 -0.487486000
H 1.806143000 1.132345000 -1.685503000
H -0.340558000 -2.487992000 -2.019031000
H 1.597312000 2.846555000 -1.914211000
H 1.333531000 -5.465072000 -0.543065000
H -3.075031000 -3.791001000 -1.320224000
H -2.570179000 2.588692000 -3.893593000
H -4.363475000 -3.196997000 -2.362133000
H -1.739626000 6.425879000 -1.228147000
H 3.482415000 0.216514000 -0.467986000
H -2.704728000 -3.287456000 -2.975123000
H 3.362004000 2.546570000 -0.259143000
H 3.289015000 -5.067864000 0.947029000
H -0.130554000 5.888843000 1.519466000
H -3.609014000 4.460656000 -2.368025000
H -0.878200000 -4.373880000 -0.100959000
H 3.576929000 -1.946388000 0.766221000
H -0.527327000 4.169069000 1.589305000
H 1.975736000 -5.814170000 1.857673000
H 4.233186000 0.377799000 1.130860000
H -0.430248000 -5.458054000 1.231142000
H -3.568699000 5.016824000 -0.690721000
H 1.922971000 3.197156000 0.546564000
H -1.758421000 5.344120000 1.127119000
H -3.069111000 2.130993000 -1.604310000
H -1.427348000 -0.971710000 -3.452724000
H 2.290081000 -4.088124000 2.029037000
H -0.246801000 -3.706319000 1.399871000
H -4.791285000 3.856877000 -1.206306000
H 2.539158000 -1.285752000 2.002384000
H -2.633918000 -1.919982000 0.797893000
H -2.909720000 -0.039849000 -3.692060000
H -5.256799000 -0.405882000 -2.190082000
H -4.658155000 0.549631000 -0.823454000
H -2.862829000 3.334029000 1.208120000
H 3.027473000 2.426322000 2.476876000
H -2.483799000 1.661706000 0.776199000
H -1.639109000 0.565193000 -2.610769000
H 2.748329000 0.733373000 2.923880000
H 0.813718000 -2.332889000 3.248000000
H 0.534747000 2.710630000 2.285059000
H -4.132950000 2.257429000 0.633679000
H -5.334707000 -1.064735000 -0.554055000
H -0.019858000 -3.655946000 4.074605000
H -1.645327000 -1.873098000 3.565053000
H -3.270681000 0.659811000 2.952675000
H 1.028954000 2.246092000 3.892842000
H 0.981336000 -2.539664000 4.995360000
H 2.322062000 -0.732858000 5.021181000
H -3.622408000 -0.428615000 4.304020000
H 2.648681000 1.001280000 5.116397000
H -1.868463000 2.842525000 3.430998000
H -1.983792000 -3.190737000 5.616947000

H	-4.305925000	1.197374000	4.273472000
H	-2.565572000	-1.547443000	5.892555000
H	2.694597000	0.020490000	6.573876000
H	-1.014009000	-2.061445000	6.556997000
H	-2.972985000	3.200048000	4.761637000
H	0.311508000	-0.337337000	6.553939000
H	-1.232467000	3.133269000	5.051971000
H	-2.350438000	1.029769000	5.857800000
H	0.891861000	2.632612000	6.095115000
H	-0.498438000	1.970048000	6.963806000
H	1.132728000	1.729605000	7.587690000
H	-3.373751000	-5.172540000	0.905818000
H	-5.378208000	-6.485877000	0.311791000
H	-7.634461000	-5.443873000	0.419743000
H	-7.859899000	-3.074909000	1.145595000
H	-5.854002000	-1.763609000	1.744353000
H	-3.491400000	-1.765413000	2.411101000
H	-2.449772000	-3.208550000	2.091317000

Table S47. Final coordinates for TS(h)

C	1.262282000	1.423024000	-4.080722000
Si	1.344886000	-0.472001000	-3.874193000
C	-0.387605000	-1.199753000	-4.249479000
C	2.505092000	-1.059943000	-5.283075000
N	1.935281000	-0.855136000	-2.211232000
Np	1.970027000	-0.142278000	-0.106037000
N	0.846163000	1.868673000	0.132871000
Si	-0.740514000	2.623305000	-0.304023000
C	-1.682320000	1.428824000	-1.501380000
C	-3.064443000	1.951171000	-1.912170000
N	4.210050000	0.273780000	0.162978000
C	4.600498000	0.085439000	1.575353000
C	3.867256000	1.063095000	2.480002000
N	2.412878000	0.856997000	2.358915000
C	1.669344000	2.125265000	2.445631000
C	1.578916000	2.754569000	1.065763000
N	1.166460000	-1.595644000	1.505613000
Si	0.857097000	-3.371921000	1.651748000
C	1.152538000	-4.134979000	3.442151000
C	-0.086229000	-4.304704000	4.329298000
Si	5.617152000	0.706012000	-0.883130000
C	6.875235000	-0.780134000	-1.061227000
C	7.679348000	-1.113330000	0.200463000
C	6.668408000	2.143984000	-0.063278000
C	7.945439000	2.449947000	-0.858144000
C	4.980806000	1.184304000	-2.652676000
C	4.490649000	2.634322000	-2.736583000
C	5.927665000	3.448143000	0.253056000
C	1.922398000	-0.135065000	3.328902000
C	0.746688000	-0.890691000	2.732613000
C	2.058175000	-4.355101000	0.496328000
C	3.532547000	-4.174507000	0.869399000
C	-0.968770000	-3.798859000	1.115834000
C	-1.142232000	-3.780937000	-0.405567000
C	1.715665000	-5.848900000	0.429304000
C	-1.766030000	3.008919000	1.331647000
C	-2.853891000	4.078363000	1.164325000
C	-0.636774000	4.368403000	-1.192758000
C	0.078954000	5.476459000	-0.410129000
C	-2.374947000	1.771215000	1.998559000
C	-0.125045000	4.352345000	-2.633595000
C	-1.791167000	-0.001051000	-0.969285000
C	-2.004292000	-2.878171000	1.769624000
C	2.300507000	-3.535483000	4.262470000
C	6.001852000	0.918082000	-3.766322000
C	6.189200000	-2.038465000	-1.592509000
C	2.975328000	-3.165256000	-2.793858000
C	1.972470000	-4.152663000	-3.354392000
C	2.633991000	-5.416119000	-3.925152000
C	1.624049000	-6.438796000	-4.449523000
C	2.271068000	-7.691982000	-5.041245000
C	1.254037000	-8.710192000	-5.550445000
H	-0.108651000	6.456141000	-0.871082000
H	7.715629000	2.908512000	-1.827302000
H	8.582828000	3.161311000	-0.315004000
H	5.321367000	3.342243000	-2.641354000
H	6.916322000	1.508213000	-3.634262000
H	5.743733000	4.040361000	-0.648281000
H	-0.243172000	5.546379000	0.634917000
H	4.020438000	2.834372000	-3.707722000
H	6.520324000	4.077335000	0.931470000
H	5.586131000	1.195432000	-4.744658000
H	1.164818000	5.329975000	-0.414519000
H	8.549929000	1.557942000	-1.051579000
H	-0.254742000	5.339259000	-3.099848000
H	3.756386000	2.877866000	-1.961791000
H	6.298386000	-0.133299000	-3.827228000
H	4.956446000	3.279820000	0.727353000
H	-1.700912000	4.647081000	-1.238957000
H	-2.458722000	5.043096000	0.833580000
H	-3.369375000	4.252732000	2.119173000
H	0.943558000	4.117279000	-2.681744000
H	6.987587000	1.719064000	0.900140000
H	-1.019621000	3.420335000	2.027914000
H	2.594250000	2.964159000	0.696950000
H	4.116799000	0.524227000	-2.816279000
H	1.112292000	3.741544000	1.183864000
H	7.590364000	-0.439206000	-1.824445000
H	3.545268000	-0.774341000	-5.099052000
H	-0.653408000	3.627641000	-3.260262000
H	2.468715000	-2.136591000	-5.467312000
H	-3.619236000	3.772244000	0.442469000
H	4.101923000	2.078857000	2.153956000
H	2.184280000	-0.558696000	-6.204839000

H	2.136756000	2.798459000	3.184009000
H	8.204397000	-0.246038000	0.615270000
H	-2.737915000	2.007185000	3.008430000
H	-3.024920000	2.960971000	-2.334252000
H	5.656590000	-1.857182000	-2.530824000
H	5.678318000	0.217283000	1.743930000
H	-1.663046000	0.945753000	2.091679000
H	8.440262000	-1.876024000	-0.016215000
H	4.183010000	0.971867000	3.532186000
H	6.912968000	-2.845363000	-1.773817000
H	-3.755734000	1.974829000	-1.061854000
H	0.654401000	1.908119000	2.790445000
H	-3.236576000	1.398621000	1.433139000
H	-1.060403000	1.399533000	-2.406974000
H	2.202352000	1.796632000	-4.496632000
H	7.039944000	-1.523698000	0.990533000
H	5.454710000	-2.413711000	-0.872049000
H	-3.516930000	1.299962000	-2.672071000
H	4.389428000	-0.941984000	1.917282000
H	2.444806000	-2.088623000	-2.414682000
H	0.459558000	1.692025000	-4.776536000
H	-0.562372000	-1.201211000	-5.332297000
H	-1.179993000	-0.604223000	-3.787161000
H	-2.437583000	-0.056174000	-0.089226000
H	1.666371000	0.351147000	4.285573000
H	-0.819507000	-0.419662000	-0.676995000
H	1.086668000	1.937430000	-3.135322000
H	2.726537000	-0.849093000	3.522370000
H	3.827203000	-3.120351000	0.911918000
H	-0.067142000	-0.180135000	2.527107000
H	-2.213945000	-0.677327000	-1.722421000
H	-0.490787000	-2.227580000	-3.890262000
H	4.189818000	-4.673469000	0.144532000
H	1.906301000	-3.935198000	-0.505745000
H	-0.873406000	-2.806914000	-0.828924000
H	0.347546000	-1.564995000	3.503961000
H	3.753168000	-4.617048000	1.847791000
H	3.198541000	-3.343210000	3.668269000
H	-0.529808000	-4.535843000	-0.908081000
H	2.005526000	-2.592665000	4.734805000
H	-1.851240000	-1.838715000	1.460859000
H	2.360730000	-6.364371000	-0.294308000
H	-2.187208000	-3.975635000	-0.683677000
H	0.679245000	-6.037990000	0.132109000
H	2.587250000	-4.215375000	5.076488000
H	1.871489000	-6.345661000	1.394973000
H	-3.023150000	-3.158329000	1.468982000
H	1.467593000	-5.151996000	3.160907000
H	-1.969517000	-2.906609000	2.862721000
H	-1.150120000	-4.825669000	1.468012000
H	-0.474402000	-3.343146000	4.686025000
H	-0.902475000	-4.822898000	3.816752000
H	0.160137000	-4.895701000	5.222520000
H	3.708540000	-2.835292000	-3.533966000
H	3.501042000	-3.534980000	-1.909841000
H	1.254580000	-4.442894000	-2.576650000
H	1.382334000	-3.679828000	-4.149272000
H	3.318798000	-5.128661000	-4.735404000
H	3.260260000	-5.883564000	-3.152699000
H	0.947978000	-6.731962000	-3.633626000
H	0.988966000	-5.964541000	-5.211603000
H	2.940476000	-7.400673000	-5.862217000
H	2.910846000	-8.162385000	-4.281975000
H	1.745592000	-9.595862000	-5.967594000
H	0.592001000	-9.049011000	-4.744828000
H	0.621178000	-8.282759000	-6.337006000

Table S48. Final coordinates for 3UNSiMe3

U	0.160719	0.015483	-0.003194
Si	0.179612	-3.701918	-0.516367
Si	0.072100	2.320047	-2.985404
Si	0.228845	1.361513	3.498228
Si	-3.552492	0.030698	0.074092
N	0.934569	-2.135931	-0.012817
N	2.827421	0.040362	-0.067253
N	0.864872	1.108700	-1.903906
N	0.953843	1.045326	1.871723
N	-1.764121	0.042544	0.036262
C	2.617033	0.354407	4.958680
C	-0.393535	-1.362824	4.285973
C	-2.130805	0.349931	4.883525
C	-1.054246	-0.045434	3.866126
C	-1.792796	3.125531	2.450511
C	0.220570	4.282087	3.421666
C	-0.707082	3.067939	3.526900
C	-1.896677	-3.611226	1.486843
C	2.557777	-4.577053	-2.076354
C	0.883160	-6.345996	-1.460238
C	1.528426	-5.020388	-1.032717
C	-0.398306	-3.020057	-3.278685
C	-2.171714	-4.370471	-2.121302
C	-1.079584	-3.309207	-1.936027
C	0.135215	-4.964185	2.102805
C	-0.784943	-4.520320	0.961211
C	2.260918	-2.253187	0.618663
C	3.265970	-1.369158	-0.108941
C	-2.288117	4.016400	-2.698246
C	0.732603	4.429369	-4.860554
C	2.413781	4.145549	-3.011279
C	1.395906	3.429461	-3.903940
C	-0.050573	0.717873	-5.411069
C	-2.007191	0.514287	-3.834512
C	-0.940585	1.451560	-4.402254
C	-0.466390	4.395370	-1.013228

C -1.158115 3.353952 -1.900269
C 2.237774 1.724874 1.623627
C 3.282704 0.719191 1.163421
C 3.222561 0.780258 -1.282989
C 2.170481 0.608529 -2.370904
C 0.989902 1.757668 6.264692
C 1.605512 1.501294 4.882303
C -4.284695 1.743380 -0.361745
C -4.238242 -0.438682 1.796850
C -4.287178 -1.223899 -1.169684
H 1.770304 2.000808 6.998760
H 0.277611 2.589360 6.264207
H 0.464065 0.873333 6.642316
H -1.704545 0.538708 5.875314
H 3.422734 0.593924 5.666616
H 2.169530 2.408126 4.617832
H -2.865698 -0.457626 5.000190
H 0.054874 -1.282318 5.282464
H -2.681546 1.248935 4.590839
H 2.155970 -0.576024 5.303610
H -1.200826 3.110456 4.508851
H 3.087220 0.144070 3.994185
H -1.130606 -2.174537 4.334025
H 0.955451 4.321561 4.232843
H 0.394574 -1.674038 3.592184
H -1.556787 -0.212851 2.901461
H -0.353903 5.217890 3.463021
H -2.515492 2.308583 2.542186
H -0.441737 -5.432358 2.912174
H -3.985520 0.306984 2.555681
H 2.631841 2.235399 2.513140
H 0.669011 -4.113837 2.543393
H -2.353011 4.069521 2.501178
H 0.772168 4.290124 2.474340
H 3.398778 -0.038601 1.942338
H -2.460978 -4.099025 2.293869
H -3.868935 -1.411159 2.134980
H 2.229579 -1.965142 1.681962
H -1.486715 -2.679370 1.889673
H 0.884745 -5.694357 1.778845
H -1.355924 3.055428 1.449167
H -5.332385 -0.496890 1.742973
H 4.267435 1.186120 1.001235
H 2.141295 2.515366 0.860540
H 2.651174 -3.280447 0.611807
H -2.612685 -3.336756 0.705794
H 4.283261 -1.465936 0.303975
H -3.902842 2.530922 0.294756
H -1.255206 -5.420669 0.539444
H -1.170839 4.822093 -0.287573
H 2.080836 -5.225976 -0.103763
H -5.374767 1.710867 -0.243395
H 0.374139 3.976574 -0.449518
H 3.300567 -1.678323 -1.156957
H 0.165864 -6.726179 -0.725225
H -4.038529 -2.255100 -0.902549
H 3.279458 1.841085 -1.024749
H -1.618092 2.603879 -1.238008
H -4.073708 2.026858 -1.396675
H -5.380824 -1.136784 -1.165762
H -0.080785 5.232445 -1.606141
H -2.732751 -4.568968 -1.202918
H 4.223231 0.460745 -1.616828
H -1.574127 -2.383544 -1.601680
H 3.082682 -3.662684 -1.786644
H 1.648522 -7.122211 -1.596893
H -2.999532 4.509943 -2.022848
H -3.941973 -1.041277 -2.191329
H 3.320288 -5.354874 -2.221867
H 2.121230 -0.455999 -2.654883
H 0.356380 -6.248544 -2.416207
H 2.906479 3.469102 -2.307112
H 1.947121 4.940855 -2.422511
H -1.756718 -5.326139 -2.462171
H 0.406918 -2.282651 -3.190338
H 2.099539 -4.392611 -3.052850
H -2.896074 -4.048220 -2.880966
H -2.857035 3.302813 -3.301804
H -1.551252 -0.295214 -3.254852
H -2.700810 1.034593 -3.166599
H 2.533497 1.126053 -3.269413
H -1.908662 4.787005 -3.379336
H 0.033730 -3.930615 -3.709228
H 3.200717 4.614786 -3.617814
H -1.120090 -2.636362 -4.010912
H 0.168921 5.197782 -4.319419
H -2.602191 0.053447 -4.635306
H 1.957692 2.721802 -4.531790
H 0.516978 -0.090425 -4.934769
H -1.453417 2.265473 -4.936574
H 0.043321 3.947492 -5.561576
H 1.489685 4.953775 -5.459826
H 0.669584 1.384367 -5.898566
H -0.652795 0.258077 -6.206814

Table S49. Final coordinates for TS(bU5)

U -0.384415 -0.323657 0.808776
Si -1.076437 -0.206253 4.588972
Si 1.620810 -3.098814 -0.847989
Si -0.901425 2.918826 -1.219357
Si -3.384766 -1.588604 -1.068157
N 1.330281 -1.768735 0.342169
N 0.017797 1.505785 -0.542753
N 2.037752 0.788439 1.425168

N -0.305627 0.306682 3.032997
N -2.291265 -1.374412 0.359810
C -0.081111 -4.931734 0.580972
C 0.065162 -4.205796 -3.053618
C 4.037688 -1.896592 -2.142657
C 0.778083 -1.810284 -3.313759
C 0.366982 -2.896614 -2.313980
C 3.743613 -4.375890 -2.357664
C 3.482104 -3.153983 -1.464920
C -0.485147 -3.028544 4.271259
C -2.352676 2.408685 4.824872
C 0.725744 3.412162 -3.652311
C -3.156776 2.475072 0.593821
C 1.562118 -0.511853 5.958433
C -3.837328 0.526614 4.111666
C -2.631265 0.908948 4.971840
C -0.015627 1.086756 7.069504
C 0.088356 -0.154243 6.176770
C -2.503969 -2.481377 5.655333
C -1.646860 -2.048189 4.459510
C -2.582476 -0.793779 -2.611247
C -3.718387 -3.417840 -1.518557
C -0.615323 4.823408 1.017287
C -3.666115 3.763536 -1.496238
C -2.792578 2.662378 -0.881438
C -0.516124 5.847756 -1.260088
C -0.207491 4.587573 -0.440149
C 3.062518 -0.238837 1.171501
C 2.433606 -1.615324 1.313455
C 2.377534 -5.242220 1.004054
C 1.318203 -4.839924 -0.026852
C -1.420061 2.187247 -4.048524
C -0.707766 3.199897 -3.150652
C 1.469572 1.701003 -0.769341
C 2.192541 1.955682 0.542950
C 1.992331 1.165699 2.845456
C 0.561856 1.484103 3.244796
C -5.101505 -0.773823 -0.835959
C -4.433997 -5.145964 2.592289
C -5.636569 -4.638237 3.084873
C -5.919339 -3.277424 2.969687
C -4.996837 -2.416488 2.362362
C -3.801426 -2.939626 1.888386
C -3.506124 -4.292843 1.982680
H -5.221120 -1.357620 2.271150
H -6.858551 -2.880304 3.348455
H -6.355373 -5.304498 3.554670
H -4.214734 -6.208217 2.675408
H -2.577479 -4.693575 1.588189
H 0.522450 0.925878 8.014359
H -1.049957 1.333815 7.327379
H 0.432380 1.971535 6.601458
H -2.885104 0.706654 6.023654
H -1.524417 2.758314 5.448181
H -0.353613 -0.982291 6.753128
H -3.238092 2.998317 5.100229
H -1.922851 -2.501388 6.585989
H 2.038298 -0.788028 6.909381
H -3.364769 -1.826483 5.822811
H -4.700013 1.169883 4.335470
H -2.894867 -3.495293 5.502311
H -2.112711 2.658308 3.785447
H 2.124406 0.342009 5.566195
H -4.151309 -0.508258 4.268682
H 1.703942 -1.349630 5.269494
H 0.156689 -3.066800 5.159648
H 0.567972 1.830307 4.288751
H -3.616343 0.644626 3.044380
H -2.283217 -2.106739 3.566764
H -0.858455 -4.047801 4.106538
H -5.024295 0.280811 -0.554139
H -4.206068 2.173136 0.704044
H 0.214263 2.349938 2.662362
H 0.152476 -2.769722 3.418245
H 2.328534 0.309474 3.433738
H -1.732689 -0.150981 -2.372354
H -2.553516 1.705505 1.088356
H 2.678517 2.004534 3.050727
H -3.028662 3.400213 1.163108
H -5.655145 -0.829453 -1.781419
H -5.696019 -1.288321 -0.076230
H -3.322921 -0.187804 -3.143165
H 2.073537 -1.733591 2.348510
H -4.730763 3.509634 -1.403720
H -0.191531 -4.223519 1.409362
H 2.354768 -4.587672 1.883144
H -2.238628 -1.568367 -3.303250
H -3.031509 1.723528 -1.400512
H -1.667554 5.119095 1.093480
H 1.729517 2.819867 1.028195
H -3.524058 4.722548 -0.984243
H -0.279645 -5.937787 0.976148
H 3.927238 -0.100107 1.840404
H 2.202270 -6.264533 1.366877
H -0.478455 3.938858 1.646097
H 3.220416 -2.374380 1.201302
H -0.865870 -4.700026 -0.146782
H -3.463727 3.924538 -2.560016
H -0.025522 5.636567 1.462671
H 3.394373 -5.215812 0.597390
H 3.259577 2.190751 0.391098
H -4.266849 -3.451329 -2.468381
H 3.418194 -0.124777 0.145857
H -1.593945 6.029289 -1.342083

H -4.321315 -3.930514 -0.765033
H -2.472593 2.054426 -3.780278
H -2.788812 -3.979525 -1.653633
H 1.373657 -5.558212 -0.857739
H 1.692427 2.542193 -1.439121
H -0.565621 -2.573372 -1.828935
H 1.911576 0.820839 -1.259711
H 0.884933 4.456783 -0.455790
H 4.065884 -3.305936 -0.545073
H -0.943084 1.202807 -4.010803
H -0.081209 6.733983 -0.777033
H -0.111814 5.807660 -2.275470
H -1.233779 4.158381 -3.276992
H 3.832314 -0.981444 -1.580967
H -0.319043 -4.986929 -2.390840
H 1.013124 -0.857520 -2.827606
H -1.391412 2.516857 -5.096746
H 3.429547 -5.317752 -1.896736
H 1.291388 2.473699 -3.656145
H -0.688304 -4.044231 -3.836376
H 5.128102 -1.969474 -2.256582
H -0.023104 -1.619308 -4.038893
H 1.288627 4.137410 -3.053787
H 3.623251 -1.756473 -3.145681
H 0.955473 -4.607932 -3.550504
H 1.657896 -2.114352 -3.890879
H 4.815037 -4.468170 -2.582935
H 3.224895 -4.290426 -3.319452
H 0.720294 3.782431 -4.686908
H -2.838126 -2.026720 1.168818

Table S50. Final coordinates for 4aU

U 0.135112 -0.006023 0.026718
Si -0.653791 3.355776 -1.441830
Si 0.814291 -2.881346 -2.364061
Si 0.303738 -0.344189 3.781432
Si -3.673734 -1.052908 0.141851
N 0.460941 1.982249 -1.099257
N 1.260373 -1.636569 -1.132891
N 2.740340 0.613066 -0.011580
N 0.892096 0.253139 2.186273
N -1.883821 -1.003661 0.071799
C -2.172782 1.120850 3.883205
C -0.191417 2.366345 4.762976
C -0.842155 0.985303 4.629158
C 0.132443 -3.164922 3.146947
C -1.556501 -2.317464 4.788110
C -0.737193 -1.965047 3.539232
C -4.256680 -2.342999 1.431710
C -4.423133 0.631550 0.623749
C -0.653663 6.212005 -1.958779
C 1.316200 5.420781 -0.613045
C 0.304999 5.041894 -1.699211
C -0.757661 3.070843 -4.332431
C -2.507480 1.812950 -3.031902
C -1.654143 3.081290 -3.090209
C -0.439431 -1.178193 -4.357963
C -1.596123 -3.367344 -3.950208
C -0.758095 -2.252330 -3.311115
C 1.953292 -4.329269 -4.606337
C 2.941298 -2.042687 -4.255012
C 2.311197 -3.257256 -3.568403
C -3.237076 4.146584 -0.308736
C -1.312765 4.016620 1.304707
C -1.921472 3.433132 0.025660
C 1.784166 2.077622 -1.740265
C 2.882471 1.911032 -0.698328
C 1.601338 -5.300881 -0.919530
C -0.761928 -4.532077 -0.556061
C 0.391694 -4.608722 -1.557216
C 2.632656 -1.775837 -0.602501
C 3.410912 -0.474711 -0.749860
C 3.184971 0.678822 1.393915
C 2.049416 1.163072 2.286373
C 2.996872 -1.420449 4.407617
C 2.080638 0.151029 6.135567
C 1.719156 -0.860871 5.043381
C -4.444605 -1.558257 -1.535995
H 2.752533 -0.307423 6.874994
H 1.203085 0.512583 6.680754
H 2.606045 1.025107 5.733122
H -1.049240 0.600079 5.639057
H 0.732994 2.350757 5.346164
H 1.228805 -1.702720 5.557919
H -0.873936 3.073506 5.254892
H -0.912147 -2.501129 5.657675
H 3.607921 -1.937004 5.160817
H -2.266995 -1.534629 5.071120
H -2.831277 1.850432 4.374541
H -2.134424 -3.237222 4.625892
H 0.045999 2.785102 3.779131
H 3.621611 -0.621871 3.992997
H -2.723030 0.177394 3.815995
H 2.796642 -2.135715 3.604659
H 0.811953 -3.449588 3.959112
H 2.442680 1.244434 3.310748
H -2.007162 1.475938 2.859383
H -1.435539 -1.769353 2.713683
H -0.488267 -4.044809 2.931464
H -3.997459 -2.042890 2.452135
H -1.992343 3.894762 2.157630
H 1.781518 2.193523 2.001015
H 0.747042 -2.969782 2.261615
H 3.458124 -0.331815 1.709017

H -4.008014 1.020694 1.557596
H -0.363648 3.536641 1.565081
H 4.082618 1.313242 1.485448
H -1.123966 5.090708 1.200670
H -5.345850 -2.461635 1.385809
H -3.810222 -3.327007 1.250166
H -5.501706 0.500733 0.773904
H -1.575978 -1.982946 0.008239
H 2.628637 -2.066272 0.461760
H -3.942967 4.074842 0.529407
H -0.490392 -3.919802 0.311457
H 1.990002 -4.733168 -0.065851
H -4.294644 1.385778 -0.157371
H -2.167399 2.378323 0.231291
H 0.826599 5.668215 0.334186
H 2.768992 2.699759 0.050315
H -3.078303 5.213462 -0.501773
H -1.025413 -5.527444 -0.172502
H 4.455716 -0.581834 -0.414865
H 1.328413 -6.295616 -0.540859
H 2.031234 4.619785 -0.407495
H 3.208424 -2.564929 -1.105801
H -1.670573 -4.107013 -0.996345
H -3.736075 3.730365 -1.189242
H 1.895998 6.304506 -0.913931
H 2.426699 -5.442576 -1.625637
H 3.889575 2.008484 -1.137219
H -5.539244 -1.546735 -1.466909
H 3.429978 -0.202891 -1.808116
H -1.224247 6.474650 -1.060507
H -4.145969 -2.568708 -1.833771
H -3.226135 1.831323 -2.205779
H -4.153660 -0.870582 -2.336392
H 0.060037 -5.232090 -2.400848
H 1.953620 3.036475 -2.251023
H -1.376118 -1.783925 -2.530144
H 1.911998 1.310145 -2.520650
H 0.873299 4.890452 -2.628954
H 3.084279 -3.694779 -2.919927
H -1.883372 0.921967 -2.902223
H -0.095833 7.111286 -2.254034
H -1.372193 6.003011 -2.758209
H -2.333418 3.942650 -3.172395
H 3.186525 -1.245663 -3.547938
H -1.930255 -4.117618 -3.227341
H 0.185042 -0.372812 -3.957573
H -3.082536 1.674970 -3.957857
H 1.546920 -5.238914 -4.152040
H -0.032990 2.248833 -4.297687
H -2.495628 -2.948332 -4.420905
H 3.871196 -2.323866 -4.768279
H -1.360519 -0.721958 -4.742835
H -0.193834 4.002571 -4.451231
H 2.276854 -1.611704 -5.009848
H -1.044888 -3.896004 -4.736460
H 0.085028 -1.604750 -5.220469
H 2.840914 -4.624823 -5.182755
H 1.214439 -3.962977 -5.328478
H -1.351387 2.934975 -5.246894

Table S51. Final coordinates for 8UNSiMe3

U 0.082861 0.001677 -0.020884
Si 0.163886 -3.775787 -0.484392
Si 0.104864 2.367802 -3.055955
Si 0.304478 1.414874 3.528602
Si -3.658829 0.014719 0.082377
N 0.926930 -2.248709 -0.068465
N 2.818702 0.010855 -0.098761
N 0.855581 1.215206 -1.963604
N 0.977048 1.049443 1.950286
N -1.937605 0.047630 0.002069
C 2.656768 0.315569 4.964267
C -0.455891 -1.277749 4.255069
C -2.064605 0.499421 4.997363
C -1.045155 0.098219 3.924131
C -1.702935 3.270541 2.620024
C 0.441090 4.317741 3.404013
C -0.542448 3.160057 3.613104
C -1.873505 -3.706757 1.551704
C 2.441608 -4.685899 -2.141703
C 0.846244 -6.469251 -1.393212
C 1.470928 -5.113543 -1.034946
C -0.560207 -3.101095 -3.210047
C -2.234237 -4.523902 -1.998672
C -1.163061 -3.436595 -1.840427
C 0.216044 -4.962362 2.160205
C -0.743614 -4.594652 1.022518
C 2.272520 -2.325618 0.507229
C 3.239688 -1.393809 -0.220078
C -2.419040 3.862952 -2.981877
C 0.791142 4.671994 -4.717480
C 2.295590 4.331552 -2.734539
C 1.407813 3.633913 -3.770626
C 0.383064 0.910706 -5.564355
C -1.729457 0.502727 -4.267615
C -0.664434 1.538868 -4.637590
C -0.825245 4.279906 -1.091988
C -1.321700 3.243511 -2.106881
C 2.278058 1.661158 1.658839
C 3.278347 0.622084 1.158687
C 3.204091 0.810556 -1.272562
C 2.164178 0.703925 -2.386247
C 1.101440 1.741487 6.318773
C 1.675148 1.491557 4.917059

C -4.488267 1.682457 -0.359691
C -4.346614 -0.423176 1.814857
C -4.452309 -1.261743 -1.103369
H 1.901675 1.952325 7.043836
H 0.408543 2.589071 6.350623
H 0.561685 0.864264 6.694883
H -1.601982 0.590311 5.987677
H 3.471917 0.507458 5.678433
H 2.266100 2.384770 4.660125
H -2.856694 -0.257146 5.086889
H 0.024783 -1.278101 5.241089
H -2.554943 1.454035 4.782524
H 2.169169 -0.611893 5.283048
H -0.957883 3.256247 4.628713
H 3.117732 0.116602 3.992905
H -1.235694 -2.050736 4.283175
H 1.241260 4.332851 4.153200
H 0.293857 -1.593337 3.522148
H -1.590711 0.005582 2.971777
H -0.069038 5.290900 3.459475
H -2.480924 2.521521 2.799914
H -0.319851 -5.420238 3.004936
H -4.086409 0.341273 2.555648
H 2.737945 2.155238 2.530350
H 0.724774 -4.073586 2.551889
H -2.182507 4.259798 2.667627
H 0.916854 4.265434 2.417513
H 3.349725 -0.168899 1.910988
H -2.380461 -4.168709 2.412320
H -3.954324 -1.381202 2.173875
H 2.280018 -2.062042 1.579954
H -1.493275 -2.733983 1.878514
H 0.989656 -5.673389 1.847484
H -1.358007 3.120601 1.591913
H -5.442162 -0.499442 1.795875
H 4.287930 1.053936 1.031903
H 2.198638 2.457537 0.896832
H 2.714098 -3.334583 0.465448
H -2.636721 -3.507138 0.793214
H 4.277310 -1.519206 0.140664
H -4.062770 2.507764 0.222170
H -1.196004 -5.527478 0.651870
H -1.638740 4.609305 -0.431328
H 2.073921 -5.279694 -0.128340
H -5.566703 1.653892 -0.153154
H -0.025699 3.886841 -0.456164
H 3.224905 -1.656358 -1.282511
H 0.169750 -6.844798 -0.618013
H -4.201420 -2.288979 -0.815089
H 3.260768 1.857813 -0.961063
H -1.774514 2.416854 -1.537236
H -4.367146 1.926900 -1.420831
H -5.547674 -1.180316 -1.100579
H -0.440387 5.177762 -1.591705
H -2.737950 -4.765690 -1.057427
H 4.208413 0.515128 -1.627307
H -1.667381 -2.529374 -1.471536
H 2.927268 -3.729150 -1.929734
H 1.621292 -7.235554 -1.544125
H -3.233679 4.262249 -2.361172
H -4.111771 -1.115925 -2.134783
H 3.237591 -5.432376 -2.283013
H 2.114088 -0.353679 -2.705465
H 0.274264 -6.415656 -2.326983
H 2.731523 3.627615 -2.019824
H 1.737250 5.071732 -2.151017
H -1.812302 -5.458764 -2.388101
H 0.228781 -2.344372 -3.142947
H 1.933856 -4.578559 -3.105998
H -3.011230 -4.207531 -2.708550
H -2.867932 3.144056 -3.674816
H -1.308353 -0.287277 -3.638061
H -2.560002 0.941959 -3.706641
H 2.583093 1.236350 -3.256241
H -2.043084 4.700533 -3.582905
H -0.126430 -3.989924 -3.684737
H 3.126147 4.868899 -3.216747
H -1.326138 -2.719416 -3.898581
H 0.146696 5.376897 -4.178878
H -2.154526 0.022187 -5.161764
H 2.074691 3.009860 -4.386330
H 0.936486 0.109690 -5.060036
H -1.161771 2.345323 -5.199358
H 0.185884 4.215312 -5.508049
H 1.568325 5.272742 -5.213409
H 1.117816 1.639874 -5.924554
H -0.086664 0.461282 -6.452093

Table S52. Final coordinates for TS(bU4)

U -0.422847 -0.321327 0.694211
Si -0.948413 -0.325012 4.542218
Si 1.791055 -3.146757 -0.774516
Si -1.084288 3.010681 -1.288601
Si -3.076161 -1.721028 -1.219034
N 1.455321 -1.773900 0.273455
N -0.122279 1.686510 -0.647965
N 1.958610 0.956224 1.341654
N -0.321796 0.304977 3.028459
N -2.096259 -1.368233 0.171047
C -0.079073 -4.956648 0.479578
C 0.390413 -4.290135 -3.089989
C 4.381986 -2.078435 -1.810310
C 1.185471 -1.920987 -3.329284
C 0.686681 -2.983991 -2.341771

C 3.968807 -4.524381 -2.134093
C 3.686882 -3.314246 -1.230701
C -0.752132 -3.128493 3.778017
C -1.985697 2.284967 5.438387
C 0.494081 3.587095 -3.726797
C -3.311004 2.392045 0.498781
C 1.735211 -1.077910 5.568384
C -3.647544 0.712458 4.457384
C -2.355243 0.803738 5.277375
C 0.465052 0.459848 7.082680
C 0.337039 -0.614521 5.995966
C -2.567007 -2.570460 5.410403
C -1.734640 -2.047639 4.234779
C -2.350261 -1.063572 -2.870783
C -3.359663 -3.592968 -1.492153
C -0.936994 4.893878 0.956458
C -3.904075 3.695323 -1.553850
C -2.946896 2.647101 -0.968149
C -0.875370 5.977385 -1.299157
C -0.512783 4.713403 -0.506487
C 3.022944 -0.013088 1.038031
C 2.526831 -1.444390 1.223009
C 2.300649 -5.178556 1.243005
C 1.387206 -4.857810 0.053288
C -1.622276 2.315835 -4.113178
C -0.928485 3.338318 -3.207924
C 1.321161 1.916295 -0.834962
C 2.041002 2.149868 0.488556
C 1.923765 1.300579 2.769722
C 0.488996 1.512497 3.234344
C -4.838511 -0.970352 -1.153163
C -5.001268 -4.533632 3.195214
C -6.166326 -3.851312 3.549010
C -6.342489 -2.528931 3.142124
C -5.364964 -1.900174 2.368117
C -4.198753 -2.576836 1.997955
C -4.024903 -3.897881 2.427043
H -5.506651 -0.869955 2.051922
H -7.243461 -1.988362 3.423602
H -6.930430 -4.346405 4.143970
H -4.853931 -5.561361 3.520163
H -3.104753 -4.422203 2.179976
H 1.064251 0.092368 7.929714
H -0.504977 0.766092 7.486690
H 0.969192 1.360836 6.711153
H -2.562275 0.398575 6.281541
H -1.083445 2.447620 6.035666
H -0.139076 -1.479979 6.485887
H -2.800707 2.842338 5.924757
H -1.947629 -2.758507 6.298136
H 2.243716 -1.615228 6.382522
H -3.365286 -1.883341 5.708751
H -4.463453 1.275986 4.934592
H -3.049157 -3.522904 5.152991
H -1.822941 2.755354 4.462224
H 2.373173 -0.223221 5.315425
H -3.997157 -0.314056 4.321691
H 1.721161 -1.742090 4.699262
H -0.075504 -3.432747 4.587608
H 0.536492 1.835035 4.288766
H -3.505731 1.136154 3.456486
H -2.423115 -1.869354 3.399229
H -1.287711 -4.032428 3.450628
H -4.812267 0.124750 -1.120324
H -4.307697 1.940580 0.588861
H 0.073309 2.380252 2.695225
H -0.130820 -2.799874 2.938191
H 2.342051 0.462907 3.332160
H -1.645825 -0.248462 -2.683936
H -2.613476 1.717714 1.004005
H 2.554790 2.185368 2.971409
H -3.325965 3.323908 1.073565
H -5.401860 -1.257584 -2.051181
H -5.410606 -1.317963 -0.288430
H -3.141323 -0.677503 -3.526517
H 2.193309 -1.551266 2.269616
H -4.946685 3.350308 -1.502451
H -0.323443 -4.195700 1.225198
H 2.199223 -4.430467 2.038559
H -1.825942 -1.845151 -3.430622
H -3.112270 1.705436 -1.512562
H -2.000410 5.149184 1.035722
H 1.542491 2.976286 1.004541
H -3.857718 4.636505 -0.992019
H -0.305871 -5.939799 0.919253
H 3.916647 0.191152 1.655460
H 2.047257 -6.151442 1.689987
H -0.774788 3.992328 1.555440
H 3.403645 -2.105941 1.138898
H -0.771036 -4.803829 -0.353775
H -3.695194 3.930167 -2.602891
H -0.381248 5.713160 1.436227
H 3.360054 -5.221062 0.964760
H 3.095989 2.443297 0.335038
H -4.143151 -3.769865 -2.241075
H 3.309893 0.118064 -0.008291
H -1.957414 6.075798 -1.447792
H -3.667491 -4.093428 -0.568158
H -2.670924 2.152678 -3.843942
H -2.448446 -4.084161 -1.851304
H 1.561900 -5.624610 -0.717438
H 1.547418 2.783326 -1.474691
H -0.262148 -2.620221 -1.919747
H 1.800010 1.062874 -1.345255

H 0.586900 4.644008 -0.512537
H 4.176351 -3.527916 -0.267872
H -1.124041 1.341708 -4.076222
H -0.546837 6.882613 -0.766301
H -0.408298 6.004742 -2.288778
H -1.473830 4.288766 -3.322604
H 4.278540 -1.199219 -1.170291
H -0.055181 -5.055752 -2.447695
H 1.411649 -0.968523 -2.837873
H -1.603893 2.644740 -5.163385
H 3.538171 -5.454531 -1.749436
H 1.086643 2.665146 -3.724259
H -0.308932 -4.117176 -3.920710
H 5.459719 -2.258204 -1.941602
H 0.434852 -1.712993 -4.104389
H 1.041985 4.331816 -3.137538
H 3.984233 -1.808577 -2.793634
H 1.295825 -4.725037 -3.530788
H 2.091998 -2.251792 -3.850164
H 5.050728 -4.691936 -2.243386
H 3.572210 -4.374299 -3.145320
H 0.476469 3.948104 -4.766153
H -3.433065 -2.136686 1.332630

Table S53. Final coordinates for 4aUan

U 0.072396 -0.014772 0.012931
Si -0.658740 3.454199 -1.469723
Si 0.887619 -2.944606 -2.342588
Si 0.362029 -0.362684 3.812056
Si -3.827050 -1.085154 0.140321
N 0.407481 2.119774 -1.101354
N 1.335026 -1.697724 -1.196394
N 2.743259 0.625694 -0.032736
N 0.953630 0.171823 2.257644
N -2.089805 -1.023127 0.129347
C -2.077157 1.153293 3.986173
C -0.046742 2.338192 4.824806
C -0.733524 0.972064 4.702341
C 0.061359 -3.155433 3.132191
C -1.562033 -2.291984 4.826579
C -0.742024 -1.929088 3.581191
C -4.504816 -2.355979 1.397234
C -4.584238 0.598759 0.585555
C -0.619168 6.330114 -2.002511
C 1.233137 5.518883 -0.514321
C 0.294612 5.143658 -1.667141
C -0.652374 3.245481 -4.365438
C -2.455116 1.976075 -3.162251
C -1.597845 3.244556 -3.158391
C -0.518885 -1.312537 -4.281806
C -1.557003 -3.555551 -3.858510
C -0.739246 -2.414445 -3.237771
C 1.953840 -4.400769 -4.641731
C 2.927996 -2.107309 -4.316100
C 2.322870 -3.325717 -3.610912
C -3.295234 4.274758 -0.454279
C -1.465088 4.046532 1.250329
C -2.004537 3.530194 -0.088719
C 1.743093 2.160546 -1.703301
C 2.838896 1.953319 -0.657628
C 1.770836 -5.307748 -0.871683
C -0.605261 -4.598072 -0.501385
C 0.534701 -4.673266 -1.520281
C 2.714990 -1.759112 -0.701789
C 3.417032 -0.408598 -0.830167
C 3.208460 0.633317 1.361495
C 2.101251 1.080978 2.312934
C 2.993457 -1.539805 4.416320
C 2.171487 0.039775 6.180723
C 1.747499 -0.935379 5.076563
C -4.589023 -1.586586 -1.538613
H 2.824958 -0.456582 6.914594
H 1.317965 0.444473 6.734720
H 2.739142 0.890232 5.783278
H -0.932605 0.594965 5.718562
H 0.905330 2.293966 5.361943
H 1.229332 -1.764840 5.586659
H -0.686178 3.059762 5.355432
H -0.916021 -2.538186 5.680501
H 3.605659 -2.087089 5.148966
H -2.230052 -1.487797 5.151131
H -2.686129 1.934899 4.464727
H -2.188083 -3.177054 4.643113
H 0.155097 2.762185 3.834802
H 3.635836 -0.761197 3.989649
H -2.677097 0.238269 3.967903
H 2.748678 -2.234930 3.608057
H 0.737550 -3.505003 3.923314
H 2.555300 1.150748 3.317123
H -1.922422 1.454300 2.943721
H -1.439843 -1.674974 2.768969
H -0.601726 -3.998487 2.891311
H -4.249415 -2.081687 2.427090
H -2.182911 3.875160 2.064048
H 1.823973 2.120061 2.058977
H 0.671007 -2.950282 2.246024
H 3.484835 -0.390521 1.630987
H -4.234411 0.955735 1.559903
H -0.526892 3.556437 1.529098
H 4.112959 1.262053 1.467284
H -1.279899 5.127268 1.216417
H -5.597878 -2.438656 1.337351
H -4.092253 -3.356064 1.214696
H -5.678276 0.526298 0.631833

H -1.764430 -1.997854 0.146392
H 2.761437 -2.071082 0.357500
H -4.039757 4.192309 0.350402
H -0.342861 -3.929216 0.325745
H 2.157252 -4.688563 -0.053337
H -4.342492 1.363712 -0.159466
H -2.270339 2.470929 0.057843
H 0.677269 5.796523 0.387793
H 2.702556 2.705270 0.125799
H -3.116619 5.345908 -0.608145
H -0.826158 -5.582832 -0.062749
H 4.484884 -0.481623 -0.550504
H 1.537006 -6.292787 -0.440422
H 1.902510 4.699720 -0.236411
H 3.343948 -2.492083 -1.235768
H -1.536421 -4.223591 -0.940679
H -3.763267 3.892607 -1.367184
H 1.862992 6.381933 -0.778033
H 2.591172 -5.457438 -1.583054
H 3.844737 2.106745 -1.092591
H -5.686542 -1.590375 -1.503601
H 3.374764 -0.103977 -1.880110
H -1.257413 6.601492 -1.153060
H -4.267817 -2.591245 -1.838688
H -3.228082 1.994344 -2.386534
H -4.284151 -0.894622 -2.331906
H 0.212205 -5.336654 -2.337968
H 1.969941 3.114058 -2.209978
H -1.349892 -1.978676 -2.431564
H 1.868628 1.391053 -2.486075
H 0.930019 4.981847 -2.552108
H 3.121667 -3.754759 -2.985881
H -1.841304 1.086926 -2.980318
H -0.032094 7.226463 -2.252817
H -1.277640 6.129216 -2.854366
H -2.272149 4.109105 -3.260877
H 3.190041 -1.311969 -3.613070
H -1.829829 -4.328169 -3.132556
H 0.065787 -0.477373 -3.883262
H -2.966530 1.832448 -4.125855
H 1.559571 -5.312456 -4.179656
H 0.072776 2.425328 -4.303927
H -2.494167 -3.172823 -4.287487
H 3.842280 -2.377062 -4.865859
H -1.475749 -0.904418 -4.635834
H -0.083692 4.178454 -4.455513
H 2.236247 -1.673115 -5.045864
H -1.019322 -4.053698 -4.674560
H 0.007025 -1.695531 -5.165237
H 2.827529 -4.695967 -5.242382
H 1.196291 -4.040013 -5.348118
H -1.203641 3.112663 -5.308346

Table S54. Final coordinates for TS 3UNSiMe3-II-U

U 0.131659 0.085710 0.031034
Si 0.367593 1.531221 3.542102
Si 0.117652 2.155527 -2.999712
Si -0.115671 -3.714298 -0.309713
Si -3.471640 0.451124 -0.198803
N 0.911386 0.954499 -1.877309
N 0.671428 -2.141377 0.134192
N 2.778393 -0.125520 -0.018691
N 1.009769 1.100007 1.905973
N -1.704667 0.724285 -0.457579
C 0.772255 4.685587 -4.238419
C 1.031602 1.347340 -5.704721
C 0.512399 -6.172032 -1.724816
C -1.425688 -4.038140 2.248367
C -0.318971 -1.118359 4.514555
C -1.685739 3.315607 2.570540
C 2.768995 0.512715 4.991738
C 0.498217 4.406396 3.168712
C -0.482852 3.280930 3.513248
C 1.234030 2.045370 6.257701
C 1.795333 1.688748 4.874380
C -1.981215 0.676612 5.066576
C -0.950544 0.194185 4.037657
C -2.711416 2.758565 -3.167211
C -0.821179 0.000340 -4.696121
C -0.185319 1.388830 -4.773923
C -1.469043 3.875060 -1.299584
C -1.546789 2.633864 -2.187642
C 1.904432 4.303980 -2.022077
C 1.365429 3.635652 -3.289016
C -1.622762 -3.020655 -2.698697
C 0.699726 -5.355893 2.005089
C -0.519449 -4.787679 1.270678
C -2.817554 -4.504802 -1.069680
C -1.798172 -3.367003 -1.217493
C 3.255486 0.567279 -1.232390
C 2.220351 0.472114 -2.342985
C -3.784300 -0.721815 1.282525
C -4.280253 -0.321348 -1.761369
C 1.646596 -4.126218 -2.656695
C 1.104201 -4.796646 -1.390138
C 2.012965 -2.343316 0.724822
C 3.077462 -1.570781 -0.043410
C 3.291283 0.522628 1.206210
C 2.352723 1.643186 1.628142
C -4.438297 2.047060 0.215477
H 2.048487 2.284768 6.954864
H 0.565656 2.912118 6.234043
H 0.676960 1.209813 6.696354
H -1.515237 0.898168 6.033096

H 3.593111 0.760560 5.675104
H 2.381492 2.558758 4.542792
H -2.737080 -0.099069 5.249341
H 0.164162 -0.993391 5.489988
H -2.512004 1.578057 4.747960
H 2.283628 -0.383169 5.390442
H -0.846457 3.451246 4.537483
H 3.218274 0.237424 4.033852
H -1.081673 -1.897733 4.638543
H 1.339490 4.464457 3.868293
H 0.435626 -1.499036 3.818106
H -1.495206 -0.018643 3.104782
H -0.004539 5.382917 3.188723
H -2.463051 2.604155 2.866867
H 0.385485 -6.048810 2.797783
H -3.575461 -0.230616 2.237781
H 2.800182 2.151894 2.492725
H 1.285880 -4.567622 2.490423
H -2.147881 4.312052 2.549435
H 0.912216 4.282276 2.160916
H 3.312879 -0.226384 2.001699
H -1.677456 -4.661031 3.117817
H -3.198900 -1.644639 1.237825
H 2.034651 -2.033194 1.781810
H -0.934296 -3.135696 2.625443
H 1.373663 -5.911273 1.343587
H -1.396727 3.068524 1.544023
H -4.843591 -1.006829 1.288868
H 4.323322 0.874565 1.047383
H 2.327568 2.409129 0.835337
H 2.321841 -3.397909 0.732303
H -2.370668 -3.728936 1.789941
H 4.087240 -1.759123 0.356138
H -4.054016 2.546476 1.108995
H -1.086712 -5.641835 0.872225
H -2.405785 4.011477 -0.740733
H -5.484532 1.777296 0.408428
H -0.666582 3.809034 -0.555618
H 3.065494 -1.903255 -1.084229
H 0.162659 -6.709419 -0.836994
H -3.547456 -0.485811 -2.555096
H 3.395342 1.622603 -0.983419
H -1.671675 1.659504 -1.374498
H -4.430503 2.767339 -0.606563
H -4.750334 -1.282459 -1.527259
H -1.309493 4.807912 -1.861368
H -3.067333 -4.724956 -0.027785
H 4.234285 0.167340 -1.542995
H -2.217071 -2.480315 -0.719381
H 1.983958 -3.101177 -2.475335
H 1.259624 -6.810300 -2.215956
H -3.650552 2.969236 -2.635976
H -5.058501 0.338823 -2.159302
H 2.499572 -4.688480 -3.060572
H 2.156626 -0.568241 -2.701609
H -0.335039 -6.087440 -2.415042
H 2.275618 3.574249 -1.294911
H 1.135133 4.898809 -1.520564
H -2.454792 -5.434285 -1.523233
H -0.888063 -2.227606 -2.858632
H 0.892051 -4.080507 -3.447781
H -3.755380 -4.247755 -1.579653
H -2.880782 1.850774 -3.755668
H -0.096882 -0.734963 -4.328612
H -1.683953 -0.030010 -4.023738
H 2.608061 1.049356 -3.194626
H -2.584616 3.585089 -3.885029
H -1.299770 -3.894768 -3.274700
H 2.734365 4.984556 -2.258745
H -2.571327 -2.685077 -3.136190
H -0.084463 5.198069 -3.786071
H -1.157985 -0.343646 -5.684138
H 2.225211 3.180642 -3.804375
H 1.796697 0.649267 -5.345208
H -0.919363 2.071247 -5.228698
H 0.428143 4.255264 -5.185266
H 1.513610 5.458669 -4.484691
H 1.509077 2.324388 -5.830374
H 0.739020 1.002588 -6.706584
H 1.965885 -4.972384 -0.728589

Table S55. Final coordinates for II-U

U 0.145881 0.034992 0.015502
Si -3.797074 0.267657 0.039838
Si 0.148644 -3.760075 -0.169753
Si 0.164427 2.121203 -3.139096
Si 0.286532 1.585286 3.456295
N -2.048715 0.469417 -0.275330
N 0.877740 -2.152272 0.208361
N 0.909722 1.035510 -1.909723
N 2.830277 -0.022720 -0.013936
N 0.981493 1.157322 1.849720
C 1.177070 4.373469 3.852250
C -2.383510 1.299419 4.621271
C -1.239425 -0.034636 -4.456497
C -1.246421 3.855482 -1.204588
C 2.039160 4.310006 -2.570681
C -2.614132 3.206227 -3.230132
C -1.361641 3.004334 -2.432081
C 0.769954 4.429845 -4.742806
C 1.427678 3.501403 -3.715928
C 1.395843 -5.622758 1.782839
C -2.634297 -4.633840 -0.411087
C 1.570385 -3.925044 -2.758545

C -1.778620 -3.190876 -2.274757
C -1.680904 -3.489172 -0.775934
C 0.678680 -6.092072 -1.823670
C 1.242750 -4.706797 -1.483051
C 2.256960 -2.303753 0.713447
C 3.233050 -1.439211 -0.070893
C -4.737189 -0.286402 -1.532754
C -4.616824 1.902653 0.611679
C -4.127458 -1.020493 1.407662
C -0.551694 -4.270113 2.601354
C 0.078516 -4.948579 1.383681
C 2.086490 -0.292449 4.870356
C -1.248824 -0.828761 3.904286
C -1.413100 0.667001 3.616412
C 0.782479 0.807046 -5.684127
C -0.378527 1.191511 -4.760679
C 2.239554 0.552696 -2.332666
C 3.259373 0.716118 -1.215530
C 3.306085 0.627939 1.221208
C 2.333830 1.713365 1.656367
C -0.943085 4.043242 2.548728
C -0.063104 3.492008 3.670892
C 1.538308 1.137280 4.890019
C 0.983733 1.480701 6.278491
H 1.752510 1.347205 7.052359
H 0.632123 2.515651 6.349153
H 0.144502 0.829174 6.549016
H -1.987946 1.276515 5.643384
H 2.935985 -0.396553 5.559565
H 2.392736 1.807667 4.713549
H -3.334865 0.750257 4.638242
H -0.882111 -1.003491 4.922174
H -2.617892 2.341700 4.383354
H 1.333173 -1.022895 5.182126
H -0.639615 3.543116 4.606927
H 2.431326 -0.590557 3.875600
H -2.206572 -1.357893 3.819895
H 1.821583 4.038908 4.672363
H -0.545206 -1.309497 3.216503
H -1.866983 0.769173 2.618264
H 0.887175 5.409963 4.074948
H -1.852513 3.451637 2.399123
H 1.223060 -6.372068 2.567950
H -3.838083 -0.644694 2.392802
H 2.749539 2.167764 2.568283
H 2.116039 -4.904080 2.189805
H -1.251610 5.077865 2.753225
H 1.789084 4.407033 2.943125
H 3.348183 -0.128711 2.008758
H -0.733065 -4.990400 3.411248
H -3.610778 -1.968424 1.232311
H 2.316441 -2.044671 1.782490
H 0.109281 -3.493787 3.000641
H 1.881178 -6.140589 0.948862
H -0.401450 4.046370 1.597668
H -5.202220 -1.234838 1.441212
H 4.327430 1.020368 1.081949
H 2.332948 2.523955 0.908858
H 2.632332 -3.336280 0.648784
H -1.509886 -3.792768 2.370163
H 4.267785 -1.561026 0.292010
H -4.143893 2.292610 1.518633
H -0.603218 -5.746571 1.054469
H -2.102442 3.710401 -0.530656
H -5.677484 1.733368 0.833439
H -0.336087 3.656990 -0.632534
H 3.213185 -1.757421 -1.117178
H 0.495005 -6.703536 -0.933722
H -4.611007 0.429759 -2.351807
H 3.319579 1.777264 -0.958536
H -1.969492 1.167111 -1.032723
H -4.563505 2.680512 -0.157599
H -4.392657 -1.263581 -1.885403
H -1.246634 4.928385 -1.461962
H -2.686692 -4.817787 0.666184
H 4.265137 0.391124 -1.530836
H -2.024545 -2.591084 -0.242152
H 1.907454 -2.906487 -2.539919
H 1.372838 -6.653752 -2.464054
H -3.501811 3.252124 -2.582948
H -5.811457 -0.362960 -1.325600
H 2.367584 -4.421509 -3.328799
H 2.204275 -0.506526 -2.636795
H -0.268425 -6.019082 -2.370814
H 2.509488 3.670325 -1.817257
H 1.284804 4.916765 -2.057610
H -2.340276 -5.575053 -0.890787
H -1.125227 -2.369181 -2.579247
H 0.705287 -3.844789 -3.424206
H -3.653697 -4.407037 -0.751451
H -2.786533 2.432232 -3.983158
H -0.634784 -0.813800 -3.981013
H -2.072115 0.184395 -3.779030
H 2.630121 1.088061 -3.210572
H -2.591050 4.170191 -3.767435
H -1.510911 -4.067140 -2.875500
H 2.807586 5.001786 -2.942782
H -2.802380 -2.911960 -2.555751
H -0.051106 5.002763 -4.295124
H -1.662200 -0.467413 -5.373412
H 2.246602 2.973387 -4.225454
H 1.445028 0.068839 -5.217167
H -0.997916 1.923204 -5.300662
H 0.359973 3.885303 -5.600240

H 1.492250 5.158267 -5.135964
H 1.396731 1.666559 -5.973110
H 0.407310 0.353196 -6.611735
H 2.196115 -4.867075 -0.956980

Table S56. Final coordinates for TS II-U-4aU

U 0.105170 0.376794 -0.028842
Si 0.256351 1.826293 3.471960
Si 0.299248 -3.394858 -0.390135
Si -3.828418 0.054302 0.155349
Si 0.141270 2.555169 -3.221001
N 0.896312 1.553911 1.804195
N 0.919773 -1.763500 0.082993
N 2.787255 0.453666 -0.046012
N -2.116188 0.189660 -0.355423
N 0.803415 1.417675 -1.985718
C -0.251616 4.681226 3.244473
C 1.082695 2.465013 6.172597
C -2.140154 0.844780 -3.910057
C 0.731346 -4.760628 2.164970
C -2.115918 -4.391369 -1.682044
C 2.484872 -3.931448 -2.332372
C -0.741453 -2.795467 -3.044156
C -1.180745 -3.176285 -1.625833
C 1.263948 -5.935205 -1.435047
C 1.734406 -4.507516 -1.127188
C -4.775797 1.710157 -0.014263
C -3.994327 -0.520513 1.964690
C -1.799486 0.477348 5.075908
C 2.879267 1.341512 4.833375
C 1.662760 2.269627 4.764010
C 0.165379 -0.928238 4.396663
C -0.741349 0.239163 3.991269
C -2.178501 3.182070 2.652102
C -0.953255 3.354518 3.549974
C 2.156937 2.253139 1.499033
C 3.229282 1.239755 1.120276
C -1.483016 -3.605277 1.865819
C -0.359951 -4.370174 1.162773
C 2.232977 -1.803524 0.759533
C 3.251117 -0.946133 0.020081
C 3.159133 1.093468 -1.322110
C 2.095936 0.811182 -2.376395
C 2.650521 4.085346 -3.544986
C -4.746395 -1.208745 -0.952765
C 0.927205 4.321825 -5.352696
C 1.540608 3.369883 -4.316713
C -0.219836 0.788211 -5.517722
C -1.008398 1.659725 -4.533987
C -1.209140 3.703276 -0.898190
C -2.134510 4.412909 -3.129118
C -0.896439 3.959680 -2.361795
C 0.842846 9.044809 -1.455349
C 0.070586 8.751393 -2.579772
C -0.236076 7.422261 -2.893915
C 0.248204 6.415958 -2.071615
C 1.015925 6.683090 -0.947877
C 1.314895 8.015276 -0.640265
H -0.192250 4.981043 -2.334674
H 1.387684 5.881440 -0.314527
H 1.915914 8.245705 0.236581
H 1.076231 10.078175 -1.212752
H -0.297128 9.555245 -3.213679
H -0.844166 7.196351 -3.766731
H 1.845256 2.874515 6.849109
H 0.231460 3.153133 6.194087
H 0.751847 1.515212 6.607813
H -1.340546 0.755652 6.030856
H 3.609658 1.721471 5.561502
H 2.029611 3.253958 4.437309
H -2.382146 -0.436187 5.254489
H 0.642580 -0.740517 5.365129
H -2.509085 1.268370 4.816106
H 2.610269 0.329324 5.150143
H -1.304244 3.391610 4.591785
H 3.399482 1.256744 3.876361
H -0.409876 -1.857140 4.501743
H 0.581743 4.884150 3.925975
H 0.960023 -1.111520 3.666485
H -1.276067 -0.062431 3.076293
H -0.949990 5.525043 3.329711
H -2.749123 2.279118 2.890057
H 0.314193 -5.371480 2.977605
H -3.658461 0.237192 2.677560
H 2.537162 2.857526 2.335318
H 1.184715 -3.879492 2.632821
H -2.865911 4.033938 2.748484
H 0.143509 4.698087 2.222059
H 3.358179 0.549528 1.958262
H -1.857123 -4.155938 2.739937
H -3.442442 -1.444904 2.156082
H 2.158041 -1.442926 1.797394
H -1.130096 -2.629519 2.218676
H 1.537459 -5.343462 1.706116
H -1.891049 3.119272 1.598530
H -5.052459 -0.717024 2.175351
H 4.204284 1.715120 0.921913
H 2.032721 2.964782 0.665717
H 2.644852 -2.819225 0.837941
H -2.336256 -3.423364 1.204296
H 4.249580 -1.000309 0.483686
H -4.327369 2.502432 0.593261
H -0.786958 -5.300413 0.759326

H -1.931312 2.883456 -0.784787
H -5.813689 1.583585 0.317059
H -0.311423 3.437105 -0.327120
H 3.343579 -1.322034 -1.001533
H 0.764429 -6.411204 -0.584845
H -4.366877 -2.225537 -0.812198
H 3.199304 2.173910 -1.159789
H -2.132019 0.225139 -1.382318
H -4.803305 2.057224 -1.052819
H -5.816682 -1.214903 -0.714363
H -1.650530 4.588798 -0.423537
H -2.538777 -4.647906 -0.706076
H 4.160953 0.762519 -1.643031
H -1.755817 -2.332312 -1.217877
H 2.786869 -2.891097 -2.182501
H 2.115074 -6.574179 -1.708461
H -2.908125 3.634870 -3.138517
H -4.647960 -0.958344 -2.015097
H 3.396132 -4.510349 -2.537081
H 2.011195 -0.280944 -2.509132
H 0.567555 -5.956489 -2.281086
H 3.176903 3.421796 -2.853856
H 2.259343 4.926977 -2.964622
H -1.605767 -5.284519 -2.060549
H -0.045357 -1.949865 -3.059387
H 1.879182 -3.962497 -3.243625
H -2.958597 -4.197280 -2.359333
H -1.918413 4.666451 -4.172080
H -1.742421 -0.029485 -3.382496
H -2.739191 1.428278 -3.203245
H 2.470503 1.178558 -3.340968
H -2.583013 5.297176 -2.657708
H -0.247517 -3.633627 -3.547951
H 3.400807 4.494397 -4.236023
H -1.607382 -2.523200 -3.661903
H 0.469145 5.192762 -4.871302
H -2.826384 0.462775 -4.678499
H 2.001130 2.543019 -4.875781
H 0.302717 -0.029786 -5.007387
H -1.465355 2.478990 -5.106799
H 0.164419 3.841216 -5.974188
H 1.701914 4.705527 -6.030493
H 0.527574 1.357147 -6.079985
H -0.891768 0.324989 -6.253546
H 2.463798 -4.586786 -0.307262

Table S57. Final coordinates for TS II-U-4U

U -5.420609050 8.671265655 -3.823682742
U 0.433700000 -0.790462000 0.389064000
Si -2.900774050 11.229188655 -2.854078742
Si -5.373653050 5.483246655 -1.766945742
Si -7.992829050 9.153833655 -6.449865742
Si -2.891558050 7.469866655 -6.547386742
Si 2.572434000 0.144953000 3.299623000
Si -0.247903000 1.887128000 -2.318389000
Si -0.868864000 -4.207035000 -0.183097000
Si -2.689928000 0.003740000 2.639375000
N -4.417106050 10.330228655 -2.591681742
N -7.163332050 9.688797655 -2.036595742
N -6.075928050 6.989933655 -2.409201742
N -7.248709050 9.552624655 -4.882692742
N -3.843860050 7.673904655 -5.063489742
N 2.330376000 -0.250611000 1.579197000
N 2.632625000 -1.464606000 -0.987310000
N 0.390028000 0.373148000 -1.587780000
N 0.465345000 -3.040533000 -0.002381000
N -1.396594000 0.007133000 1.420802000
C -1.730950050 13.831264655 -2.429642742
C -2.996041050 13.017924655 -2.113961742
C -1.401734050 10.383209655 -1.973392742
C -1.296532050 8.902613655 -2.349496742
C -5.000477050 10.552780655 -1.258722742
C -6.493927050 10.816010655 -1.364641742
C -7.459866050 8.590036655 -1.100700742
C -7.408771050 7.248647655 -1.820513742
C -6.619002050 3.979385655 -1.881047742
C -7.346204050 3.504626655 -0.616495742
C -2.566826050 11.234431655 -4.746762742
C -3.440821050 12.241830655 -5.505859742
C -4.249082050 13.832881655 -2.456016742
C -7.903426050 10.637897655 -7.688756742
C -6.485791050 11.207711655 -7.787456742
C -3.221115050 8.876112655 -7.770631742
C -1.035110050 7.443328655 -6.149531742
C -3.284649050 5.849005655 -7.458102742
C -8.361229050 10.134942655 -2.770258742
C -7.965138050 10.615848655 -4.158539742
C -9.878874050 8.757890655 -6.253338742
C -10.555890050 8.450889655 -7.597844742
C -6.981841050 7.687543655 -7.181439742
C -7.321852050 6.333993655 -6.543436742
C -10.244945050 7.684737655 -5.221408742
C -7.031458050 7.577931655 -8.712131742
C -8.905857050 11.774652655 -7.442568742
C -4.766932050 5.695342655 0.066645258
C -3.655373050 6.737746655 0.247910258
C -3.944948050 4.927809655 -2.922261742
C -4.359239050 4.280381655 -4.224184742
C -5.874023050 6.013758655 1.086178258
C -2.600161050 4.990600655 -2.654916742
C -7.625791050 4.105621655 -3.033928742
C -1.094831050 11.407960655 -5.145632742
C -1.391546050 10.545152655 -0.447633742
C 4.442790000 -0.273599000 5.456214000

C	4.266297000	-0.540648000	3.953927000
C	2.637408000	2.056315000	3.585386000
C	1.433405000	2.749913000	2.939209000
C	3.547967000	-0.028777000	0.780792000
C	3.808349000	-1.209525000	-0.138656000
C	2.618890000	-0.599443000	-2.180265000
C	1.187448000	-0.332010000	-2.621854000
C	-1.066755000	1.536411000	-4.052665000
C	-0.186000000	1.687063000	-5.302952000
C	1.078515000	-0.571466000	4.274042000
C	1.198036000	-2.082742000	4.511200000
C	4.582118000	-2.004391000	3.627038000
C	-0.817047000	-5.594844000	1.162907000
C	-0.777120000	-5.023368000	2.583613000
C	-2.617397000	-1.559168000	3.701205000
C	-2.554802000	1.525615000	3.766358000
C	-4.408866000	0.062376000	1.832314000
C	2.527966000	-2.894689000	-1.332923000
C	1.792687000	-3.638392000	-0.226692000
C	-0.745085000	-5.129314000	-1.882670000
C	-1.810674000	-6.223322000	-2.052757000
C	-2.509787000	-3.227785000	0.036959000
C	-2.878281000	-2.360253000	-1.174292000
C	-0.704839000	-4.239837000	-3.131064000
C	-3.711221000	-4.090443000	0.450119000
C	0.287524000	-6.648128000	0.994686000
C	1.262580000	3.067735000	-2.594632000
C	0.913455000	4.440174000	-3.183108000
C	-1.618272000	2.759007000	-1.304619000
C	-2.943339000	2.017606000	-1.209693000
C	2.150588000	3.248077000	-1.357144000
C	-1.212642000	3.441003000	-0.003908000
C	-1.822330000	0.201332000	-4.121429000
C	0.769361000	0.147252000	5.594779000
C	3.938743000	2.730027000	3.129903000
H	-10.305270050	9.706749655	-5.891618742
H	-7.886908050	2.570890655	-0.822095742
H	-6.662282050	3.301846655	0.213365258
H	-3.423195050	6.872837655	1.312731258
H	-2.719652050	6.470246655	-0.245850742
H	-5.928669050	3.163993655	-2.145581742
H	-8.093832050	4.227233655	-0.268583742
H	-4.357146050	4.707676655	0.329860258
H	-8.130886050	3.147084655	-3.212824742
H	-3.963429050	7.714514655	-0.141350742
H	-6.599760050	5.564356655	-6.844749742
H	-5.470388050	5.969726655	2.106319258
H	-8.310770050	5.982129655	-6.857317742
H	-4.934645050	3.360322655	-4.066383742
H	-7.706484050	6.472972655	-1.098880742
H	-7.159759050	4.413467655	-3.973073742
H	-8.405515050	4.839295655	-2.803728742

H	-4.991559050	4.946317655	-4.822294742
H	-6.396980050	6.753954655	-9.064092742
H	-9.985944050	6.680921655	-5.571717742
H	-7.324136050	6.369505655	-5.447853742
H	-8.046189050	7.373816655	-9.072310742
H	-6.259329050	7.029189655	0.949343258
H	-6.722411050	5.330359655	1.042311258
H	-10.208862050	7.496360655	-8.010042742
H	-8.190322050	7.229947655	-2.596157742
H	-11.324545050	7.684734655	-5.020131742
H	-2.743858050	5.818553655	-8.411374742
H	-3.487943050	4.017742655	-4.834019742
H	-11.644721050	8.368383655	-7.480613742
H	-9.730068050	7.834271655	-4.267969742
H	-5.939249050	7.917122655	-6.909311742
H	-8.433275050	8.750960655	-0.607378742
H	-6.686168050	8.489921655	-9.209101742
H	-2.041615950	3.829606345	-2.098229258
H	-4.352880050	5.761089655	-7.677788742
H	-0.797644050	6.697650655	-5.383453742
H	-3.492844050	6.973784655	-4.393363742
H	-6.690590050	8.594492655	-0.324118742
H	-2.986115050	4.968657655	-6.881858742
H	-10.367337050	9.221746655	-8.351971742
H	-9.034733050	9.281178655	-2.879506742
H	-2.217764050	5.598619655	-1.839166742
H	-0.455423050	7.194369655	-7.045773742
H	-1.902731050	4.866369655	-3.486406742
H	-8.154757050	10.190982655	-8.663020742
H	-0.689853050	8.416225655	-5.787800742
H	-8.899427050	10.910522655	-2.199575742
H	-5.744710050	10.447762655	-8.053030742
H	-8.882951050	10.945783655	-4.667412742
H	-4.832855050	9.691701655	-0.591100742
H	-6.951164050	11.007764655	-0.378942742
H	-2.493557050	8.814629655	-8.587913742
H	-2.164816050	8.348392655	-1.975827742
H	-9.943024050	11.426638655	-7.398437742
H	-6.174003050	11.640463655	-6.831194742
H	-7.342059050	11.519666655	-4.067834742
H	-4.216779050	8.800153655	-8.216477742
H	-6.425677050	12.001821655	-8.543800742
H	-6.642725050	11.708090655	-1.979740742
H	-2.278441050	10.093947655	0.012693258
H	-0.399673050	8.442095655	-1.912992742
H	-8.694371050	12.305458655	-6.507372742
H	-4.553390050	11.409464655	-0.734409742
H	-3.122167050	9.862794655	-7.311356742
H	-8.848947050	12.520510655	-8.246761742
H	-1.256756050	8.746377655	-3.431500742
H	-0.517256050	10.047535655	-0.006517742
H	-2.867167050	10.221956655	-5.061269742

H -4.495261050 12.179196655 -5.217906742
H -1.355578050 11.594405655 -0.136705742
H -3.383860050 12.083102655 -6.590745742
H -5.171534050 13.305823655 -2.195718742
H -0.509439050 10.894882655 -2.365152742
H -4.301810050 14.070699655 -3.522762742
H -0.445742050 10.650582655 -4.695813742
H -3.111288050 13.270389655 -5.320899742
H -0.976193050 11.335346655 -6.234928742
H -3.000701050 12.864869655 -1.024184742
H -4.252988050 14.787664655 -1.912761742
H -0.706623050 12.388557655 -4.848260742
H -1.662205050 14.069515655 -3.496974742
H -0.811079050 13.306813655 -2.149584742
H -1.736882050 14.787931655 -1.890244742
H 0.228280000 -5.641623000 -1.827361000
H -0.800265000 1.640335000 -6.212249000
H 0.363891000 2.631833000 -5.331631000
H 1.821141000 5.008393000 -3.426052000
H 0.321122000 4.372493000 -4.101430000
H -1.821256000 2.336490000 -4.105725000
H 0.550400000 0.879162000 -5.387339000
H 1.863508000 2.534066000 -3.348502000
H -2.432859000 0.144692000 -5.032636000
H 0.344380000 5.043616000 -2.467121000
H -3.710092000 -1.686300000 -0.934163000
H 3.127737000 3.670924000 -1.626191000
H -3.198555000 -2.977391000 -2.020652000
H -3.325295000 1.745249000 -2.198440000
H 1.246589000 0.234464000 -3.561389000
H -2.486551000 0.043426000 -3.267930000
H -1.128927000 -0.645593000 -4.148284000
H -2.888117000 1.090820000 -0.625895000
H -4.603246000 -3.467758000 0.598927000
H -1.684759000 -3.804397000 -3.349185000
H -2.048306000 -1.735067000 -1.525003000
H -3.964306000 -4.82955500 -0.318264000
H 1.698636000 3.935714000 -0.634205000
H 2.321271000 2.302213000 -0.835378000
H -2.813260000 -5.79212200 -2.154148000
H 0.715141000 -1.286249000 -2.903703000
H -0.406537000 -4.817217000 -4.016321000
H -5.187624000 0.031228000 2.603230000
H -3.707612000 2.650414000 -0.736297000
H -1.626011000 -6.812204000 -2.961247000
H -0.001896000 -3.408478000 -3.025113000
H -2.302412000 -2.539129000 0.870211000
H 3.213056000 -1.051145000 -2.992876000
H -3.536568000 -4.636028000 1.382669000
H -0.415509000 4.175305000 -0.156753000
H -4.564401000 -0.790287000 1.164331000
H -2.543515000 2.451098000 3.180746000
H -1.552796000 0.862390000 0.873712000
H 3.088076000 0.353587000 -1.919506000
H -4.556386000 0.977440000 1.250501000
H -1.837374000 -6.923647000 -1.211799000
H 1.949276000 -2.986007000 -2.255642000
H -0.843136000 2.744122000 0.759874000
H -3.408745000 1.576158000 4.451681000
H -2.063950000 3.971356000 0.443620000
H -1.779803000 -6.114357000 1.038608000
H -1.642498000 1.498522000 4.369179000
H 3.527583000 -3.319267000 -1.525850000
H -1.594261000 -4.322280000 2.779772000
H 1.747961000 -4.698685000 -0.516020000
H 3.479874000 0.892306000 0.180383000
H 4.709144000 -1.060276000 -0.758032000
H -3.329933000 -1.468114000 4.528694000
H 1.462506000 2.635869000 1.849388000
H 0.288993000 -7.109327000 0.001419000
H 0.159301000 -4.484409000 2.761628000
H 2.395830000 -3.614664000 0.694461000
H -2.895479000 -2.446913000 3.125867000
H -0.842852000 -5.821103000 3.335650000
H 3.975005000 -2.096916000 0.477824000
H 4.100068000 2.611906000 2.052202000
H 1.427573000 3.826689000 3.156209000
H 1.281918000 -6.219553000 1.163208000
H 4.449296000 0.103992000 1.396732000
H -1.627220000 -1.724802000 4.131923000
H 0.165762000 -7.457275000 1.727293000
H 0.477703000 2.339622000 3.281615000
H 3.908319000 3.809857000 3.329031000
H 0.219619000 -0.399753000 3.605979000
H 1.428576000 -2.632391000 3.592709000
H 4.820923000 2.333528000 3.642931000
H 0.269251000 -2.500094000 4.921412000
H 4.460018000 -2.224944000 2.562734000
H 2.561629000 2.193801000 4.675254000
H 3.933233000 -2.693984000 4.175124000
H 0.585219000 1.216858000 5.456068000
H 1.988957000 -2.306731000 5.235083000
H -0.123623000 -0.279768000 6.070129000
H 5.024666000 0.070034000 3.441134000
H 5.616983000 -2.252081000 3.899370000
H 1.591067000 0.048331000 6.312943000
H 3.754516000 -0.879183000 6.056623000
H 4.269277000 0.775905000 5.716658000
H 5.459567000 -0.527659000 5.784625000

Table S58. Final coordinates for 4bU

U -1.665006 0.032749 -1.633523
Si -2.801170 0.091292 2.102666
Si 1.825380 0.732707 -0.610471

Si -2.679887 -3.551137 -2.273360
Si -4.211115 2.707305 -2.237524
N -2.591976 -0.299695 0.382180
N 0.477076 0.829833 -1.769352
N -1.733466 -2.070014 -2.556021
N -0.881208 0.196306 -4.194718
N -3.034532 1.480770 -2.781492
C -6.621273 1.693372 -3.536721
C -4.835912 3.870157 0.366461
C -0.123073 -4.923104 -1.904013
C -4.667288 -3.723745 -0.201237
C -3.836637 -3.890515 -4.973356
C -2.361008 -3.792200 0.644521
C -3.357120 -3.628506 -0.480043
C -5.082843 -2.429503 -3.391840
C -4.179844 -3.666336 -3.492661
C 3.231835 3.287529 -1.028684
C 1.906854 0.065773 2.228367
C 3.170637 -1.446867 -2.091296
C 1.139596 -1.764269 0.686884
C 1.176966 -0.247221 0.914462
C 4.584346 -0.148490 -0.464364
C 3.385282 -0.084057 -1.422564
C 0.858085 1.481000 -3.034393
C 0.543638 0.573862 -4.215648
C -2.150867 -1.267897 3.261252
C -4.640698 0.318994 2.514262
C -1.870567 1.676099 2.555202
C 1.256174 3.328777 0.518673
C 2.412994 2.477919 -0.013478
C -2.947142 4.797672 -3.905635
C -2.473226 4.160682 -0.452402
C -3.706046 3.247854 -0.465653
C -1.633916 -6.065659 -3.553454
C -1.582842 -5.168147 -2.311670
C -0.860871 -2.224514 -3.740052
C -1.109734 -1.137953 -4.776446
C -1.714242 1.222112 -4.845937
C -3.104482 1.240333 -4.233651
C -6.097142 0.774280 -1.255060
C -6.009562 2.003219 -2.164452
C -4.291781 4.201354 -3.473709
C -5.228990 5.308998 -2.969069
H -5.386860 6.072389 -3.742693
H -6.214250 4.925914 -2.683625
H -4.809796 5.822867 -2.096782
H -5.176270 4.821444 -0.056993
H -3.086566 5.546928 -4.696681
H -4.762064 3.787294 -4.378634
H -4.494781 4.080119 1.388923
H -2.710519 5.150919 -0.856830
H -5.710132 3.216461 0.442153
H -2.438810 5.298464 -3.076168
H -6.613153 2.803499 -1.708769
H -2.261716 4.037620 -4.290876
H -2.100720 4.318768 0.568206
H -6.647885 2.569659 -4.193024
H -1.647329 3.752934 -1.045064
H -3.432549 2.297541 0.019516
H -7.654489 1.335166 -3.433729
H -5.738974 0.975979 -0.241382
H 3.606085 4.214012 -0.572692
H -2.112846 2.508541 1.890707
H -3.682790 2.007891 -4.767468
H 2.626576 3.585622 -1.892264
H -7.129672 0.408132 -1.175022
H -6.063276 0.906074 -4.056408
H -1.246805 2.194329 -4.668060
H 1.620906 4.270092 0.951259
H -0.787400 1.526451 2.540246
H 0.334793 2.442499 -3.157447
H 0.560667 3.588237 -0.286089
H 4.101788 2.739935 -1.405871
H -5.489412 -0.045013 -1.653336
H -2.143184 1.972317 3.574482
H -1.746037 1.060293 -5.936927
H -3.615028 0.291486 -4.467391
H 1.928985 1.727072 -3.090702
H 0.676010 2.817633 1.293481
H 0.805215 1.042756 -5.179305
H -5.055452 1.211434 2.036661
H 3.080990 2.255142 0.833123
H -5.018182 -3.893840 0.818319
H -4.778390 0.424269 3.596520
H -5.444457 -3.662367 -0.956493
H 1.141223 -0.336563 -4.121356
H 4.823504 0.823014 -0.020087
H -2.736018 -2.188657 3.184284
H -2.155815 -1.193282 -5.090724
H -3.263270 -1.055209 0.180638
H -5.231184 -0.543839 2.187810
H -1.105112 -1.513568 3.052720
H 1.863593 1.126963 2.491145
H -0.484523 -1.282753 -5.673808
H 0.134111 0.086737 1.033569
H 2.280824 -1.460181 -2.727260
H 5.485250 -0.498260 -0.986109
H -2.866082 -3.940324 1.605328
H -2.212073 -0.922501 4.300022
H 4.031822 -1.717490 -2.716723
H 0.201083 -2.210396 -3.449895
H 4.403195 -0.849968 0.358256
H -3.253018 -4.794661 -5.152927
H -3.273312 -3.046315 -5.385861

H 2.964961 -0.215649 2.178388
H 0.643275 -2.041386 -0.250297
H 3.048408 -2.245826 -1.353917
H 1.465969 -0.495983 3.062292
H -1.709676 -2.917791 0.736291
H 0.441201 -4.446193 -2.711624
H -0.030165 -4.281260 -1.023764
H -1.002541 -3.191792 -4.246697
H -1.712058 -4.659361 0.478249
H 2.150800 -2.183710 0.654256
H -4.756198 -3.971558 -5.568353
H 0.609069 -2.271915 1.502247
H -4.545515 -1.534314 -3.724311
H 0.380939 -5.872209 -1.678186
H -4.752561 -4.541627 -3.148133
H -1.201026 -5.580328 -4.435972
H -2.050085 -5.749399 -1.501440
H -5.433528 -2.229078 -2.377133
H -5.968093 -2.535325 -4.032695
H -2.654173 -6.371262 -3.804321
H -1.054951 -6.983298 -3.382303
H 3.657718 0.622179 -2.222368

Table S59. Final coordinates for TS 8UNSiMe3-II-Uan

C -4.438297000 2.047060000 0.215477000
Si -3.471640000 0.451124000 -0.198803000
N -1.704667000 0.724285000 -0.457579000
U 0.131659000 0.085710000 0.031034000
N 1.009769000 1.100007000 1.905973000
C 2.352723000 1.643186000 1.628142000
C 3.291283000 0.522628000 1.206210000
N 2.778393000 -0.125520000 -0.018691000
C 3.077462000 -1.570781000 -0.043410000
C 2.012965000 -2.343316000 0.724822000
N 0.671428000 -2.141377000 0.134192000
Si -0.115671000 -3.714298000 -0.309713000
C 1.104201000 -4.796646000 -1.390138000
C 1.646596000 -4.126218000 -2.656695000
C -4.280253000 -0.321348000 -1.761369000
C -3.784300000 -0.721815000 1.282525000
N 0.911386000 0.954499000 -1.877309000
C 2.220351000 0.472114000 -2.342985000
C 3.255486000 0.567279000 -1.232390000
C -1.798172000 -3.367003000 -1.217493000
C -2.817554000 -4.504802000 -1.069680000
C -0.519449000 -4.787679000 1.270678000
C 0.699726000 -5.355893000 2.005089000
C -1.622762000 -3.020655000 -2.698697000
Si 0.117652000 2.155527000 -2.999712000
C 1.365429000 3.635652000 -3.289016000
C 1.904432000 4.303980000 -2.022077000
C -1.546789000 2.633864000 -2.187642000
C -1.469043000 3.875060000 -1.299584000
C -0.185319000 1.388830000 -4.773923000
C -0.821179000 0.000340000 -4.696121000
C -2.711416000 2.758565000 -3.167211000
Si 0.367593000 1.531221000 3.542102000
C -0.950544000 0.194185000 4.037657000
C -1.981215000 0.676612000 5.066576000
C 1.795333000 1.688748000 4.874380000
C 1.234030000 2.045370000 6.257701000
C -0.482852000 3.280930000 3.513248000
C 0.498217000 4.406396000 3.168712000
C 2.768995000 0.512715000 4.991738000
C -1.685739000 3.315607000 2.570540000
C -0.318971000 -1.118359000 4.514555000
C -1.425688000 -4.038140000 2.248367000
C 0.512399000 -6.172032000 -1.724816000
C 1.031602000 1.347340000 -5.704721000
C 0.772255000 4.685587000 -4.238419000
H 1.965885000 -4.972384000 -0.728589000
H 0.739020000 1.002588000 -6.706584000
H 1.509077000 2.324388000 -5.830374000
H 1.513610000 5.458669000 -4.484691000
H 0.428143000 4.255264000 -5.185266000
H -0.919363000 2.071247000 -5.228698000
H 1.796697000 0.649267000 -5.345208000
H 2.225211000 3.180642000 -3.804375000
H -1.157985000 -0.343646000 -5.684138000
H -0.084463000 5.198069000 -3.786071000
H -2.571327000 -2.685077000 -3.136190000
H 2.734365000 4.984556000 -2.258745000
H -1.299770000 -3.894768000 -3.274700000
H -2.584616000 3.585089000 -3.885029000
H 2.608061000 1.049356000 -3.194626000
H -1.683953000 -0.030010000 -4.023738000
H -0.096882000 -0.734963000 -4.328612000
H -2.880782000 1.850774000 -3.755668000
H -3.755380000 -4.247755000 -1.579653000
H 0.892051000 -4.080507000 -3.447781000
H -0.888063000 -2.227606000 -2.858632000
H -2.454792000 -5.434285000 -1.523233000
H 1.135133000 4.898809000 -1.520564000
H 2.275618000 3.574249000 -1.294911000
H -0.335039000 -6.087440000 -2.415042000
H 2.156626000 -0.568241000 -2.701609000
H 2.499572000 -4.688480000 -3.060572000
H -5.058501000 0.338823000 -2.159302000
H -3.650552000 2.969236000 -2.635976000
H 1.259624000 -6.810300000 -2.215956000
H 1.983958000 -3.101177000 -2.475335000
H -2.217071000 -2.480315000 -0.719381000
H 4.234285000 0.167340000 -1.542995000

H	-3.067333000	-4.724956000	-0.027785000
H	-1.309493000	4.807912000	-1.861368000
H	-4.750334000	-1.282459000	-1.527259000
H	-4.430503000	2.767339000	-0.606563000
H	-1.671675000	1.659504000	-1.374498000
H	3.395342000	1.622603000	-0.983419000
H	-3.547456000	-0.485811000	-2.555096000
H	0.162659000	-6.709419000	-0.836994000
H	3.065494000	-1.903255000	-1.084229000
H	-0.666582000	3.809034000	-0.555618000
H	-5.484532000	1.777296000	0.408428000
H	-2.405785000	4.011477000	-0.740733000
H	-1.086712000	-5.641835000	0.872225000
H	-4.054016000	2.546476000	1.108995000
H	4.087240000	-1.759123000	0.356138000
H	-2.370668000	-3.728936000	1.789941000
H	2.321841000	-3.397909000	0.732303000
H	2.327568000	2.409129000	0.835337000
H	4.323322000	0.874565000	1.047383000
H	-4.843591000	-1.006829000	1.288868000
H	-1.396727000	3.068524000	1.544023000
H	1.373663000	-5.911273000	1.343587000
H	-0.934296000	-3.135696000	2.625443000
H	2.034651000	-2.033194000	1.781810000
H	-3.198900000	-1.644639000	1.237825000
H	-1.677456000	-4.661031000	3.117817000
H	3.312879000	-0.226384000	2.001699000
H	0.912216000	4.282276000	2.160916000
H	-2.147881000	4.312052000	2.549435000
H	1.285880000	-4.567622000	2.490423000
H	2.800182000	2.151894000	2.492725000
H	-3.575461000	-0.230616000	2.237781000
H	0.385485000	-6.048810000	2.797783000
H	-2.463051000	2.604155000	2.866867000
H	-0.004539000	5.382917000	3.188723000
H	-1.495206000	-0.018643000	3.104782000
H	0.435626000	-1.499036000	3.818106000
H	1.339490000	4.464457000	3.868293000
H	-1.081673000	-1.897733000	4.638543000
H	3.218274000	0.237424000	4.033852000
H	-0.846457000	3.451246000	4.537483000
H	2.283628000	-0.383169000	5.390442000
H	-2.512004000	1.578057000	4.747960000
H	0.164162000	-0.993391000	5.489988000
H	-2.737080000	-0.099069000	5.249341000
H	2.381492000	2.558758000	4.542792000
H	3.593111000	0.760560000	5.675104000
H	-1.515237000	0.898168000	6.033096000
H	0.676960000	1.209813000	6.696354000
H	0.565656000	2.912118000	6.234043000
H	2.048487000	2.284768000	6.954864000

Table S60. Final coordinates for II-Uan

U	0.039453	0.226259	-0.269415
Si	-3.898629	-0.370696	0.368496
Si	0.043391	-3.663826	-0.414221
Si	0.314098	1.956916	-3.023537
Si	0.264801	1.924567	3.238736
N	-2.213805	-0.014318	0.063630
N	0.680791	-2.059295	-0.047240
N	1.279558	0.801452	-2.114421
N	2.841044	-0.028023	-0.016333
N	0.875949	1.399353	1.673016
C	1.731212	4.465859	3.772610
C	-2.392278	2.314031	4.402683
C	-0.872315	-0.175154	-4.558589
C	-0.894964	3.373030	-0.846063
C	2.166878	4.194024	-2.427040
C	-2.488795	2.282199	-2.398379
C	-1.082293	2.194970	-1.804703
C	0.517156	4.545308	-4.290348
C	1.375506	3.513026	-3.546735
C	1.159152	-5.464026	1.713644
C	-2.641901	-4.736226	-0.888675
C	1.668770	-3.883899	-2.855983
C	-1.717029	-3.252722	-2.681610
C	-1.724559	-3.540584	-1.176447
C	0.808747	-6.059055	-1.917673
C	1.263697	-4.632808	-1.581390
C	1.960016	-2.216485	0.669799
C	3.105586	-1.469124	0.004502
C	-4.880516	-0.714831	-1.232059
C	-4.805418	1.081848	1.214300
C	-4.146326	-1.894570	1.474259
C	-0.826232	-4.048033	2.306843
C	-0.110315	-4.789655	1.173217
C	1.555571	-0.327240	4.650550
C	-1.754899	-0.045791	3.883976
C	-1.583845	1.416134	3.456240
C	0.741605	1.235926	-5.844614
C	-0.307473	1.237205	-4.725524
C	2.649502	0.457391	-2.416118
C	3.492485	0.626020	-1.156890
C	3.202011	0.625112	1.247392
C	2.282689	1.808519	1.522311
C	-0.422498	4.676787	2.518676
C	0.342533	3.849086	3.556599
C	1.370163	1.195095	4.675953
C	0.969197	1.650178	6.085693
H	1.694394	1.306073	6.838679
H	0.904334	2.739665	6.180629
H	-0.004474	1.239290	6.379438
H	-1.992652	2.297451	5.424448
H	2.436400	-0.636682	5.232303

H 2.360442 1.632404 4.472483
H -3.434693 1.970080 4.469593
H -1.392681 -0.207605 4.906248
H -2.420950 3.359533 4.081101
H 0.694810 -0.848591 5.082352
H -0.196307 3.948985 4.512128
H 1.677758 -0.707926 3.631949
H -2.815585 -0.330952 3.880127
H 2.325643 3.934435 4.524343
H -1.224498 -0.741209 3.226886
H -2.010595 1.510509 2.447579
H 1.649350 5.510331 4.109835
H -1.429247 4.292543 2.325377
H 0.905126 -6.216677 2.474937
H -3.507619 -1.871439 2.361063
H 2.691615 2.312807 2.413279
H 1.828186 -4.745816 2.200917
H -0.529372 5.723285 2.841920
H 2.315014 4.484359 2.845223
H 3.080697 -0.095919 2.060386
H -1.025945 -4.709338 3.163241
H -3.946035 -2.823937 0.932008
H 1.878237 -1.874660 1.715420
H -0.219214 -3.212539 2.673411
H 1.739791 -5.977080 0.938620
H 0.102036 4.690159 1.557876
H -5.188378 -1.935882 1.816453
H 4.264408 0.932428 1.237991
H 2.402985 2.546612 0.709463
H 2.282492 -3.266443 0.747087
H -1.784021 -3.624784 1.989045
H 4.067586 -1.684933 0.506007
H -4.433798 1.255462 2.229665
H -0.772943 -5.602448 0.838807
H -1.635614 3.342906 -0.030192
H -5.883825 0.888288 1.282863
H 0.090443 3.385031 -0.362893
H 3.188783 -1.818427 -1.028854
H 0.570032 -6.649799 -1.026204
H -4.896927 0.158743 -1.893376
H 3.557214 1.694974 -0.931858
H -2.239692 0.792874 -0.595898
H -4.673964 2.014750 0.653547
H -4.443066 -1.546517 -1.795251
H -1.022161 4.366331 -1.321178
H -2.794429 -4.910514 0.181188
H 4.525128 0.253389 -1.289878
H -2.153075 -2.657624 -0.683316
H 1.884444 -2.829928 -2.654482
H 1.585939 -6.609345 -2.469135
H -3.268199 2.274857 -1.615190
H -5.922628 -0.976496 -1.006199
H 2.562458 -4.328009 -3.318769
H 2.741875 -0.579899 -2.783924
H -0.084978 -6.058317 -2.553404
H 2.771478 3.481235 -1.857434
H 1.508899 4.701406 -1.713080
H -2.250556 -5.667526 -1.317495
H -1.060129 -2.419449 -2.940934
H 0.875950 -3.909405 -3.611208
H -3.635134 -4.579790 -1.334007
H -2.732346 1.459332 -3.080503
H -0.075752 -0.878358 -4.290613
H -1.629972 -0.232247 -3.770506
H 3.099698 1.085300 -3.204668
H -2.671269 3.218606 -2.960954
H -1.388025 -4.125064 -3.259162
H 2.851839 4.958273 -2.825761
H -2.723880 -2.998394 -3.041467
H -0.226747 4.997711 -3.623261
H -1.334796 -0.545109 -5.486427
H 2.113424 3.121848 -4.265763
H 1.621520 0.638329 -5.572909
H -1.132418 1.895152 -5.042223
H -0.031572 4.108580 -5.132462
H 1.126599 5.368852 -4.693215
H 1.096437 2.240767 -6.097769
H 0.337831 0.796575 -6.769773
H 2.173887 -4.723996 -0.967292

Table S61. Final coordinates for TS II-Uan-4aUan

U -0.175444 0.301287 -0.152113
Si -0.426368 1.769716 3.332542
Si 0.999477 -3.260607 -0.448657
Si -3.861222 -0.964224 0.341948
Si -0.667321 2.865995 -3.083861
N 0.312397 1.590570 1.726677
N 1.283136 -1.557856 -0.078177
N 2.385899 1.113095 -0.212248
N -2.403323 0.028197 0.228713
N 0.040472 1.492301 -2.057220
C -1.138974 4.600741 3.254247
C 0.341492 2.514123 6.054836
C -3.550753 2.704840 -2.510512
C 1.182624 -4.504522 2.171677
C -1.200373 -4.493136 -1.952122
C 3.617667 -3.604247 -1.826834
C 0.415840 -3.116386 -3.296595
C -0.252178 -3.286339 -1.923352
C 2.328790 -5.682787 -1.258718
C 2.633885 -4.236961 -0.837962
C -5.375967 0.104855 -0.082725
C -4.176641 -1.646357 2.095296

C -2.419458 0.254959 4.837164
C 2.116621 1.285674 4.766313
C 0.918609 2.238697 4.659519
C -0.391426 -1.024663 4.119960
C -1.338169 0.124432 3.755695
C -2.928828 3.018313 2.503048
C -1.733077 3.198635 3.442889
C 1.322493 2.619038 1.396504
C 2.630120 1.979517 0.951213
C -1.025530 -3.493180 1.557038
C 0.199438 -4.238114 1.024778
C 2.583347 -1.230341 0.528280
C 3.246123 -0.083529 -0.217505
C 2.449260 1.826993 -1.506081
C 1.437127 1.172620 -2.434789
C 1.521479 3.603081 -4.885996
C -3.825901 -2.511979 -0.754262
C -0.642653 3.243430 -6.044241
C 0.214128 2.795922 -4.848515
C -2.685795 1.009566 -4.150279
C -2.502815 2.416494 -3.575784
C -1.028886 4.675237 -0.899615
C -0.942118 5.687716 -3.152608
C -0.450920 4.523874 -2.298563
C 4.381547 7.770722 -1.407245
C 3.858373 7.645391 -2.695459
C 2.719467 6.867419 -2.916295
C 2.071887 6.190588 -1.873249
C 2.613345 6.340940 -0.587714
C 3.751200 7.114886 -0.348387
H 1.161199 5.400691 -2.072281
H 2.131569 5.846005 0.255170
H 4.145496 7.211614 0.662275
H 5.273200 8.369447 -1.232285
H 4.340348 8.157176 -3.527438
H 2.325159 6.793080 -3.929726
H 1.110628 2.921016 6.727076
H -0.483389 3.233951 6.040299
H -0.033418 1.596936 6.525086
H -1.991226 0.533053 5.808204
H 2.945811 1.751971 5.317290
H 1.316568 3.196718 4.292519
H -2.943462 -0.699935 4.981863
H 0.104621 -0.840364 5.079583
H -3.176814 1.005367 4.591530
H 1.859278 0.367243 5.303229
H -2.113703 3.137079 4.474876
H 2.500833 0.982725 3.787858
H -0.938632 -1.970982 4.226712
H -0.350260 4.827477 3.980692
H 0.390886 -1.179401 3.370272
H -1.845757 -0.139273 2.815557
H -1.911687 5.374681 3.367253
H -3.400981 2.035502 2.601739
H 0.669988 -4.947236 3.038022
H -4.407658 -0.853076 2.815475
H 1.547377 3.286979 2.239742
H 1.655090 -3.579491 2.524928
H -3.705333 3.771145 2.700210
H -0.715518 4.728217 2.252246
H 3.025449 1.360221 1.761621
H -1.457265 -3.996708 2.433693
H -3.320572 -2.208269 2.481585
H 2.477235 -0.946888 1.589388
H -0.764587 -2.474994 1.860397
H 1.984045 -5.195541 1.888225
H -2.628441 3.148871 1.457647
H -5.036483 -2.329388 2.082835
H 3.387503 2.743934 0.714856
H 0.978513 3.282487 0.592009
H 3.289761 -2.074489 0.525993
H -1.816474 -3.416090 0.805088
H 4.242014 0.152066 0.193311
H -5.351207 1.050185 0.473130
H -0.140419 -5.209127 0.633791
H -2.118287 4.893452 -0.861683
H -6.310054 -0.404434 0.184744
H -0.890778 3.780985 -0.277210
H 3.387925 -0.392669 -1.257897
H 1.705037 -6.213229 -0.530743
H -3.239090 -3.312803 -0.295185
H 2.159660 2.872916 -1.371916
H -2.683023 0.973173 0.518641
H -5.420544 0.351484 -1.148424
H -4.849951 -2.896931 -0.845486
H -0.555669 5.518987 -0.357093
H -1.800229 -4.583874 -1.041867
H 3.476625 1.800289 -1.905819
H -0.874049 -2.388758 -1.763367
H 3.890899 -2.581053 -1.556638
H 3.255121 -6.263803 -1.373891
H -2.044734 5.823678 -3.166695
H -3.450309 -2.347257 -1.765700
H 4.549376 -4.186185 -1.876014
H 1.607972 0.080279 -2.394235
H 1.810991 -5.720548 -2.223912
H 2.231389 3.339677 -4.096351
H 1.319892 4.671195 -4.755670
H -0.647505 -5.433307 -2.069059
H 1.245664 -2.403160 -3.274710
H 3.210328 -3.573102 -2.842629
H -1.900037 -4.432068 -2.797173
H -0.629261 5.617310 -4.203923
H -3.400689 2.109996 -1.605439

H -3.545259 3.760295 -2.220119
H 1.657811 1.433361 -3.471273
H -0.537485 6.652848 -2.784506
H 0.810688 -4.070313 -3.666636
H 2.041050 3.483724 -5.850362
H -0.301963 -2.758818 -4.046079
H -0.932537 4.298006 -5.970187
H -4.559910 2.483461 -2.889947
H 0.448738 1.733372 -5.025041
H -2.506762 0.251908 -3.378107
H -2.686265 3.149842 -4.377385
H -1.559935 2.658910 -6.160703
H -0.077572 3.140083 -6.983683
H -1.989175 0.794113 -4.969157
H -3.705036 0.842045 -4.535398
H 3.142780 -4.294503 0.135818

Table S62. Final coordinates for TS II-Uan-4U

U 0.355704494 -0.946335110 0.473051927
U -5.329403000 8.848148000 -3.876224000
Si -2.873883506 -0.320212110 2.799339927
Si -1.014025506 -4.383689110 -0.393170073
Si -0.096842506 1.895010890 -2.174137073
Si 2.555316494 -0.248218110 3.494191927
Si -2.782126000 7.642685000 -6.724133000
Si -8.129200000 8.883081000 -6.451532000
Si -5.026707000 5.826591000 -1.534671000
Si -3.050700000 11.802909000 -3.190166000
N -1.489028506 -0.193195110 1.751127927
N 0.306172494 -3.270139110 -0.156958073
N 0.470343494 0.348350890 -1.537559073
N 2.621484494 -1.642113110 -0.918393073
N 2.354460494 -0.559432110 1.787581927
N -3.670622000 7.883191000 -5.248352000
N -7.404361000 9.424851000 -4.961924000
N -5.863090000 7.209597000 -2.219233000
N -7.125932000 9.884698000 -2.075999000
N -4.439520000 10.823225000 -2.786859000
C 3.952634494 2.324874890 3.484834927
C 0.741001494 -0.351566110 5.798145927
C -1.692075506 0.368241890 -4.080151073
C -0.978814506 3.402744890 0.184464927
C 2.340639494 3.123984890 -1.142546073
C -2.743580506 2.002956890 -0.951779073
C -1.441516506 2.771432890 -1.121966073
C 1.173573494 4.474722890 -2.902273073
C 1.452881494 3.057361890 -2.391761073
C 0.057724494 -6.950805110 0.611822927
C -3.858860506 -4.294251110 0.306469927
C -0.792831506 -4.288658110 -3.330199073
C -3.067559506 -2.554190110 -1.326736073
C -2.669466506 -3.427273110 -0.129206073
C -2.069227506 -6.244526110 -2.393082073
C -0.932992506 -5.245019110 -2.140987073
C 1.633994494 -3.845022110 -0.375312073
C 2.449046494 -3.026178110 -1.374054073
C -4.555507506 -0.199234110 1.901281927
C -2.890752506 1.061662890 4.119335927
C -2.882953506 -1.984554110 3.718221927
C -0.902860506 -5.378007110 2.309493927
C -1.006498506 -5.874137110 0.864007927
C 4.538758494 -2.424112110 3.763852927
C 1.090111494 -2.504542110 4.539661927
C 1.035701494 -0.976278110 4.428559927
C -0.021982506 1.879409890 -5.171677073
C -0.918396506 1.686739890 -3.939965073
C 1.296748494 -0.331722110 -2.547589073
C 2.689704494 -0.694763110 -2.037856073
C 3.761371494 -1.509184110 -0.006305073
C 3.558667494 -0.364829110 0.982031927
C 1.448171494 2.392797890 3.354567927
C 2.653447494 1.642853890 3.931743927
C 4.239439494 -0.977421110 4.170024927
C 4.402608494 -0.801666110 5.685070927
C -1.504824000 11.572926000 -0.729253000
C -1.251910000 11.935860000 -5.504663000
C -7.318723000 4.247778000 -2.421404000
C -2.327254000 5.082217000 -2.413941000
C -5.140465000 6.549206000 1.337995000
C -4.127459000 4.610743000 -4.035428000
C -3.661308000 5.182264000 -2.717518000
C -3.251402000 7.469150000 0.013584000
C -4.188534000 6.263765000 0.166628000
C -9.348400000 11.335053000 -7.562417000
C -7.110261000 7.318321000 -8.711794000
C -10.199710000 7.315392000 -5.056277000
C -7.264352000 6.112488000 -6.511546000
C -7.036025000 7.471552000 -7.186491000
C -10.652163000 7.817643000 -7.481316000
C -9.977965000 8.315060000 -6.196222000
C -8.172534000 10.447153000 -4.249735000
C -8.396530000 10.073880000 -2.785479000
C -3.078934000 5.948538000 -7.559120000
C -0.891869000 7.763652000 -6.469234000
C -3.257436000 8.950939000 -8.019069000
C -6.915898000 10.968774000 -8.032807000
C -8.264114000 10.277575000 -7.808528000
C -4.660721000 14.281110000 -3.120618000
C -3.687801000 12.380249000 -5.945243000
C -2.688021000 11.611650000 -5.072519000
C -6.751042000 3.866575000 -0.007042000
C -6.190514000 4.250202000 -1.380418000
C -7.139355000 7.467178000 -1.542316000

C -7.236184000 8.891345000 -1.000519000
C -6.578798000 11.158399000 -1.596781000
C -5.056228000 11.121218000 -1.496162000
C -1.108129000 9.815366000 -2.480597000
C -1.437755000 11.292081000 -2.235909000
C -3.320214000 13.673174000 -2.693816000
C -2.160968000 14.584134000 -3.116318000
H 5.410015494 -1.094094110 6.019103927
H 4.241541494 0.234062890 6.004533927
H 3.691412494 -1.427077110 6.237761927
H 1.551065494 -0.530833110 6.516139927
H 5.559875494 -2.717479110 4.052649927
H 5.011256494 -0.351155110 3.696146927
H -0.172897506 -0.779764110 6.234376927
H 1.866871494 -2.828710110 5.243588927
H 0.593409494 0.731308890 5.737560927
H 3.853834494 -3.130432110 4.244200927
H 2.603132494 1.714429890 5.029936927
H 4.442549494 -2.570424110 2.684583927
H 0.138341494 -2.914283110 4.905322927
H 4.840056494 1.892521890 3.961006927
H 1.298090494 -2.971579110 3.571527927
H 0.191711494 -0.726917110 3.765802927
H 3.942585494 3.397422890 3.729657927
H 0.493223494 1.963460890 3.676160927
H -0.047374506 -7.783900110 1.323535927
H -1.950513506 -2.133173110 4.270372927
H 4.486792494 -0.307900110 1.578306927
H 1.070208494 -6.551337110 0.741701927
H 1.452443494 3.453392890 3.645674927
H 4.089777494 2.248847890 2.399858927
H 3.838733494 -2.438813110 0.566944927
H -0.962692506 -6.208631110 3.028961927
H -2.999694506 -2.820015110 3.021244927
H 2.205580494 -3.919311110 0.567643927
H 0.049095494 -4.862972110 2.475486927
H -0.000467506 -7.376577110 -0.396190073
H 1.462426494 2.351495890 2.259909927
H -3.714955506 -2.028542110 4.431157927
H 4.704902494 -1.388251110 -0.574247073
H 3.535070494 0.584211890 0.416743927
H 1.611843494 -4.875864110 -0.773079073
H -1.693037506 -4.665008110 2.564528927
H 3.432130494 -3.495989110 -1.573291073
H -2.012256506 0.992466890 4.769204927
H -1.989382506 -6.356548110 0.743340927
H -1.813421506 3.891641890 0.707097927
H -3.786208506 1.003505890 4.750170927
H -0.557091506 2.666080890 0.882638927
H 1.901123494 -3.008080110 -2.320087073
H -2.165755506 -6.981879110 -1.588383073
H -4.659183506 0.757837890 1.379628927
H 3.172155494 0.220462890 -1.682430073
H -1.385316506 0.809388890 1.548668927
H -2.880841506 2.052220890 3.650175927
H -4.653116506 -0.993788110 1.154007927
H -0.203200506 4.160226890 0.029219927
H -3.662266506 -4.843111110 1.233459927
H 3.317950494 -1.103755110 -2.853748073
H -2.443706506 -2.740078110 0.700272927
H -0.030765506 -3.525657110 -3.149050073
H -1.916615506 -6.800305110 -3.330692073
H -3.503748506 2.636491890 -0.470548073
H -5.392783506 -0.291502110 2.604488927
H -0.520641506 -4.829032110 -4.249181073
H 0.811648494 -1.252694110 -2.917455073
H -3.034735506 -5.732155110 -2.483224073
H 2.458075494 2.140057890 -0.679149073
H 1.911682494 3.785222890 -0.381340073
H -4.125449506 -5.033090110 -0.459355073
H -2.246509506 -1.912057110 -1.662152073
H -1.728520506 -3.759011110 -3.535223073
H -4.750305506 -3.674762110 0.476885927
H -2.622137506 1.109359890 -0.325554073
H -1.009433506 -0.486671110 -4.129712073
H -2.367012506 0.184431890 -3.240213073
H 1.461901494 0.273368890 -3.455802073
H -3.158161506 1.678240890 -1.911352073
H -3.383694506 -3.167266110 -2.179562073
H 3.339687494 3.519198890 -1.378505073
H -3.909582506 -1.897666110 -1.070752073
H 0.615483494 5.059479890 -2.162427073
H -2.293752506 0.359488890 -5.000968073
H 2.035361494 2.538455890 -3.170857073
H 0.715596494 1.073211890 -5.268125073
H -1.660444506 2.499456890 -3.959020073
H 0.592872494 4.489427890 -3.830570073
H 2.107464494 5.022125890 -3.098422073
H 0.529791494 2.823930890 -5.156085073
H -0.619973506 1.868750890 -6.094845073
H 0.001612494 -5.827212110 -2.093566073
H -2.271752000 15.596572000 -2.698646000
H -1.189439000 14.199431000 -2.787437000
H -2.116114000 14.694374000 -4.206370000
H -1.002087000 12.991222000 -5.339224000
H -4.805288000 15.279723000 -2.680113000
H -3.316672000 13.653720000 -1.592820000
H -1.114034000 11.739322000 -6.577482000
H -3.533371000 13.463692000 -5.866316000
H -0.509158000 11.337363000 -4.968082000
H -4.724021000 14.397737000 -4.207502000
H -0.617123000 11.898199000 -2.651880000
H -5.506333000 13.657470000 -2.817416000
H -3.578473000 12.119482000 -7.007106000

H -1.646299000 12.636942000 -0.505895000
H -4.723473000 12.169780000 -5.659617000
H -2.846798000 10.535688000 -5.249636000
H -0.582518000 11.252579000 -0.222628000
H -0.982232000 9.583435000 -3.543147000
H -9.394886000 12.053817000 -8.394730000
H -3.068447000 9.962106000 -7.647592000
H -4.756126000 12.098627000 -1.078101000
H -9.142012000 11.915327000 -6.655671000
H -0.185975000 9.514527000 -1.962348000
H -2.331078000 11.025316000 -0.261425000
H -6.846140000 11.930009000 -2.326040000
H -6.968613000 11.706858000 -8.847420000
H -4.317371000 8.879632000 -8.281373000
H -7.675817000 11.433869000 -4.289071000
H -6.594795000 11.496304000 -7.128536000
H-10.348156000 10.899903000 -7.453921000
H -1.918278000 9.176392000 -2.113892000
H -2.676857000 8.811450000 -8.938769000
H -7.038458000 11.440799000 -0.629002000
H -4.774387000 10.391330000 -0.715066000
H -9.177676000 10.623129000 -4.674204000
H -6.119126000 10.261121000 -8.282168000
H -9.021800000 10.829069000 -2.269933000
H -0.606391000 8.767009000 -6.135930000
H -8.531068000 9.749900000 -8.737669000
H -1.623348000 4.915336000 -3.231948000
H -0.346039000 7.546372000 -7.395724000
H -1.910765000 5.618072000 -1.563263000
H -8.943902000 9.127616000 -2.759984000
H -10.564727000 8.534218000 -8.305673000
H -2.750719000 5.122317000 -6.920044000
H -6.401958000 9.048155000 -0.310059000
H -3.235629000 7.277764000 -4.538728000
H -0.549946000 7.052150000 -5.709043000
H -4.142928000 5.800071000 -7.771827000
H -1.883853494 3.869796110 -1.879520927
H -6.837218000 8.237669000 -9.240001000
H -8.173348000 9.039909000 -0.428453000
H -6.008573000 7.776091000 -6.935256000
H -9.683773000 7.622568000 -4.142535000
H -11.724839000 7.626342000 -7.325772000
H -3.282882000 4.295090000 -4.659786000
H -2.530861000 5.874426000 -8.506957000
H -11.269294000 7.204510000 -4.822753000
H -7.997661000 7.292007000 -2.214623000
H -10.209355000 6.873933000 -7.821723000
H -5.802477000 5.713730000 1.573883000
H -5.770728000 7.422390000 1.134031000
H -8.117523000 7.038038000 -9.044708000
H -7.206119000 6.180831000 -5.420303000
H -9.822430000 6.320534000 -5.313035000
H -6.428187000 6.529150000 -9.057523000
H -4.708376000 5.342123000 -4.608931000
H -8.107039000 4.960751000 -2.157510000
H -6.967820000 4.517476000 -3.421263000
H -7.321521000 6.798346000 -0.681750000
H -4.771526000 3.732662000 -3.892435000
H -8.246052000 5.696862000 -6.769862000
H -4.568919000 6.781099000 2.248742000
H -6.509843000 5.382671000 -6.834405000
H -3.812790000 8.351172000 -0.314350000
H -7.788189000 3.255090000 -2.486052000
H -3.582388000 5.385032000 0.434837000
H -7.452610000 4.616722000 0.378275000
H -5.498111000 3.441752000 -1.658374000
H -2.463110000 7.311815000 -0.725922000
H -2.764081000 7.716255000 0.967713000
H -5.961345000 3.731985000 0.738800000
H -7.302277000 2.916556000 -0.066124000
H -10.491729000 9.245348000 -5.904853000

Table S63. Final coordinates for 4bUan

U -1.689174 0.032552 -1.602871
Si -2.818801 0.177839 2.246266
Si 1.902093 0.694253 -0.562221
Si -2.674935 -3.587631 -2.354108
Si -4.261224 2.733296 -2.309520
N -2.630849 -0.198992 0.557929
N 0.594635 0.760117 -1.717099
N -1.744705 -2.139941 -2.639481
N -0.857760 0.202158 -4.206754
N -3.080503 1.561175 -2.849669
C -6.663083 1.679167 -3.584134
C -4.925204 3.930237 0.285175
C -0.119437 -5.026750 -2.191535
C -4.566494 -4.010006 -0.226133
C -3.922837 -3.847885 -5.009549
C -2.260564 -3.664727 0.556952
C -3.295362 -3.714882 -0.540464
C -5.178129 -2.542742 -3.303518
C -4.232846 -3.733810 -3.510039
C 3.296826 3.258460 -0.983172
C 2.009429 0.061748 2.298451
C 3.287885 -1.464341 -2.013355
C 1.244002 -1.788865 0.781495
C 1.280377 -0.269202 0.989416
C 4.692318 -0.173926 -0.368338
C 3.502647 -0.107237 -1.334592
C 0.937349 1.400328 -2.989023
C 0.575429 0.523078 -4.186286
C -2.200575 -1.188465 3.434809
C -4.644875 0.468119 2.730725
C -1.848794 1.746597 2.701555

C 1.318063 3.285210 0.556174
C 2.494250 2.448736 0.043867
C -3.059909 4.863827 -3.960316
C -2.550697 4.205894 -0.501956
C -3.788095 3.300606 -0.529899
C -1.807928 -6.161980 -3.659586
C -1.616918 -5.244866 -2.447114
C -0.891322 -2.244215 -3.828512
C -1.123202 -1.105621 -4.817195
C -1.645776 1.267425 -4.838395
C -3.068941 1.311336 -4.290029
C -6.131686 0.806512 -1.289010
C -6.068885 2.026144 -2.213745
C -4.395526 4.234522 -3.549379
C -5.374765 5.318106 -3.080385
H -5.540343 6.075583 -3.861155
H -6.353371 4.905747 -2.811783
H -4.991042 5.848228 -2.200952
H -5.266038 4.877189 -0.150500
H -3.195671 5.596429 -4.770070
H -4.830150 3.791392 -4.459065
H -4.594916 4.151173 1.310072
H -2.772421 5.199375 -0.911474
H -5.798268 3.274200 0.360037
H -2.589320 5.392312 -3.124893
H -6.691972 2.820635 -1.773058
H -2.343783 4.113434 -4.306760
H -2.189141 4.359566 0.524287
H -6.716547 2.548129 -4.250018
H -1.724983 3.781891 -1.082679
H -3.516953 2.353799 -0.035900
H -7.683669 1.279794 -3.488239
H -5.778386 1.029607 -0.277421
H 3.643200 4.209568 -0.551801
H -2.116051 2.580855 2.046600
H -3.600029 2.080890 -4.876425
H 2.688194 3.512578 -1.858548
H -7.155693 0.413350 -1.206108
H -6.067439 0.911263 -4.091322
H -1.166128 2.222478 -4.602277
H 1.652500 4.258868 0.944023
H -0.770404 1.581837 2.616189
H 0.433273 2.376998 -3.102864
H 0.599954 3.477607 -0.247927
H 4.183434 2.724401 -1.342569
H -5.497674 0.000051 -1.673217
H -2.057668 2.043603 3.736067
H -1.640741 1.154512 -5.940128
H -3.574447 0.364379 -4.555627
H 2.013282 1.627328 -3.093971
H 0.765647 2.785714 1.358332
H 0.880552 0.998605 -5.138396
H -5.054718 1.345901 2.220845
H 3.164840 2.250499 0.895027
H -4.867119 -4.201342 0.806616
H -4.749794 0.625557 3.811314
H -5.359008 -4.083488 -0.965641
H 1.132461 -0.414149 -4.098799
H 4.917493 0.796293 0.087721
H -2.776502 -2.112643 3.316671
H -2.174956 -1.126455 -5.118537
H -3.354990 -0.894243 0.331390
H -5.265052 -0.393976 2.459107
H -1.148905 -1.426988 3.243527
H 1.964038 1.126699 2.547365
H -0.513608 -1.241318 -5.731802
H 0.235762 0.059596 1.100467
H 2.400244 -1.460977 -2.652246
H 5.605532 -0.515404 -0.878303
H -2.690110 -3.941379 1.527830
H -2.290403 -0.870242 4.481094
H 4.152703 -1.743824 -2.633040
H 0.178033 -2.256310 -3.555610
H 4.504016 -0.881424 0.448285
H -3.303805 -4.712561 -5.259532
H -3.403323 -2.954084 -5.372234
H 3.069340 -0.218128 2.254609
H 0.736642 -2.064060 -0.149329
H 3.147664 -2.264782 -1.279716
H 1.568282 -0.488991 3.141019
H -1.838077 -2.657985 0.650615
H 0.372874 -4.592489 -3.067939
H 0.068863 -4.347196 -1.355506
H -1.043235 -3.179529 -4.398131
H -1.426499 -4.346568 0.352832
H 2.256028 -2.210104 0.748135
H -4.852022 -3.929206 -5.592711
H 0.714218 -2.286513 1.604817
H -4.672459 -1.606967 -3.566720
H 0.386953 -5.978700 -1.974627
H -4.755413 -4.654253 -3.203677
H -1.417285 -5.709054 -4.578931
H -2.014390 -5.796528 -1.580986
H -5.515477 -2.437653 -2.269474
H -6.070964 -2.626830 -3.940059
H -2.860026 -6.411805 -3.833057
H -1.267109 -7.110262 -3.520719
H 3.778559 0.602879 -2.130763

References

1. Brown, J. L.; Gaunt, A. J.; King, D. M.; Liddle, S. T.; Reilly, S. D.; Scott, B. L.; Wooles, A. J. Neptunium and plutonium complexes with a sterically encumbered triamidoamine (TREN) scaffold. *Chem. Commun.* **2016**, *52*, 5428-5431.
2. Dutkiewicz, M. S.; Goodwin, C. A. P.; Perfetti, M.; Gaunt, A. J.; Griveau, J. -C.; Colineau, E.; Kovács, A.; Wooles, A. J.; Caciuffo, R.; Walter, O.; Liddle, S. T. A Terminal Neptunium(V)-Mono(Oxo) Complex *Nat. Chem.* **2022**, *14*, 342-349.
3. King, D. M.; Tuna, F.; McInnes, E. J. L.; McMaster, J.; Lewis, W.; Blake, A. J.; Liddle, S. T. Isolation and characterisation of a uranium(VI)-nitride triple bond. *Nat. Chem*. **2013**, *5*, 482-488.
4. Bergbreiter, D. E.; Killough, J. M. Reactions of potassium-graphite *J. Am. Chem. Soc.* **1978**, *100*, 2126-2134.
5. King, D. M.; Gardner, B. M.; Lewis, W.; Liddle, S. T. Uranium halide complexes stabilized by a new sterically demanding tripodal tris(N-adamantylamidodimethylsilyl)methane ligand. *J. Coord. Chem.* **2016**, *69*, 1893-1903.
6. Popovych, O. Conductometric Determination of Solubility and Solubility Products of Silver Salts in a Medium of Low Dielectric Constant. *Anal. Chem*. **1966**, *38*, 117-119.
7. O'Connor, A. R.; Nataro, C.; Golen, J. A.; Rheingold, A. L. Synthesis and reactivity of $[N(C_6H_4Br)_3][B(C_6F_5)_4]$: the X-ray crystal structure of $[Fe(C_5H_5)_2][B(C_6F_5)_4]$. *J. Organomet. Chem.* **2004**, *689*, 2411-2414.
8. Bruker *SAINT.* **2012**. Bruker AXS Inc., Madison, Wisconsin, USA.
9. Bruker *SADABS.* **2016**. Bruker AXS Inc., Madison, Wisconsin, USA.
10. CrysAlisPRO version 40.69, Oxford Diffraction /Agilent Technologies UK Ltd, Yarnton, England.
11. Sheldrick, G.M. A short history of SHELX. *Acta Cryst. Sect. A* **2008**, 112-122.

12. Sheldrick, G. M. SHELXT - Integrated space-group and crystal-structure determination. *Acta Cryst. Sect. A* **2015**, *71*, 3-8.

13. Sheldrick, G. M. Crystal structure refinement with SHELXL. *Acta Cryst. Sect. C* **2015**, *71*, 3-8.

14. Dolomanov, O. V.; Bourhis, L. J.; Gildea, R. J.; Howard, J. A. K.; Puschmann, H. OLEX2: a complete structure solution, refinement and analysis program. *J. Appl. Cryst.* **2009**, *42*, 339-341.

15. Farugia, L. J. WinGX and ORTEP for Windows: an update. *J. Appl. Cryst.* **2012**, *45*, 849-854.

16. Persistence of Vision (TM) Raytracer, Persistence of Vision Pty. Ltd., Williamstown, Victoria, Australia.

17. Speldrich, M.; Van Leusen, J.; Kögerler, P. CONDON 3.0: an updated software package for magnetochemical analysis—all the way to polynuclear actinide complexes. *J. Comput. Chem.* **2018**, *39*, 2133-2145.

18. Lahalle, M. P.; Krupa, J. C.; Guillaumont, R.; Rizzoli, C. Optical Spectroscopy of Np^{4+} ($5f^3$) Ion Diluted in $ThSiO_4$ and ThO_2 Crytsalline Hosts. *J. Less-Common Met.* **1986**, *122*, 65-73.

19. Schilder, H.; Speldrich, M.; Lueken, H.; Sutorik, A. C.; Kanatzidis, M. G. The Valence State of Uranium in $K_6Cu_{12}U_2S_{15}$. *J. Alloys Compd.* **2004**, *374*, 249-252.

20. Gatteschi, D.; Sessoli, R.; Villain, J. *Molecular Nanomagnets*. Vol. 5. Oxford University Press, USA, 2006.

21. Fonseca Guerra, C.; Snijders, J. G.; Te Velde, G.; Baerends, E. J. Towards an order-N DFT Method. *Theor. Chem. Acc.* **1998**, *99*, 391-403.

22. Te Velde, G.; Bickelhaupt, F. M.; Baerends, E. J.; Fonseca Guerra, C.; Van Gisbergen, S. J. A.; Snijders, J. G.; Ziegler, T. Chemistry with ADF. *J. Comput. Chem.* **2001**, *22*, 931-967.

23. Van Lenthe, E.; Baerends, E. J.; Snijders, J. G. Relativistic regular two-component Hamiltonians. *J. Chem. Phys.* **1993**, *99*, 4597-4610.

24. Van Lenthe, E.; Baerends, E. J.; Snijders, J. G. Relativistic total energy using regular approximations. *J. Chem. Phys.* **1994**, *101*, 9783-9792.

25. Van Lenthe, E.; Ehlers, A. E.; Baerends, E. J. Geometry optimization in the Zero Order Regular Approximation for relativistic effects. *J. Chem. Phys.* **1999**, *110*, 8943-8953.

26. Vosko, S. H.; Wilk, L.; Nusair, M. Accurate spin-dependent electron liquid correlation energies for local spin density calculations: a critical analysis. *Can. J. Phys.* **1980**, *58*, 1200-1211.

27. Becke, A. D. Density-functional exchange-energy approximation with correct asymptotic behaviour. *Phys. Rev. A.* **1988**, *38*, 3098-3100.

28. Perdew, J. P. Density-functional approximation for the correlation energy of the inhomogeneous electron gas. *Phys. Rev. B.* **1986**, *33*, 8822-8824.

29. Baker, C. F.; Seed, J. A.; Adams, R. W.; Lee, D.; Liddle, S. T. $^{13}C_{carbene}$ nuclear magnetic resonance chemical shift analysis confirms $Ce^{IV}=C$ double bonding in cerium(iv)–diphosphonioalkylidene complexes. *Chem. Sci.* **2024**, *15*, 238-249.

30. Du, J.; Seed, J. A.; Berryman, V. E. J.; Kaltsoyannis, N.; Adams, R. W.; Lee, D.; Liddle, S. T. Exceptional Uranium(VI)-Nitride Triple Bond Covalency from ^{15}N Nuclear Magnetic Resonance Spectroscopy and Quantum Chemical Analysis. *Nat. Commun.* **2021**, *12*, 5649.

31. Du, J.; Hurd, J.; Seed, J. A.; Baláẓs, G.; Scheer, M.; Adams, R. W.; Lee, D.; Liddle, S. T. ^{31}P Nuclear Magnetic Resonance Spectroscopy as a Probe of Thorium-Phosphorus Bond Covalency: Correlating Phosphorus Chemical Shift to Metal-Phosphorus Bond Order. *J. Am. Chem. Soc.* **2023**, *145*, 21766-21784.

32. Bader, R. F. W. *Atoms in Molecules: A Quantum Theory*, Oxford University Press, New York, 1990.

33. Bader, R. F. W. A bond path: a universal indicator of bonded interactions. *J. Phys. Chem. A* **1998**, *102*, 7314-7323.

34. Motta, L. C.; Autschbach, J. Actinide inverse trans influence versus cooperative pushing from below and multi-center bonding. *Nat. Commun.* **2023**, *14*, 4307.

35. Karlström, G.; Lindh, R.; Malmqvist, P.-Å.; Roos, B. O.; Ryde, U.; Veryazov, V.; Widmark, P. -O.; Cossi, M.; Schimmelpfennig, B.; Neogrady, P.; Seijo, L. MOLCAS: A Program Package for Computational Chemistry. *Comput. Mat. Sci.* **2003**, *28*, 222-239.

36. Aquilante, F.; Autschbach, J.; Carlson, R. K.; Chibotaru, L. F.; Delcey, M. G.; De Vico, L.; Galván, I. F.; Ferré, N.; Frutos, L. M.; Gagliardi, L.; Garavelli, M.; Giussani, A.; Hoyer, C. E.; Li Manni, G.; Lischka, H.; Ma, D.; Malmqvist, P. Å.; Müller, T.; Nenov, A.; Olivucci, M.; Pedersen, T. B.; Peng, D.; Plasser, F.; Pritchard, B.; Reiher, M.; Rivalta, I.; Schapiro, I.; Segarra-Martí, J.; Stenrup, M.; Truhlar, D. G.; Ungur, L.; Valentini, A.; Vancoillie, S.; Veryazov, V.; Vysotskiy, V. P.; Weingart, O.; Zapata F.; Lindh, R. MOLCAS 8: New Capabilities for Multiconfigurational Quantum Chemical Calculations Across the Periodic Table. *J. Comput. Chem.* **2016**, *37*, 506-541.

37. Roos, B. O. in Advances in Chemical Physics, Ab Initio Methods in Quantum Chemistry - II, Vol., 69 69, (Ed. K. P. Lawley), John Wiley & Sons Ltd., Chichester, pp. 399-446 (1987).

38. Douglas, N.; Kroll, N. M. Quantum Electrodynamical Corrections to the Fine Structure of Helium. *Ann. Phys.* **1974**, *82*, 89-155.

39. Hess, B. A. Relativistic Electronic-Structure Calculations Employing a Two-Component No-Pair Formalism with External-Field Projection Operators. *Phys. Rev. A* **1986**, *33*, 3742-3748.

40. Roos, B. O.; Lindh, R.; Malmqvist, P.-Å.; Veryazov, V.; Widmark, P. -O. New Relativistic ANO Basis Sets for Actinide Atoms. *Chem. Phys. Lett.* **2005**, *409*, 295-299.

41. B. O. Roos, R. Lindh, P.-Å. Malmqvist, V. Veryazov and P.-O. Widmark, Main Group Atoms and Dimers Studied with a New Relativistic ANO Basis Set, *J. Phys. Chem. A* **2004**, *108*, 2851-2858.

42. Widmark, P.-O.; Malmqvist P.-A.; Roos, B. O. Density matrix averaged atomic natural orbital (ANO) basis sets for correlated molecular wave functions. *Theor. Chim. Acta* **1990**, *77*, 291-306.

43. Spivak, M.; Vogiatzis, K. D.; Cramer, C. J.; De Graaf, C.; Gagliardi, L. Quantum Chemical Characterization of Single Molecule Magnets Based on Uranium. *J. Phys. Chem. A* **2017**, *121*, 1726-1733.

44. Gaggioli, C. A.; Gagliardi, L. Theoretical Investigation of Plutonium-Based Single-Molecule Magnets. *Inorg. Chem.* **2018**, *57*, 8098-8105.

45. Apostolidis, C.; Kovács, A.; Walter, O.; Colineau, E.; Griveau, J. -C.; Morgenstern, A.; Rebizant, J.; Caciuffo, R.; Panak, P. J.; Rabung, T.; Schimmelpfennig B.; Perfetti, M. Tris-{hydridotris(1-pyrazolyl)borato}actinide Complexes: Synthesis, Spectroscopy, Crystal Structure, Bonding Properties and Magnetic Behaviour. *Chem. Eur. J.* **2020**, *26*, 11293-11306.

46. Andersson, K.; Malmqvist, P.-Å.; Roos, B. O.; Sadlej, A.; Wolinski, K. Second-Order Perturbation Theory with a CASSCF Reference Function. *J. Phys. Chem.* **1990**, *94*, 5483-5488.

47. Andersson,, K.; Malmqvist P.-Å.; Roos, B. O. Second-Order Perturbation Theory with a Complete Active Space Self-Consistent Field Reference Function. *J. Chem. Phys.* **1992**, *96*, 1218-1226.

48. Roos, B. O. Malmqvist, P.-Å. Relativistic Quantum Chemistry: The Multiconfigurational Approach. *Phys. Chem. Chem. Phys.* **2004**, *6*, 2919-2927.

49. Gaussian 16, Revision B.01, Frisch, M. J.; Trucks, G. W.; Schlegel, H. B.; Scuseria, G. E.; Robb, M. A.; Cheeseman, J. R.; Scalmani, G.; Barone, V.; Petersson, G. A.; Nakatsuji, H.; Li, X.; Caricato, M.; Marenich, A. V.; Bloino, J.; Janesko, B. G.; Gomperts, R.; Mennucci, B.; Hratchian, H. P.; Ortiz, J. V.; Izmaylov, A. F.; Sonnenberg, J. L.; Williams-Young, D.; Ding, F.; Lipparini, F.; Egidi, F.; Goings, J.; Peng, B.; Petrone, A.; Henderson, T.; Ranasinghe, D.; Zakrzewski, V. G.; Gao, J.; Rega, N.; Zheng, G.; Liang, W.; Hada, M.; Ehara, M.; Toyota, K.; Fukuda, R.; Hasegawa, J.; Ishida, M.; Nakajima, T.; Honda, Y.; Kitao, O.; Nakai, H.; Vreven, T.; Throssell, K.; Montgomery, J. A.; Peralta, Jr., J. E.; Ogliaro, F.; Bearpark, M. J.; Heyd, J. J.; Brothers, E. N.; Kudin, K. N.; Staroverov, V. N.; Keith, T. A.; Kobayashi, R.; Normand, J.; Raghavachari, K.; Rendell, A. P.; Burant, J. C.; Iyengar, S. S.; Tomasi, J.; Cossi, M.; Millam,

J. M.; Klene, M.; Adamo, C.; Cammi, R.; Ochterski, J. W.; Martin, R. L.; Morokuma, K.; Farkas, O.; Foresman, J. B.; Fox, D. J. Gaussian, Inc., Wallingford CT, 2016.

50. Becke, A. D. Density functional thermochemistry. III. The role of exact exchange. *J. Chem. Phys.* **1993**, *98*, 5648-5652.

51. Burke, K.; Perdew, J. P.; Yang, W. in Electronic Density Functional Theory: Recent Progress and New Directions, Eds: J. F. Dobson, G. Vignale, M. P. Das, Plenum, New York, 1998.

52. Kuechle, W.; Dolg, M.; Stoll, H.; Preuss, H. Energy-adjusted pseudopotentials for the actinides. Parameter sets and test calculations for thorium and thorium monoxide. *J. Chem. Phys.* **1994**, *100*, 7535-7542.

53. Cao, X.; Dolg, M.; Stoll, H. Valence basis sets for relativistic energy-consistent small-core actinide pseudopotentials. *J. Chem. Phys.* **2003**, *118*, 487-496.

54. Cao, X.; Dolg, M. Segmented contraction scheme for small-core actinide pseudopotential basis sets. *J. Molec. Struct. (Theochem)* **2004**, *673*, 203-209.

55. Hollwarth, A.; Bohme, M.; Dapprich, S.; Ehlers, A.W.; Gobbi, A.; Jonas, V.; Kohler, K.F.; Stegmann, R.; Veldkamp, A.; Frenking G. A set of d-polarization functions for pseudo-potential basis sets of the main group elements Al Bi and f-type polarization functions for Zn, Cd, Hg. *Chem. Phys. Lett.* **1993**, *208*, 237-240.

56. Hehre, W. J.; Ditchfield, R.; Pople, J. A. Self-Consistent Molecular Orbital Methods. XII. Further Extensions of Gaussian-Type Basis Sets for Use in Molecular Orbital Studies of Organic Molecules. *J. Chem. Phys.* **1972**, *56*, 2257-2261.

57. McLean, A. D.; Chandler, G. S. Contracted Gaussian Basis Sets for Molecular Calculations. I. Second Row Atoms, Z = 11-18. *J. Chem. Phys.* **1980**, *72*, 5639-5648.

58. Gonzalez, C.; Schlegel, H. B. An Improved Algorithm for reaction Path Following. *J. Chem. Phys.* **1989**, *90*, 2154-2161.

59. Gonzalez, C.; Schlegel, H. B. Reaction Path Following in Mass-Weighted Internal Coordinates. *J. Phys. Chem.* **1990**, *94*, 5523-5527.
